# One-Metal/Two-Ligand for Dual Activation Tandem Catalysis: Photoinduced Cu-Catalyzed Anti-hydroboration of Alkynes

**DOI:** 10.1021/jacs.2c05805

**Published:** 2022-07-05

**Authors:** Javier Corpas, Miguel Gomez-Mendoza, Jonathan Ramírez-Cárdenas, Víctor A. de la Peña O’Shea, Pablo Mauleón, Ramón Gómez Arrayás, Juan C. Carretero

**Affiliations:** † Departamento de Química Orgánica and Centro de Innovación en Química Avanzada (ORFEO-CINQA), Facultad de Ciencias, Universidad Autónoma de Madrid (UAM), 28049 Madrid, Spain; ‡ Photoactivated Processes Unit, IMDEA Energy Institute, Technological Park of Mostoles, Avda. Ramón de la Sagra 3, 28935 Madrid, Spain; § Institute for Advanced Research in Chemical Sciences (IAdChem), UAM, 28049 Madrid, Spain

## Abstract

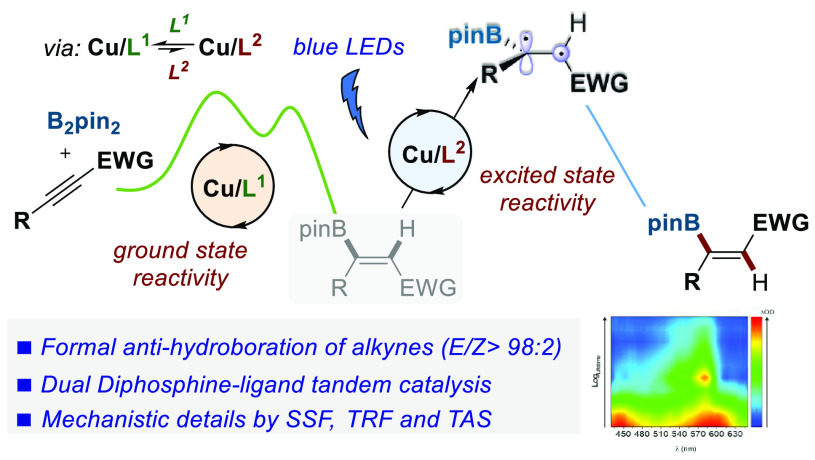

A dual catalyst system based on ligand exchange of two diphosphine ligands possessing different properties in a copper complex has been devised to merge metal- and photocatalytic activation modes. This strategy has been applied to the formal anti-hydroboration of activated internal alkynes via a tandem sequence in which Cu/Xantphos catalyzes the B_2_pin_2_-*syn*-hydroboration of the alkyne whereas Cu/BINAP serves as a photocatalyst for visible light-mediated isomerization of the resulting alkenyl boronic ester. Photochemical studies by means of UV–vis absorption, steady-state and time-resolved fluorescence, and transient absorption spectroscopy have allowed characterizing the photoactive Cu/BINAP species in the isomerization reaction and its interaction with the intermediate *syn*-alkenyl boronic ester through energy transfer from the triplet excited state of the copper catalyst. In addition, mechanistic studies shed light into catalyst speciation and the interplay between the two catalytic cycles as critical success factors.

## Introduction

The wealth of knowledge on transition metal/ligand (M/L) interaction has allowed tailoring reactivity and selectivity of catalytic processes and has guided the rational discovery of new reactivity profiles.^1^ However, transformations involving cascade reactions do not easily fit into this single site M/L paradigm, especially when distinct mechanisms are into play. Bimetallic catalysis has emerged as a viable solution to this challenge exploiting two catalytic cycles, each one promoted by an optimal M/L combination (M^1^L^1^/M^2^L^2^).^2^ A simpler strategy allowing distinct mechanisms to operate in tandem is to use a single metal in combination with two ligands (ML^1^/L^2^), each one with a specific role to induce the desired reactivity to the metal. Despite impressive recent accomplishments,^3^ the full potential of this concept is yet to be revealed.

**Scheme 1:**
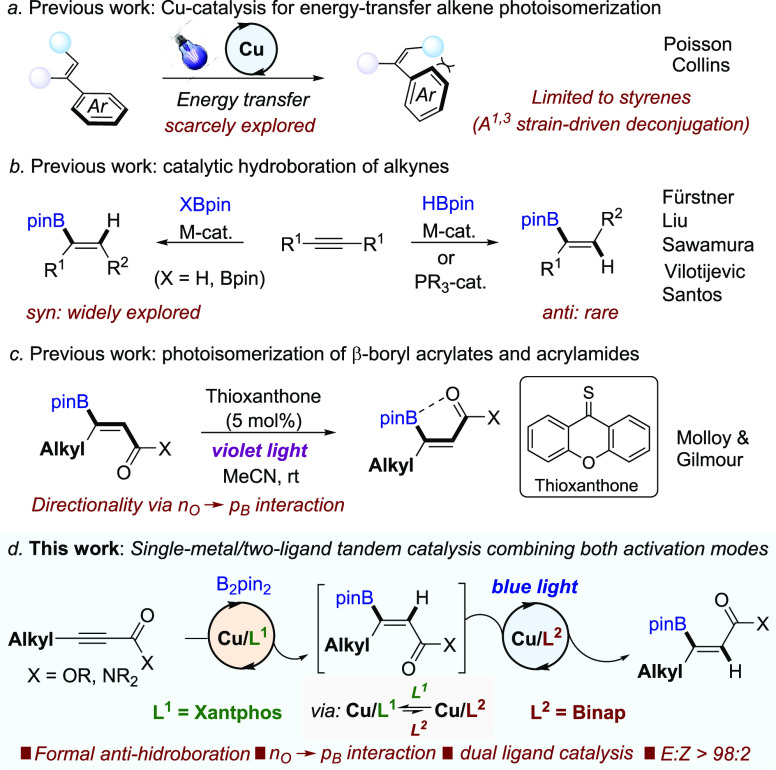
Sources of Inspiration for This Work

Visible light-mediated Cu^I^ photocatalysts are receiving increasing attention as cost-effective alternatives to the conventional Ru or Ir catalysts while still enabling easy fine-tuning of the excited-state properties through ligand modification.^4^ Homoleptic Cu(phen)^2+^, and heteroleptic cationic Cu^I^ complexes with N- and P-bidentate ligands have proved to be efficient photocatalysts.^4,5^ However, for this class of coordinatively saturated complexes to serve, not only as photosensitizers (PSs) but also as catalysts for inner sphere bond-formation, a single electron transfer (SET) from the photoexcited (Cu^I^)* is required to generate a Cu^II^ species having new available coordination sites for substrate coordination. Recently, this photoexcitation/SET reactivity mode has also been applied to visible light absorbing LCu^I^–substrate complexes.^6^ Less developed is the use of Cu-based PSs in energy-transfer (E_*n*_T) catalysis mediated by visible light, pioneered by Collins,^7^ building on the discovery by McMillin that Cu complexes of the type Cu(NN)(PP)X can have unprecedentedly long excited-state lifetimes.^8^ Its application to the isomerization of alkenes has been recently exploited (Scheme 1a). Poisson has reported that Cu^II^/BINAP complexes function as dual-functional photo/Lewis acid catalysts for (E_*n*_T)-induced *E* → *Z* isomerization of activated alkenes upon bidentate complexation to Cu.^9^ Collins used heteroleptic Cu complexes such as Cu(bphen)(Xantphos)BF_4_ as PSs for isomerization of a range of di- and trisubstituted alkenes, including 1,3-enynes.^10^ However, the use of these Cu PSs for the modulation of olefin geometry is yet limited to styrene-type alkenes, for which deconjugation of the aromatic unit with the olefin driven by A^1,3^ interaction ensures directionality.^11^

We questioned whether it would be possible to develop a non-SET tandem relay catalytic process combining the PS ability of Cu^I^ complexes with their efficient participation in polar (bielectronic) mechanisms. This goal should be attained by careful ligand selection to (i) prevent saturating the metal coordination sphere to allow substrate coordination and (ii) match each catalytic cycle while ensuring catalyst compatibility. Guided by our interest in alkyne functionalization,^3c,12^ we selected as an ideal platform to test our hypothesis the formal anti-hydroboration of internal alkynes through a tandem sequence involving *syn*-selective Cu^I^-catalyzed B_2_pin_2_-hydroboration and subsequent alkene isomerization via E_*n*_T catalysis.

The catalytic hydroboration of internal alkynes generally provides trisubstituted alkenyl boronic esters with *syn*-addition stereochemistry, which is dictated by the *syn*-insertion of B–M species across the alkyne (Scheme 1b, left).^13^ Only a handful of methods enabling direct access to the opposite anti-stereochemistry have been devised (Scheme 1b, right). Precious-metal-based catalysts (Ru,^14^ Pd^15^ or Au)^16^ have been used for symmetrical internal alkynes, 1,3-enynes, and propargyl amines, respectively. Additionally, metal-free protocols have been developed for anti-hydroboration of alkynoic acid derivatives with HBpin catalyzed by trialkylphosphines (typically PBu_3_).^17^ The anti-hydroboration of NH propiolamides mediated by stoichiometric amounts of strong bases (typically BuLi) has also been reported.^18^ An important drawback from these methods is the modest reactivity, limited scope, and incomplete anti-stereoselectivity with alkyl-substituted alkynes. The use of PMe_3_ instead of PBu_3_ goes some way to addressing this limitation,^17a^ but PMe_3_ is an expensive reagent, pyrophoric, has an unpleasant odor, and is toxic. Furthermore, none of these methods has demonstrated to be amenable to the diversification of complex multifunctional molecules. Clearly, new *anti*-hydroboration methods complementing the existing ones are needed to expand the current scope.

Recently, the group of Gilmour has reported the (E_*n*_T)-induced *E* → *Z* isomerization of trisubstituted β-borylacrylic acid derivatives using thioxanthone as the photocatalyst, where the directionality is controlled by a non-covalent n_O_ → p_B_ interaction between a carbonyl group and a boron atom which disrupts conjugation between the olefin and the carbonyl moiety by C(sp^2^)–B bond rotation (Scheme 1c).^19a,20^

Herein, we disclose the realization of our goal, namely the Cu-catalyzed formal anti-hydroboration of β-alkyl-substituted propiolates and propiolamides through the cooperative action of two different P ligands (Scheme 1d). This protocol provides excellent anti-stereocontrol, is tolerant of functionalized heterocyclic ring systems, and enables preserving the stereochemical integrity of easily enolizable chiral α-amino acid derivatives, a class of substrates that remains unexplored in this reaction. Furthermore, it allows modification of alkynes embedded in complex molecules. Mechanistic studies suggest that the interplay between the two catalytic cycles is essential for the generation of the required photoactive Cu species. Importantly, to the best of our knowledge, this ML^1^/L^2^ strategy has not yet been harnessed to merge metal- and triplet E_*n*_T photocatalytic activation modes.

## Results and Discussion

Although the Cu-catalyzed *syn*-hydroboration of alkynes activated by electron-withdrawing groups had been documented,^21^ at the outset, it was unclear whether the requisite isomerization of the intermediate β-borylacrylate could be achieved using Cu-based PSs. To probe this, a set of photoactive Cu^I^ complexes (10 mol %) were tested in the isomerization of boronic ester *Z*-**2a** under blue light irradiation in THF for 24 h (Table 1; see Supporting Information for complete studies). Neither heteroleptic ([Cu(phen)(BINAP)]PF_6_ or [Cu(bphen)(Xantphos)]BF_4_) nor homoleptic [Cu(BINAP)_2_PF_6_] complexes were effective, resulting in the exclusive recovery of the starting material (entries 1–3).^22^ These results are likely due to the very different triplet energies of the β-borylacrylate nature of **2a**, compared to styrene-like substrates previously studied. Based upon the idea that an open coordination site on the Cu center (available upon ligand dissociation) could facilitate isomerization through metal–substrate interaction, monophosphine complexes of the type Cu(phen)(PR_3_)Cl were tested, which also were found to be essentially inactive (entry 4).

**Table 1:**
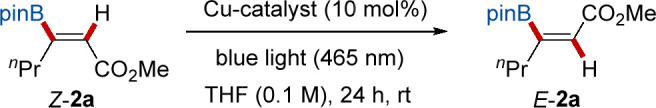

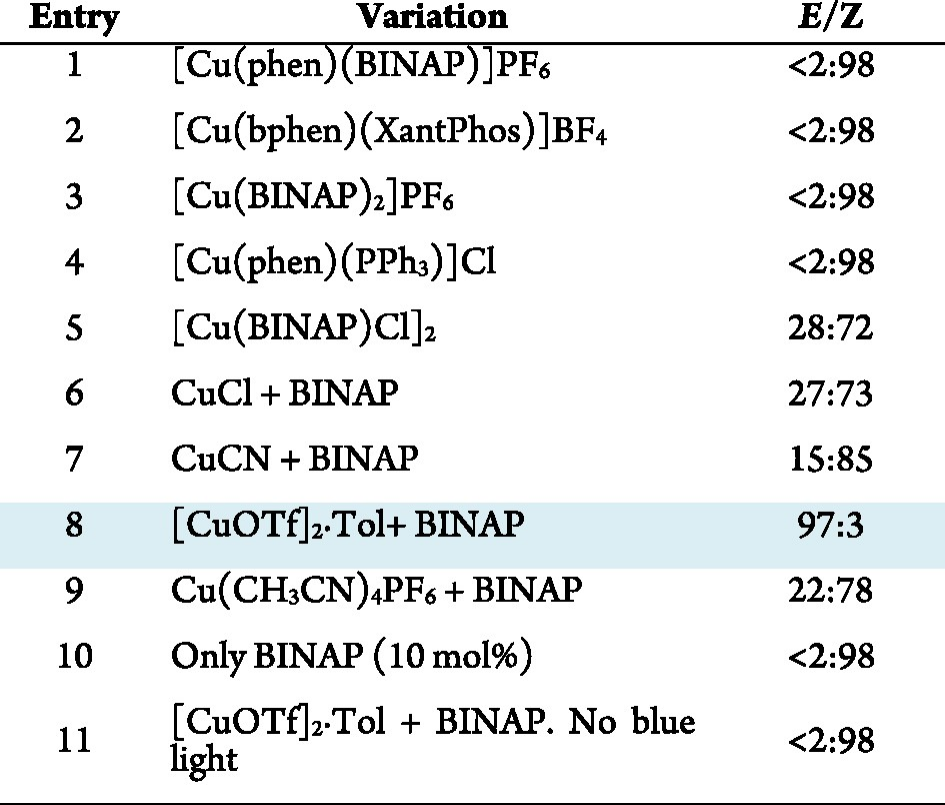
Optimization Studies for Isomerization of Alkenyl Boronic Ester *Z*-**2a**^a^

a Determined in the crude reaction by ^1^H NMR (1,3,5-trimethoxy-benzene was used as an internal standard).

However, the dimer [Cu(BINAP)Cl]_2_ showed some catalytic activity (*E*/*Z* = 28:72, entry 5), which could be ascribed to the easier generation of a free coordination site for substrate coordination via thermal dissociation (similar result was obtained combining 10 mol % of CuCl and BINAP, entry 6). In line with this hypothesis, our attention was shifted to modifying the nature of the counteranion of copper. Although stronger Lewis bases such as CN^–^ proved detrimental to reactivity (entry 7), the use of the more cationic Cu(OTf)_2_·toluene complex resulted in almost quantitative isomerization (*E*/*Z* = 97:3, entry 8). This result is in accordance with reported studies showing that the most non-coordinating counteranions can lead to an increase in the lifetime or triplet excited state of the chromophore, thus favoring the E_*n*_T process.^23^ In contrast, the highly cationic Cu(CH_3_CN)_4_PF_6_ complex displayed a low catalytic activity (*E*/*Z* = 22:78, entry 9). We speculated that in this case the cationic copper is better able to coordinate two ligand units, forming the inactive complex [Cu(BINAP)_2_PF_6_] in the reaction medium. A series of bidentate phosphine ligands with varied steric and electronic properties were then studied, all of them showing lower performance than BINAP (not shown, see the Supporting Information). The catalytic activity was negatively influenced by changes in solvent, with THF being the most effective one (toluene, CH_2_Cl_2_, CH_3_CN, or C_6_F_6_ were less effective, see the Supporting Information for details). Finally, the isomerization was not observed when either copper or blue light irradiation was not present (entries 10 and 11, respectively).

Having established a viable Cu catalyst for photoisomerization of trisubstituted alkenyl boronic esters, we examined the possibility of integrating the present system within a Cu-catalyzed B_2_pin_2_-borylation of internal alkynes.^13,21^ Although both processes are catalyzed by the same metal, we anticipated that the disparate electronic requirements of the ligand needed for each reaction might benefit from using simultaneously two ligands with different electronic characteristics. In such a dual ligand system, achieving orthogonal reactivity of the CuL^1^/CuL^2^ catalysts so that both operate in tandem without negative interferences poses a key challenge. Not unsurprisingly, when BINAP was used as the only ligand in the Cu-catalyzed model reaction between **1a** and B_2_pin_2_ under blue light irradiation for 24 h, the desired alkenyl boronic ester *E*-**2a** (the product from formal anti-addition) was obtained with almost complete stereoselectivity, albeit with a very low yield (15%, Table 2, entry 1). This result suggests that whereas the BINAP is highly effective for promoting the photoisomerization of the newly formed alkenyl boronic ester, it is largely inefficient in the alkyne borylcupration step, for which a stronger σ-electron-donating ancillary ligand is typically required.^24^ In an effort to enhance the borylation conversion, we explored the effect of an additional ligand in the catalyst system. The presence of PCy_3_ favored borylcupration, but it came at the cost of a complete lack of photoisomerization reactivity (entry 2). Minor improvements in isomerization were realized when less bulky P(*p*-MeOC_6_H_4_)_3_ or PBu_3_ was employed (entries 3 and 4). This observation is plausibly ascribed to the ligands forming species of the type Cu(BINAP)(PR_3_), which would hinder the coordination of the metal to both -Bpin and alkyne substrate, rather than engaging in two copper complexes with orthogonal reactivity. Therefore, our attention was shifted to the effect of bidentate phosphine ligands, in particular Xantphos because of its stronger back-donation ability, which is considered to be important for the addition of borylcopper(I).^25^ To our delight, this ligand provided a dramatic increase in the catalytic activity of both processes, leading to the desired *E*-**2a** in 67% yield with a promising stereoselectivity (*E/Z* = 80:20, entry 5). Through systematic screening, we were pleased to observe that the stereoselectivity could be increased when the reaction was performed at a lower concentration (0.05 M, entry 6). We attribute this dilution effect to the low solubility of BINAP in THF. To our delight, further studies demonstrated that yield increased to 78%, accompanied by complete isomerization (*E/Z* = >98:2) when a slightly higher amount of B_2_pin_2_ (1.5 equiv, entry 7) was employed. The use of DPEphos, with a more flexible backbone and slightly lower bite angle, and the related bulkier ^*t*^Bu_2_-Xantphos provided lower performance (entries 8 and 9, respectively). Finally, shorter reaction times (12 h) had minimal impact on yield but was detrimental for isomerization (entry 10), suggesting that the latter step requires prolonged reaction times. We proved that the presence of both the BINAP ligand and blue light were crucial to promoting the isomerization reaction (entries 11 and 12). Finally, the presence of the copper salts was required to promote the borylation reaction (entry 13).

**Table 2:**
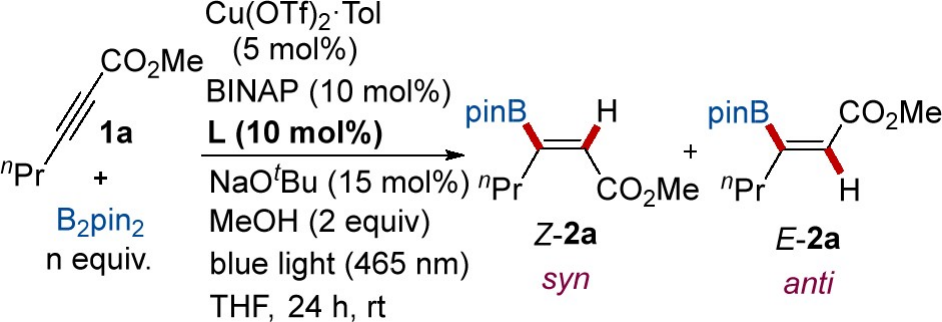

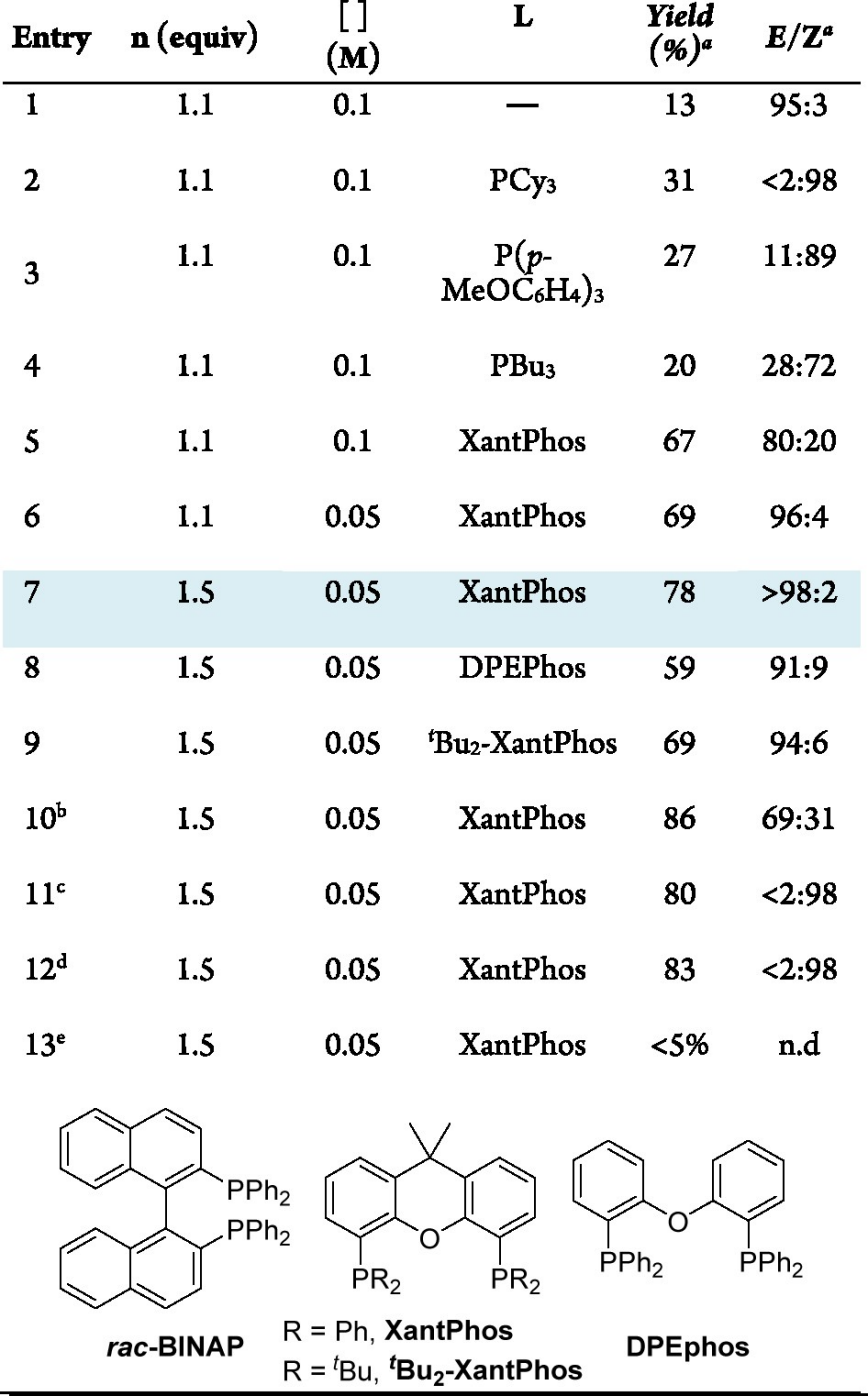
Optimization Studies for Isomerization of Alkenyl Boronic Ester **1a**

a Determined in the crude reaction by ^1^H NMR spectroscopy (1,3,5-trimethoxy-benzene was used as an internal standard).

b Reaction run for 12 h.

c No BINAP ligand was added.

d No blue light irradiation.

e No copper salt was added.

**Scheme 2:**
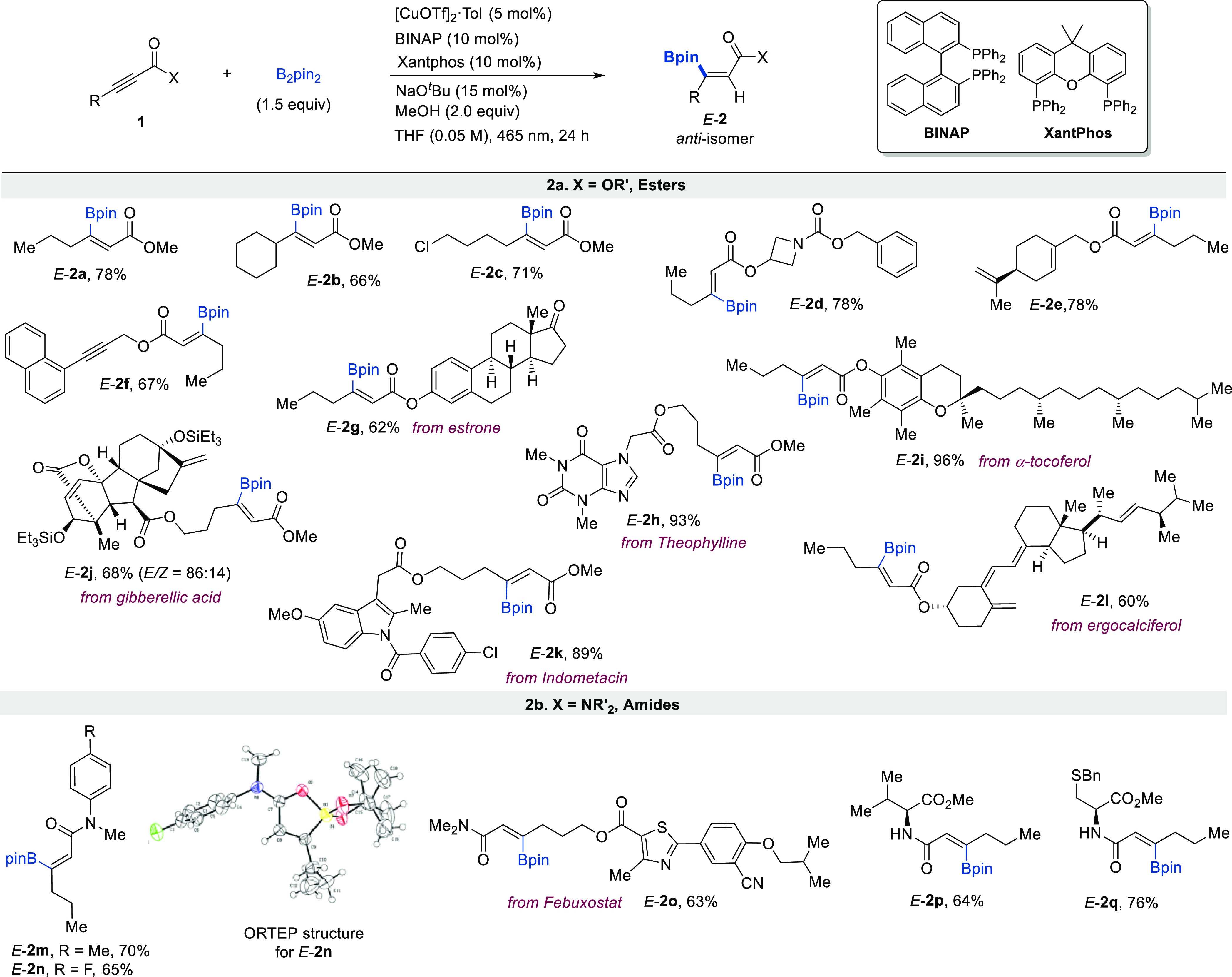
Reaction Scope Unless otherwise noted, *E/Z* > 98:2 (determined by ^1^H NMR in the crude reaction). Reaction yields after purification by flash column chromatography.

The generality and robustness of this transformation were evaluated next (Scheme 2). A diversity of propiolate-type alkynes proved to be efficient participants in this transformation, revealing wide tolerance toward the variation of the substituent at both the alkyne and the ester units (Scheme 2a). The reaction tolerates alkynes bearing branching at the propargylic position (*E*-**2b**, for which low stereocontrol has been previously observed),^17a,17c^ as well as the presence of sensitive functional groups such as aliphatic chlorides (*E*-**2c**). Non-activated alkenes (*E*-**2e**) and alkynes (*E*-**2f**) are not as reactive and remain intact after the transformation, evidencing the importance of the proximal ester group, likely due to metal coordination. The versatility of the reaction is best exemplified by the anti-borylation of alkynes embedded in complex molecules, thus also showing its potential to rapidly change the properties of existing compounds having biological properties (*E-***2g**-**2l**). The reaction is tolerant of functionalized heterocyclic ring systems such as azetidine (*E*-**2d**) indole (*E*-**2k**), thiazole (*E*-**2o**), or xanthine (a purine base, *E*-**2h**). Aryl chlorides (*E*-**2k**) and enolizable aliphatic ketones (*E*-**2g**) were also compatible.

Alkynyl amides were also amenable to the reaction, providing comparable reactivity and levels of regio- and stereocontrol (Scheme 2b, *E*-**2m**-**2q**). For the fluorinated amide product *E*-**2n**, suitable crystals for X-ray diffraction analysis could be obtained, showing a strong interaction between the amide carbonyl and the boron atom (O–B bond distance 1.735 Å) with virtually no conjugation between the Bpin moiety and the C–C double bond. These observations are in complete agreement with previous X-ray studies on β-borylacrylamides performed by the groups of Santos^18^ and Gilmour.^19^ Interestingly, mildly acidic N–H bonds of enantiopure α-amino esters derived from l-valine (*E*-**2p**) and l-Bn-cysteine (*E-***2q**) were well tolerated, affording the corresponding products with no erosion of the enantiomeric purity. Importantly, this class of substrates remains unexplored in this reaction.

### Photochemical Studies

**Figure 1:**
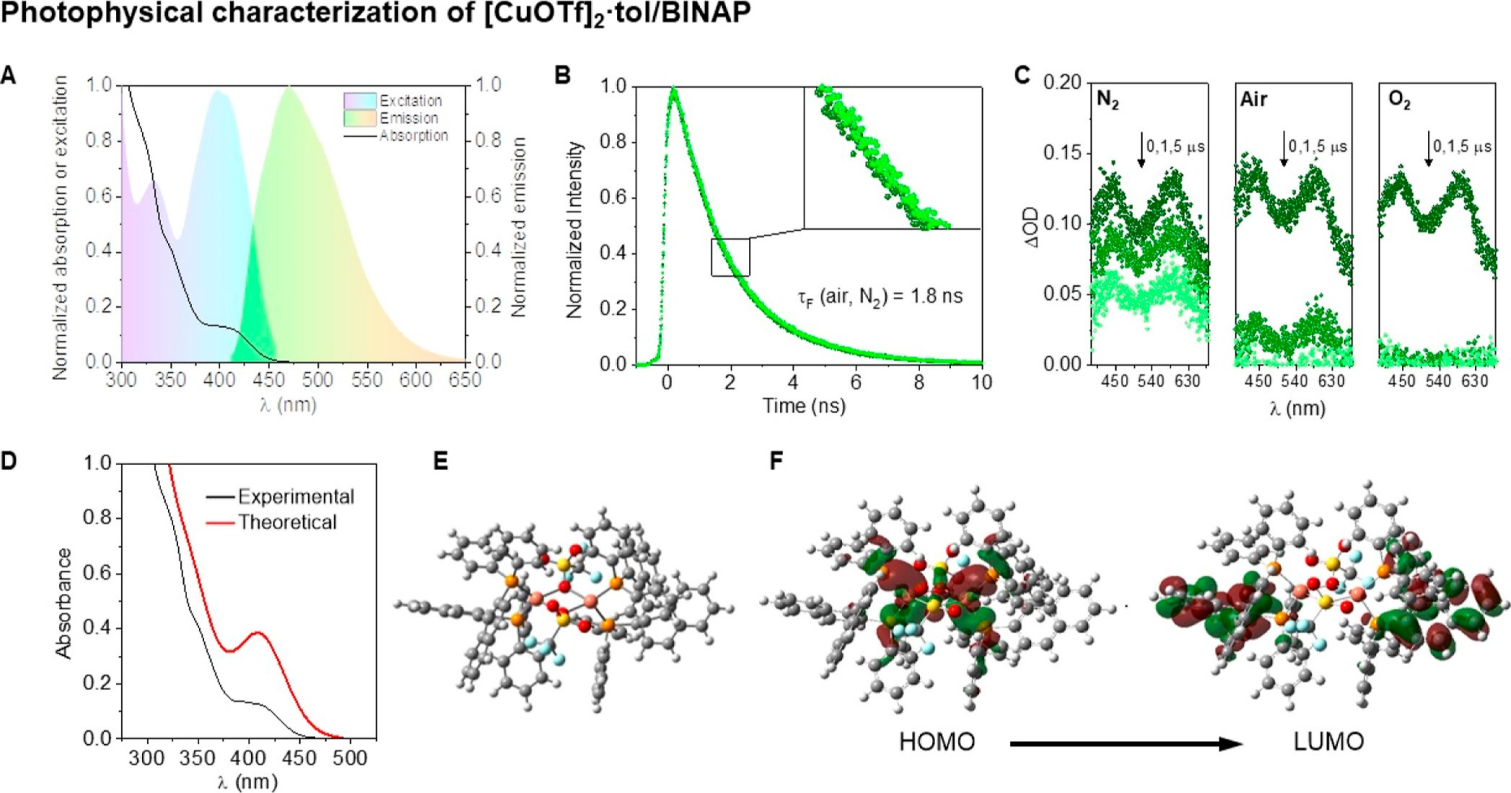
Photophysical characterization of [CuOTf]_2_·tol/BINAP in acetonitrile. (A) Normalized absorption (black line), excitation (violet-blue), and fluorescence (λ_exc_ = 400 nm, green) spectra for [CuOTf]_2_·tol/BINAP (20 μM) in acetonitrile. (B) Time-resolved fluorescence of [CuOTf]_2_·tol/BINAP (20 μM) in aerated (green olive) or deaerated (green) acetonitrile. Inset: zoom image. (C) Transient absorption spectra (λ_exc_ = 355 nm) for [CuOTf]_2_·tol/BINAP (20 μM) in acetonitrile at different timescales (0, 1, and 5 μs) after laser pulse in an aerated and purged (by N_2_ or O_2_) atmosphere. (D) Experimental (black) and calculated (red) UV–vis absorption spectra for [Cu(BINAP)(OTf)]_2_. (E) Computed geometry of [Cu(BINAP)(OTf)]_2_ complex. (F) HOMO and LUMO orbitals for the [Cu(BINAP)(OTf)]_2_ complex.

For a better understanding on the excited state of the Cu/BINAP complex and its kinetic implications on the overall reaction pathways, UV–vis absorption, steady-state and time-resolved fluorescence (SSF and TRF, respectively), and transient absorption spectroscopy (TAS) experiments were performed. Initially, we studied the photophysical properties of the complex formed in situ from [CuOTf]_2_·toluene and BINAP in a 1:1 [Cu]/BINAP ratio, in CH_3_CN to ensure complete solubility of the complex (Figure 1). The UV–vis spectrum of this species depicts a broad shoulder at ∼400 nm, whereas the fluorescence spectrum showed a maximum at ∼470 nm and presents a molar extinction coefficient (ε) of 6460 M^–1^ cm^–1^ (Figure 1A). A singlet excited-state energy (E_s_) value of 66 kcal·mol^–1^, with a fluorescence lifetime (τ_S_) of 1.8 ns (Figure 1B), and a poor fluorescence quantum yield (ϕ_F_) of 0.09 in acetonitrile were determined. TAS measurements for this Cu/BINAP species, under an inert atmosphere, revealed two main TA bands at 450 and 600 nm, respectively (Figure 1C), that presents a first-order kinetic with a transient lifetime (τ) of 10 μs (see Supporting Information, Figure S17). Quenching experiments by molecular oxygen confirmed the triplet nature of the observed transient (Figure 1C),^26^ with lifetimes of τ = 850 and 138 ns in aerated and purged O_2_ solutions, respectively, and a quenching constant of *k*_q_ = 8 × 10^8^ M^–1^ s^–1^ (see Figure S17d in Supporting Information for details). The calculated intersystem crossing quantum yield (ϕ_ISC_) was 0.91 (from 1–ϕ_F_).^27^ Because the formation of dimers of the type [Cu(BINAP)X]_2_ is known to be present in solution from copper(I) salts and BINAP, we studied the photophysical properties of [Cu(BINAP)(OTf)]_2_ by means of TD-DFT studies, showing a good agreement with experimental UV–vis spectra (Figure 1D).^28^ In addition, the higher intense energy electronic transition is attributed to a (metal + ligand) to ligand charge transfer [(M+L)LCT] process^26c^ from HOMO and HOMO–1 to LUMO, LUMO+1 orbital, thus explaining the origin of the observed luminescence.

**Figure 2:**
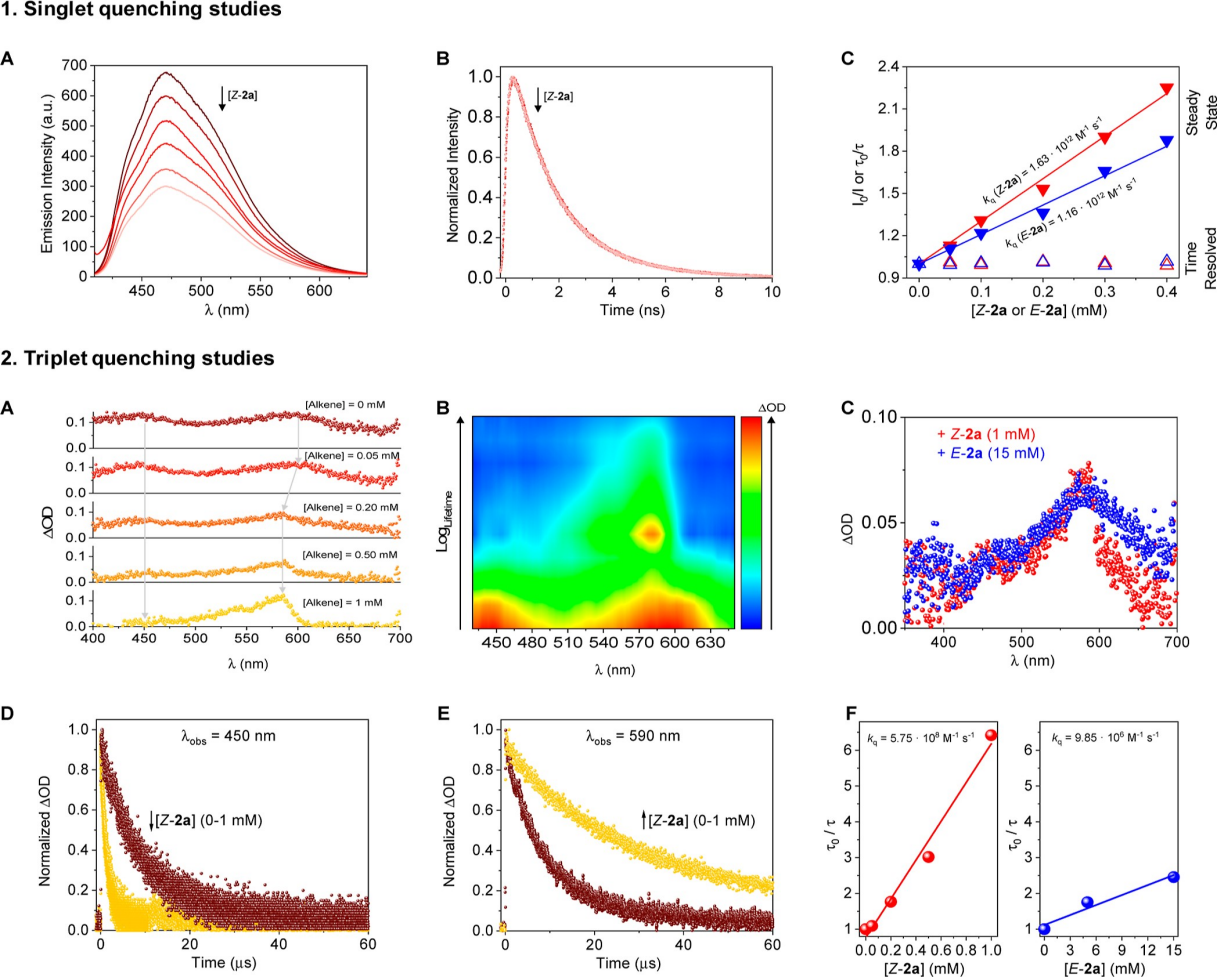
Quenching studies of [CuOTf]_2_·tol/BINAP (PS) with model substrate **2a**. 1: (A) Fluorescence emission (λ_exc_ = 400 nm) and (B) decay traces (λ_exc_ = 445 nm, band pass filter centered at 500 nm) for PS (20 μM) upon addition of increasing concentrations of *Z*-**2a** (up to 400 μM) in acetonitrile. (C) Stern–Volmer plots for steady-state (solid triangles) or time-resolved (empty triangles) quenching fluorescence experiments by addition of the *Z*-**2a** (red) or *E-***2a** (blue). 2: (A) Transient absorption spectra (TAS, λ_exc_ = 355 nm) for PS (20 μM) upon addition of increasing concentrations of *Z*-**2a** in acetonitrile monitored after 3 μs laser pulse under N_2_. The gray line indicates the maxima band position for ^3^[Cu/BINAP]* absorption. (B) TAS mapping (λ_exc_ = 355 nm) for PS (20 μM) in the presence of 5 mM of *Z*-**2a** at different timescales under N_2_. (C) Transient comparative (λ_exc_ = 355 nm) for PS (20 μM) in the presence of 1 mM of *Z*-**2a** (red) or 15 mM *E*-**2a** (blue) under N_2_. (D,E) Decay traces comparison (λ_exc_ = 355 nm) for PS (20 μM) in the presence of 1 mM of *Z*-**2a** after monitoring at λ_mon_ = 450 (c) or 590 nm (d) pulse under N_2_. (F) Stern–Volmer plots for PS (20 μM) upon addition of increasing concentrations of *Z*-**2a** (red) or *E*-**2a** (blue).

To determine the interaction between the excited states of PS Cu/BINAP and the alkene substrate, quenching experiments were performed employing model substrate **2a** (Figure 2). UV–vis absorption and fluorescence spectroscopies confirm the negligible overlapping between *Z*-**2a** or *E-***2a** at 300 μM and the Cu/BINAP bands (see Supporting Information, Figures S18 and S19). Nevertheless, we performed quenching experiments above 400 nm to avoid potential inner interferences from alkene absorption/emission.^29^

Regarding the singlet fluorescence quenching studies of the Cu/BINAP system by model substrate *Z-***2a**, Stern–Volmer experiments showed a highly efficient quenching in the emission for Cu/BINAP upon increasing addition of the *syn*-isomer (*Z*-**2a**) (*k*_q_ = 1.63 × 10^12^ M^–1^ s^–1^, Figure 1A). In addition, no changes were observed in the band maximum of the emission spectra with the increase in the isomer concentration (Figure 1A).^30^ Surprisingly, no changes on the emission decay traces were observed by excitation of the Cu/BINAP complex at 445 nm, revealing a static fluorescence mechanism quenching^31^ due a potential binding interaction between the Cu complex and the boronic ester in the ground state (Figure 1B). A similar behavior was observed when quenching studies were performed with the corresponding anti-isomer *E*-**2a** with *k*_q_ = 1.16 × 10^12^ M^–1^ s^–1^ (Figure 1C, see Supporting Information for details), suggesting that Cu/BINAP and both isomers of boronic ester **2a** can interact in the ground state, ruling out the possibility of quenching between the singlet excited state (S_1_) of the Cu/BINAP complex and model substrate **2a**.

Then, triplet quenching studies were performed by means of TAS (Figure 22).^32^ In this regard, we observed in the TA spectrum a continuous decrease of the triplet transient from the PS (Cu/BINAP) between 400 and 500 nm upon addition of *Z*-**2a**, concomitantly with a growth in the 530–600 nm range, with increasing alkene concentrations (Figure 2A,B) with no interference by alkene absorption at 355 nm (the transient negative band detected for the alkene isomers showed τ = 10 ns, which was residual compared to ^3^τ* for Cu/BINAP (10 μs), see the Supporting Information for details). More interestingly, a new transient band with a maximum at 585 nm (Figure 2A,B) was observed. This new photogenerated species showed a significant lifetime increment of 3 times compared to the corresponding ^3^Cu/BINAP* band without alkene (Figure 2C,D). This signal could be attributed to the excited state of the intermediate ^3^[CuBINAP]*@alkene, which promotes the photosensitization of the alkenyl boronic ester within the inner coordination sphere and the subsequent *Z* → *E* isomerization. Because direct excitation of alkene *Z*-**2a** did not show triplet excited states (see Figure S19 in Supporting Information),^33^ the observed transient signal is clear spectroscopic evidence of photosensitized ^3^Z* → ^3^E* conversion.^34^ In addition, the delay observed in the generation of ^3^[BINAPCu^+^]*@alkene (Figure 2B) is attributed to diffusion issues which are considered as the limiting step in the energy transfer. On the contrary, when TAS experiments were performed in the presence of the complementary *E*-**2a** isomer, the formation of the same transient species was observed after sensitization (see the Supporting Information). However, while increasing concentrations of *Z*-**2a** up to 1 mM was enough to observe spectral and kinetic changes in the ^3^[Cu/BINAP]* TA signal, using *E*-**2a** as the quencher required an increased concentration of at least 15 mM (Figure 2C).

Finally, it is worth mentioning that quenching TAS experiments for ^3^Cu/BINAP* (Figure 2D,E) showed a mono-exponential lifetime that indicates that intermediate transient cannot be assigned to an electron transfer because these processes exhibit a bi-exponential long-lived behavior due to radical generation (similar results were obtained with *E*-**2a**, see the Supporting Information). Therefore, the sensitization of the *syn*-alkenyl boronic ester *Z*-**2a** occurs through an energy-transfer process from the ^3^[Cu/BINAP]* species. Additionally, the changes observed on the transient decay traces at 450 nm using *Z*-**2a**, where there is no overlap with the newly formed transient, unequivocally revealed a quenching of ^3^[Cu/BINAP]* species with *k*_q_ = 5.75 × 10^8^ M^–1^ s^–1^ (Figure 2F, red].^35^ Furthermore, this behavior was observed regardless of the excitation wavelength (see the Supporting Information), while using *E*-**2a** as the quencher led to a significantly lower *k*_q_ = 9.85 × 10^6^ M^–1^ s^–1^ (Figure 2F, blue]. These findings corroborated that upon excitation of the Cu/BINAP complex with visible light, the triplet excited state of the PS is efficiently populated and able to sensitize both *Z*- and *E*-alkenyl boronic esters. This interaction leads to a common transient intermediate after triplet–triplet energy transfer through a Dexter mechanism.^19,20^ However, based on the experimental *k*_q_ values from triplet quenching experiments, there is a kinetic preferential sensitization of the *Z*-isomer in the excited state, which in combination with the n_O_ → p_B_ interaction in the resulting *E*-isomer, accounts for the observed selectivity.

### Mechanistic Studies on Tandem Catalysis

**Figure 3:**
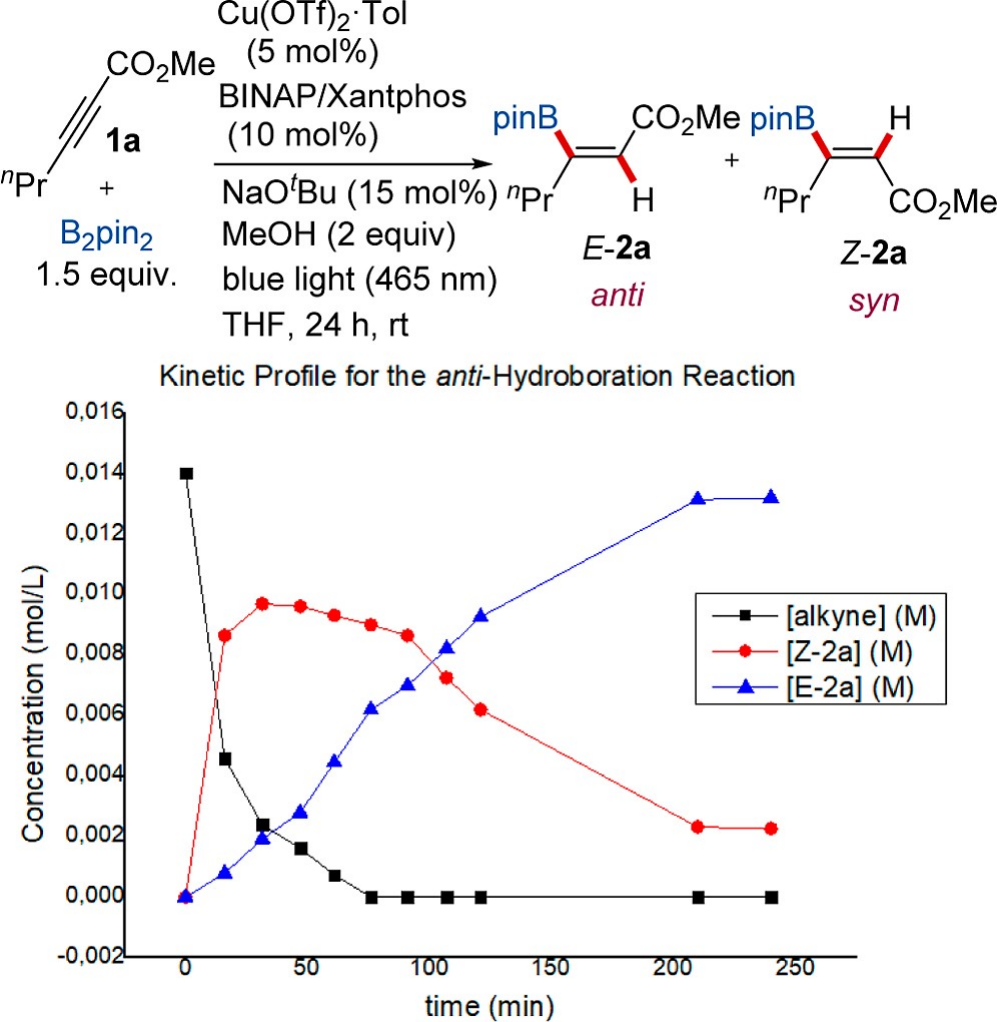
Kinetic profile for the anti-hydroboration of substrate **1a**. The kinetic experiment was performed at an initial concentration of alkyne **1a** of 0.014 M at 1 mmol scale.

To gain insights into whether a tandem process involving the cooperative action of two copper catalysts (namely, [Cu]/Xantphos and [Cu]/BINAP) is operative, a kinetic analysis of the anti-hydroboration of alkynoate **1a** was performed using a Kessil lamp. In this study, aliquots were taken from the reaction mixture at 10 timepoints over 250 min, and each one was analyzed by ^1^H NMR spectroscopy using dicyclohexyl phthalate as an internal standard to determine the ratio of **1a**, *Z*-**2a**, and *E*-**2a** (Figure 3).

The resulting kinetic profile shows that the initial borylcupration of the alkyne is very fast, with complete consumption of **1a** in the first 75 min of the reaction, whereas the photocatalytic alkene isomerization is significantly slower. A consequence of this is that there is accumulation of the *syn*-addition product *Z*-**2a** in the initial stage of the reaction, reaching its maximum concentration after 35 min. This isomer is converted steadily into *E*-**2a** until the *E*/*Z* ratio reaches 85:25, from where the progress of the reaction is almost negligible. This explains why a prolonged reaction time of 24 h is needed to observe full conversion toward the anti-isomer *E*-**2a**.

**Scheme 3:**
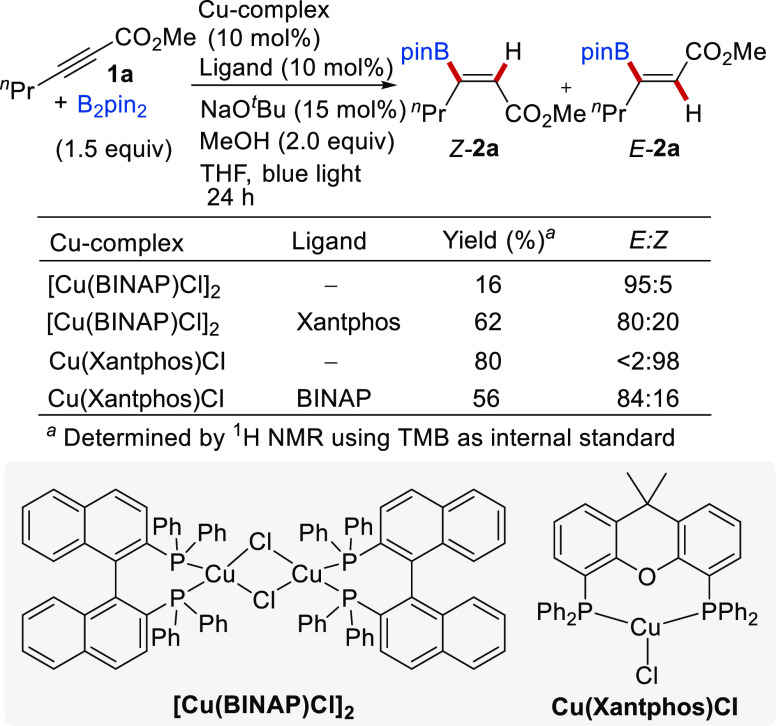
Control Experiments Suggesting Ligand Exchange

**Scheme 4:**
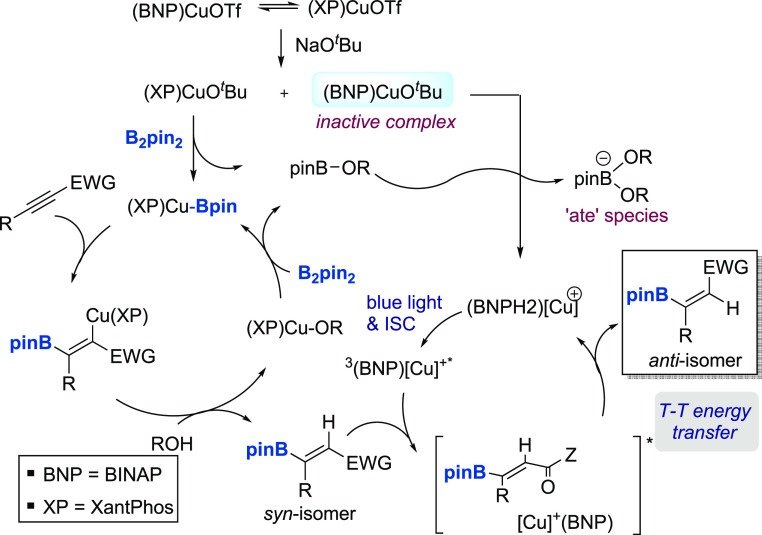
Mechanistic Proposal

This experiment suggests that the alkyne hydroboration and photoisomerization steps are independent processes catalyzed by two different Cu/biphosphine complexes formed in situ in the reaction medium. To substantiate this hypothesis, we studied the potential ligand exchange of the corresponding preformed copper complexes in the presence of an additional ligand. Because Cu/diphosphine complexes from [CuOTf]_2_·toluene were not sufficiently stable for isolation, these experiments were carried out with [Cu(BINAP)Cl]_2_ and Cu(Xantphos)Cl. Although previous optimization studies of the photoisomerization reaction revealed that the chloride counteranion was detrimental to the catalytic activity (see Table 1, entry 6), the thought behind using these complexes was that when exposed to NaO^*t*^Bu (required for the borylcupration step), a rapid ligand exchange of Cl^–^ with ^*t*^BuO^–^ should occur leading to the same complex of type Cu(diphosphine)O^*t*^Bu regardless of the counteranion (TfO^–^ or Cl^–^) of the initial Cu species. Indeed, when substrate **1a** was subjected to the tandem hydroboration/isomerization reaction in the presence of [Cu(BINAP)Cl]_2_ (5 mol %) as the only Cu/ligand source, the product **2a** was obtained with an exceptional 95% of *E*-stereoselectivity, albeit in a low yield (16%, Scheme 4). This result strongly suggests that (i) there is anion exchange during the reaction and (ii) the in situ generated Cu^I^/BINAP complex efficiently promotes the isomerization step, but it is poorly competent for the borylcupration step. However, the addition of 10 mol % of exogenous Xantphos ligand dramatically improved the conversion to **2a** to over 60% without precluding the isomerization process (*E/Z* = 80:20). Conversely, if Cu(Xantphos)Cl is used as the only precatalyst (10 mol %), the borylation product **2a** was formed in a good yield (80%) but the isomerization was totally inefficient (only *Z*-**2a** was detected in the reaction mixture, Scheme 3). The photoisomerization activity was restored to a significant extent without compromising the hydroboration reactivity when 10 mol % of BINAP was added to the reaction mixture, affording **2a** in 56% yield and 84% *E*-selectivity. Taken together, these data strongly argue in favor of an in situ ligand exchange process leading to the assembly of a [Cu]/Xantphos complex that catalyzes borylcupration of the alkyne and a [Cu]/BINAP complex that serves as a photocatalyst for the alkene isomerization. This notion of ligand exchange in solution was also supported by analysis of catalyst speciation by HRMS (identities supported by isotope patterns) and ^31^P NMR (based on chemical shift perturbations). These experiments suggest in situ formation of the expected [Cu(Xantphos)]^+^ and [Cu(BINAP)]^+^ complexes in solution (this analysis did not allow us to determine the nature of the counteranion). Importantly, the formation of the [Cu(Xantphos)]^+^ complex was observed to be favored over its BINAP analog. Additionally, [Cu(BINAP)_2_]^+^ species was also identified as minor species, which demonstrated no photocatalytic activity in the optimization studies. The latter could serve as a reservoir of the actual monoligated, photoactive Cu/BINAP complex (see the Supporting Information for details).

### Role of Lewis Acid Additives

Additional control experiments were designed to gain insights into the role of the alkoxide and possible intermediates during the course of the photoisomerization under the tandem conditions (Table 3). First, we investigated the photoisomerization of *Z*-**2a** catalyzed by [CuOTf]_2_·toluene/BINAP in the presence of a 15 mol % of NaO^*t*^Bu, which has an essential role in the hydroboration step by promoting the formation of the catalytically active LCuO^*t*^Bu (entry 2). The isomerization was completely inhibited, resulting in the exclusive recovery of the starting material. The same lack of reactivity was observed when NaOMe was used instead of NaO^*t*^Bu (entry 3) and when the photoactive Cu/BINAP couple (10 mol %) was used in combination with Xantphos (10 mol %) and NaO^*t*^Bu (15 mol %, entry 4). From these results, it appears that the resulting LCuO^*t*^Bu (L = BINAP or Xantphos) species are not competent for isomerization, which stands in complete agreement with the more strongly coordinating nature of the alkoxide compared to triflate.^22^ At this point, we hypothesized that the boron species present in the reaction (either B_2_pin_2_ or the borate of type pinB-OR formed upon σ-bond metathesis between the LCuOR and B_2_pin_2_) could behave as a Lewis acid and might “distract” the alkoxide from interacting with copper. Consistent with this proposal, the addition of a 50 mol % of the commercially available pinB-O^*i*^Pr as an additive to the photoisomerization of *Z-***2a** catalyzed by [CuOTf]_2_·Tol/BINAP (10 mol %) in the presence of NaO^*t*^Bu (15 mol %), triggered the isomerization process to a significant extent (*E/Z* = 45:55, entry 5). An increase of the amount of pinB-O^*i*^Pr to 1.5 equiv proved detrimental to the reaction outcome, leading to precipitation of a black solid, likely due to decomposition of the metal catalyst (entry 6). Interestingly, however, the use of a stronger Lewis acid such as BF_3_·Et_2_O (30 mol %), resulted in a clean isomerization to the anti-isomer in >98% conversion (entry 7). We rationalized that the Lewis acid species present in the reaction mixture could disrupt the tight ion pairing between a alkoxide and copper through acid–base interaction, thereby generating the photoactive cationic Cu/BINAP species having a weakly coordinating boron-ate as the counterion.^22,36,37^

**Table 3:**
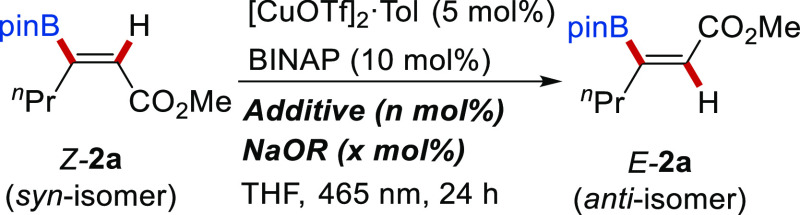
Role of the Alkoxide and the Boron Species in Isomerization

entry	NaOR (*x* mol %)	additive (*n* mol %)	*E*/*Z*^a^
1			97:3
2	NaO^*t*^Bu (15)		<2:98
3	NaOMe (15)		<2:98
4	NaO^*t*^Bu (15)	Xantphos (10)	<2:98
5	NaO^*t*^Bu (15)	pinB-O^*i*^Pr (50)	45:55
6	NaO^*t*^Bu (15)	pinB-O^*i*^Pr (150)	decomp
7	NaO^*t*^Bu (15)	BF_3_·Et_2_O	<98:2

a Determined in the crude reaction by ^1^H NMR spectroscopy (1,3,5-trimethoxy-benzene was used as an internal standard).

### Mechanistic Proposal

Although a full understanding of this tandem transformation will require further investigations, the mechanism shown in Scheme 4 accounts for the observations made to date. In the presence of Xantphos and BINAP ligands, a mixture of two cationic complexes Xantphos/CuOTf and BINAP-CuOTf complexes would coexist in dynamic equilibrium within the reaction system, as supported by HRMS and ^31^P NMR studies, the latter suggesting that the Xantphos complex is the predominant species at equilibrium (see the Supporting Information). Then, substitution of the readily displaceable triflate by alkoxide would occur upon addition of NaO^*t*^Bu, leading to the corresponding copper alkoxides. Although the BINAP complex is poorly reactive, the Xantphos/CuO^*t*^Bu complex readily reacts with B_2_pin_2_ following a σ-bond metathesis pathway to generate the nucleophilic Xantphos/Cu-Bpin species, along with the borate pinB(OR). Alkyne coordination to copper and 1,2-migration of the copper–boron bond across the alkyne results in an alkenyl–copper intermediate whose MeOH-assisted protonolysis affords the *syn*-hydroboration product. In a parallel cycle, the BINAP/CuO^*t*^Bu, with incompetent photoisomerization reactivity, can be activated by pinBOR, resulting in a catalytically competent cationic complex [BINAPCu^+^][pinB(OR)_2_^-^]. Blue light irradiation of this complex would generate the long-lived photoexcited ^3^[BINAPCu^+^]* complex that is capable of facilitating triplet sensitization of the *Z*-alkenyl boronic ester within the inner coordination sphere. This excitation allows for *Z* → *E* isomerization, whose directionality could be efficiently controlled by a stabilizing interaction between the carbonyl and the boron atom.^17,19^

## Conclusions

In summary, we have devised an innovative example of single metal/two ligands cooperative catalysis characterized by the in situ generation of two copper complexes engaged in two catalytic cycles that operate in tandem, each one providing a mechanistically distinct activation mode (organometal and photocatalytic). This strategy has made possible the development of a copper-catalyzed formal anti-hydroboration of internal alkynes activated with electron-withdrawing groups (i.e., esters and amides), providing access to an interesting and versatile class of trisubstituted alkenyl boronic esters. Mechanistic experiments have provided various pieces of evidence supporting the coexistence of a Cu/Xantphos complex, which is highly reactive toward the hydroboration of the alkyne, along with a cationic photoactive Cu/BINAP complex responsible for efficient photoisomerization of the resulting alkenyl boronic ester. Photophysical studies have shown that the triplet excited state of the in situ formed [Cu/BINAP] species is able to populate the triplet excited state of the *Z*-alkenyl boronic ester via an energy-transfer process. This step is crucial to modulate the geometry of the resulting olefin to access the desired anti-hydroboration product of *E*-configuration. Furthermore, the interplay between the two catalytic cycles is of critical importance for success because the pinB-OR species generated as a byproduct in the first step plays a crucial role in assisting alkoxide abstraction from the copper complex to generate the photoactive cationic Cu/BINAP species necessary in the second step. Further studies aimed at exploiting this mode of cooperative catalysis in other transformations are currently underway.

## Supporting Information Available

Experimental procedures, characterization of new compounds, photochemical and mechanistic studies, as well as copies of ^1^H and ^13^C NMR spectrums (PDF)

## Dedication

Dedicated to Prof. Dr. Carmen Carreño on the occasion of her retirement.
