# Supplementary material for: One-Metal/Two-Ligand for Dual Activation Tandem Catalysis: Photoinduced Cu-Catalyzed Anti-hydroboration of Alkynes
Source: J Am Chem Soc. 2022 Jul 5;144(28):13006–17. doi: 10.1021/jacs.2c05805 (PMC9348838; doi:10.1021/jacs.2c05805)
Supplement: Supplementary file 1 — ja2c05805_si_001.pdf [file ja2c05805_si_001.pdf]

One-Metal/Two-Ligand for Dual Activation Tandem Catalysis: Photoinduced Cu-Catalyzed *anti*-Hydroboration of Alkynes

Javier Corpas,[a] Miguel Gomez-Mendoza,[b] Jonathan Ramírez-Cárdenas,[a] Víctor A. de la Peña O'Shea,[b] Pablo Mauleón,*[a,c] Ramón Gómez Arrayás,*[a,c] and Juan C. Carretero[a,c]

[a] Departamento de Química Orgánica and Centro de Innovación en Química Avanzada (ORFEO-CINQA), Facultad de Ciencias, Universidad Autónoma de Madrid (UAM), 28049 Madrid (Spain).
[b] Photoactivated Processes Unit, IMDEA Energy Institute, Technological Park of Mostoles, Avda. Ramón de la Sagra 3, 28935, Madrid, Spain
[c] Institute for Advanced Research in Chemical Sciences (IAdChem), UAM, 28049 Madrid, Spain.

*E-mail: pablo.mauleon@uam.es
*E-mail: ramon.gomez@uam.es

Table of Contents

General Methods 3
Experimental Procedures 5
Synthesis of Starting Materials 5
Optimization studies. 10
General procedure for the *anti*-hydroboration of alkynes: 15
Unsuccesful substrates 19
Additive effects 20
Mechanistic experiments. 22
HRMS studies. 22
^{31}P NMR studies. 24
Kinetic studies. 25
Effects of Lewis acids in the reaction 26
Comparison with Thioxanthone sensitizer: attempts to develop Cu/Thioxanthone Tandem Catalysis ... 29
Determination of the stereochemistry 30
Stereochemical assignment by X-ray diffraction 35
Photochemical studies 38
DFT Studies 43
References 52
NMR spectra 54

General Methods

Anhydrous tetrahydrofuran (free of BHT inhibitor), dichloromethane, toluene, and acetonitrile were taken from a PureSolv MD purification system. Sodium tert-butoxide (2 M in THF), XantPhos, rac-BINAP, MeOH, alkyne **1a** and copper salts were purchased from commercial sources. Bis(pinacolato)diboron (B_2pin_2) was generously donated by Frontier Scientific and was used after washing with a diluted solution of HCl.[1] All other compounds were purchased from commercial sources and were used without further purification. All reactions were carried out in anhydrous solvents and under inert atmosphere, unless otherwise noted. Column liquid chromatography was performed on silica gel (230-400 mesh ASTM). TLC analysis was performed on 0.2 mm aluminum-based plates (230-400 mesh). ^{1}H, ^{13}C, ^{11}B, and ^{19}F NMR spectra were recorded in $CDCl_3$ solutions at 25 °C on AV-300, AVII-300 y AVIII-HD-300 (300, 75, 96, and 282 MHz, respectively) spectrometers (δ, ppm; J, Hz). ^{1}H and ^{13}C NMR spectra were referenced using the solvent signal as internal standard. In all boron containing compounds, the carbon attached to boron was not observed due to quadrupole broadening caused by the ^{11}B nucleus.[2] HRMS by electron ionization (EI), electrospray ionization (ESI) and atmospheric-pressure chemical ionization (APcI) were recorded using VG-Autospec (Waters) and HPLC1100 (Agilent)-coupled MAXIS II (Bruker) mass spectrometers. Chiral HPLC analysis were performed on an Agilent Technologies 1200 Series HPLC-DAD instrument equipped with a Daicel CHIRALPAK® IA, IC, ID, IF columns 4.6 mm x 250 mL, in order to determine enantiomeric excesses

Photocatalytic reactions. A custom-made photoreactor setup was used for the photocatalytic reactions developed by *Servicios Generales de Apoyo a la Investigación Experimental* (SEGAINVEX) at *Universidad Autónoma de Madrid* (UAM).[3] Photochemical reactions were run employing borosilicate glass vials. For reactions at room temperature, the vial was placed inside the fitted well in which irradiation takes place at 465 nm using 350 mW single LEDs located 1 cm beneath the base of the vial. Reaction temperature was kept at 20-25 ºC using a recirculating chiller. For the kinetic analysis the reaction flask was irradiated with a 34 W blue LEDs Kessil lamp (PR160L-440nm) located at 10 cm from the reaction vessel.

Ultraviolet–visible spectra (UV–Vis). Sample solutions in acetonitrile were obtained by a Perkin Elmer Lambda 1050 UV/Vis/NIR spectrometer.

Steady-state and time-resolved fluorescence (SS_F and TR_F). Experiments for acetonitrile solutions were carried out with a fluorescence spectrometer Perkin Elmer LS 55, with an excitation wavelength of 400 nm. On the other hand, time correlated single photon counting (TC-SPC) *Mini Tau* from Edinburgh Instruments was used as a Time-resolved fluorescence equipment. *Mini Tau* is provided with a band pass filter, using an EPL-375 ps pulsed diode laser with emission at 445nm as excitation source (both from Edinburgh Instruments). The instrument response function (IRF) signal was included as reference.
The singlet excited state energy (E_s) was calculated using the following equation:

$$E_S = N_A \frac{hc}{\lambda} \quad [J \cdot mol^{-1}]$$

where N_A is the Avogrado's number, *h* the Planck's constant, *c* is the velocity of light and λ is the wavelength of the intersection of the normalized emission spectra and excitation expressed in meters. Then, the conversion between Jules to calories can be applied to obtain the value in terms of Kcal · mol^{-1}.

Fluorescence quantum yield (ϕ_F) for Cu-BINAP was determined using the following standards: fluorescein[4] or riboflavin[5] (R. Martinez-Haya, M. A. Miranda and M. L. Marin, Eur. J. Org. Chem. 2017, 2164–2169) using the following equation:

$$\phi_{Fi} = \phi_s \frac{I_i}{I_s} \frac{n_i^2}{n_s^2} \frac{1 - 10^{Abs_s}}{1 - 10^{Abs_i}}$$

where the subscripts "i" and "s" refer to the sample of interest and the standard, respectively, *n* represents the refractive index of the corresponding solvents, *I* represent the integrals of the corresponding emission spectra and *Abs* represents the absorbance at the λexcitation.

Transient Absorption Spectroscopy (TAS). The laser flash photolysis equipment LP980 from Edinburgh Instruments is provided by an optical parametric oscillator (OPO) pumped by the third harmonic of a Nd:YAG laser (EKSPLA). The single pulses were ca. 5 ns duration, and the energy was adjusted to 1 mJ by pulse at excitation wavelength (355 or 445 nm). A pulsed xenon flash lamp (150 W) was employed as detecting light source. A monochromator (TMS302-A, grating 150 lines/mm) disperses the probe light after it has passed the sample. The probe light is then passed on to a PMT detector (Hamamatsu Photonics) to obtain the transient signals. The change in optical density in transitory measurements is defined as ΔOD. ΔOD correspond to the change in triplet absorbance as a function of time is recorded by monitoring the probe beam (xenon flash lamp) at the triplet-triplet transition wavelength. An experimental measurement involves the simultaneous combination of pump and probe beams. The "probe-only" spectrum corresponds to the ground state absorption, whereas the "pump + probe" spectrum contains contributions from both the ground and excited states. Transient data is presented as the change in absorption ΔOD, which is effectively the difference between the "pump + probe" and the "probe-only" spectra.

The absorbance of the solutions containing the Cu/BINAP sensitizer in acetonitrile was kept at ~0.3 (20 μM of dye) at λexcitation = 355 nm or 60 μM of dye at λexcitation = 455 nm and the samples were recorded at room temperature using 1 × 1 cm^2 quartz cells with 3 mL capacity and were bubbled for 15 min with N_2 or O_2 before acquisition. All transient lifetimes were fitted both as mono or bi-exponential functions for the decay traces registered at 450 and 650 nm. In the case of photophysical measurements for alkenes, concentration was taken into account instead of absorbance due to the low absorbance of them.

Quenching Experiments. In a typical quenching experiment, the appropriate volumes of a freshly prepared of *syn*- or *anti*-isomer acetonitrile solution were added to the aerated or purged Cu-BINAP solution. The absorbance of the samples was kept at ~0.1 or 0.3 for fluorescence or TAS experiments at λexcitation = 400-445 or 355-445 nm for fluorescence or TAS measurements, respectively. All the photophysical measurements were performed at room temperature in a quartz cell of 1.0 cm optical path length.

Experimental Procedures

Synthesis of Starting Materials

Methyl 6-hydroxyhex-2-ynoate (I4):

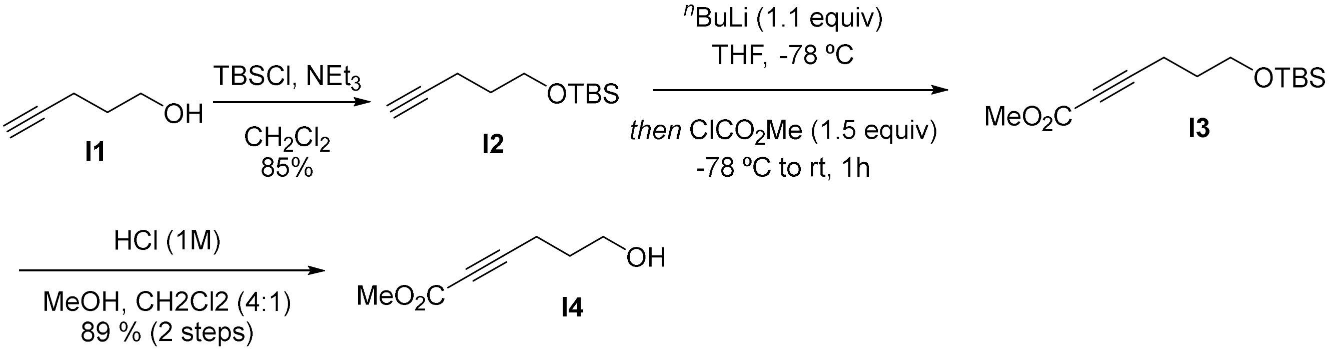

Precursor **I4** was synthesized according to a modified reported method:[6] ether **I2** was prepared from 4-pentyn-1-ol (**I1,** 0.553 mL, 5.94 mmol, 1.0 equiv) upon treatment with NEt_3 (1.24 mL, 1.5 equiv) and TBSCl (1.07 g, 1.2 equiv) in CH_2Cl_2 (20 mL) under a nitrogen atmosphere at 0 °C. The solution was allowed to stir overnight at room temperature, before being quenched with a saturated solution of NH_4Cl (1x20 mL) and the resulting organic phase was washed with water (1x 20 mL) and brine (1x 20 mL). The resulting organic phase was dried with Na_2SO_4, filtered and the resulting the solvent was removed from vacuo yielding a yellow oil which was further purified by column chromatography (SiO_2, heptane:AcOEt (100:5)), to give silyl ether **I2** (1.0, 85%) as a colorless oil.

Synthesis of methyl ester **I3**: ^{n}BuLi (2.22 mL, 2.5 M in hexanes, 5.56 mmol, 1.1 equiv) was added dropwise to a stirred solution of alkyne **I2** (1.0, 5.05 mmol, 1.0 equiv) in THF (25 mL) under a nitrogen atmosphere at −78 °C. The solution was allowed to stir at −78 °C for 20 min before $ClCO_2Me$ (0.58 mL, 7.58 mmol, 1.5 equiv) was added dropwise. The resulting solution was allowed to warm to 0 °C and stirred for 1.5 h, before being quenched with sat. aq. NH_4Cl (25 mL). The layers were separated, and the aqueous layer was extracted with AcOEt (3 × 25 mL). The combined organic phases were washed with brine (25 mL) and dried over Na_2SO_4. Filtration and removal of the solvent under reduced pressure gave methyl ester **I3** as a yellow oil which was taken directly for the next step without further purification.

Synthesis of **I4**: alkyne **I3** (1.29 g, 5.05 mmol, 1.0 equiv) was treated with a solution of 1.0 M HCl (7.58 mL, 7.58 mmol, 1.5 equiv) was in MeOH/CH_2Cl_2 (4:1, 17 mL) at 0 °C. The solution was allowed to warm to room temperature and stirred for 24 h. CH_2Cl_2 (25 mL), brine (25 mL) and H_2O (10 mL) were added, and the layers separated. The aqueous layer was extracted with CH_2Cl_2 (3 × 25 mL), and the combined organic phases were washed with brine (25 mL) and dried over Na_2SO_4. Filtration and removal of the solvent under reduced pressure resulted in a yellow oil, which was purified by flash chromatography (SiO_2, 5% to 50% EtOAc/n-heptane), affording alcohol **I4** (639 mg, 89% over two steps) as an oil. Spectroscopic data were identical to those reported in the literature.

6-Hydroxy-*N,N*-dimethylhex-2-ynamide (I6):

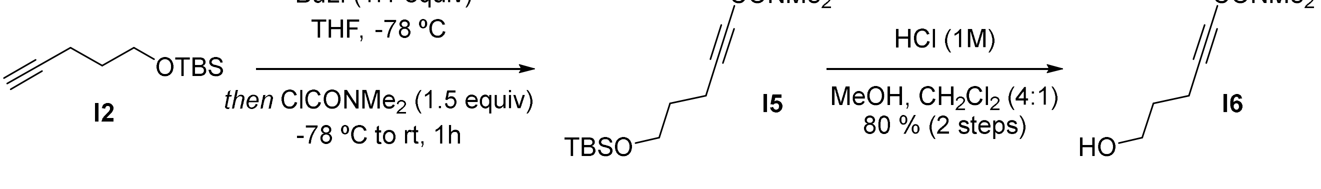

Synthesis of **I5**: ^{n}BuLi (2.22 mL, 2.5 M in hexanes, 5.56 mmol, 1.1 equiv) was added dropwise to a stirred solution of alkyne **I2** (1.0, 5.05 mmol, 1.0 equiv) in THF (25 mL) under a nitrogen atmosphere at −78 °C. The solution was allowed to stir at −78 °C for 20 min before dimethylcarbamyl chloride ($ClCONMe_2$, 0.70 mL, 7.58 mmol, 1.5 equiv) was added dropwise. The resulting solution was allowed to warm to 0 °C and stirred for 1.5 h, before being quenched with sat. aq. NH_4Cl (25 mL). The layers were separated, and the aqueous layer was extracted with AcOEt (3 × 25 mL). The combined organic phases were washed with brine (25 mL) and dried over Na_2SO_4. Filtration and removal of the solvent under reduced pressure gave dimethyl amide **I5** as a yellow oil which was taken directly for the next step without further purification.

Deprotection step: the residue obtained din the previous step was treated with a solution of 1.0 M HCl (7.58 mL, 7.58 mmol, 1.5 equiv) was in MeOH/CH_2Cl_2 (4:1, 17 mL) at 0 °C. The solution was allowed to warm to room temperature and stirred for 24 h. CH_2Cl_2 (25 mL), brine (25 mL), and H_2O (10 mL) were added, and the layers separated. The aqueous layer was extracted with CH_2Cl_2 (3 × 25 mL), and

the combined organic phases were washed with brine (25 mL) and dried over Na_2SO_4. Filtration and removal of the solvent under reduced pressure resulted in a yellow oil, which was purified by flash chromatography (SiO_2, 5% to 70% EtOAc/^{n}-heptane), affording amide **I6** (627 mg, 80% over two steps) as an oil. **^{1}H-NMR** (300 MHz, $CDCl_3$): δ 154.9, 92.9, 74.3, 61.1, 38.5, 34.2, 30.6, 15.6. **^{13}C-NMR** (75 MHz, $CDCl_3$): δ 3.73 (t, J = 6.1 Hz, 2H), 3.17 (s, 3H), 2.94 (s, 3H), 2.48 (t, J = 7.1 Hz, 2H), 1.81 (p, J = 6.6, 6.1 Hz, 2H). **HRMS** (ESI+): *m/z* $[M + H]^+$ calcd for $C_8H_{14}NO_2$ 156.1019, found 156.1023.

Synthesis of Alkynes (procedure 1):

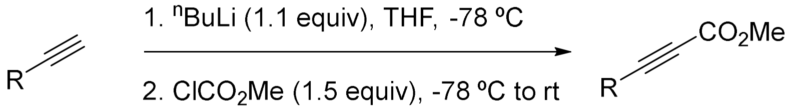

Alkynes **1b** and **1c** were prepared according to procedure 1: the terminal alkyne (1 mmol, 1.0 equiv) in THF (50 mL) was treated with ^{n}BuLi (0.44 mL, 2.5 M, 1.1 mmol, 1.1 equiv) by dropwise addition at -78 °C. After that, the reaction was allowed to stir for 30 min at -78 °C and then methyl chloroformate (0.12 mL, 1.5 mmol, 1.5 equiv) was added dropwise at -78 °C. The reaction mixture was stirred at -78 °C for 1 h before reaching room temperature. Then, the reaction mixture was quenched with a saturated solution of NH_4Cl (100 mL) at 0 °C. The layers were separated, and the aqueous layer was extracted with AcOEt (3 × 50 mL). The combined organic phases were washed with brine (100 mL) and dried over Na_2SO_4. Filtration and removal of the solvent under reduced pressure gave an oil which was further purified by silica gel chromatography.

Methyl 3-cyclohexylpropiolate (1b):

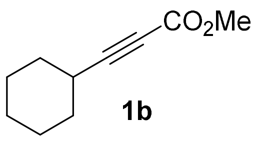

1b

Following the procedure 1, the treatment of cyclohexylacetylene (0.13 mL, 1 mmol, 1.0 equiv) with ^{n}BuLi (0.44 mL, 2.5 M, 1.1 mmol, 1.1 equiv), and methyl chloroformate (0.12 mL, 1.5 mmol, 1.5 equiv) afforded the desired alkyne 1b after purification by column chromatography (SiO_2, n-heptane:AcOEt, 20:1) as a colorless oil (156 mg, 94% yield). **^{1}H-NMR** (300 MHz, $CDCl_3$): δ 3.68 (s, 3H), 2.45 (tt, J = 8.8, 3.7 Hz, 1H), 1.83 – 1.71 (m, 2H), 1.70-1.58 (m, 2H), 1.54-1.37 (m, 3H), 1.34-1.20 (m, 3H). **^{13}C-NMR** (75 MHz, $CDCl_3$): δ154.3, 93.1, 72.8, 52.4, 31.4, 28.8, 25.6, 24.6. **HRMS** (ESI+): *m/z* $[M + H]^+$ calcd for $C_{10}H_{15}O_2$ 167.1067, found 167.1072.

methyl 7-chlorohept-2-ynoate (1c):

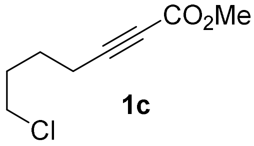

1c

Following the procedure 1, the treatment of cyclohexylacetylene (0.12 mL, 1 mmol, 1.0 equiv) with ^{n}BuLi (0.44 mL, 2.5 M, 1.1 mmol, 1.1 equiv), and methyl chloroformate (0.12 mL, 1.5 mmol, 1.5 equiv) afforded the desired alkyne 1c after purification by column chromatography (SiO_2, n-heptane:AcOEt, 20:1) as a colorless oil (126 mg, 72% yield).
^{1}H-NMR (300 MHz, $CDCl_3$): δ 3.67 (s, 3H), 3.50 (t, J = 6.1 Hz, 2H), 2.33 (t, J = 6.8 Hz, 2H), 1.91 – 1.76 (m, 2H), 1.75-1.62 (m, 2H). **^{13}C-NMR** (75 MHz, $CDCl_3$): δ154.0, 88.5, 73.3, 52.5, 44.1, 31.3, 24.7, 17.9. **HRMS** (ESI+): *m/z* $[M + H]^+$ calcd for $C_8H_{12}ClO_2$, 175.6327 found 175.6323.

Synthesis of Alkynes (procedure 2):

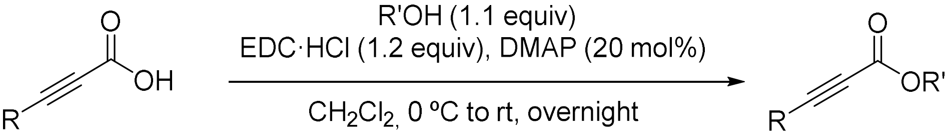

General procedure for the esterification reaction (procedure 2):[7] to a solution of the carboxylic acid (1 mmol, 1.0 equiv) in CH_2Cl_2 (10 mL) was added EDC·HCl (230 mg, 1.2 mmol, 1.2 equiv) at 0 ºC followed by a catalytic amount of DMAP (24 mg, 0.2 mmol, 20 mol%). Then, the corresponding alcohol (1.1 equiv) was added to the mixture and the reaction was allowed to stir for 30 min at 0ºC. After that, the resulting solution was stirred overnight. Then, the reaction mixture was washed with H_2O (3 x 15 mL). The layers were separated, and the aqueous layer was extracted with CH_2Cl_2 (2 × 20 mL). The combined organic phases were washed with brine (15 mL) and dried over Na_2SO_4. Filtration and removal of the solvent under reduced pressure gave a residue which was further purified by silica gel chromatography.

Synthesis of Alkynes (procedure 3):

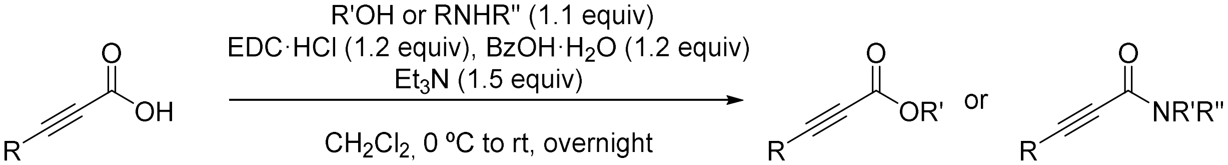

General procedure for the esterification reaction (procedure 3):[8] to a solution of the carboxylic acid (1 mmol, 1.0 equiv) in CH_2Cl_2 (10 mL) was added EDC·HCl (230 mg, 1.2 mmol, 1.2 equiv) at 0 ºC followed by Et_3N (0.21 mL, 1.5 mmol, 1.5 equiv) and 1-hydroxybenzotriazole hydrate (BzOH·H_2O, (162.14 mg, 1.2 mmol, 1.2 equiv). Then, the corresponding alcohol or amine (1.1 equiv)

was added to the mixture and the reaction was allowed to stir for 30 min at 0ºC. After that, the resulting solution was stirred overnight. Then, the reaction mixture was washed with H_2O (3 x 15 mL). The layers were separated, and the aqueous layer was extracted with CH_2Cl_2 (2 × 20 mL). The combined organic phases were washed with brine (15 mL) and dried over Na_2SO_4. Filtration and removal of the solvent under reduced pressure gave a residue which was further purified by silica gel chromatography.

Synthesis of Alkynes (procedure 4):

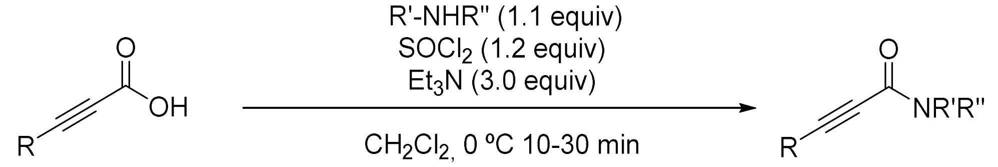

Following the previously reported method in the literature:[9] the corresponding carboxylic acid (1 mmol, 1.0 equiv) and the amine (1.1 mmol, 1.1 equiv) were dissolved in CH_2Cl_2 (10 mL). After that, Et_3N was added (0.42 mL, 3.0 mmol, 3.0 equiv) followed by the addition of $SOCl_2$ (0.087 mL, 1.2 mmol, 1.2 equiv), and the mixture was followed by TLC analysis until completion (typically 20 min). After that, the solvent was removed in vacuo yielding a residue that is dissolved in CH_2Cl_2 (10 mL). This solution was washed with 1M HCl (10 mL), 1M NaOH (10 mL) and water (2 x 10 mL). The organic phase was dried with Na_2SO_4, filtered, and the solvent was removed under vacuo. The resulting residue was further purified by column chromatography.

Benzyl 3-(hex-2-ynoyloxy)azetidine-1-carboxylate (1d):

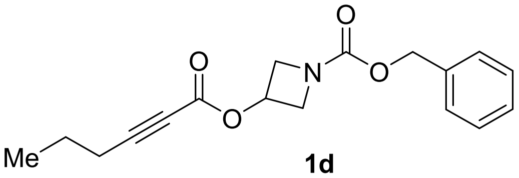

1d

Following the general procedure 2, the treatment of 2-hexynoic acid (0.11 mL, 1 mmol, 1.0 equiv) with EDC·HCl (230 mg, 1.2 mmol, 1.2 equiv) and DMAP (24 mg, 0.2 mmol, 20 mol%) in presence of 1-Cbz-3-Hydroxyazetidine (CAS: 128117-22-6, 228 mg, 1.1 mmol, 1.1 equiv) yielded the titled compound after purification by column chromatography (SiO_2, n-heptane:AcOEt, 6:1). as an oil (262 mg, 87%). **^{1}H-NMR** (300 MHz, $CDCl_3$): δ 7.38 – 7.25 (m, 5H), 5.27 – 5.14 (m, 1H), 5.10 (s, 2H), 4.33 (dd, J = 10.2, 6.8 Hz, 2H), 4.08 – 3.95 (m, 2H), 2.33 (t, J = 7.0 Hz, 2H), 1.61 (p, J = 7.2 Hz, 2H), 1.02 (t, J = 7.4 Hz, 3H). **^{13}C-NMR** (75 MHz, $CDCl_3$): δ 156.3, 152.9, 136.5, 128.6, 128.3, 128.2, 91.4, 72.7, 67.0, 64.8, 56.3, 21.1, 20.8, 13.6. **HRMS** (ESI+): *m/z* $[M + H]^+$ calcd for $C_{17}H_{20}NO_4$ 302.1387, found 302.1394.

(*S*)-(4-(prop-1-en-2-yl)cyclohex-1-en-1-yl)methyl hex-2-ynoate (1e):

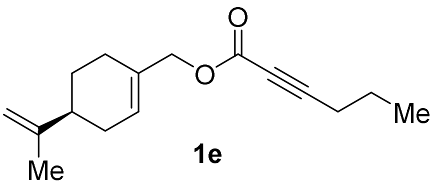

1e

Following the general procedure 2, the treatment of 2-hexynoic acid (0.11 mL, 1 mmol, 1.0 equiv) with EDC·HCl (230 mg, 1.2 mmol, 1.2 equiv) and DMAP (24 mg, 0.2 mmol, 20 mol%) in presence of (*S*)-(-)-perillyl alcohol (CAS: 18457-55-1, 0.18 mL, 1.1 mmol, 1.1 equiv) yielded the titled compound after purification by column chromatography (SiO_2, n-heptane:AcOEt, 15:1). as an oil (224 mg, 91%). **^{1}H-NMR** (300 MHz, $CDCl_3$): δ 5.83 – 5.75 (m, 1H), 4.74-4.69 (m, 2H), 4.54 (s, 2H), 2.30 (t, J = 7.1 Hz, 2H), 2.20 – 2.06 (m, 4H), 2.04 – 1.91 (m, 1H), 1.90 – 1.79 (m, 1H), 1.73 (s, 3H), 1.62 (dt, J = 14.5, 7.3 Hz, 2H), 1.56 – 1.44 (m, 1H), 1.01 (t, J = 7.4 Hz, 3H). **^{13}C-NMR** (75 MHz, $CDCl_3$): δ 154.0, 149.6, 132.0, 127.0, 108.9, 89.6, 73.3, 69.8, 40.8, 30.6, 27.4, 26.5, 21.2, 20.8, 20.7, 13.6. **HRMS** (ESI+): *m/z* $[M + NH_4]^+$ calcd for $C_{16}H_{26}NO_2$ 264.1958, found 264.1958.

3-(Naphthalen-1-yl)prop-2-yn-1-yl hex-2-ynoate (1f):

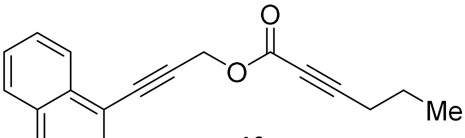

1f

Following the general procedure 2, the treatment of 2-hexynoic acid (0.11 mL, 1 mmol, 1.0 equiv) with EDC·HCl (230 mg, 1.2 mmol, 1.2 equiv) and DMAP (24 mg, 0.2 mmol, 20 mol%) in presence of 3-(1-naphthyl)-2-propyn-1-ol (CAS: 16176-22-0, 200 mg, 1.1 mmol, 1.1 equiv) yielded the titled compound after purification by column chromatography (SiO_2, n-heptane:AcOEt, 15:1). as an oil (260 mg, 94%). **^{1}H-NMR** (300 MHz, $CDCl_3$): δ 8.32 (d, J = 8.3 Hz, 1H), 7.85 (d, J = 8.2 Hz, 2H), 7.70 (d, J = 8.0 Hz, 1H), 7.62-7.48 (m, 2H), 7.42 (dd, J = 8.2, 7.2 Hz, 1H), 5.14 (s, 2H), 2.34 (t, J = 7.1 Hz, 2H), 1.63 (h, J = 7.3 Hz, 2H), 1.03 (t, J = 7.4 Hz, 3H). **^{13}C-NMR** (75 MHz, $CDCl_3$): δ 153.2, 133.5, 133.2, 131.2, 129.5, 128.4, 127.1, 126.6, 126.2, 125.2, 119.7, 91.1, 87.0, 85.4, 72.8, 54.2, 21.1, 20.8, 13.6. **HRMS** (GC EI+): *m/z* $[M]^+$ calcd for $C_{19}H_{16}O_2$ 276.1150, found 276.1140.

(*8R,9S,13S,14S*)-13-methyl-17-oxo-7,8,9,11,12,13,14,15,16,17-decahydro-6*H*-cyclopenta[*a*]phenanthren-3-yl hex-2-ynoate (1g):

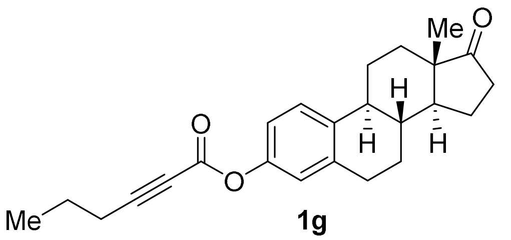

1g

Following the general procedure 2, the treatment of 2-hexynoic acid (0.11 mL, 1 mmol, 1.0 equiv) with EDC·HCl (230 mg, 1.2 mmol, 1.2 equiv) and DMAP (24 mg, 0.2 mmol, 20 mol%) in presence of estrone (CAS: 53-16-7, 297.4 mg, 1.1 mmol, 1.1 equiv) yielded the titled compound after purification by column chromatography (SiO_2, n-heptane:AcOEt, 10:1 to 5:1). as white solid (292 mg, 80%). **^{1}H-NMR** (300 MHz, $CDCl_3$): δ 7.28 (d, J = 8.5 Hz, 1H), 6.89 (dd, J = 8.4, 2.5 Hz, 1H), 6.85 (d, J = 2.5 Hz, 1H), 2.90 (dd, J = 8.7, 4.1 Hz, 2H), 2.50 (dd, J = 18.3, 8.3 Hz, 1H), 2.37 (t, J = 7.0 Hz, 2H), 2.34 – 2.22 (m, 1H), 2.23-2.11 (m, 1H), 2.10-1.90 (m, 3H), 1.72 – 1.32 (m, 8H), 1.04 (t, J = 7.4 Hz, 3H), 0.90 (s, 3H).
^{13}C-NMR (75 MHz, $CDCl_3$): δ 220.8, 152.5, 148.1, 138.3, 138.0, 126.6, 121.5, 118.7, 92.0, 73.1, 50.5, 48.0, 44.3, 38.1, 35.9, 31.7, 29.5, 26.4, 25.8, 21.7, 21.1, 20.9, 13.9, 13.6. **HRMS** (ESI+): *m/z* $[M + H]^+$ calcd for $C_{24}H_{29}O_3$ 365.2111, found 365.2114.

Methyl 6-(2-(1,3-dimethyl-2,6-dioxo-1,2,3,6-tetrahydro-7H-purin-7-yl)acetoxy)hex-2-ynoate (1h):

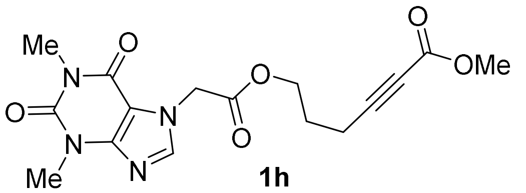

1h

Following the general procedure 2, the treatment of theophylline-7-acetic acid (CAS: 652-37-9, 238.2 mg, 1.0 mmol, 1.0 equiv) with EDC·HCl (230 mg, 1.2 mmol, 1.2 equiv) and DMAP (24 mg, 0.2 mmol, 20 mol%) in presence of precursor **I4** (156.2 mg, 1.1 mmol, 1.1 equiv) yielded the titled compound after purification by column chromatography (SiO_2, n-heptane:AcOEt, 2:1 to AcOEt:MeOH (1%)) as yellow oil (239 mg, 66%). **^{1}H-NMR** (300 MHz, $CDCl_3$): δ 7.64 (s, 1H), 5.12 (s, 2H), 4.34 (t, J = 6.1 Hz, 2H), 3.76 (s, 3H), 3.61 (s, 3H), 3.38 (s, 3H), 2.47 (t, J = 6.9 Hz, 2H), 2.04 – 1.93 (m, 2H). **^{13}C-NMR** (75 MHz, $CDCl_3$): δ 167.0, 155.1, 153.9, 151.5, 148.5, 142.0, 107.0, 87.7, 73.4, 64.5, 52.6, 47.2, 29.7, 27.8, 26.4, 15.4. **HRMS** (ESI+): *m/z* $[M + H]^+$ calcd for $C_{16}H_{19}N_4O_6$ 363.1299, found 363.1308.

***rac*-(*R*)-2,5,7,8-tetramethyl-2-((*4R,8R*)-4,8,12-trimethyltridecyl)chroman-6-yl hex-2-ynoate (1i):**

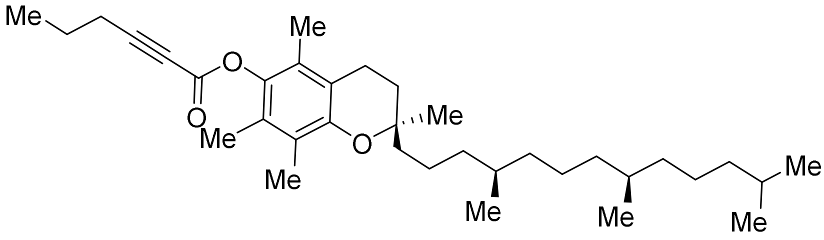

1i

Following the general procedure 2, the treatment of 2-hexynoic acid (0.11 mL, 1 mmol, 1.0 equiv) with EDC·HCl (230 mg, 1.2 mmol, 1.2 equiv) and DMAP (24 mg, 0.2 mmol, 20 mol%) in presence of α-tocopherol (CAS: 10191-41-0, 473.8 mg, 1.1 mmol, 1.1 equiv) yielded the titled compound after purification by column chromatography (SiO_2, n-heptane:AcOEt, 40:1) as yellow oil (509 mg, 97%). **^{1}H-NMR** (300 MHz, CDCl3): δ 2.60 (t, J = 6.8 Hz, 2H), 2.39 (td, J = 7.1, 1.7 Hz, 2H), 2.10 (s, 3H), 2.07 (s, 3H), 2.03 (s, 3H), 1.78 (ddd, J = 21.4, 14.3, 7.0 Hz, 2H), 1.73 – 1.62 (m, 2H), 1.59 – 1.05 (m, 26H), 0.92-0.84 (m, 13H). **^{13}C-NMR** (75 MHz, CDCl3): δ 152.8, 149.9, 140.2, 126.8, 125.1, 123.3, 117.6, 91.4, 75.3, 73.1, 39.5, 37.6 (2C), 37.5, 37.4, 32.9, 32.8 (2C), 28.1, 25.0, 24.6, 22.9, 22.8, 21.2, 21.1, 20.9, 20.7, 19.9, 19.8. **HRMS** (ESI+): *m/z* $[M + H]^+$ calcd for $C_{35}H_{57}O_3$ 525.4302, found 525.4298.

6-Methoxy-6-oxohex-4-yn-1-yl(*2S,4aR,7S,9aS,10S*)-1-methyl-8-methylene-13-oxo-2,7-bis((triethylsilyl)oxy)-1,2,4b,5,6,7,8,9,10,10a-decahydro-4a,1-(epoxymethano)-7,9a-methanobenzo[*a*]azulene-10-carboxylate (1j):

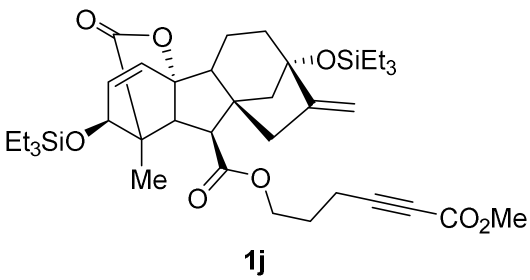

1j

Following the general procedure 2, the treatment of silylated gibberellic acid[6] (574.9 mg, 1.0 mmol, 1.0 equiv) with EDC·HCl (230 mg, 1.2 mmol, 1.2 equiv) and DMAP (24 mg, 0.2 mmol, 20 mol%) in presence of precursor **I4** (156.2 mg, 1.1 mmol, 1.1 equiv) yielded the titled compound after purification by column chromatography (SiO_2, n-heptane:AcOEt, 20:1, 10:1 to 5:1) as an oil (549.6 mg, 71%). **^{1}H-NMR** (300 MHz, $CDCl_3$): δ 6.12 (d, J = 9.3 Hz, 1H), 5.66 (dd, J = 9.3, 3.6 Hz, 1H), 5.13 (s, 1H), 4.79 (s, 1H), 4.13 (td, J = 6.3, 2.7 Hz, 2H), 4.04 (d, J = 3.6 Hz, 1H), 3.66 (s, 3H), 3.22 (d, J = 10.9 Hz, 1H), 2.66 (d, J = 10.9 Hz, 1H), 2.35 (t, J = 7.2 Hz, 2H), 2.07 – 1.74 (m, 9H), 1.09 (s, 3H), 0.91 – 0.81 (m, 18H), 0.57 – 0.45 (m, 12H). **^{13}C-NMR** (75 MHz, $CDCl_3$): δ 179.1, 171.8, 157.0, 154.0, 131.6, 107.5, 90.9, 87.3, 79.2, 73.7, 70.1, 63.2, 54.3, 52.8, 52.7, 51.2, 51.1, 50.8, 44.4, 42.9, 40.7, 26.8, 17.1, 15.7, 15.1, 7.1, 6.9, 6.7, 5.1. **HRMS** (ESI+): *m/z* $[M + H]^+$ calcd for $C_{38}H_{59}O_8Si_2$ 699.3743, found 699.3765.

Methyl 6-(2-(1-(4-chlorobenzoyl)-5-methoxy-2-methyl-1*H*-indol-3-yl)acetoxy)hex-2-ynoate (1k):

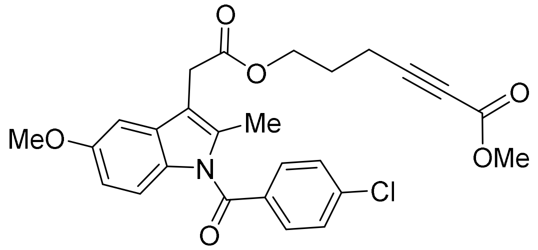

1k

Following the general procedure 2, the treatment of Indomethacin (CAS: 53-86-1, 357.7 mg, 1.0 mmol, 1.0 equiv) with EDC·HCl (230 mg, 1.2 mmol, 1.2 equiv) and DMAP (24 mg, 0.2 mmol, 20 mol%) in presence of precursor **I4** (156.2 mg, 1.1 mmol, 1.1 equiv) yielded the titled compound after purification by column chromatography (SiO_2, n-heptane:AcOEt, 10:1 to 3:1) as yellow solid (434 mg, 90%). **^{1}H-NMR** (300 MHz, $CDCl_3$): δ 7.65 (d, J = 8.4 Hz, 2H), 7.46 (d, J = 8.4 Hz, 2H), 6.94 (t, J = 2.3 Hz, 1H), 6.85 (dd, J = 9.0, 4.7 Hz, 1H), 6.68-6.62 (m, 1H), 4.19 (t, J = 6.1 Hz, 2H), 3.83 (s, 3H), 3.74 (s, 3H), 3.67 (s, 2H), 2.40 – 2.30 (m, 5H), 1.95 – 1.83 (m, 2H). **^{13}C-NMR** (75 MHz, $CDCl_3$): δ 170.8, 168.4, 156.2, 154.0, 139.4, 136.0, 134.0, 131.3, 130.9, 130.7, 129.2, 115.1, 112.5, 111.8, 101.3, 87.9, 73.5, 63.3, 55.8, 52.7, 30.4, 26.8, 15.6, 13.4. **HRMS** (ESI+): *m/z* $[M + H]^+$ calcd for $C_{26}H_{25}ClNO_6$ 482.1365, found 482.1380.

(*S,Z*)-3-(2-((*1R,3aS,7aR,E*)-1-((*2R,5R,E*)-5,6-dimethylhept-3-en-2-yl)-7a-methyloctahydro-4*H*-inden-4-ylidene)ethylidene)-4-methylenecyclohexyl hex-2-ynoate (1l):

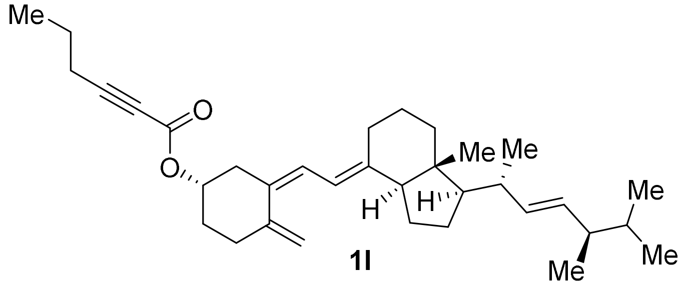

1l

Following the general procedure 3, the treatment of 2-hexynoic acid (0.11 mL, 1 mmol, 1.0 equiv) with EDC·HCl (230 mg, 1.2 mmol, 1.2 equiv), Et_3N (0.21 mL, 1.5 mmol, 1.5 equiv) and 1-hydroxybenzotriazole hydrate (162.14 mg, 1.2 mmol, 1.2 equiv) in presence of ergocalciferol (CAS: 50-14-6, 436.3 mg, 1.1 mmol, 1.1 equiv) yielded the titled compound after purification by column chromatography (SiO_2, n-heptane:AcOEt, 15:1) as yellow solid (251 mg, 51%). ^{1}H-NMR (300 MHz, $CDCl_3$): δ 6.22 (d, J = 11.2 Hz, 1H), 6.01 (d, J = 11.2 Hz, 1H), 5.20 – 5.16 (m, 2H), 5.05 (bs, 1H), 5.03-4.95 (m, 1H), 4.83 (d, J = 2.0 Hz, 1H), 2.81 (dd, J = 11.3, 3.3 Hz, 1H), 2.61 (dd, dd, J = 13.3, 4.0 Hz, 1H), 2.44 – 2.37 (m, 2H), 2.29 (t, J = 7.1 Hz, 2H), 2.22 – 2.14 (m, 2H), 2.03 – 1.94 (m, 5H), 1.84 (q, J = 6.1 Hz, 2H), 1.67 – 1.58 (m, 6H), 1.52-1.40 (m, 2H), 1.36-1.20 (m, 2.0 Hz, 2H), 1.00 (t, J = 6.3 Hz, 3H), 0.90 (d, J = 6.8 Hz, 6H), 0.83 (d, J = 6.7 Hz, 3H), 0.81 (d, J = 6.7 Hz, 3H), 0.54 (s, 3H). **^{13}C-NMR** (75 MHz, $CDCl_3$): δ 153.5, 144.4, 142.8, 135.7, 134.0, 132.1, 122.9, 117.6, 113.0, 89.4, 73.7, 73.6, 56.6, 45.7, 42.9, 42.1, 40.5, 40.5, 33.2, 32.3, 32.0, 29.2, 27.9, 23.8, 22.3, 21.2, 21.2, 20.8, 20.1, 19.8, 17.7, 13.7, 12.4. **HRMS** (ESI+): *m/z* $[M + H]^+$ calcd for $C_{34}H_{51}O_2$ 491.3884, found 491.3889.

***N*-methyl-N-(p-tolyl)hex-2-ynamide (1m):**

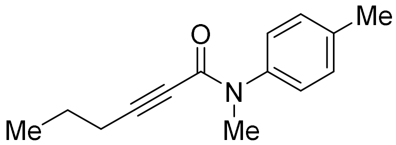

1m

Following the general procedure 4, the treatment of 2-hexynoic acid (0.11 mL, 1 mmol, 1.0 equiv), *N*-methyl-*p*-toluidine (0.15 mL, 1-2 mmol, 1.2 equiv), Et_3N (0.42 mL, 3.0 mmol, 3.0 equiv) and $SOCl_2$ (0.087 mL, 1.2 mmol, 1.2 equiv), yielded the titled compound after purification by column chromatography (SiO_2, n-heptane:AcOEt, 3:1 to 1:1) as brown oil (204.5 mg, 95%). **^{1}H-NMR** (300 MHz, $CDCl_3$): δ 7.15-6.99 (m, 4H), 3.17 (s, 3H), 2.26 (s, 3H), 1.96 (t, J = 6.8 Hz, 2H), 1.17 (h, J = 7.1 Hz, 2H), 0.59 (t, J = 7.3 Hz, 3H). **^{13}C-NMR** (75 MHz, $CDCl_3$): δ 154.5, 140.9, 137.6, 129.6, 127.0, 93.7, 75.1, 36.4, 21.1, 21.0, 20.7, 13.0. **HRMS** (ESI+) m/z [M + H]+ calcd for $C_{14}H_{18}NO$ 216.2988, found 216.2978.

***N*-(4-fluorophenyl)-*N*-methylhex-2-ynamide (1n):**

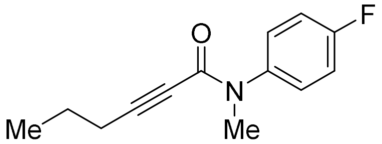

1n

Following the general procedure 4, the treatment of 2-hexynoic acid (0.11 mL, 1 mmol, 1.0 equiv), 4-fluoro-*N*-methylaniline (0.14 mL, 1-2 mmol, 1.2 equiv), Et_3N (0.42 mL, 3.0 mmol, 3.0 equiv) and $SOCl_2$ (0.087 mL, 1.2 mmol, 1.2 equiv), yielded the titled compound after purification by column chromatography (SiO_2, n-heptane:AcOEt, 3:1 to 1:1) as brown oil (199 mg, 91%). **^{1}H-NMR** (300 MHz, $CDCl_3$): δ 7.20 – 7.15 (m, 2H), 7.01 (t, J = 8.5 Hz, 2H), 3.22 (s, 3H), 2.01 (t, J = 6.9 Hz, 2H), 1.23 (h, J = 7.1, 6.7 Hz, 2H), 0.66 (t, J = 7.4 Hz, 3H). **^{13}C-NMR** (75 MHz, $CDCl_3$): δ 161.9 (d, J = 247.6 Hz), 154.5, 139.6 (d, J = 3.1 Hz), 129.2 (d, J = 8.7 Hz), 116.0 (d, J = 22.8 Hz), 94.4, 75.0, 36.5, 21.0, 20.8, 13.2. **^{19}F-NMR** (75 MHz, $CDCl_3$): δ -113.75. **HRMS** (ESI +): *m/z* $[M + H]^+$ calcd for $C_{13}H_{15}FNO$ 220.1132, found 220.1129.

6-(Dimethylamino)-6-oxohex-4-yn-1-yl 2-(3-cyano-4-isobutoxyphenyl)-4-methylthiazole-5-carboxylate (1o):

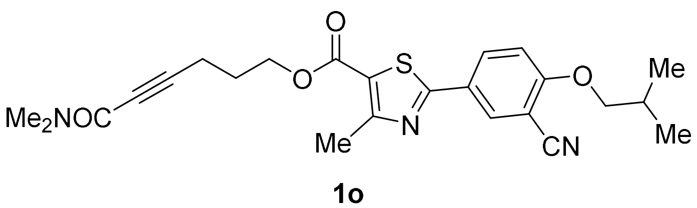

1o

Following the general procedure 3, the treatment of Febuxostat (CAS: 144060-53-7, 316.4 mg, 1.0 mmol, 1.0 equiv) with EDC·HCl (230 mg, 1.2 mmol, 1.2 equiv), Et_3N (0.21 mL, 1.5 mmol, 1.5 equiv) and 1-hydroxybenzotriazole hydrate (162.14 mg, 1.2 mmol, 1.2 equiv) in presence of precursor **I6** (170.7 mg, 1.1 mmol, 1.1 equiv) yielded the titled compound after purification by column chromatography (SiO_2, n-heptane:AcOEt,5:1 to AcOEt) as a pale yellow solid (417.3 mg, 92%). **^{1}H-NMR** (300 MHz, $CDCl_3$): δ 8.17 (d, J = 2.2 Hz, 1H), 8.07 (dd, J = 8.8, 2.2 Hz, 1H), 7.00 (d, J = 8.8 Hz, 1H), 4.39 (t, J = 6.2 Hz, 2H), 3.89 (d, J = 6.5 Hz, 2H), 3.18 (s, 3H), 2.94 (s, 3H), 2.74 (s, 3H), 2.54 (t, J = 7.0 Hz, 2H), 2.25-2.12 (m, 1H), 2.03 (p, J = 6.7 Hz, 2H), 1.07 (d, J = 6.7 Hz, 6H). **^{13}C-NMR** (75 MHz, $CDCl_3$): δ 167.5, 162.7, 161.9, 161.6, 154.6, 132.7, 132.2, 126.0, 121.4, 115.6,

112.8, 103.1, 91.1, 75.8, 74.9, 63.8, 38.4, 34.2, 28.3, 27.2, 19.2, 17.6, 16.1. **HRMS** (ESI+) *m/z* $[M + H]^+$ calcd for $C_{24}H_{28}N_3O_4S$ 454.1795, found 454.1798.

Methyl hex-2-ynoyl-*L*-valinate (1p):

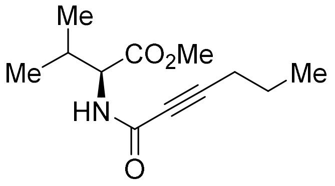

1p

Following the general procedure 3, the treatment of 2-hexynoic acid (0.11 mL, 1 mmol, 1.0 equiv) with EDC·HCl (230 mg, 1.2 mmol, 1.2 equiv), Et_3N (0.21 mL, 1.5 mmol, 1.5 equiv) and 1-hydroxybenzotriazole hydrate (162.14 mg, 1.2 mmol, 1.2 equiv) in presence of *L*-valine methyl ester hydrochloride (184.5 mg, 1.1 mmol, 1.1 equiv) yielded the titled compound after purification by column chromatography (SiO_2, n-heptane:AcOEt, 10:1) as an oil (162.2 mg, 72%). **^{1}H-NMR** (300 MHz, $CDCl_3$): δ 6.24 (d, J = 9.0 Hz, 1H), 4.58 (dd, J = 9.0, 4.9 Hz, 1H), 3.74 (s, 3H), 2.27 (t, J = 7.1, 2H), 2.23-2.19 (m, 1H), 1.58 (h, J = 7.3, 2H), 0.99 (t, J = 7.4 Hz, 3H), 0.94 (d, J = 6.9 Hz, 3H), 0.91 (d, J = 6.9 Hz, 3H). **^{13}C-NMR** (75 MHz, $CDCl_3$): δ 172.1, 153.4, 88.4, 75.4, 57.3, 52.4, 31.5, 21.3, 20.7, 19.0, 17.9, 13.7. **HRMS** (ESI+): *m/z* $[M + H]^+$ calcd for $C_{12}H_{20}NO_3$ 226.1438, found 226.1434.

Methyl *S*-benzyl-*N*-(hex-2-ynoyl)-*L*-cysteinate (1q):

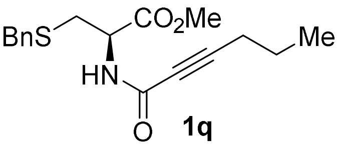

1q

Following the general procedure 3, the treatment of 2-hexynoic acid (0.11 mL, 1 mmol, 1.0 equiv) with EDC·HCl (230 mg, 1.2 mmol, 1.2 equiv), Et_3N (0.21 mL, 1.5 mmol, 1.5 equiv) and 1-hydroxybenzotriazole hydrate (162.14 mg, 1.2 mmol, 1.2 equiv) in presence of *S*-benzyl-*L*-cysteine methyl ester hydrochloride (287.9 mg, 1.1 mmol, 1.1 equiv) yielded the titled compound after purification by column chromatography (SiO_2, n-heptane:AcOEt, 10:1 to 3:1) as a yellow solid (265 mg, 83%). **^{1}H-NMR**(300 MHz, $CDCl_3$): δ 7.36 – 7.22 (m, 5H), 6.50 (d, J = 7.4 Hz, 1H), 4.82 (dt, J = 7.4, 5.2 Hz, 1H), 3.75 (s, 3H), 3.71 (s, 2H), 2.93 (dd, J = 14.0, 5.0 Hz, 1H), 2.84 (dd, J = 14.0, 5.4 Hz, 1H), 2.29 (t, J = 7.1 Hz, 2H), 1.60 (h, J = 7.2 Hz, 2H), 1.01 (t, J = 7.4 Hz, 3H). **^{13}C-NMR** (75 MHz, $CDCl_3$): δ 170.8, 153.1, 137.6, 129.0, 128.7, 127.4, 88.9, 75.2, 52.9, 52.1, 36.8, 33.2, 21.3, 20.7, 13.6. **HRMS** (ESI+) *m/z* $[M + H]^+$ calcd for $C_{17}H_{22}NO_3S$ 320.1315, found 320.1315.

Optimization studies.

Initial studies were conducted to test if copper in combination with a proper set of ligands could be utilized for the photoisomerization of alkenyl boronates. For this reason, we started the study with a collection of photoactivatable copper complexes bearing different phenanthroline-type ligands and related compounds (Scheme S1). We submitted the model substrate *Z*-**2a** under blue light irradiation (465 nm) in THF for 24 h in presence of different photoactive copper complexes. In this regard, heteroleptic complexes such as $[Cu(phen)(BINAP)]PF_6$ and $[Cu(bphen)(XantPhos)]BF_4$ were completely inactive in the photoisomerization of the corresponding alkenyl boronate *Z*-**2a**, which was recovered unaltered.[10] The homoleptic complex $[Cu(BINAP)_2]PF_6$ showed comparable inefficiency, as substrate *Z*-**2a** did not isomerize.[11] Along these lines, the monophosphine analogs $[Cu(phen)(PR_3)]Cl$ bearing different phosphine ligands, which could display available coordination sites at the copper (I) atom via ligand dissociation, were also inefficient sensitizers for this transformation.[8]

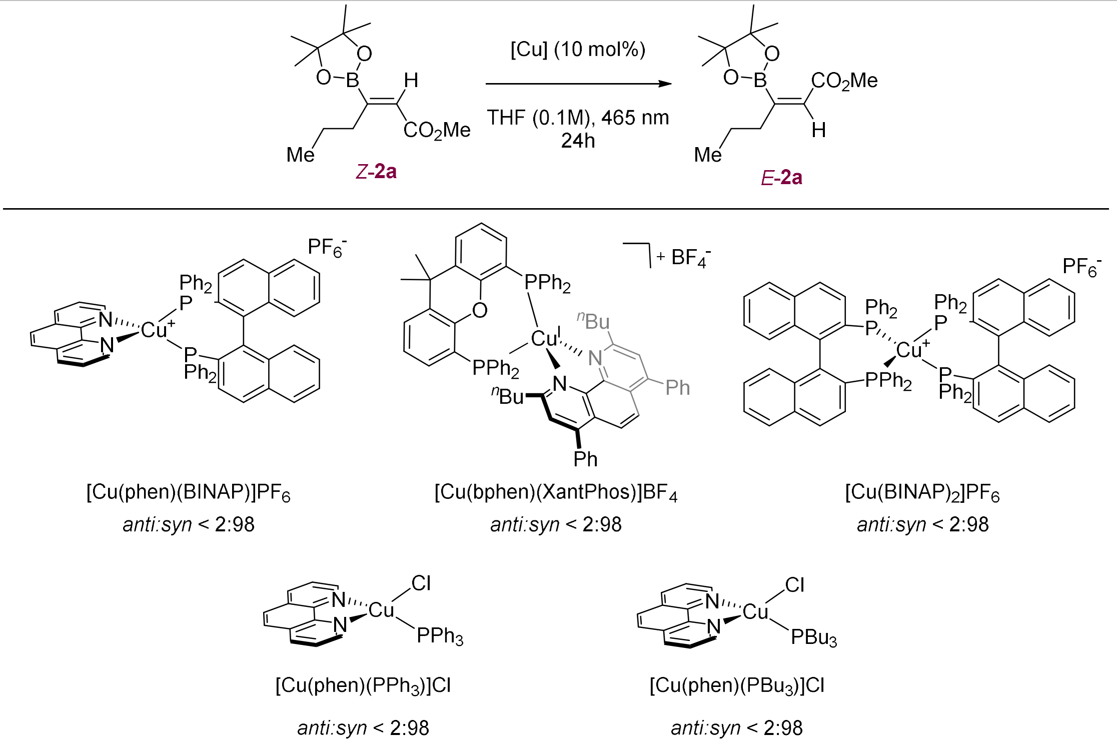

Scheme S1. Effect of copper complexes in the photoisomerization of alkenyl boronate *Z*-**2a.**

We then moved to the exploration of other copper complexes based on bisphosphine ligands to promote the photoisomerization of substrate *Z*-**2a**. In particular, we focused on Poisson's work[12] in which the employment of BINAP ligand in presence of different copper salts was particularly effective for the isomerization of β-aryl-α,β-unsaturated carbonyls. We prepared different samples of [Cu(BINAP)X] complexes[11] and they were studied as sensitizers in the photoisomerization reaction (Scheme S2). To our delight, we observed that after 24 h both $[Cu(BINAP)I]_2$ and $[Cu(BINAP)Cl]_2$ were modestly active in the photoisomerization step since ***E*-2a** (*anti*-isomer) was observed in a 26:74 to 28:72 ratio, respectively. When using the analog Cu-complex bearing bromide as the anion the isomerization took place with a higher 38:62 ratio.

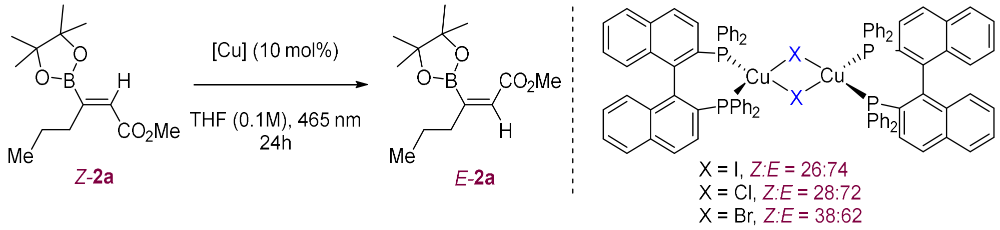

Scheme S2. Effect of BINAP-ligated copper complexes in the photoisomerization of alkenyl boronate *Z*-**2a.**

Encouraged by these results we hypothesized about the possibility of dimeric dissociation from $[Cu(BINAP)X]_2$ complexes in solution to generate a Cu(BINAP)(X) complex with a vacant coordination site available for interaction with substrate *Z*-**2a**. For this reason, we then conducted experiments varying the nature of the anion at the copper complex, which would greatly influence the formation of the required unsaturated active species (Table S1). When CuCl was employed as the copper source the photoisomerization of ***Z*-2a** took place in a 27:73 ratio favoring the *syn*-isomer (entry 1). This result is in close accordance with the isomerization of substrate Z-**2a** in presence of the isolated $[Cu(BINAP)Cl]_2$ complex (Scheme S2), suggesting that very similar speciation of the copper complex takes place in solution. To our delight, when the cationic $[Cu(OTf)]_2$·toluene complex was studied as the precursor, almost quantitative isomerization was observed favoring the formation of ***E*-2a** in a 97:3 stereoselectivity (entry 2). This result points out the requirement of a cationic Cu/BINAP complex to promote efficient coordination of the alkenyl boronate to the copper(I) center, which could be facilitated by the presence of the non-coordinating triflate anion. In comparison, when other copper salts such as CuTc and CuCN were employed, low levels of stereoselectivity were observed again (entries 3 and 4, respectively). Finally, the highly electrophilic $Cu(CH_3CN)_4PF_6$ salt was employed obtaining a similar value to that obtained for the copper halide precursors (entry 5). This result is particularly intriguing since a coordinatively unsaturated complex capable of efficiently coordinating to the alkenyl boronate is expected due to the lack of strong anionic ligands in the media. Therefore, the formation of non-active $[Cu(BINAP)_2]PF_6$ in solution is likely taking place, which would account for the low reactivity observed for this metal/ligand combination.

Table S1. Effect of copper salt in the photoisomerization of alkenyl boronate *Z*-**2a.**

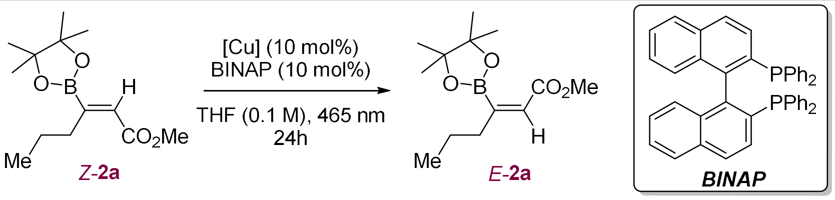

Entry	Cu salt	*anti:syn (%)*[a]
1	CuCl	27:73
2	$[CuOTf]_2$·toluene	97:3
3	CuTc	13:87
4	CuCN	15:85
5	$Cu(CH_3CN)_4PF_6$	22:78

[a]Determined in the reaction crude by ^{1}H NMR spectroscopy employing 1,3,5-trimethoxybenzene (TMB) as an internal standard.

We then studied the effect of the ligand in the photoisomerization reaction employing $[CuOTf]_2$·toluene as the copper salt (Scheme S3). When the BINAP ligand was replaced by their analogs tol-BINAP and DM-BINAP, in which the aryl group of the phosphine unit contains electron-donating groups, the efficiency of the photoisomerizations dropped from 97:3 to 77:23 and 55:45, respectively. When the naphthyl group of the BINAP ligand was replaced by a phenyl ring, such as the case of the dppbz ligand, a low 30:70 ratio of anti and syn isomers was obtained, pointing out the importance of the π-extended system for the photoisomerization step. The employment of SEGPHOS and DM-SEGPHOS led to a similar ratio favoring the presence of the syn-isomer in the photostationary state. A similar electronic dependence was obtained by comparison of the electron-deficient (R)-BTFM-GarPhos (anti:syn = 32:65) with (R)-XylGarPhos or DTB-Biphep ligands, containing electron-releasing groups at the meta position of the aryl unit bound to the phosphorous atom, and inhibited the photoisomerization reaction. Interestingly, the use of (S)-PANEPHOS furnished the anti-isomer in a 40:60 ratio. Finally, the use of ligands lacking aromatic groups either in the linker connecting the two heteroatoms, such as (S,S)-NORPHOS and (S)-BINAPINE, or in the P-substituents such as iPr-DUPHOS was reflected in no reactivity.
The experiments above highlight the requirement of an aromatic linker between the two coordinating heteroatoms, with optimal results found for the naphthyl substituent. Additionally, the use of substituents with electron-donating character at the P-aromatic substituent decreased the practicality of the method.

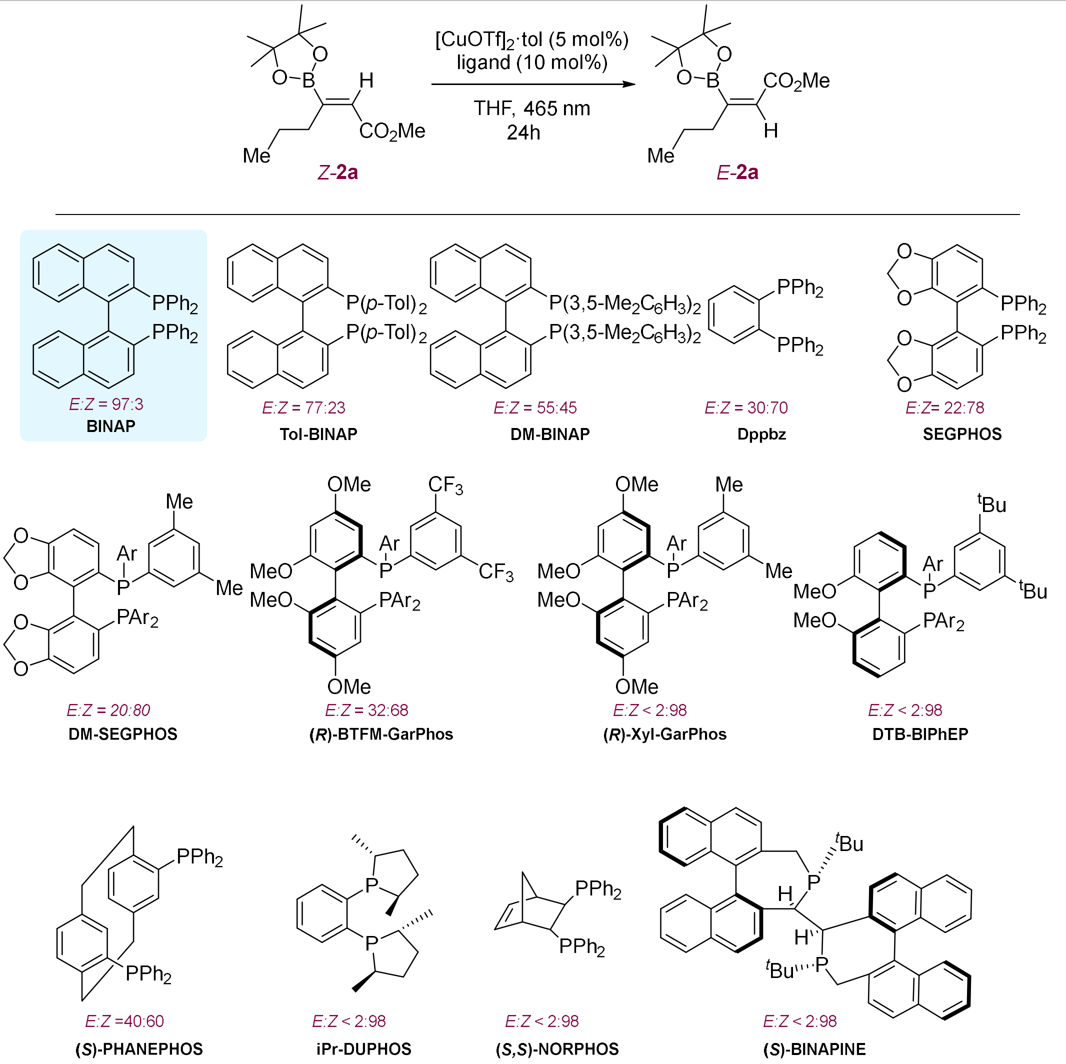

Scheme S3. Effect of different phosphine ligands in the photoisomerization of alkenyl boronate *Z*-**2a.**

External factors can also influence the sensitization of the substrate from the excited state of the photocatalyst such as the solvent, since the vibrational state of the excited species can be higher than that of the surrounding medium, and deactivation of the photocatalyst becomes a competitive pathway. Therefore, we conducted a screening of solvents to evaluate if the photoisomerization would be affected (Table S2). In comparison with THF, the use of non-coordinating solvents such as toluene led to a lower ratio of the photoisomerized product in the photostationary state, since an *anti:syn* = 48:52 ratio was observed after 24h (entry 2). Interestingly, both CH_2Cl_2 and CH_3CN, which are solvents with different polar and coordinating properties, delivered a very similar value favoring the *anti*-isomer (entries 3 and 4). Finally, the use of hexafluorobenzene delivered the worst result in terms of stereoselectivity (*anti:syn* = 34:66, entry 5).

Table S2. Effect of solvent in the photoisomerization.

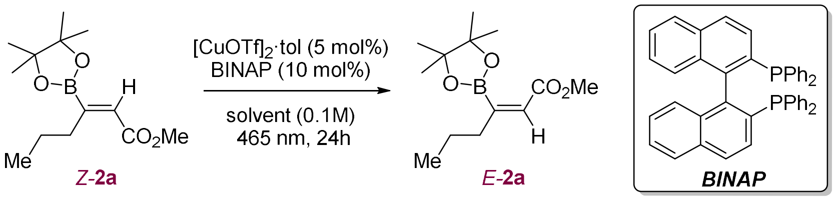

Entry	Cu salt	*anti:syn* (%)[a]
1	THF	97:3
2	Toluene	48:52
3	CH_2Cl_2	74:26
4	CH_3CN	75:25
5	C_6F_6	34:66

[a]Determined in the reaction crude by ^1H NMR spectroscopy employing 1,3,5-trimethoxybenzene (TMB) as an internal standard.

With suitable conditions to promote efficient *syn*-to-*anti* photoisomerization of alkenyl boronates with the $[CuOTf]_2$·tol/BINAP system in THF, we then commenced the quest for a tandem hydroboration/photoisomerization process (Table S3). We choose the commercially available alkyne **1a** as model substrate with B_2pin_2 as borylating system in presence of $[CuOTf]_2$·tol, NaO^tBu as a base, and MeOH in THF as solvent. All the experiments were carried out under blue light irradiation for 24 h. When BINAP was used as the only ligand present in the reaction media the corresponding *anti*-alkenyl boronate *E*-**2a** was obtained with almost complete stereoselectivity (*anti:syn* = 95:5), albeit with very low yield (< 15%, entry 1). This result highlights the effectiveness of BINAP as a ligand to promote the photoisomerization, but its inefficacy in promoting the formation of a competent copper complex capable of carrying out the hydroboration step. For this reason, we then studied the addition of monodentate ligands to enhance the catalytic performance toward the formation of the hydroborylated product, as they are known to promote this reaction. When PCy_3 was added to the reaction the yield was slightly increased to 31 %, however, the photoisomerization did not take place (entry 2). Other monophosphine complexes such as PBu_3 and $P(p\text{-}MeOC_6H_4)_3$ were also ineffective for the formation of the desired *anti*-alkenyl boronate E-**2a** since both low yields and poor stereoselectivities were systematically observed (entries 3 and 4). Although monophosphine complexes are known to perform well in Cu-catalyzed hydroboration of alkynes, the presence of the BINAP ligand could be promoting the formation of species of the type $Cu(BINAP)(PR_3)$, which would not have enough coordination sites available for the Bpin and the alkyne partners. Therefore, we then moved to the exploration of other bidentate phosphines. To our delight, when XantPhos was tested in the reaction using 1.1 equivalents of B_2pin_2, a promising yield (69%) and a better stereoselectivity were observed (entry 5). Interestingly, the dilution of the reaction from 0.1 to 0.05 M further increased the observed *anti:syn* stereoselectivity from 80:20 to 96:4 (entry 6). This result could be explained due to the relatively low solubility of BINAP in THF, which would be translated into a lower concentration of the active Cu/BINAP complex responsible for the photoisomerization step. To increase the reaction yield, the amount of B_2pin_2 was raised from 1.1 to 1.5 equiv., obtaining a 78% yield with complete stereoselectivity (entry 7). Attempts to further increase the yield by adding more B_2pin_2 were unsuccessful because the borylation of the alkenyl boronate **2a** to furnish the corresponding gem-diborylalkane becomes a competitive pathway. The reduction of the reaction time from 24 to 12h led to the formation of the borylated product with good yield (76%), but a lower stereoselectivity was obtained (*anti:syn* = 79:21), thus indicating that the photoisomerization step requires prolonged times (entry 8). Finally, the analogs $^tBu_2$-XantPhos and DPEPhos ligands were also suitable co-ligands for this transformation albeit with slightly lower efficiency (entries 9, and 10, respectively).

Table S3. Optimization for the formal *anti*-borylation of **1a**.

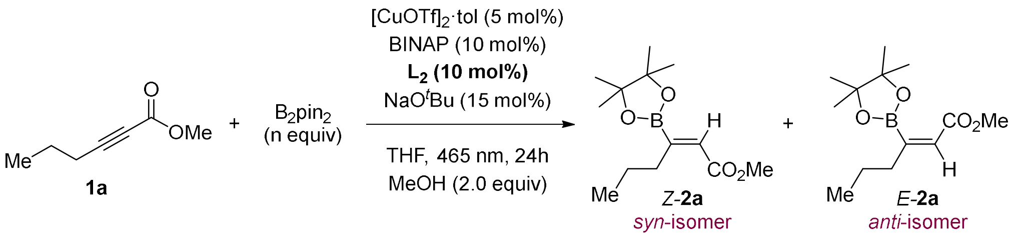

Entry	equiv. B_2pin_2 (n)	Concentr. (M)	L_2	yield (%)[a]	*anti:syn* (%)[a]
1	1.1	0.1	-	13	95:5
2	1.1	0.1	PCy_3	31	<2:98
3	1.1	0.1	PBu_3	20	28:72
4	1.1	0.1	$P(p\text{-}MeOC_6H_4)_3$	27	11:89
5	1.1	0.1	XantPhos	67	80:20
6	1.1	0.05	XantPhos	69	96:4
7	1.5	0.05	XantPhos	78	> 98:2
8[b]	1.5	0.05	XantPhos	86	69:31
9	1.5	0.05	DPEPhos	59	91:9
10	1.5	0.05	$^tBu_2$-XantPhos	69	94:6

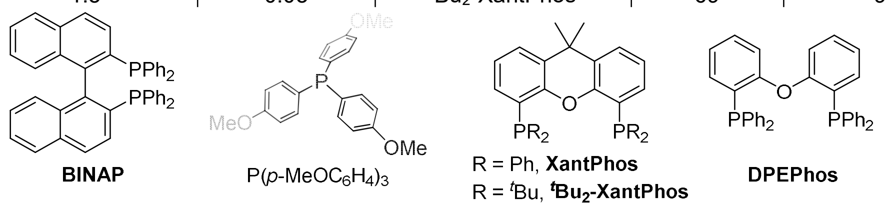

[a]Determined in the reaction crude by ^1H NMR spectroscopy employing 1,3,5-trimethoxybenzene (TMB) as an internal standard. [b]Reaction time = 12h.

General procedure for the *anti*-hydroboration of alkynes:

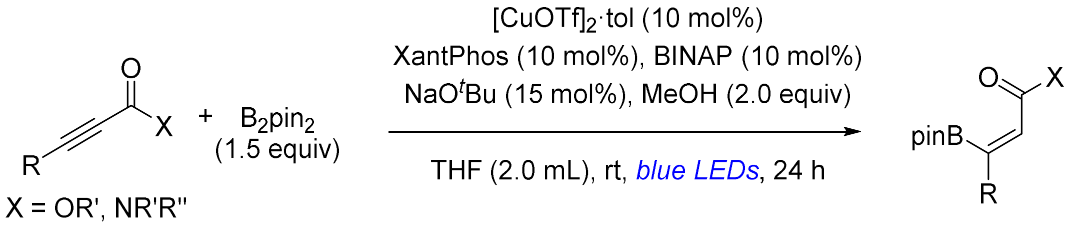

An oven-dried scintillation vial provided with a magnetic stir bar was charged with the alkyne (0.1 mmol, 1.0 equiv), $(Bpin)_2$ (38 mg, 0.15 mmol, 1.5 equiv), $[Cu(OTf)]_2$·tol (2.58 mg, 0.005 mmol, 10 mol % [Cu]), XantPhos (5.78 mg, 0.01 mmol, 10 mol%) and *rac*-BINAP (6.22 mg, 0.01 mmol, 10 mol%). After removal of the air atmosphere, the vial was flushed with Ar and THF (2.0 mL) was added under Ar atmosphere. The resulting suspension was allowed to stir for 15 minutes until the appearance of a yellowish solution. After that, MeOH (8 µL, 0.2 mmol, 2.0 equiv) and NaO^{t}Bu (7.5 µL, 2M, 0.015 mmol, 15 mol%) were added with a Hamilton syringe in this order (*the order of addition at this step is crucial since the reverse addition resulted in the formation of brown solutions which could compromise reproducibility*). The vial was allowed to stir under blue light irradiation at 20-25 °C for 24 h until completion. Then, the reaction mixture was filtered through a pad of celite, and the resulting residue was purified by column chromatography (*caution: long chromatographic columns should be avoided to prevent product decomposition*).

Methyl (*E*)-3-(4,4,5,5-tetramethyl-1,3,2-dioxaborolan-2-yl)hex-2-enoate (*E*-2a):

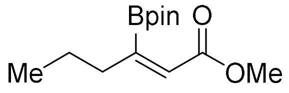

E-**2a**

Following the general procedure for the *anti*-hydroboration reaction, methyl 2-hexynoate **1a** (13.5 µL, 0.1 mmol, 1.0 equiv) afforded the titled compound after purification by column chromatography (SiO_2, n-heptane:AcOEt 10:1) as an oil (19.8 mg, 78%). **^{1}H-NMR** (300 MHz, $CDCl_3$): δ 6.00 (s, 1H), 3.71 (s, 3H), 2.24 (t, J = 7.2 Hz, 2H), 1.55 – 1.48 (m, 2H), 1.35 (s, 12H), 0.92 (t, J = 7.3 Hz, 3H). **^{13}C-NMR** (75 MHz, $CDCl_3$): δ 168.3, 125.7, 84.1, 51.7, 38.0, 25.0, 21.3, 14.0. **^{11}B NMR** (96 MHz, $CDCl_3$) δ 31.4. **HRMS** (ESI+) *m/z* $[M + H]^+$ calcd for $C_{13}H_{24}BO_4$ 255.1762, found 255.1759.

Methyl (*E*)-3-cyclohexyl-3-(4,4,5,5-tetramethyl-1,3,2-dioxaborolan-2-yl)acrylate (*E*-2b):

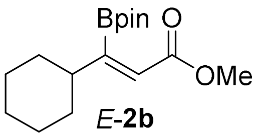

Following the general procedure for the *anti*-hydroboration reaction, alkyne **1b** (16.6 mg, 0.1 mmol, 1.0 equiv) afforded the titled compound after purification by column chromatography (SiO_2, n-heptane:AcOEt 10:1) as an oil (19.4 mg, 66%). **^{1}H-NMR** (300 MHz, $CDCl_3$): δ 5.97 (s, 1H), 3.68 (s, 3H), 2.26 – 2.06 (m, 1H), 1.83 – 1.72 (m, 4H), 1.70 – 1.60 (m, 1H), 1.35 (s, 12H), 1.26 – 1.13 (m, 5H). ^{13}C-NMR (75 MHz, $CDCl_3$): δ 168.6, 124.0, 84.1, 51.6, 44.8, 32.1, 26.4, 26.1, 25.1. **^{11}B NMR** (96 MHz, $CDCl_3$) δ 31.3. **HRMS** (APCI+) *m/z* $[M + H]^+$ calcd for $C_{16}H_{28}BO_4$ 295.2077, found 295.2074.

Methyl (*E*)-7-chloro-3-(4,4,5,5-tetramethyl-1,3,2-dioxaborolan-2-yl)hept-2-enoate (*E*-2c):

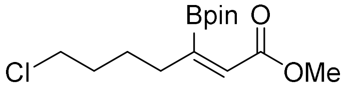

E-**2c**

Following the general procedure for the *anti*-hydroboration reaction, alkyne **1c** (17.4 mg, 0.1 mmol, 1.0 equiv) afforded the titled compound after purification by column chromatography (SiO_2, n-heptane:AcOEt 10:1) as an oil (21.5 mg, 71%). **^{1}H-NMR** (300 MHz, $CDCl_3$): δ 6.02 (s, 1H), 3.72 (s, 3H), 3.54 (t, J = 6.3 Hz, 2H), 2.31 (t, J = 7.4 Hz, 2H), 1.82 – 1.75 (m, 2H), 1.67 – 1.63 (m, 2H), 1.36 (s, 12H). **^{13}C-NMR** (75 MHz, $CDCl_3$): δ 168.2, 126.2, 84.3, 51.8, 44.9, 35.0, 32.1, 25.3, 25.0. **^{11}B NMR** (96 MHz, $CDCl_3$) δ 31.5. **HRMS** (ESI+) *m/z* $[M + H]^+$ calcd for $C_{14}H_{25}BClO_4$ 303.1529, found 303.1539.

Benzyl (*E*)-3-((3-(4,4,5,5-tetramethyl-1,3,2-dioxaborolan-2-yl)hex-2-enoyl)oxy)azetidine-1-carboxylate (*E*-2d):

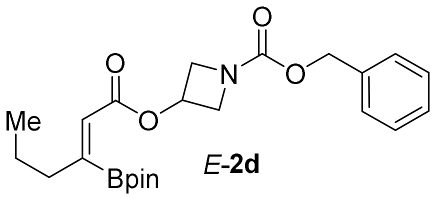

Following the general procedure for the *anti*-hydroboration reaction, alkyne **1d** (30.1 mg, 0.1 mmol, 1.0 equiv) afforded the titled compound after purification by column chromatography (SiO_2, n-heptane:AcOEt 10:1) as yellow oil (33.5 mg, 78%). **^{1}H-NMR** (300 MHz, $CDCl_3$): δ 7.34 (s, 5H), 6.02 (s, 1H), 5.24 (tt, J = 6.8, 4.3 Hz, 1H), 5.09 (s, 2H), 4.30 (dd, J = 10.1, 6.8 Hz, 5H), 4.00 (dd, J = 10.1, 4.3 Hz, 3H), 2.26 (t, J = 7.5 Hz, 2H), 1.58 – 1.45 (m, 2H), 1.33 (s, 12H), 0.93 (t, J = 7.3 Hz, 3H). **^{13}C-NMR** (75 MHz, $CDCl_3$): δ 167.0, 156.3, 136.6, 128.6, 128.2, 128.1, 124.7, 84.2, 66.9, 63.4, 56.7, 38.1, 24.9, 21.2, 14.0. **^{11}B NMR** (96 MHz, $CDCl_3$) δ 30.6. **HRMS** (ESI+) *m/z* $[M + H]^+$ calcd for $C_{23}H_{33}BNO_6$ 430.2395, found 430.2403.

(*S*)-(4-(prop-1-en-2-yl)cyclohex-1-en-1-yl)methyl (*E*)-3-(4,4,5,5-tetramethyl-1,3,2-dioxaborolan-2-yl)hex-2-enoate (*E*-2e):

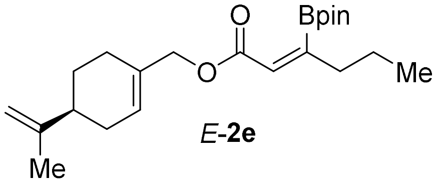

E-**2e**

Following the general procedure for the *anti*-hydroboration reaction, alkyne **1e** (24.6 mg, 0.1 mmol, 1.0 equiv) afforded the titled compound after purification by column chromatography (SiO_2, n-heptane:AcOEt 10:1) as an oil (29.2 mg, 78%). **^{1}H-NMR** (300 MHz, $CDCl_3$): δ 6.02 (s, 1H), 5.75 (s, 1H), 4.73 – 4.70 (m, 2H), 4.50 (s, 2H), 2.24 (t, J = 7.6 Hz, 2H), 2.19 – 2.05 (m, 5H), 1.72 (s, 3H), 1.57 – 1.44 (m, 4H), 1.35 (s, 12H), 0.91 (t, J = 7.4 Hz, 3H). **^{13}C-NMR** (75 MHz, $CDCl_3$): δ 168.0, 149.8, 132.8, 126.0, 125.9, 108.9, 84.1, 68.6, 41.0, 37.9, 30.6, 27.5, 26.5, 25.0, 21.3, 20.9, 14.0. **^{11}B NMR** (96 MHz, $CDCl_3$) δ 29.9. **HRMS** (ESI+) *m/z* $[M + H]^+$ calcd for $C_{22}H_{36}BO_4$ 375.2701, found 375.2699.

3-(Naphthalen-1-yl)prop-2-yn-1-yl (*E*)-3-(4,4,5,5-tetramethyl-1,3,2-dioxaborolan-2-yl)hex-2-enoate (*E*-2f):

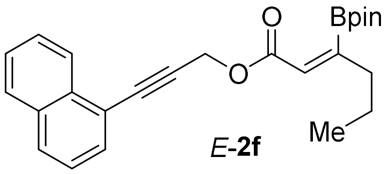

E-**2f**

Following the general procedure for the *anti*-hydroboration reaction, alkyne **1f** (27.6 mg, 0.1 mmol, 1.0 equiv) afforded the titled compound after purification by column chromatography (SiO_2, n-heptane:AcOEt 10:1) as an oil (27 mg, 67%). **^{1}H-NMR** (300 MHz, $CDCl_3$): δ 8.31 (d, J = 7.8 Hz, 1H), 7.84 (d, J = 8.3 Hz, 2H), 7.69 (d, J = 7.1 Hz, 1H), 7.59 – 7.49 (m, 2H), 7.44 – 7.39 (m, 1H), 6.12 (s, 1H), 5.12 (s, 2H), 2.29 (t, J = 7.6 Hz, 2H), 1.59 – 1.52 (m, 2H), 1.38 (s, 12H), 0.95 (t, J = 7.3 Hz, 3H). **^{13}C-NMR** (75 MHz, $CDCl_3$): δ 167.1, 133.5, 133.2, 131.1, 129.3, 128.3, 127.0, 126.6, 126.3, 125.3, 125.2, 120.1, 88.1, 84.7, 84.2, 53.1, 38.1, 25.0, 21.3, 14.0. **^{11}B NMR** (96 MHz, $CDCl_3$) δ 29.8. **HRMS** (ESI+) *m/z* $[M + H]^+$ calcd for $C_{25}H_{30}BO_4$ 405.2232, found 405.2236.

(*8R,9S,13S,14S*)-13-methyl-17-oxo-7,8,9,11,12,13,14,15,16,17-decahydro-6*H*-cyclopenta[*a*]phenanthren-3-yl (*E*)-3-(4,4,5,5-tetramethyl-1,3,2-dioxaborolan-2-yl)hex-2-enoate (*E*-2g):

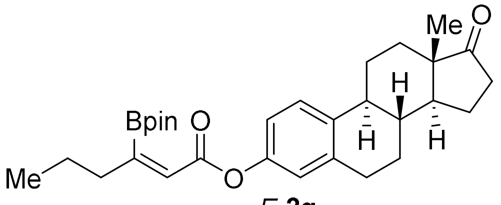

E-**2g**

Following the general procedure for the *anti*-hydroboration reaction, alkyne **1g** (36.4 mg, 0.1 mmol, 1.0 equiv) afforded the titled compound after purification by column chromatography (SiO_2, n-heptane:AcOEt 7:1) as an oil (30.5 mg, 62%). **^{1}H-NMR** (300 MHz, $CDCl_3$): δ 7.24 (d, J = 8.3 Hz, 1H), 6.88 (d, J = 8.5 Hz, 1H), 6.85-6,78 (m, 1H), 6.20 (s, 1H), 2.91 – 2.86 (m, 2H), 2.50 (dd, J = 18.3, 8.3 Hz, 1H), 2.41 – 2.26 (m, 4H), 2.15 – 1.93 (m, 5H), 1.60 – 1.48 (m, 7H), 1.29 (s, 12H), 0.97 (t, J = 7.4 Hz, 3H), 0.90 (s, 3H). **^{13}C-NMR** (75 MHz, $CDCl_3$): δ 220.9, 165.9, 148.7, 137.9, 137.2, 126.3, 125.7, 121.8, 119.1, 84.3, 50.6, 48.1, 44.3, 38.4, 38.1, 36.0, 31.7, 31.0, 29.5, 26.5, 25.8, 25.0, 21.7, 21.3, 14.1. **^{11}B NMR** (96 MHz, $CDCl_3$) δ 30.6. **HRMS** (ESI+) *m/z* $[M + H]^+$ calcd for $C_{30}H_{42}BO_5$ 493.3120, found 493.3128.

Methyl (*E*)-6-(2-(1,3-dimethyl-2,6-dioxo-1,2,3,6-tetrahydro-7H-purin-7-yl)acetoxy)-3-(4,4,5,5-tetramethyl-1,3,2-dioxaborolan-2-yl)hex-2-enoate (*E*-2h):

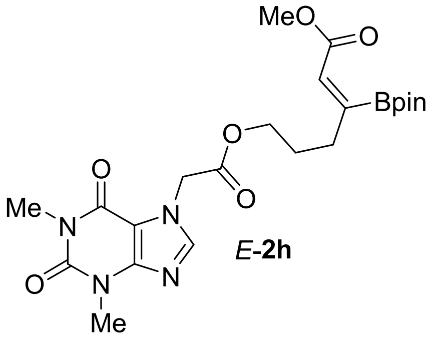

E-**2h**

Following the general procedure for the *anti*-hydroboration reaction, alkyne **1h** (36.2 mg, 0.1 mmol, 1.0 equiv) afforded the titled compound after purification by column chromatography (SiO_2, n-heptane:AcOEt 2:1 to AcOEt:MeOH (99:1)) as an oil (45.6 mg, 93%). ^{1}H-NMR (300 MHz, $CDCl_3$): δ 7.65 (s, 1H), 6.02 (s, 1H), 5.08 (s, 2H), 4.22 (t, J = 6.2 Hz, 2H), 3.72 (s, 3H), 3.63 (s, 3H), 3.38 (s, 3H), 2.35 (t, J = 7.2 Hz, 2H), 1.92 – 1.85 (m, 2H), 1.35 (s, 12H). ^{13}C-NMR (75 MHz, CDCl3): δ 168.1, 167.0, 155.4, 153.9, 151.7, 148.1, 126.6, 103.9, 84.3, 65.6, 51.8, 47.5, 31.9, 28.0, 26.9, 24.9. **^{11}B NMR** (96 MHz, CDCl3) δ 31.2. **HRMS** (ESI+) *m/z* [M + H]+ calcd for $C_{22}H_{32}BN_4O_8$ 491.2308, found 491.2304.

***Rac*-(*R*)-2,5,7,8-tetramethyl-2-((*4R,8R*)-4,8,12-trimethyltridecyl)chroman-6-yl (*E*)-3-(4,4,5,5-tetramethyl-1,3,2-dioxaborolan-2-yl)hex-2-enoate (*E*-2i):**

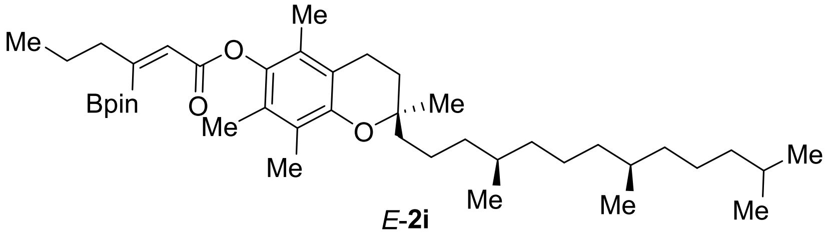

E-**2i**

Following the general procedure for the *anti*-hydroboration reaction, alkyne **1i** (52.4 mg, 0.1 mmol, 1.0 equiv) afforded the titled compound after purification by column chromatography (SiO_2, n-heptane:AcOEt 20:1) as yellow oil (62.6 mg, 96%). **^{1}H-NMR** (300 MHz, $CDCl_3$): δ 6.24 (s, 1H), 2.58 (t, J = 6.2 Hz, 2H), 2.33 (t, J = 7.6 Hz, 2H), 2.07 (s, 3H), 2.01 (s, 3H), 1.96 (s, 3H), 1.83-1.71 (m, 3H), 1.65 – 1.35 (m, 17H), 1.24 (s, 12H), 0.98 (t, J = 7.3 Hz, 3H), 0.91 – 0.82 (m, 20H). **^{13}C-NMR** (75 MHz, $CDCl_3$): δ 165.5, 149.4, 140.5, 127.2, 125.5, 125.4, 122.9, 117.4, 84.2, 75.1, 39.5, 38.5, 37.7, 37.6, 37.5, 37.4, 32.9, 32.89, 32.8, 28.1, 25.0, 24.9, 24.6, 22.9, 22.8, 21.3, 21.2, 20.7, 19.9, 19.8, 19.8, 14.1, 13.2, 12.4, 12.0. **^{11}B NMR** (96 MHz, $CDCl_3$) δ 31.2. **HRMS** (ESI+) *m/z* $[M + H]^+$ calcd for $C_{41}H_{70}BO_5$ 653.5311, found 653.5322.

(*E*)-6-methoxy-6-oxo-4-(4,4,5,5-tetramethyl-1,3,2-dioxaborolan-2-yl)hex-4-en-1-yl (*2S,4aR,7S,9aS,10S*)-1-methyl-8-methylene-13-oxo-2,7-bis((triethylsilyl)oxy)-1,2,4b,5,6,7,8,9,10,10a-decahydro-4a,1-(epoxymethano)-7,9a-methanobenzo[*a*]azulene-10-carboxylate (*E*-2j):

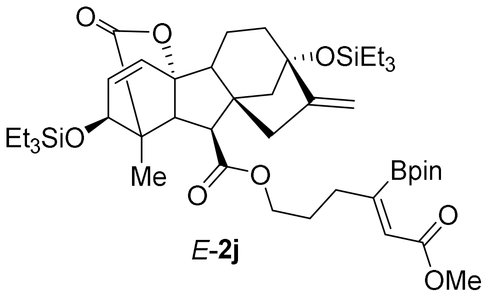

E-**2j**

Following the general procedure for the *anti*-hydroboration reaction, alkyne **1j** (69.9 mg, 0.1 mmol, 1.0 equiv) afforded the titled compound after purification by column chromatography (SiO_2, n-heptane:AcOEt 8:1) as an oil (56.2 mg, 68%). **^{1}H-NMR** (300 MHz, $CDCl_3$): (asterisk refers to the *E*-isomer) δ 6.46* (s, 0.15 H), 6.21 (d, J = 9.3 Hz, 1H), 6.01 (s, 1H), 5.75 (dd, J = 9.2, 3.4 Hz, 1H), 5.21 (s, 1H), 4.87 (s, 1H), 4.25 – 4.01 (m, 4H), 3.72 (s, 3H), 3.31 (d, J = 10.8 Hz, 1H), 2.73 (d, J = 10.8 Hz, 1H), 2.34 (t, J = 7.3 Hz, 2H), 2.25 – 1.63 (m, 10H), 1.35 (s, 12H), 1.17 (s, 3H), 1.03 – 0.85 (m, 18H), 0.72 - 0.52 (m, 12H). **^{13}C-NMR** (75 MHz, $CDCl_3$): δ 179.2, 172.0, 168.1, 157.1, 133.3, 131.6, 126.7, 107.5, 91.1, 84.3, 79.3, 70.2, 64.3, 54.3, 52.9, 51.8, 51.3, 51.1, 50.8, 44.5, 43.0, 40.7, 32.1, 27.2, 25.0, 17.2, 15.1, 7.2, 6.9, 6.6, 5.2. **^{11}B NMR** (96 MHz, $CDCl_3$) δ 30.0. **HRMS** (ESI+) *m/z* [M + H]+ calcd for $C_{44}H_{72}BO_{10}Si_2$ 828.0185, found 828.0178.

Methyl (*E*)-6-(2-(1-(4-chlorobenzoyl)-5-methoxy-2-methyl-1H-indol-3-yl)acetoxy)-3-(4,4,5,5-tetramethyl-1,3,2-dioxaborolan-2-yl)hex-2-enoate (*E*-2k):

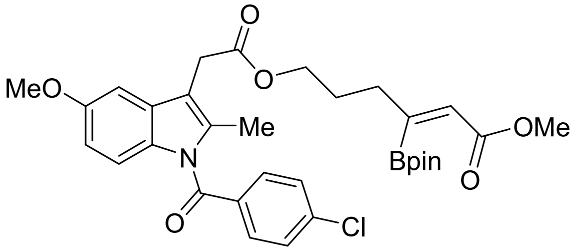

E-**2k**

Following the general procedure for the *anti*-hydroboration reaction, alkyne **1k** (69.9 mg, 0.1 mmol, 1.0 equiv) afforded the titled compound after purification by column chromatography (SiO_2, n-heptane:AcOEt 6:1) as yellow oil (48.2 mg, 89%). **^{1}H-NMR** (300 MHz, $CDCl_3$): δ 7.65 (d, J = 8.5 Hz, 2H), 7.46 (d, J = 8.5 Hz, 2H), 6.95 (d, J = 2.4 Hz, 1H), 6.85 (d, J = 9.0 Hz, 1H), 6.65 (dd, J = 9.0, 2.4 Hz, 1H), 5.90 (s, 1H), 4.10 (t, J = 6.4 Hz, 2H), 3.82 (s, 3H), 3.70 (s, 3H), 3.65 (s, 2H), 2.38 (s, 3H), 2.27 (t, J = 7.2 Hz, 2H), 1.85 – 1.78 (m, 2H), 1.32 (s, 12H). **^{13}C-NMR** (75 MHz, $CDCl_3$): δ 170.8, 168.4, 168.1, 156.2, 139.3, 136.0, 134.0, 131.3, 129.2, 126.5, 115.1, 112.7, 111.8, 101.4, 84.2, 64.3, 55.8, 51.8, 32.0, 30.5, 27.1, 24.9, 13.4. **^{11}B NMR** (96 MHz, $CDCl_3$) δ 30.4. **HRMS** (ESI+) *m/z* $[M + H]^+$ calcd for $C_{32}H_{38}BClNO_8$ 610.2374, found 610.2373.

(*S,Z*)-3-(2-((*1R,3aS,7aR,E*)-1-((*2R,5R,E*)-5,6-dimethylhept-3-en-2-yl)-7a-methyloctahydro-4*H*-inden-4-ylidene)ethylidene)-4-methylenecyclohexyl (*E*)-3-(4,4,5,5-tetramethyl-1,3,2-dioxaborolan-2-yl)hex-2-enoate (*E*-2l):

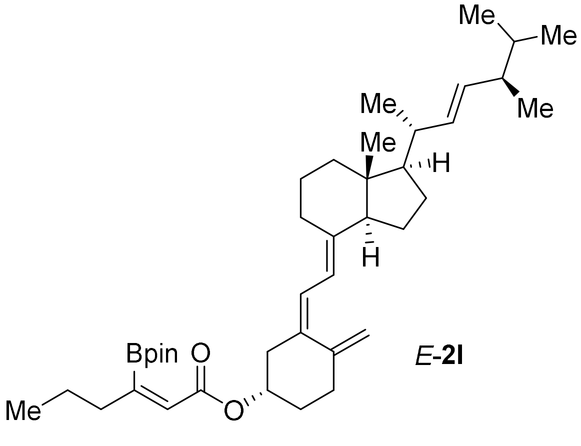

E-**2l**

Following the general procedure for the *anti*-hydroboration reaction, alkyne **1k** (49.1 mg, 0.1 mmol, 1.0 equiv) afforded the titled compound after purification by column chromatography (SiO_2, n-heptane:AcOEt 20:1) as yellow oil (37.1 mg, 60%). **^{1}H-NMR** (300 MHz, $CDCl_3$): δ 6.52 (d, J = 11.1 Hz, 1H), 6.32 (d, J = 11.1 Hz, 1H), 6.01 (s, 1H), 5.28 – 5.16 (m, 4H), 5.15 – 5.09 (m, 1H), 2.66 – 2.37 (m, 4H), 2.27 – 2.19 (m, 4H), 1.90 – 1.72 (m, 9H), 1.56 – 1.44 (m, 5H), 1.35 (s, 12H), 1.07 – 0.99 (m, 5H), 0.96 – 0.90 (m, 6H), 0.88 – 0.79 (m, 10H). **^{13}C-NMR** (75 MHz, $CDCl_3$): δ 167.8, 150.0, 135.6, 132.3, 131.4, 130.8, 126.7, 125.7, 124.8, 123.4, 84.0, 70.4, 56.5, 44.0, 43.0, 39.5, 37.7, 37.2, 33.3, 31.3, 31.0, 27.7, 26.2, 25.0, 24.4, 23.1, 21.5, 21.3, 20.1, 19.8, 19.2, 19.1, 18.5, 17.8, 14.1. ^{11}B NMR (96 MHz, $CDCl_3$) δ 31.0. **HRMS** (ESI+) m/z $[M + Na]^+$ calcd for $C_{40}H_{63}BNaO_4$ 641.4712, found 641.4728.

(*E*)-*N*-methyl-3-(4,4,5,5-tetramethyl-1,3,2-dioxaborolan-2-yl)-*N*-(p-tolyl)hex-2-enamide (*E*-2m):

Following the general procedure for the *anti*-hydroboration reaction, alkyne **1m** (21.5 mg, 0.1 mmol, 1.0 equiv) afforded the titled compound after purification by column chromatography (SiO_2, n-heptane:AcOEt 5:1) as yellow oil (24 mg, 70%). ^{1}H-**RMN** (300 MHz, $CDCl_3$): δ 7.23 (d, J = 8.0 Hz, 2H), 7.04 (d, J = 7.7 Hz, 2H), 5.60 (s, 1H), 3.44 (s, 3H), 2.39 (s, 3H), 2.30 (t, J = 7.6 Hz, 2H), 1.50-1.35

(m, 2H), 1.27 (s, 12H), 0.85 (t, J = 7.1 Hz, 3H). **^{13}C-NMR** (75 MHz, $CDCl_3$): δ 174.6, 139.0, 138.7, 130.6, 126.3, 117.8, 80.5, 39.1, 34.5, 25.5, 21.3, 20.6, 14.2. **^{11}B NMR** (96 MHz, $CDCl_3$) δ 14.1. **HRMS** (ESI+) *m/z* $[M + H]^+$ calcd for $C_{20}H_{31}BNO_3$ 344.2392, found 344.2390.

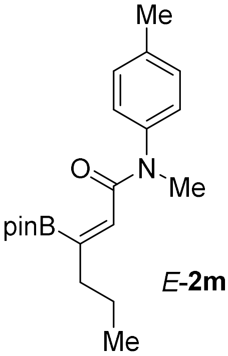

E-**2m**

(*E*)-*N*-(4-fluorophenyl)-*N*-methyl-3-(4,4,5,5-tetramethyl-1,3,2-dioxaborolan-2-yl)hex-2-enamide (*E*-2n):

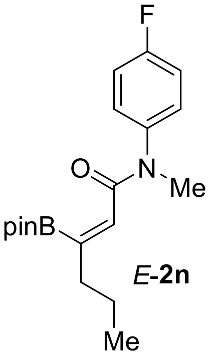

E-**2n**

Following the general procedure for the *anti*-hydroboration reaction, alkyne **1n** (21.9 mg, 0.1 mmol, 1.0 equiv) afforded the titled compound after purification by column chromatography (SiO_2, n-heptane:AcOEt 5:1) as pale yellow solid (22.6 mg, 65%). **^{1}H-NMR** (300 MHz, $CDCl_3$): δ 7.18 – 7.10 (m, 4H), 5.56 (s, 1H), 3.43 (s, 3H), 2.30 (t, J = 7.6 Hz, 2H), 1.47 – 1.39 (m, 2H), 1.27 (s, 12H), 0.85 (t, J = 7.3 Hz, 3H). **^{13}C-NMR** (75 MHz, $CDCl_3$): δ 174.6, 162.4 (d, J = 249.8 Hz), 137.4, 128.6 (d, J = 8.8 Hz), 117.4 (d, J = 27.9 Hz), 116.9, 80.6, 39.2, 34.6, 25.5, 20.6, 14.2. ^{11}B NMR (96 MHz, $CDCl_3$) δ 14.1. **HRMS** (ESI+) *m/z* $[M + H]^+$ calcd for $C_{19}H_{28}BFNO_3$ 348.2141, found 348.2141.

(*E*)-6-(dimethylamino)-6-oxo-4-(4,4,5,5-tetramethyl-1,3,2-dioxaborolan-2-yl)hex-4-en-1-yl 2-(3-cyano-4-isobutoxyphenyl)-4-methylthiazole-5-carboxylate (*E*-2o):

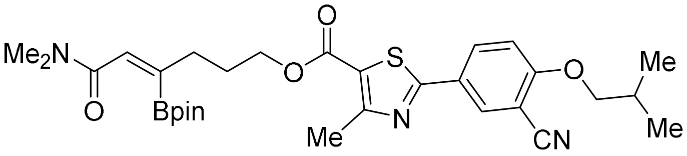

E-**2o**

Following the general procedure for the *anti*-hydroboration reaction, alkyne **1o** (45.4 mg, 0.1 mmol, 1.0 equiv) afforded the titled compound after purification by column chromatography (SiO_2, n-heptane:AcOEt 2:1) as pale yellow solid (36.6 mg, 63%). **^{1}H-NMR** (300 MHz, $CDCl_3$): δ 8.17 (d, J = 2.2 Hz, 1H), 8.09 (dd, J = 8.8, 2.3 Hz, 1H), 7.01 (d, J = 8.8 Hz, 1H), 6.22 (s, 1H), 4.33 (t, J = 6.5 Hz, 2H), 3.90 (d, J = 6.5 Hz, 2H), 3.16 (s, 6H), 2.76 (s, 3H), 2.58 (t, J = 7.1 Hz, 2H), 2.25 – 2.16 (m, 1H), 2.08 – 1.97 (m, 2H), 1.25 (s, 12H), 1.08 (d, J = 6.7 Hz, 6H). **^{13}C-NMR** (75 MHz, $CDCl_3$): δ 174.2, 167.4, 162.7, 162.1, 161.1, 132.8, 132.3, 126.0, 122.1, 116.4, 115.5, 112.8, 103.2, 80.6, 75.9, 65.3, 37.5, 37.4, 28.7, 28.3, 26.7, 25.4, 25.0, 24.7, 19.2, 17.7. ^{11}B NMR (96 MHz, $CDCl_3$) δ 13.2. **HRMS** (ESI+) *m/z* $[M + H]^+$ calcd for $C_{30}H_{41}BN_3O_6S$ 582.2804, found 582.2802.

Methyl (*E*)-(3-(4,4,5,5-tetramethyl-1,3,2-dioxaborolan-2-yl)hex-2-enoyl)-*L*-valinate (*E*-2p):

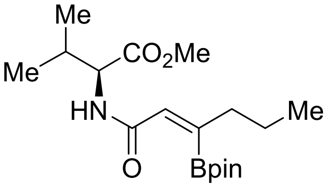

E-**2p**

Following the general procedure for the *anti*-hydroboration reaction, alkyne **1p** (22.5 mg, 0.1 mmol, 1.0 equiv) afforded the titled compound after purification by column chromatography (SiO_2, n-heptane:AcOEt 2:1) as pale yellow solid (22.6 mg, 64%). **^{1}H-NMR** (300 MHz, $CDCl_3$): δ 6.48 (d, J = 8.5 Hz, 1H), 5.94 (s, 1H), 4.66 (dd, J = 8.7, 4.5 Hz, 1H), 3.76 (s, 3H), 2.32 (t, J = 7.5 Hz, 2H), 2.25 – 2.17 (m, 1H), 1.54 (q, J = 7.4 Hz, 2H), 1.25 (s, 12H), 0.98-0.88 (m, 9H). **^{13}C-NMR** (75 MHz, $CDCl_3$): δ 172.8, 171.6, 120.4, 81.2, 58.8, 52.6, 34.9, 31.9, 25.5, 25.1, 20.7, 19.0, 17.9, 14.2. **^{11}B NMR** (96 MHz, $CDCl_3$) δ 16.6.**HRMS** (ESI+) *m/z* $[M + H]^+$ calcd for $C_{18}H_{33}BNO_5$ 354.2446, found 354.2449. The enantiomeric excess (e.e.) was determined by HPLC-DAD using a CHIRALPAK® ID column (hexane/i-PrOH 90:10, 1 mL/min, 205 nm, 25ºC); tr (major) = 7.75 min, tr (minor) = 5.17 min (94% ee).[13]

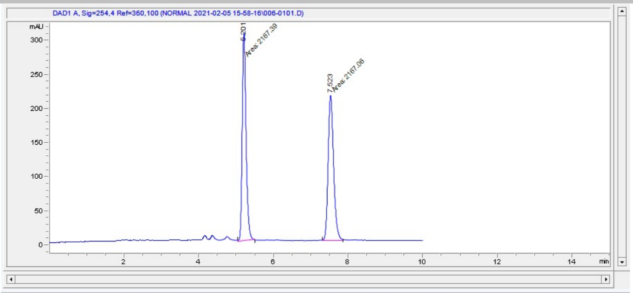

#	Time	Area	Height	Width	Area%	Symmetry
1	5.201	2167.4	304.3	0.1187	50.004	0.755
2	7.523	2167.1	212.1	0.1703	49.996	0.789

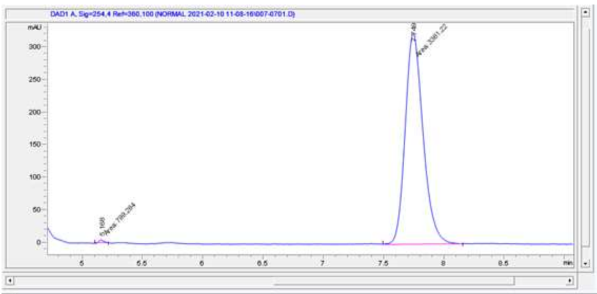

#	Time	Area	Height	Width	Area%	Symmetry
1	5.168	98.9	60.9	0.0271	2.859	0.757
2	7.749	3361.2	322.7	0.1736	97.141	0.787

Methyl (*E*)-S-benzyl-N-(3-(4,4,5,5-tetramethyl-1,3,2-dioxaborolan-2-yl)hex-2-enoyl)-*L*-cysteinate (*E*-2q):

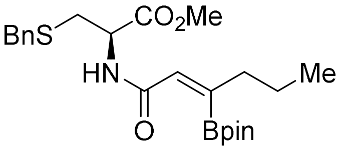

E-**2q**

Following the general procedure for the *anti*-hydroboration reaction, alkyne **1q** (31.9 mg, 0.1 mmol, 1.0 equiv) afforded the titled compound after purification by column chromatography (SiO_2, n-heptane:AcOEt 2:1) as white solid (34 mg, 76%). ^{1}H-NMR (300 MHz, $CDCl_3$): δ 7.40 – 7.21 (m, 5H), 6.51 (d, J = 7.5 Hz, 1H), 5.90 (s, 1H), 4.92 (dt, J = 8.0, 5.0 Hz, 1H), 3.75 (s, 3H), 3.69 (s, 2H), 2.94 (s, 1H), 2.31 (t, J = 7.6 Hz, 2H), 1.62-1.48 (m, 2H), 1.28 (s, 12H), 0.94 (t, J = 7.4 Hz, 3H). ^{13}C-NMR (75 MHz, $CDCl_3$): δ 171.2, 170.6, 129.1, 128.8, 127.4, 122.0, 81.9, 53.0, 36.8, 35.5, 33.7, 25.3, 25.2, 20.8, 14.2. **^{11}B NMR** (96 MHz, $CDCl_3$) δ 16.9. **HRMS** (ESI+) *m/z* $[M + H]^+$ calcd for $C_{23}H_{35}BNO_5S$ 448.2324, found 448.2337. The enantiomeric excess (e.e.) was determined by HPLC-DAD using a CHIRALPAK® ID column (hexane/i-PrOH 90:10, 1 mL/min, 205 nm, 25ºC); tr (major) = 12.25 min, tr (minor) = 6.99 min (90% ee).[11]

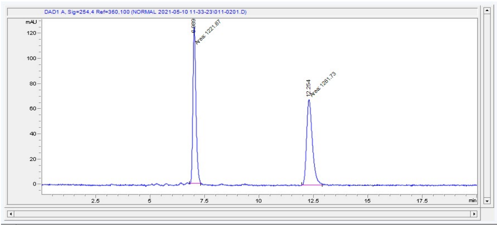

#	Time	Area	Height	Width	Area%	Symmetry
1	6.989	1221.7	124.5	0.1636	49.193	0.739
2	12.254	1261.7	68.1	0.3089	50.807	0.673

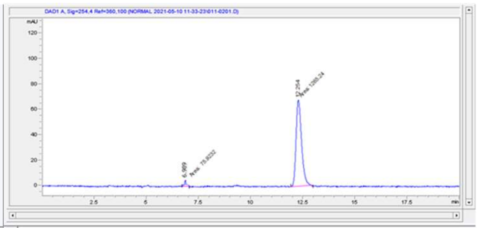

#	Time	Area	Height	Width	Area%	Symmetry
1	6.989	75.9	5.2	0.1636	5.661	0.739
2	12.254	1265.2	68.3	0.3087	94.339	0.69

Unsuccesful substrates

During optimization studies we tried to extend the method to non-activated internal alkynes bearing different polar groups at the propargylic position with a dual function: 1) as an element of regiocontrol during the hydroboration step, and 2) as a point for interaction with the vacant p orbital of the boron atom once the substrate isomerizes, leading to a favorable interaction which could impart directionality in terms of *anti*-stereoselectivity of the process. For this study, we first studied the photoisomerization of different *syn*-alkenyl boronates[14] in presence of the $[CuOTf]_2$·tol/BINAP system (Scheme S4). From this study, it is clear that the interaction with the Cu/BINAP catalyst is almost negligible, as no isomerization was observed for substrates Z-**2r**, Z-**2s**, Z-**2t**, Z-**2u**, and Z-**2v**, bearing an aryl sulfone, alcohol, ether, and NHTs groups, respectively. However, when the naphthyl thioether Z-**2w** was studied the corresponding *anti*-Z-**2w** was observed in the reaction crude in a 65:35 ratio in favor of the *anti*-isomer.

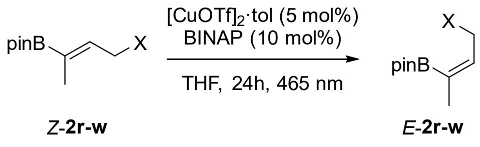

X	*anti:syn*[a]
-SO_2Ph, *Z*-**2r**	< 2:98
-SO_2(2-Py), *Z*-**2s**	< 2:98
-OH, *Z*-**2t**	< 2:98
-OBn, *Z*-**2u**	< 2:98
-NHTs, *Z*-**2v**	< 2:98
-S(1-napthyl), *Z*-**2w**	65:35

[a]Determined in the reaction crude by ^{1}H-NMR spectroscopy

Scheme S4. Isomerization studies on non-activated alkenyl boronates.

With this preliminary result, we studied the feasibility of promoting the borylation/isomerization sequence starting from alkyne **1w** with the optimized conditions for the *anti*-hydroboration protocol shown in Scheme S5. However, the hydroboration reaction did not take place with the BINAP/XantPhos dual system. For this reason, we tested the combination of a monodentate ligand with BINAP to promote the borylation, but as expected from the optimization studies this mixture did not afford the hydroborylated product in any case (Scheme S5).

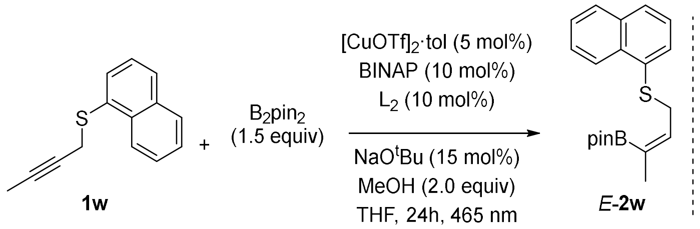

L_2	*anti:syn*[a]
XantPhos	-
PCy_3	-
$PtBu_3$	-
PBu_3	-

Scheme S5. Attempts to develop a tandem *anti*-borylation protocol.

Apart from esters and amides, ketones could be useful reagents in the anti-hydroboration reaction. However, when we tested the optimized reaction conditions on substrate **1y** we observed the degradation of the substrate in the reaction crude, and we did not detect signals in the ^{1}H NMR spectrum suggesting the formation of the desired product (Scheme S6a). Additionally, aryl-substituted alkynoate **1x** was also ineffective for this transformation since no isomerization was observed, and the corresponding *syn*-hydroborylated product undergoes protodeborylation in the reaction media (Scheme S6b), indicating that these conditions are not well tolerated by these substrates. Additionally, the use of other diboron compounds such as B_2cat_2, $B_2(OH)_2$ and BpinBdan led to deomposition of the borylated product or poor levels of isomerization, as in the case of using BpinBdan (Scheme 6c)

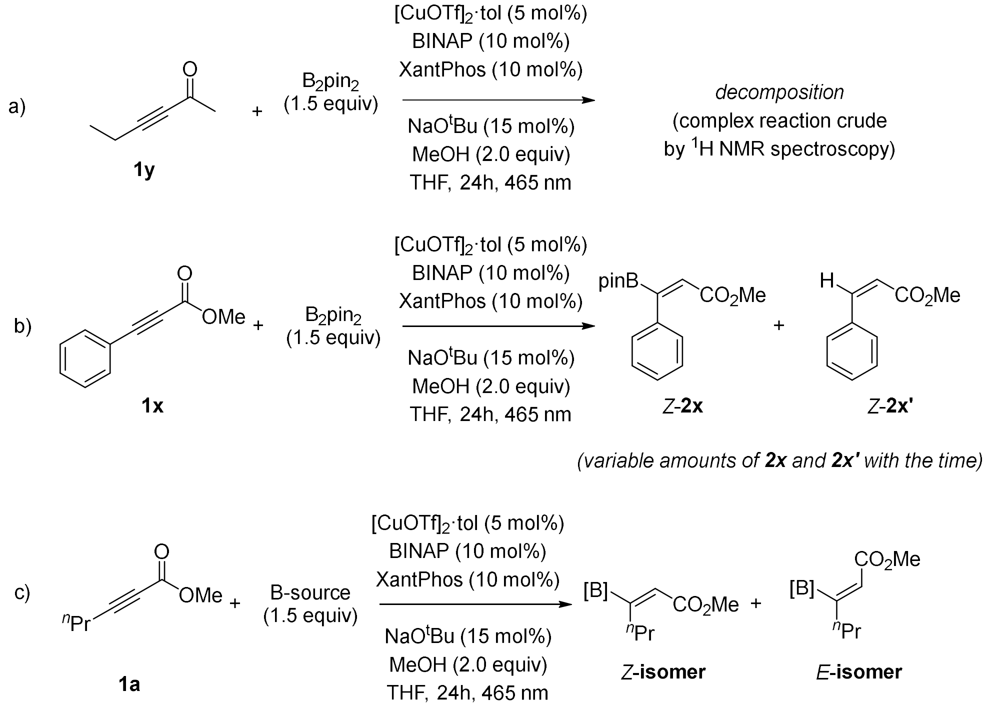

B-source	Result
B_2cat_2	decomposition
$B_2(OH)_2$	no reaction
BpinBdan	63%, *Z*:*E* = 90:10

Scheme S6. Unsucessful substrates.

Additive effects

To further understand the potential of this method a series of additives were screened to study their impact on the overall process. These types of studies allow for rapid screening of functional groups potentially present in other substrates with a minimal change in the optimized reaction conditions (Scheme S7). Initial experiments were performed in presence of the acid **3a** to test the feasibility of acidic groups in the media, albeit the reactivity was completely inhibited, which was ascribed to the potential degradation or even lack of formation of the required copper-alkoxide species. On the contrary, the reaction tolerated the presence of the Lewis acid $PhB(OH)_2$ **3b** with no erosion of the reactivity and high stereoselectivity (anti:syn = 95:5). We then studied different amines and related species, which could potentially coordinate to the catalytically relevant copper species and retard or inhibit the catalysis. From this study becomes clear that simple amine groups such as aniline (**3c**) were perfectly compatible with the reaction conditions, as no depletion of both reactivity and stereoselectivity was observed (72%, anti:syn = 92:8). However, the presence of dibasic species such as 7-azaindole (**3d**) and imidazole (**3e**) impacts negatively the photoisomerization step. In this latter case, when the basic NH position was methylated (**3f**) the hydroboration/photoisomerization sequence took place with excellent results (85% yield and almost complete stereoselectivity). Other potentially coordinating bases such as 2-mercaptopyridine **3g** delivered the corresponding *anti*-isomer with high stereoselectivity but lower yield, indicating the potential incompatibility of this group in the hydroboration step. A similar result in terms of reactivity was attained by the addition of the acetophenone imine (**3h**), albeit no photoisomerization took place in this case. On the contrary, the more basic alkyl amines **3i** and **3j** did not exhibit a deleterious effect in the obtaining of the anti-hydroborylated product E-**2a**, since both good yield and stereoselectivities were observed (>70% yield, anti:syn = 95:5). Interestingly, other aromatic amines such as **3k** bearing potentially S-coordinating heteroatoms afforded the hydroborylated product with 60% yield, but poor stereoselectivity (anti:syn = 20:80). We then studied the effect of adding alkenylated additives to the reaction media. The presence of the coordinating 1,5-cyclooctadiene (**3l**) was well tolerated, with no signals of deleterious effect for the catalysis of either the hydroborylation or the photoisomerization step. The highly reactive 3-methyl cyclohexanone (**3m**) also delivered preferentially the anti-isomer, although it had a slight effect on the reaction yield (60%) and a lower stereoselectivity was obtained (anti:syn = 90:10). Conversely, the addition of the maleimide **3n** resulted in high reactivity (78% yield), but the stereoselectivity dropped to an anti:syn ratio of 27:30, thus indicating that this species could act as a quencher of the photoactive Cu/BINAP complex. Finally, other highly reactive systems were tested such as the ketone **3o**, which had a remarkable impact on the reaction yield, and the disulfide **3p** which did not affect the efficiency of the method.

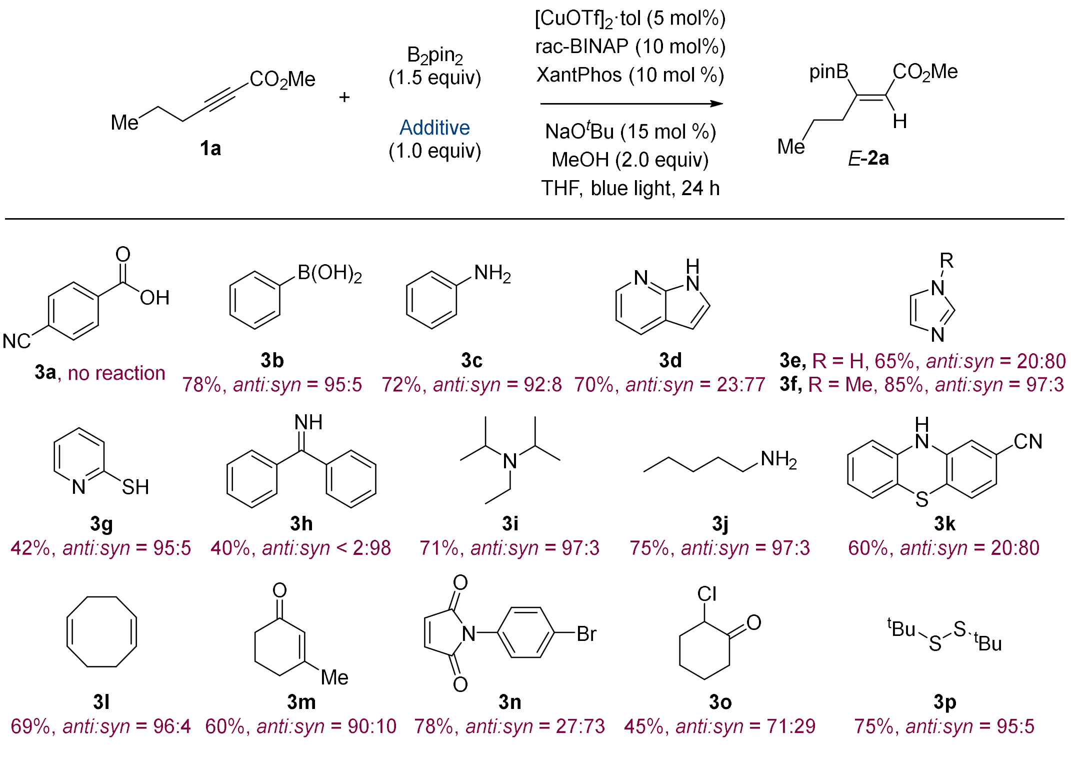

Scheme S7. Additive effects.

Mechanistic experiments.

HRMS studies.

To better understand the potential equilibration of BINAP- and XantPhos/Cu complexes in the media we performed a series of experiments based on HRMS. Firstly, we studied different solutions of each ligand in combination with $[CuOTf]_2$·toluene in THF by HRMS. When a 1:1 mixture of [Cu] and BINAP was analyzed by electrospray we detected an ion at m/z = 685.1256 assigned to the corresponding [BINAP + Cu]+ species that display a similar isotopic distribution pattern to that shown by the theoretical simulation (Figure S1a). We also detected the corresponding complex in which two units of BINAP are coordinated with the copper atom at m/z = 1307.3213 [2BINAP + Cu]+ (Figure S1b) and other complexes in which the results suggest potential oxidation of one of the phosphines in different ratios.

a) Comparison of isotopic distribution by ESI$^+$ for the [BINAP + Cu] peak

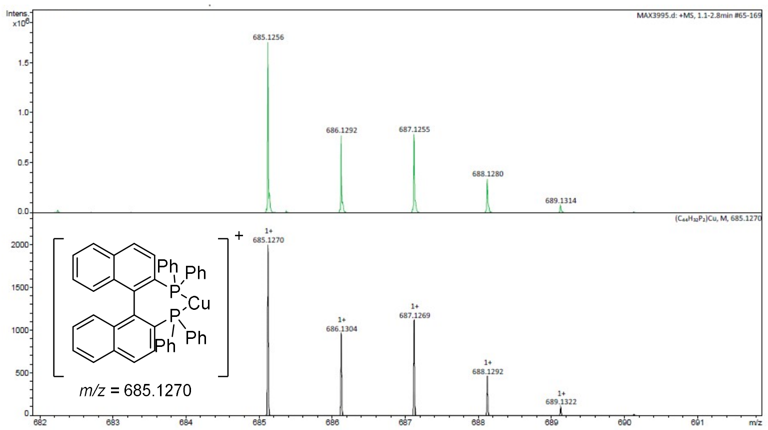

b) Comparison of isotopic distribution by ESI$^+$ for the [2BINAP + Cu] peak

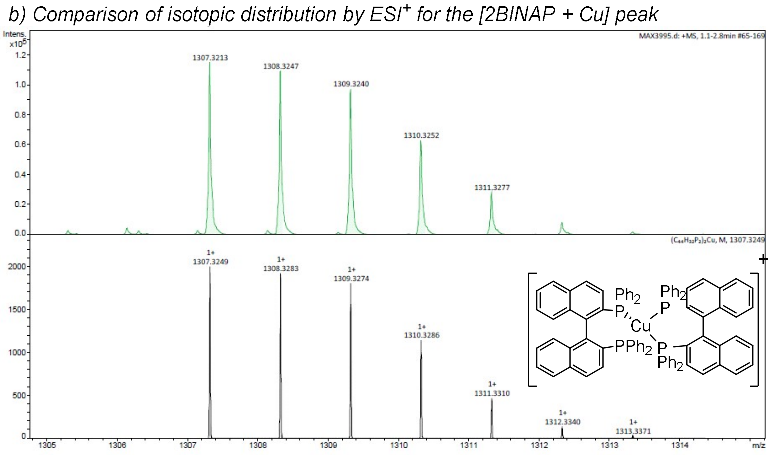

Figure S1. HRMS determination of Cu/BINAP complexes in THF solution.

The analysis of this mixture by MALDI showed as a base peak a compound without copper at m/z = 1497.2, assigned to the BINAP dimer with an additional unknown group. The two [BINAP + Cu]+ and [2BINAP + Cu]+ complexes were detected, along with complexes in which the phosphorous atom is oxidized ([BINAP + Cu + O]+, m/z = 701.2; [2BINAP + Cu + O]+, m/z = 1323.3; [2BINAP + Cu + 2O]+, m/z = 1339.3). This analysis revealed the formation of a complex with two copper atoms at m/z = 1386.3 and 1396.2. By using the FAB technique, the corresponding [BINAP + Cu]+ complex was observed, along with a dimer from BINAP (m/z = 1323).

When a 2:1 mixture of [Cu] and BINAP was studied, the amount of the corresponding complexes assigned to the [BINAP + Cu]+ and [2BINAP + Cu]+ increased in all the techniques (for results using the ESI+ technique see Figure S2). Importantly, the amount of the relative intensity of [BINAP + Cu]+ increased when the 2:1 [Cu]/BINAP solution was analyzed in comparison with the 1:1 solution, in which the [2BINAP + Cu]+ complex appears with more intensity.

a) analysis of the 1:1 [Cu]/BINAP mixture by ESI⁺

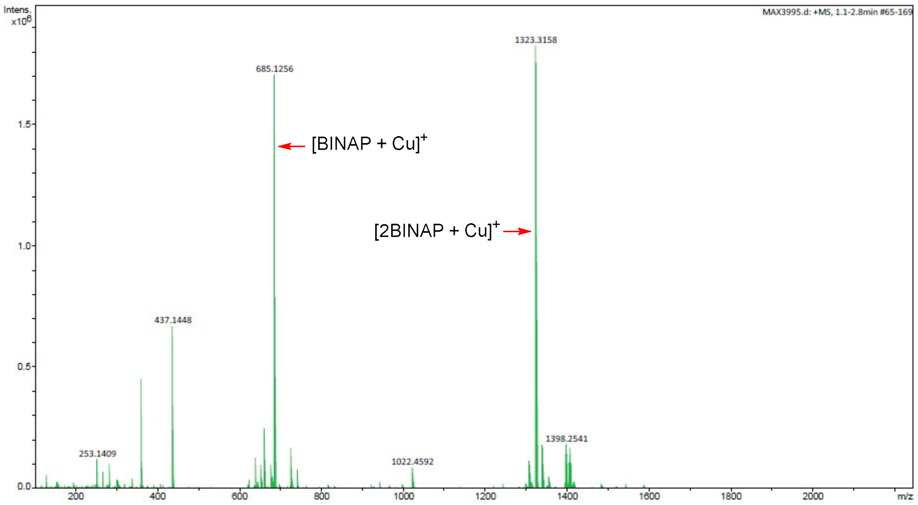

b) analysis of the 2:1 [Cu]/BINAP mixture by ESI⁺

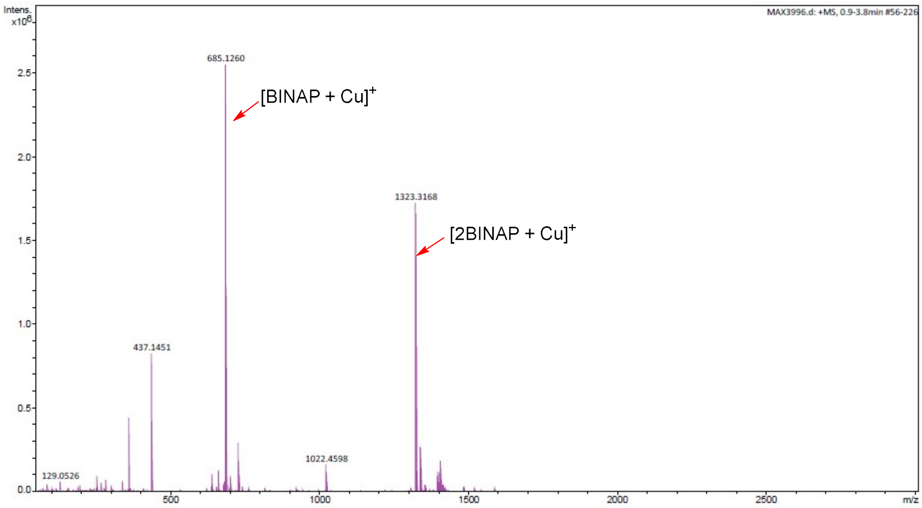

Figure S2. Analysis of the Cu/BINAP solutions in THF.

The analysis of a 1:1 mixture of [Cu] and XantPhos gave similar results to that obtained with the BINAP ligand. By using the ESI+ technique, the corresponding [XantPhos + Cu]+ complex was detected with high intensity at m/z = 641.1205 (Figure S3). Dimers corresponding with the formula [2XantPhos + Cu + O]+ and [2XantPhos + Cu + 2O]+ (m/z = 1235 and 1251, respectively). By MALDI and FAB peaks assigned to the [XantPhos + Cu]+ complex were observed, along with different oxidation products.

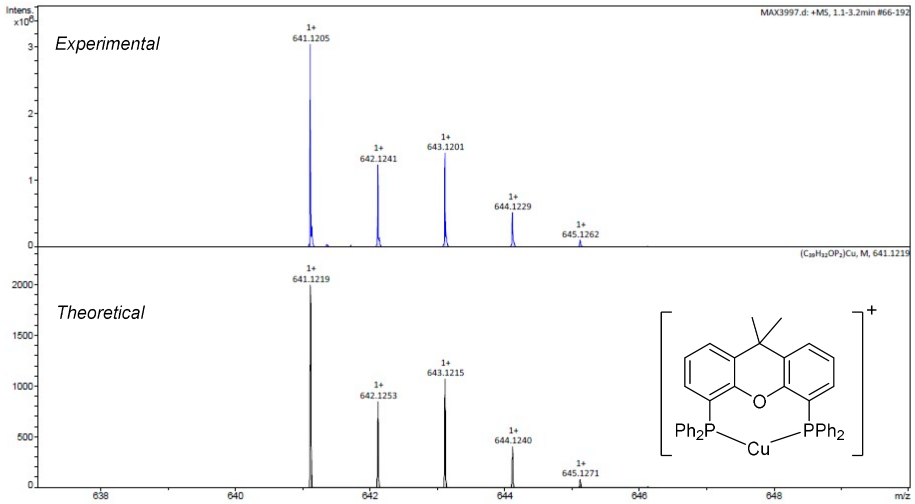

Figure S3. Cu/XantPhos complex determined in THF solutions.

Finally, when a 1:1:1 mixture of [Cu] and both ligands was analyzed by the three techniques we observed each copper complex with one ligand [XantPhos + Cu]+ and [BINAP + Cu]+, but also the oxidized homo- and heteroleptic dimers, such as the [XantPhos + BINAP + Cu +O]+ at m/z = 1279.3121 by electrospray (Figure S4), and [XantPhos + BINAP + Cu + 2O]+ at m/z = 1295.3066 by ESI+ and MALDI.

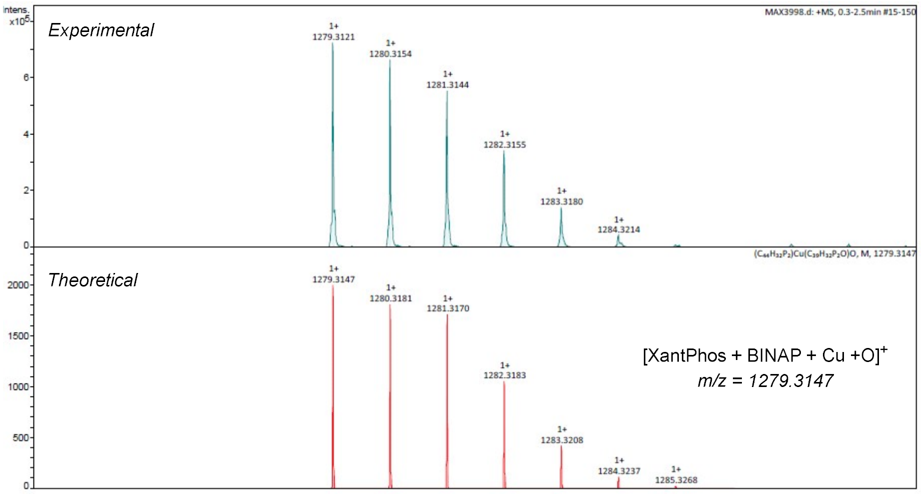

Figure S4. Mixed complexes determined by HRMS in THF solutions.

As a conclusion both ligand-copper complexes co-exist when they are disolved in THF. In addition, some oxidation products have been detected, which could correspond to the reduction of some quantities of Cu(II) in the $[CuOTf]_2$·toluene salt. Interestingly, the formation of the homo- and heteroleptic complexes using the 1:1 stoichiometry is feasible in solution.

^{31}P NMR studies.

Initially, we recorded the corresponding ^{31}P NMR spectrum of each ligand in THF-d_8, from which the corresponding chemical shift for free XantPhos (δ = -20.05 ppm) and BINAP (δ = -17.28 ppm) could be obtained (Figure S5a and b, respectively).

The combination of $[Cu(OTf)]_2$·toluene and BINAP ([Cu]:BINAP = 1:1) in THF-d_8 led to partial oxidation of the ligand since a new signal appeared at 30.92 ppm (**1**).[15] Additionally, we observed the complete disappearance of the free BINAP, while two new species appeared in solution at 11.75 ppm (**2**) and -5.21 ppm (**3**), which were assigned to the corresponding $Cu(BINAP)_2OTf$ and $[Cu(BINAP)(OTf)]_2$ species, respectively, based on related complexes described in the literature (Figure S5c).[16] Importantly, we observed that the formation of the dimeric species **3** is favored compared to the presence of the $Cu(BINAP)_2^+$ **2** species. On the contrary, when $[Cu(OTf)]_2$·toluene and XantPhos ([Cu]:XantPhos = 1:1) were mixed in THF-d_8 we only detected the corresponding XantPhos oxide (**4**) at 35.38 ppm[17] and the monomeric $Cu(XantPhos)^+$ species (**5**) at -19.03 ppm[18] as a broad singlet (Figure S5d).

When $[Cu(OTf)]_2$·toluene is mixed with BINAP and XantPhos ($[Cu]/L_1/L_2$ = 1:1:1, Figure S5e) in THF-d_8 we could detect free BINAP at -17.28 ppm (s), and the two phosphine oxides from both ligands (species **1** and **4**), while the only detectable copper complex was the $Cu(XantPhos)^+$ species (**5**). Contrarily, when the same experiment was performed with a slight decrease in the amount of the XantPhos ligand ($[Cu]/L_1/L_2$ = 1:1:0.5, Figure S5f) we could observed both Cu-complexes from BINAP and XantPhos (species **3** and **5**), along with partial formation of the $Cu(BINAP)_2^+$ complex **2**. Interestingly, using this copper/ligands combination, we only detected the corresponding oxide from the BINAP ligand **1**, while the XantPhos oxide **4** was not present in the media. In addition, we observed other species in the region of phosphine oxides (20-35 ppm) that could correspond to partial oxidation of the ligands.

In light of these experiments, we can conclude that in THF both complexes of Cu/BINAP and Cu/XantPhos are formed in equilibrium, in which for the BINAP ligand two speciation modes have been identified, leading to the coordinatively saturated $Cu(BINAP)_2^+$ species and the dimeric $[Cu(BINAP)OTf]_2$ complex. However, the formation of the Cu-XantPhos complex seems to be thermodynamically favored, as large amounts of free BINAP are observed. Additionally, large amounts of the corresponding phosphine oxides from the ligands are observed in all the experiments, thus suggesting that some Cu(II) impurities in the commercial Cu-source could mediate these oxidation processes.

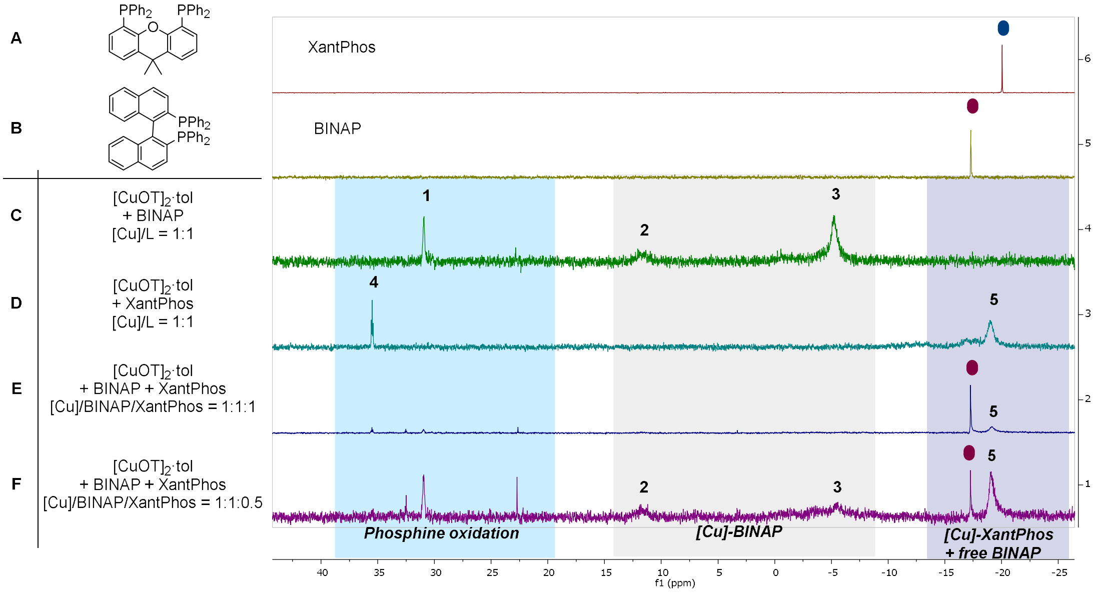

Figure S5. Analysis of Cu-speciation by ^{31}P NMR spectroscopy.

Kinetic studies.

To differentiate a tandem process from an *in situ* isomerization of the intermediate alkenyl-copper species we performed a kinetic analysis of the *anti*-hydroboration of alkynoate **1a** with B_2pin_2 under the optimized reaction conditions employing a Kessil lamp. This kinetic study was performed by taking aliquots from the reaction media at the indicated time using dicyclohexyl phthalate as an internal standard and subsequent analysis by ^1H NMR spectroscopy (Figure S6). From this study, it can be concluded that the first Cu-catalyzed B_2pin_2-addition to the alkyne is very fast since the starting material **1a** is consumed after 75 min. The main product observed from the initial stages of the reaction is the *syn*-addition isomer *Z*-**2a**, which raises its maximum value at 35 min. From the kinetic profile, it is clear that this isomer is then transformed into the *anti*-isomer *E*-**2a** until the *anti:syn* ratio reaches a constant value of 85:25 at 200 min which is maintained for several hours (not shown). From this point, the quantity of each isomer changes very slowly with time, which explains why it is important to carry out the irradiation of the reaction for 24 h. After this time, complete conversions toward the *anti*-isomer are observed.

Experimental procedure: to an oven-dried 50 mL Schlenk flask equipped with a stir bar, B_2pin_2 (380.11 mg, 1.5 mmol), $[CuOTf]_2$·tol (25.9 mg, 0.05 mmol), XantPhos (57.9 mg, 0.1 mmol), *rac*-BINAP (62.3 mg, 0.1 mmol), and dicyclohexyl phthalate (165.21 mg, 0.5 mmol) were added and the air atmosphere was replaced by a nitrogen atmosphere using a Schlenk line. Then, 70 mL of dried THF were added under N_2 atmosphere and was allowed to stir for 15 min, followed by the addition of methyl 2-hexynoate (0.134 mL, 1 mmol), MeOH (81 µL, 2 mmol) and NaO^{t}Bu 1M in THF (0.15 mL, 0.15 mmol). After that, the flask was exposed to blue light irradiation employing a Kessil lamp (model PR160L-440, see https://kessil.com/science/PR160L.php) at room temperature (24 ºC). For the kinetic analysis, aliquots were taken at the specified times, and were filtered off through a pad of silica gel to be analyzed by ^1H NMR spectroscopy (Table S4).

Table S4. Kinetic data for the anti-hydroboration of model substrate **1a**.

Time (min)	[**1a**] (M)	[*Z*-**2a**] (M)	[*E*-**2a**] (M)
0	0,014	0	0
16,06	0,00458	0,00864	7,8525E-4

31,45	0,0024	0,00968	0,00192
47	0,00161	0,0096	0,00279
61	7,38E-4	0,0093	0,00445
76	0	0,00899	0,00619
91	0	0,00864	0,00698
107	0	0,00724	0,0082
121	0	0,00619	0,00925
210	0	0,00232	0,01313
240	0	0,00226	0,01319

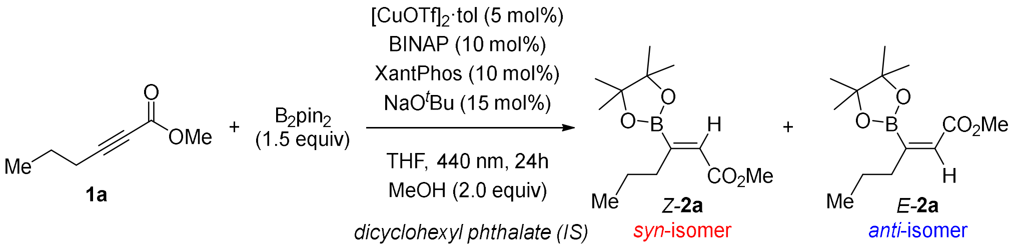

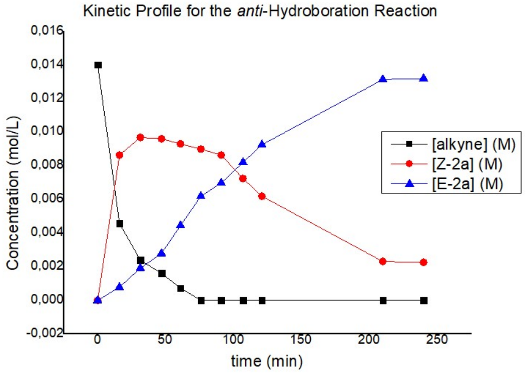

Figure S6. Kinetic analysis.

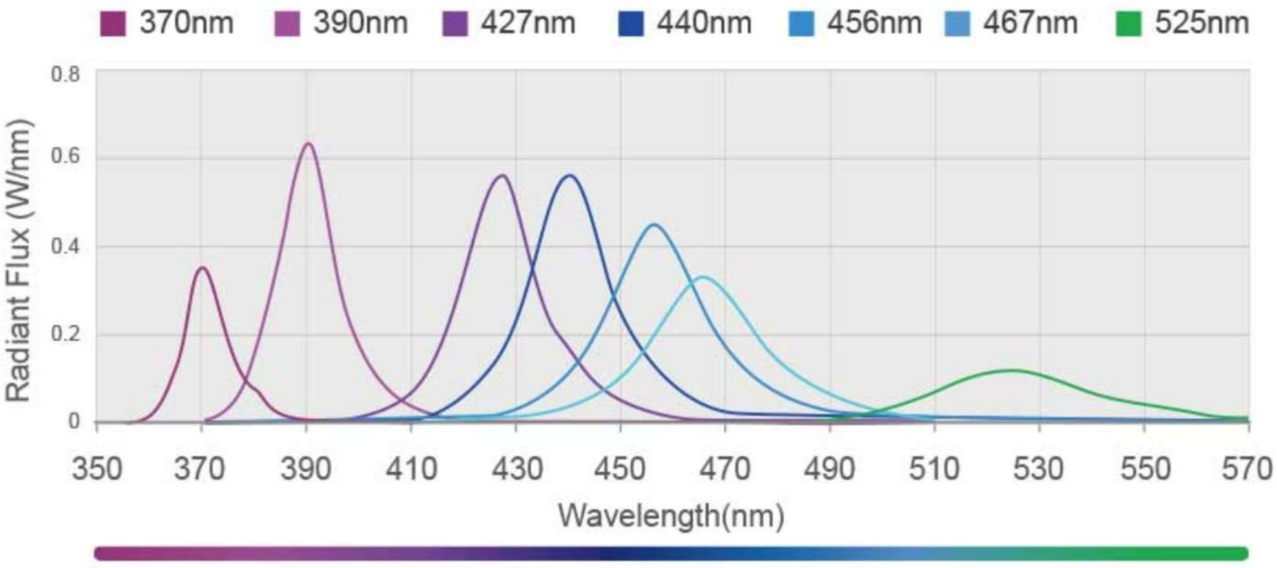

Figure S7. Emission spectra of commercial Kessil lamps. Model PR160L-440 (440 nm) was employed for this study (source: https://kessil.com/science/PR160L.php)

Effects of Lewis acids in the reaction

To understand the photoisomerization step during the tandem process, we carried out a series of control experiments to determine their impact on the reaction (Table S5). One important issue for this transformation pertains to the role of the NaO^{t}Bu, which is a fundamental additive for the generation of the catalytically active copper species. For this reason, we carried out the photoisomerization of alkenyl boronate Z-**2a** in presence of 10 mol% of the $[CuOTf]_2$·toluene/BINAP mixture and 15 mol% NaO^{t}Bu to ensure the quantitative

formation of the copper-alkoxide (entry 2). Interestingly, the photoisomerization was completely inhibited, as E-**2a** was not present in the reaction crude after irradiation for 24h. The same results were obtained upon the addition of NaOMe (entry 3). We then tried the photoisomerization in presence of the photoactive Cu/BINAP mixture in presence of 15 mol% of NaO^{t}Bu and 15 mol % of XantPhos, but the isomerization did not take place (entry 4). At this point, we hypothesized that the copper-alkoxide species coordinated with the BINAP ligand are not competent catalytic species for the photoisomerization step. Therefore, we studied the addition of different Lewis acids that would be likely involved in the reaction. Firstly, we added to the photoactive $[CuOTf]_2$·toluene/BINAP mixture with NaO^{t}Bu (15 mol%) a 30 mol% of B_2pin_2 (entry 5). In this case, we observed a 10:90 mixture of anti and syn isomers after 24h of irradiation, along with 15% of hydroboration product. This result encouraged us to propose the key role of Lewis acids in the formation of the required photoactive species for the isomerization step, although the results are not enough to explain the observed reactivity in the tandem process.

Another potential Lewis acid present in the media refers to the borate ($B(OR)_3$) formed upon σ-bond metathesis between the copper-alkoxide species and the diboron reagent. In this case when the experiment was performed in presence of 30 mol% of a commercially available pinBO^{i}Pr we observed an anti:syn ratio of 20:80 (entry 6), which was further increased to 45:55 when 50 mol% of pinBO^{i}Pr was added (entry 7). Unfortunately, when an excess of the pinBO^{i}Pr borate was added at the outset of the reaction (1.5 equivalents) we observed the precipitation of a black solid in the reaction media, which likely corresponds to the decomposition of the copper complex (entry 8). Interestingly, when 30 mol% of the more acidic BF_3·OEt was used, complete photoisomerization towards the E-**2a** isomer was observed (entry 9). These experiments support the hypothesis that the copper alkoxide coordinated with BINAP is likely formed upon the addition of NaO^{t}Bu, forming a complex which is not prone to photosensitization of the alkenyl boronate. In presence of a Lewis acid, this complex could undergo alkoxide-abstraction via an acid-base reaction to generate the cationic BINAP/Cu species in which the corresponding boron-"ate" anion would act as the counter-ion. This species is expected to exhibit greater abilities for interaction with the alkenyl boronate, thereby promoting the photoisomerization step. Finally, we compared the efficiency of $Cu(OTf)_2$ in this reaction. Although the isomerization was not inhibited, the efficiency was lower in comparison with the $[CuOTf]_2$·toluene complex (anti:syn = 74:26 vs. >98:2, respectively), indicating that the active species is likely a Cu(I) complex instead of a Cu(II) species. In this case, is well-known the ability of copper(II) to oxidize the BINAP ligand, yielding corresponding Cu(I)/BINAP complex.[19] This side-reaction could be ensuring the formation of a photoactive Cu(I)-based photocatalyst that accounts for the isomerization observed in this last experiment.

Table S5. Effect of Lewis acids in the photoisomerization reaction in presence of base.

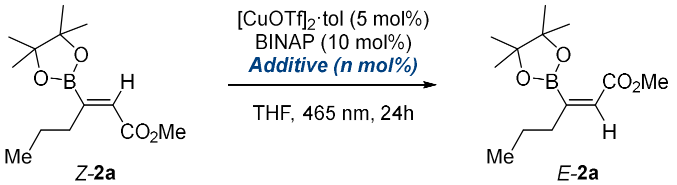

Z-**2a** *syn*-isomer — *E*-**2a** *anti*-isomer

Entry	NaO^{t}Bu	additive (n mol%)	*anti:syn (%)*[a]
1	-	-	>98:2
2	15 mol%	-	<2:98
3	NaOMe (15 mol%)	-	<2:98
4	15 mol%	XantPhos (10 mol%)	<2:98
5	15 mol%	B_2pin_2 (30 mol%)	10:90 (+ 15% HB)
6	15 mol%	pinBO^{i}Pr (30 mol%)	20:80
7	15 mol%	pinBO^{i}Pr (50 mol%)	45:55
8	15 mol%	pinBO^{i}Pr (150 mol%)	Decomposition Cu-cat
9	15 mol%	$BF_3·Et_2O$ (30 mol%)	>98:2
10	-	$Cu(OTf)_2$ (10 mol%)	74:26

[a]Determined in the reaction crude by ^{1}H NMR spectroscopy employing 1,3,5-trimethoxybenzene (TMB) as an internal standard.

Further analysis of the reaction mixture employing the pinBO^{i}Pr species and the potential copper-alkoxide species were studied by ^{11}B NMR spectroscopy to determine whether the formation of boron-"ate" species would be feasible. A mixture of $[CuOTf]_2$·toluene and BINAP ([Cu]/ligand 1:1) were dissolved in THF at room temperature and after 1 h 0.90 equivalents of NaO^{t}Bu were added, observing a change-color from yellowish to dark brown. The resulting solution was further kept at room temperature for 1 h to ensure the formation of the copper-alkoxide. After that, 1.05 equivalents of pinBO^{i}Pr were added to this solution and the resulting mixture was transferred to a J-Young NMR tube and was analyzed by ^{11}B NMR spectroscopy (Figure S8). In the reaction mixture we could detect a signal at 22 ppm as the major species, which was assigned to the free pinBO^{i}Pr by comparison with a pure sample of this compound. Additionally, we observed the formation of two signals at 7.85, and 3.99, which fall in the range of boron-"ate" species. The more shielded peak at 3.99 ppm was assigned to the pinB(O^{i}Pr)(O^{t}Bu) complex since the same signal is observed when pinBO^{i}Pr is treated with an equimolar amount of NaO^{t}Bu (^{11}B NMR = δ 3.99 ppm).

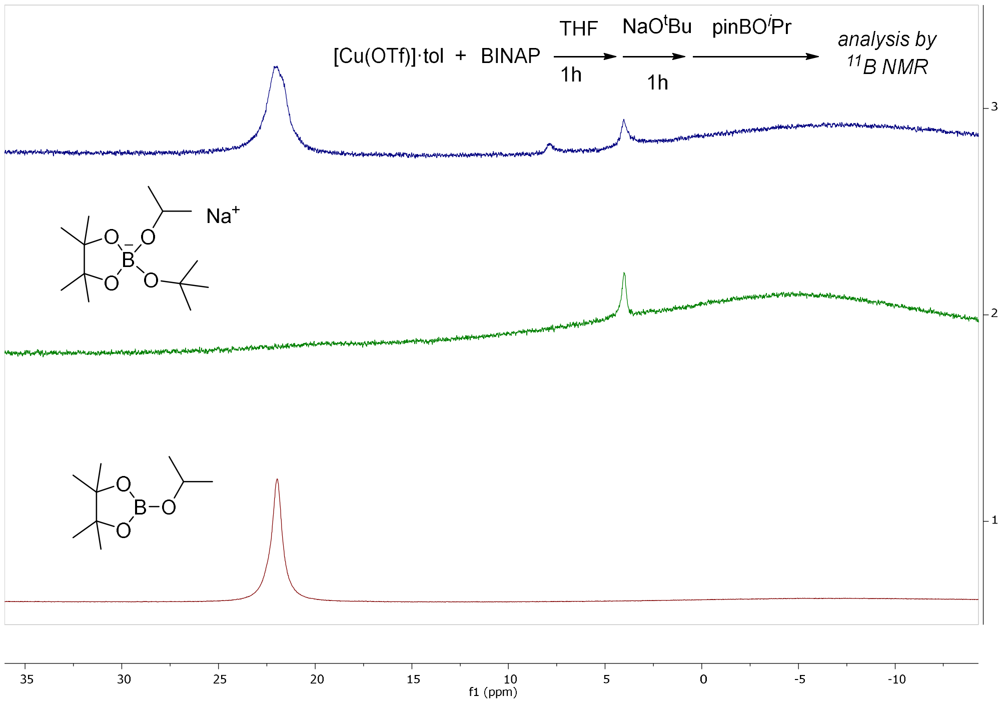

Figure S8. Analysis of the [Cu/BINAP + base] system in presence of pinBO^{i}Pr by ^{11}B NMR spectroscopy.

The signal at 7.85 ppm was tentatively assigned to a potential species of the type [(BINAP)Cu][B(O^{i}Pr)(O^{t}Bu)(pin)] in which an oxygen atom of one of the alkoxide groups would be interacting with both the copper and boron centers by μ-type coordination, acting as a bridged ligand (Figure S9). This complex is expected to display a more deshielded signal in the ^{11}B NMR spectrum than the [pinB(O^{i}Pr)(O^{t}Bu)]$^-$ anion. In this regard, related complexes in which boron-“ate” species interact with copper(I) have been described in the literature, such as the isolation and characterization of NHC-Cu(I) with alkoxy-boron-“ate” anions, as described in 2012 by Hou[20] and recently by Popp and co-workers[21] from boracarboxylation of alkynes and alkenes, respectively (Figure S9a and b). In these complexes, the interaction between the boron-“ate” intermediate and the copper atom occurs through the oxygen from the pinacol unit. The ^{11}B NMR signal for these species is 7.5 and 13.9 ppm for the Hou’s and Popp’s complexes, respectively. Of importance is the remarkable similarity between the chemical shift displayed by the Hou’s complex which is in closed resemblance with the signal observed in our experiments at 7.85 ppm. Other complexes have been also described, for instance Westcott reported the utilization of copper(I) arylspiroborates for cyclizations of alkynoic acids (Figure S9d)[22] The corresponding copper complexes were analyzed by X-Ray diffraction, showing no O—Cu bonds and chemical shifts about 13 ppm by ^{11}B NMR spectroscopy.

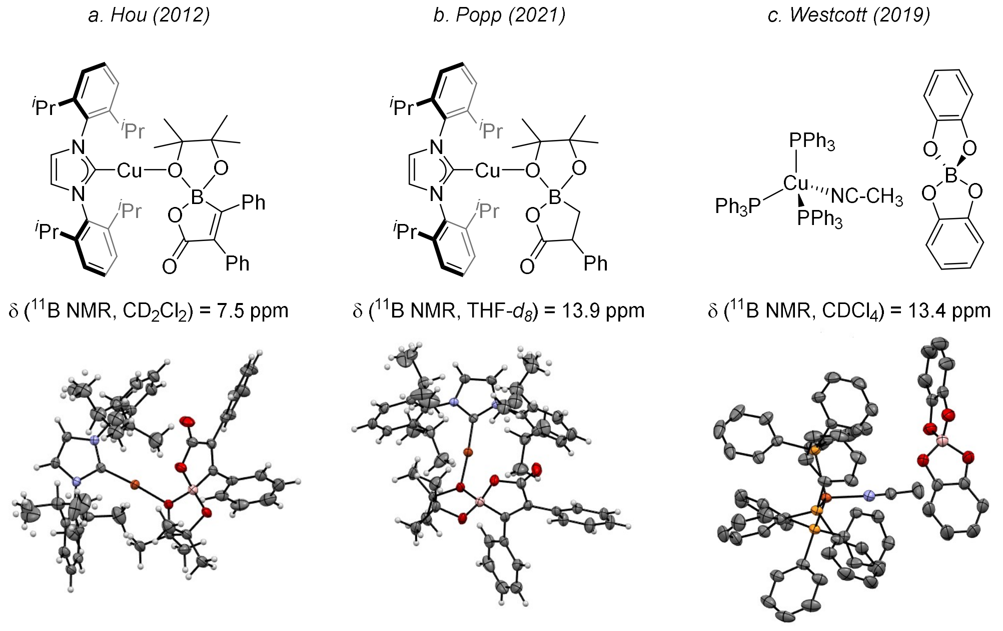

Figure S9. Boron-'ate' anions in copper complexes.

Comparison with Thioxanthone sensitizer: attempts to develop Cu/Thioxanthone Tandem Catalysis

To test whether comparable results can be obtained if a tandem Cu-catalyzed syn-hydroboration can operate in tandem with a thioxanthone-catalyzed photoisomerization with similar efficiency we carried out a series of experiments upon replacement of the Cu/BINAP system by thioxanthone. Initially, we employed reaction conditions reported by Gilmour and co-workers in their Science paper for the isomerization of boryl acrylates (see ref. 19 in the main text), which requires the use of thioxanthone in CH_3CN as solvent under purple irradiation. Therefore, we use of these conditions in combination with model substrate **1a**, B_2pin_2 (1.5 equiv) and the catalytic system for promoting the borylation reaction ($[CuOTf]_2$·tol (5 mol%), XantPhos (10 mol%), NaO^{t}Bu (15 mol%), MeOH). To our surprise, we did not observe any borylation product either under purple or blue light irradiation, likely resulting from the incompatibility of the base in CH_3CN, inhibiting the borylation reaction.

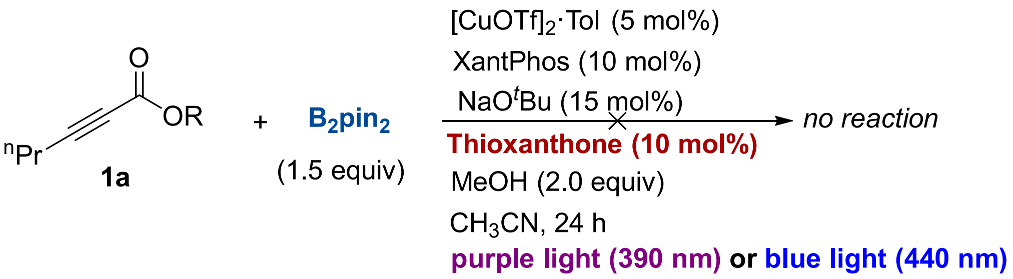

As a second approach, we subjected three representative substrates from Scheme 2 (see the main text) to the standard reaction conditions replacing the BINAP ligand with thioxanthone sensitizer (10 mol%) employing our conditions, that require the use of THF as solvent. The results are summarized in the Scheme below. Under blue-LED light, very poor efficiency was observed in the photoisomerization step, producing the hydroboration product in moderate to good yields but with poor isomerization (anti/syn ratios ranging from 23:77 to 3:97). This result was not unexpected because the maximum wavelength absorption of thioxanthone (380 nm in MeCN, JACS. 2020, 142, 14947) does not match with the wavelength of blue-LED (465 nm). Indeed, in line with this observation, the isomerization of of β-borylacrylic acid derivatives via selective energy-transfer using thioxanthone as a photocatalyst was reported by Gilmour to proceed under more energetic purple light. With this knowledge in hand, when the same set of experiments were performed using purple-LED light (390 nm) under otherwise identical reaction conditions, complex reaction mixtures were observed showing mainly decomposition products. Given that Gilmour has demonstrated in the above-mentioned report that even complex alkenylboronic esters derived from cholesterol can survive for 16 h under thioxanthone/purple light conditions, these results suggest that the active Cu-complex is not stable when exposed to the purple-LED (390 nm). Exposure to shorter reaction times under thioxanthone/purple light conditions the reaction led to poor conversion (31% upon 2 hours) or mainly decomposition (higher than 5 hours). These new experiments provide compelling evidence of the importance of this one-metal/two ligand combination not only in productive catalysis, thereby showcasing its utility for solving a relevant synthetic problem, but also as a potential general solution to replace thioxanthone/purple light combination in the design of tandem multicatalytic approaches where some metal complex involved could be sensitive to said conditions.

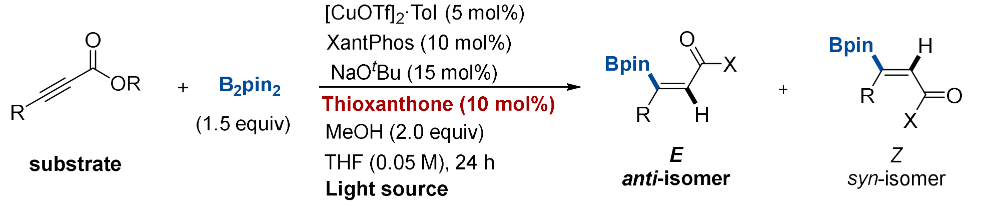

substrate	ligth source	yield[a]	anti/syn[b]	[Result in Scheme 2]
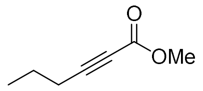	blue LEDs (465 nm)<br>purple LEDs (390 nm)	70%<br>13%[c]	7:93<br>50:50	**78%,**<br>***anti/syn* = >98:2**
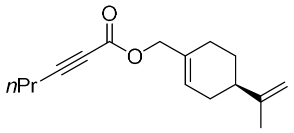	blue LEDs (465 nm)<br>purple LEDs (390 nm)	58%<br>decomp.	84:16<br>–	**78%,**<br>***anti/syn* = >98:2**
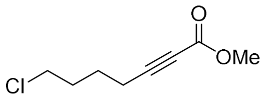	blue LEDs (465 nm)<br>purple LEDs (390 nm)	67%<br>21%[c]	23:77<br>>98:2	**71%,**<br>***anti/syn* = >98:2**

[a] Determined by ^{1}H NMR from the crude mixture (1,3,5-trimethoxy-benzene was used as internal standard).
[b] Determined by ^{1}H NMR from the crude mixture. [c] Mainly decomposition products were detected

Determination of the stereochemistry

To determine the configuration of the alkenyl boronates, we performed a series of spectroscopic experiments based on the nuclear Overhausser effect (nOe) for a selected family of hydroborylated products. The alkenyl boronate Z-**2a** was irradiated at the olefinic proton and two main interactions with the pinacol unit of the -Bpin fragment and the methyl group of the ester were observed with values of 0.12% and 0.22%, respectively (Figure S10). This experiment was further compared with the analysis of the isomer E-**2a**, where these interactions were not observed. In this case, when the olefinic proton was irradiated a nOe effect of 0.85% was observed with the allylic proton of the n-propyl chain (Figure S11). These interactions are in accordance with the assigned stereochemistry.

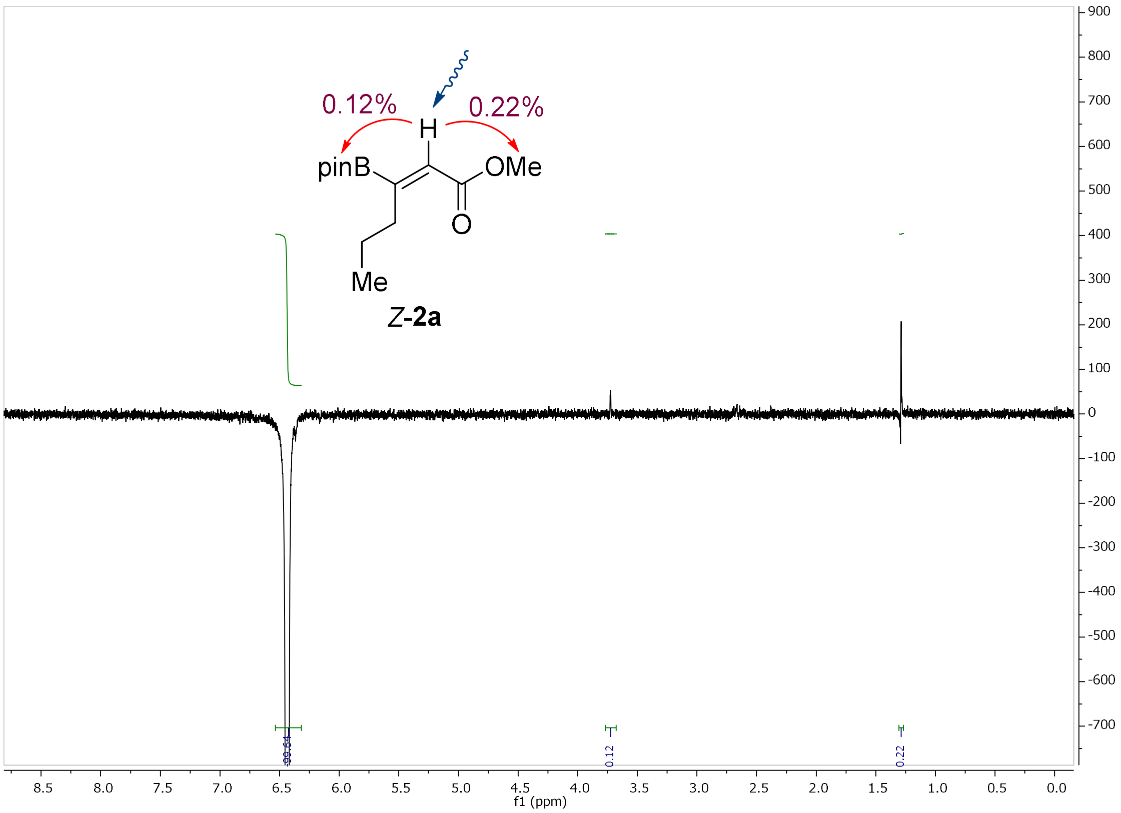

Figure S10. Key interactions by nOe effects for alkenyl boronate *Z*-**2a***.*

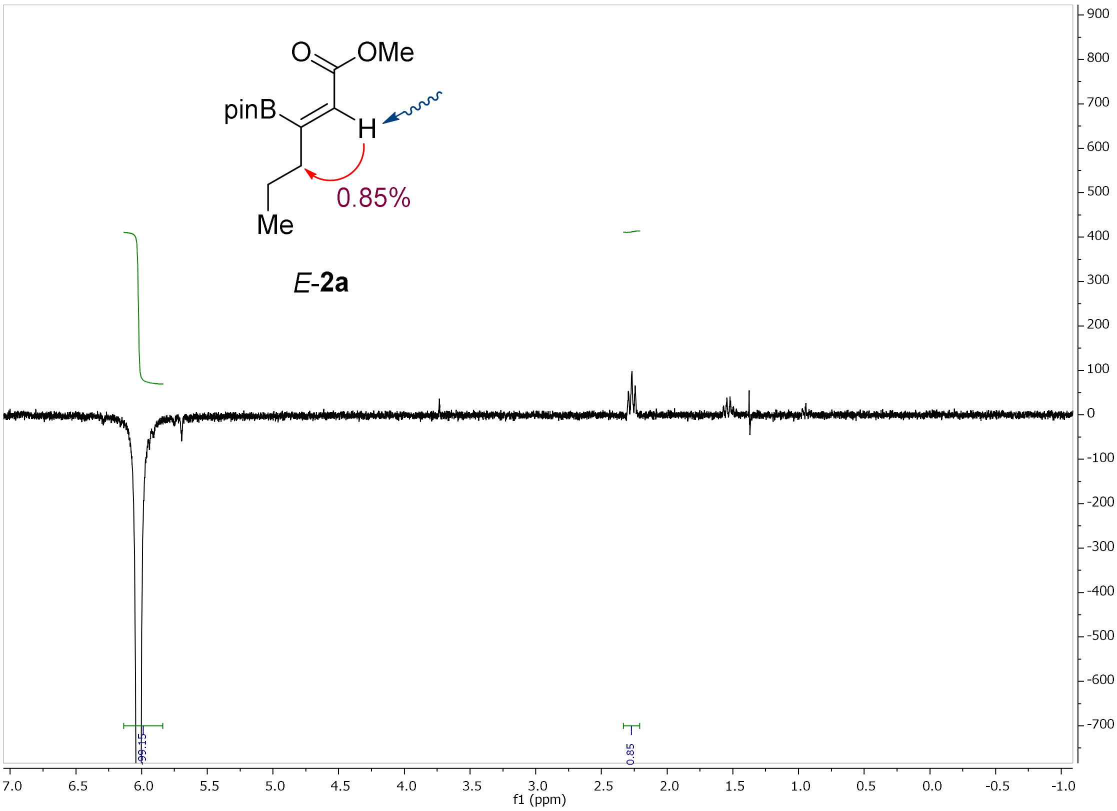

Figure S11. Key interactions by nOe effects for alkenyl boronate *E*-**2a***.*

We then studied the nOe effect for other substrates to further corroborate the postulated anti-stereochemistry. For substrate E-**2d** a 2.22% value was obtained from the nOe experiment between the olefinic and allylic protons when the former was irradiated (Figure S12). In the case of alkenyl boronate E-**2f**, a strong nOe between the olefinic proton and the allylic hydrogen was obtained with a value of 1.71% (Figure S13). In the case of the amide series, we studied the same interaction in substrates E-**2m** and E-**2q** obtaining values of 0.74% and 1.47%, respectively (Figures S14 and S15). For this latter compound, additional interactions between the olefinic proton with the N-H and the methyl ester groups were observed with values 1.77% and 0.18%, respectively.

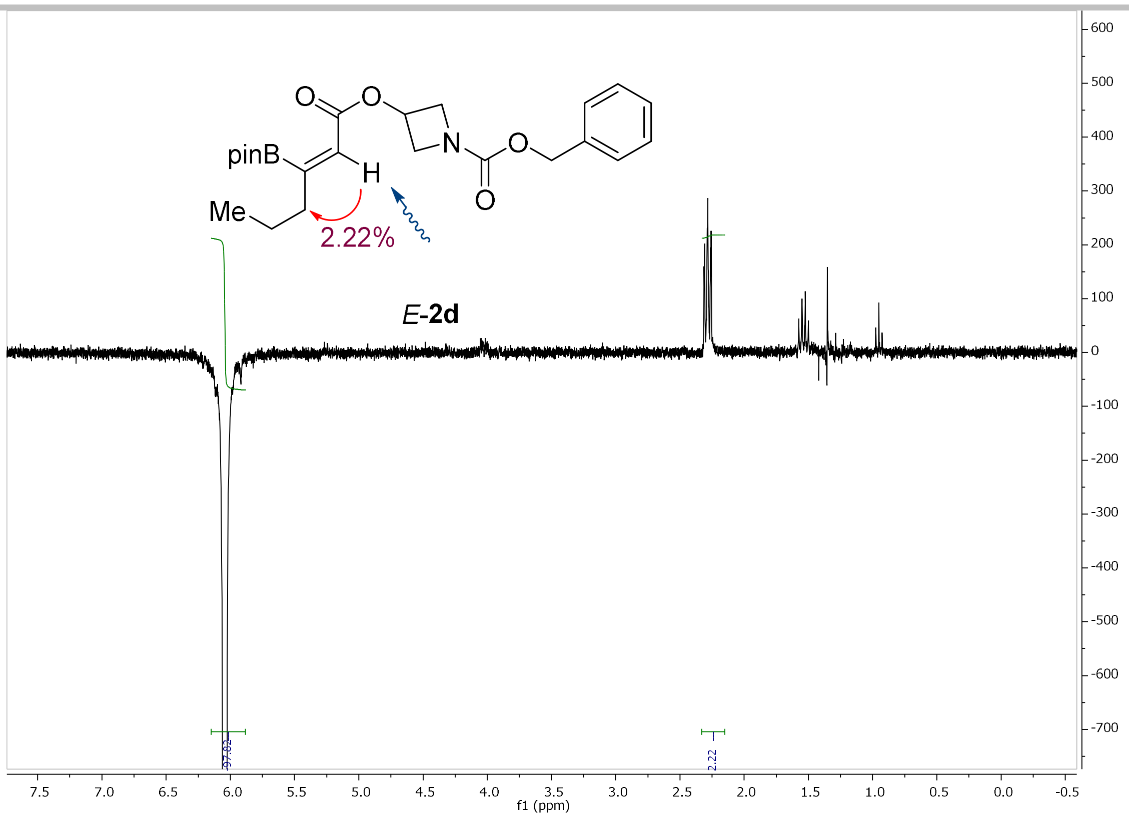

Figure S12. Key interactions by nOe effects for alkenyl boronate *E*-**2e.**

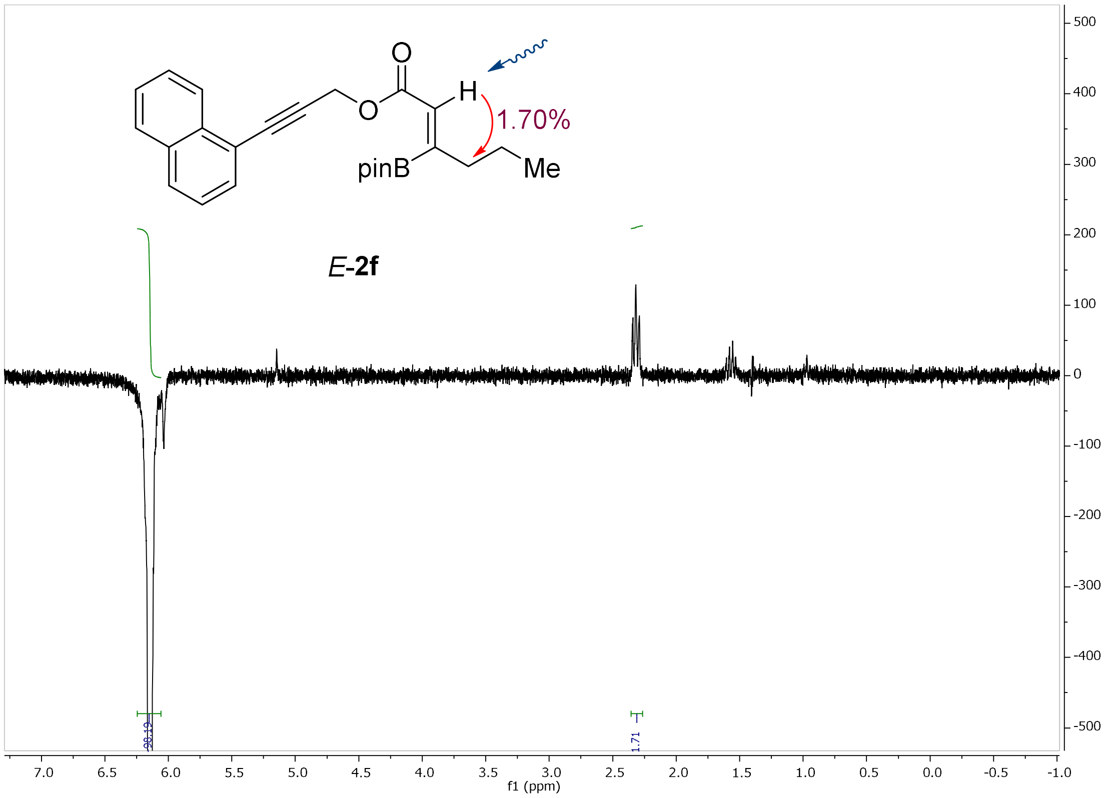

Figure S13. Key interactions by nOe effects for alkenyl boronate *E*-**2f.**

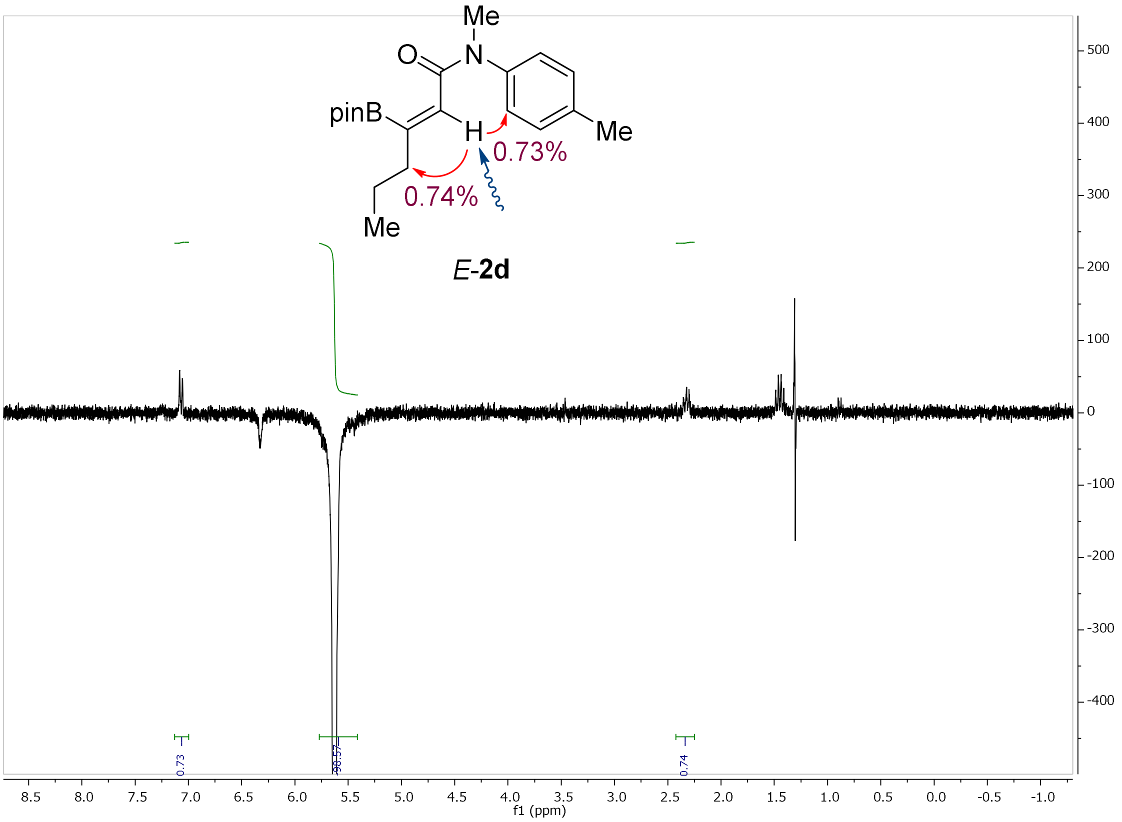

Figure S14. Key interactions by nOe effects for alkenyl boronate *E*-**2d.**

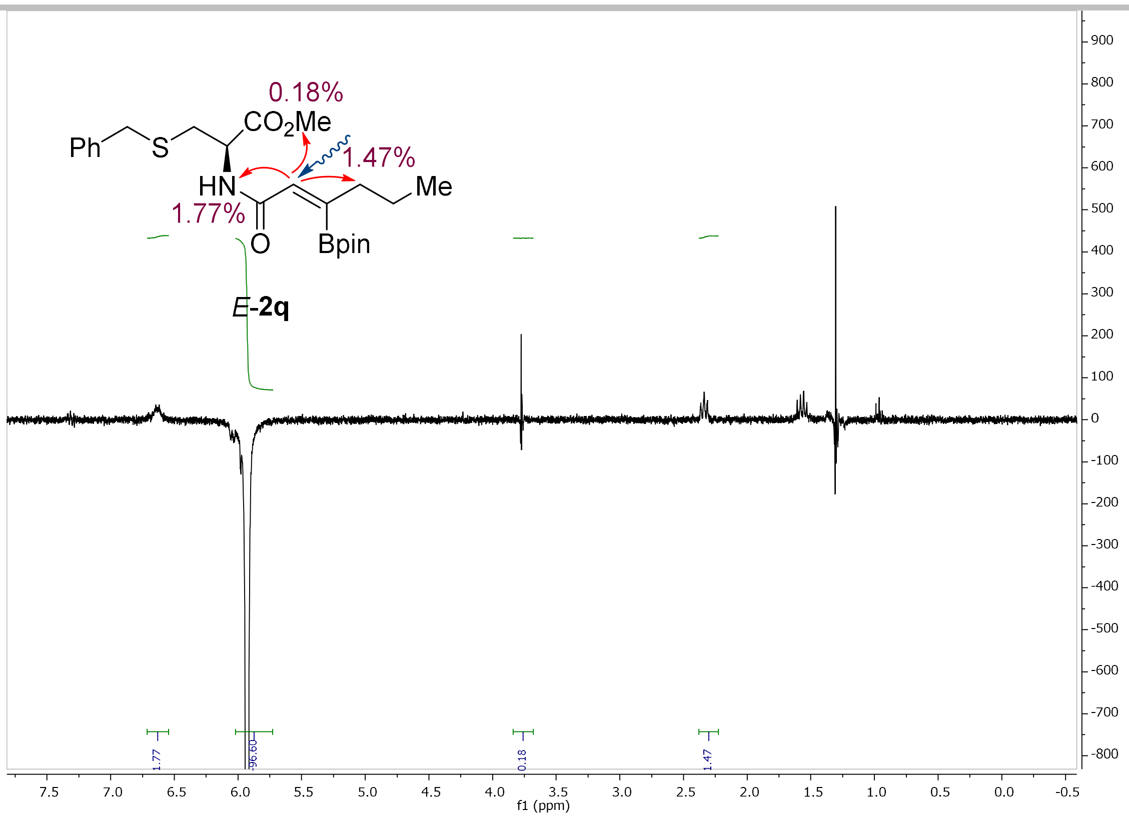

Figure S15. Key interactions by nOe effects for alkenyl boronate *E*-**2q.**

Stereochemical assignment by X-ray diffraction

Suitable crystals from product *E*-**2n** for X-ray diffraction by slow difussion of n-pentane into a solution of the product in CH_2Cl_2 at 0 ºC. CCDC 2158474 contains the supplementary crystallographic data. These data can be obtained free of charge from the Cambridge Crystallographic Data Center via https://www.ccdc.cam.ac.uk

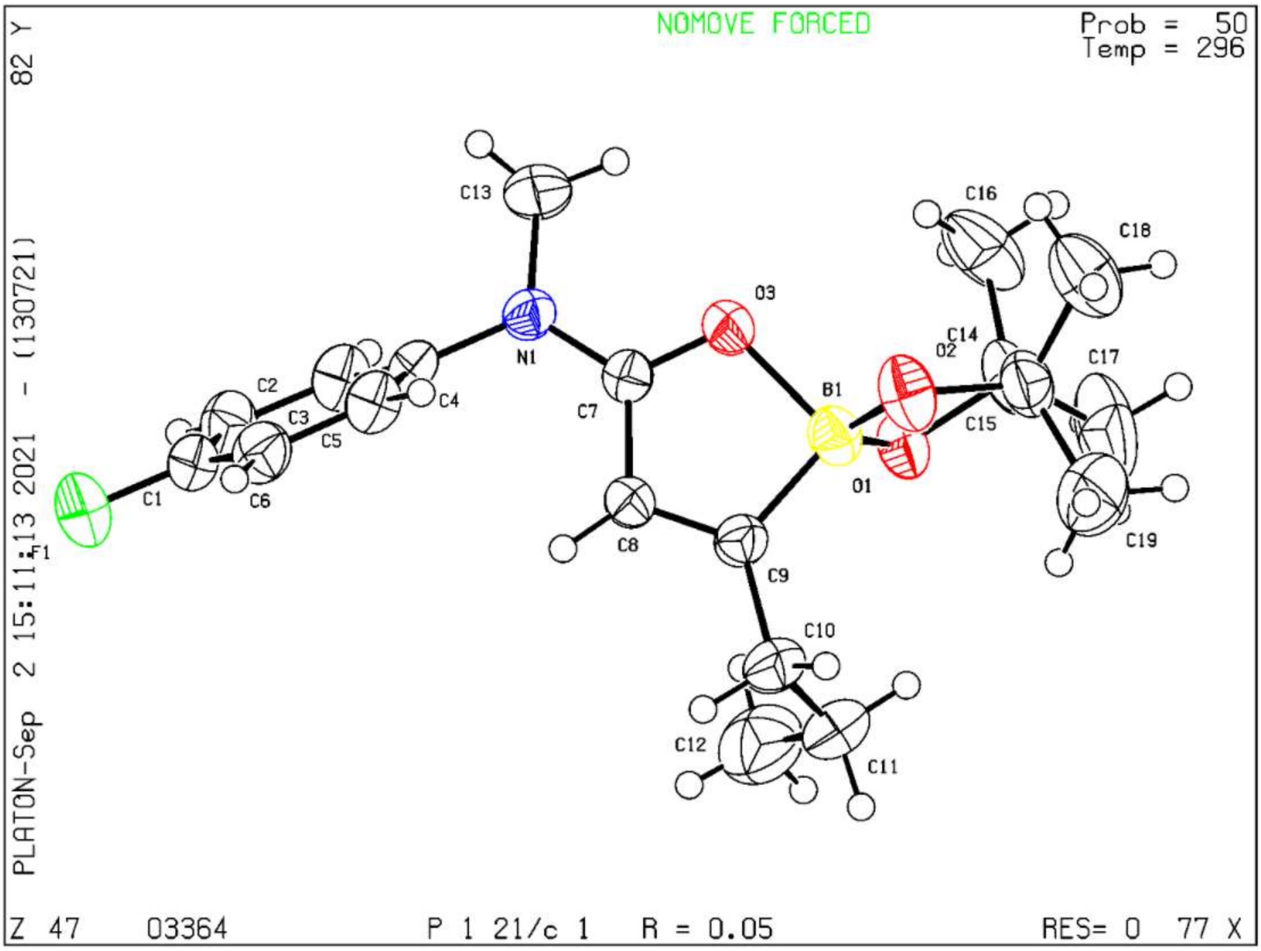

Table S6. Sample and crystal data

**Chemical formula**	$C_{19}H_{27}BFNO_3$
**Formula weight**	347.22 g/mol
**Temperature**	296(2) K
**Wavelength**	0.71073 Å
**Crystal size**	0.256 x 0.456 x 0.523 mm
**Crystal habit**	clear colourless prismatic
**Crystal system**	monoclinic
**Space group**	P 1 21/c 1
**Unit cell dimensions**	a = 10.105(3) Å; α = 90°<br>b = 10.914(3) Å; β = 94.921(12)°<br>c = 17.886(5) Å; γ = 90°
**Volume**	1965.3(11) Å^3
**Z**	4
**Density (calculated)**	1.173 g/cm^3

**Absorption coefficient**	0.084 mm^{-1}
**F(000)**	744

Table S7. Data collection and structure refinement

**Theta range for data collection**	2.29 to 25.35°	
**Index ranges**	-12<=h<=12, -13<=k<=13, -21<=l<=20	
**Reflections collected**	55516	
**Independent reflections**	3581 [R(int) = 0.0316]	
**Coverage of independent reflections**	99.9%	
**Absorption correction**	Multi-Scan	
**Max. and min. transmission**	0.9800 and 0.9300	
**Structure solution technique**	direct methods	
**Structure solution program**	XT, VERSION 2018/2	
**Refinement method**	Full-matrix least-squares on F^2	
**Refinement program**	SHELXL-2018/3 (Sheldrick, 2018)	
**Function minimized**	$\Sigma\ w(F_o^2 - F_c^2)^2$	
**Data / restraints / parameters**	3581 / 0 / 233	
**Goodness-of-fit on F^2**	1.015	
**Final R indices**	3180 data; $I>2\sigma(I)$	R1 = 0.0454, wR2 = 0.1290
	all data	R1 = 0.0502, wR2 = 0.1349
**Weighting scheme**	$w=1/[\sigma^2(F_o^2)+(0.0735P)^2+0.6894P]$ where $P=(F_o^2+2F_c^2)/3$	
**Absolute structure parameter**	0.00(11)	
**Extinction coefficient**	0.0300(30)	
**Largest diff. peak and hole**	0.319 and -0.201 eÅ^{-3}	
**R.M.S. deviation from mean**	0.043 eÅ^{-3}	

Photochemical studies

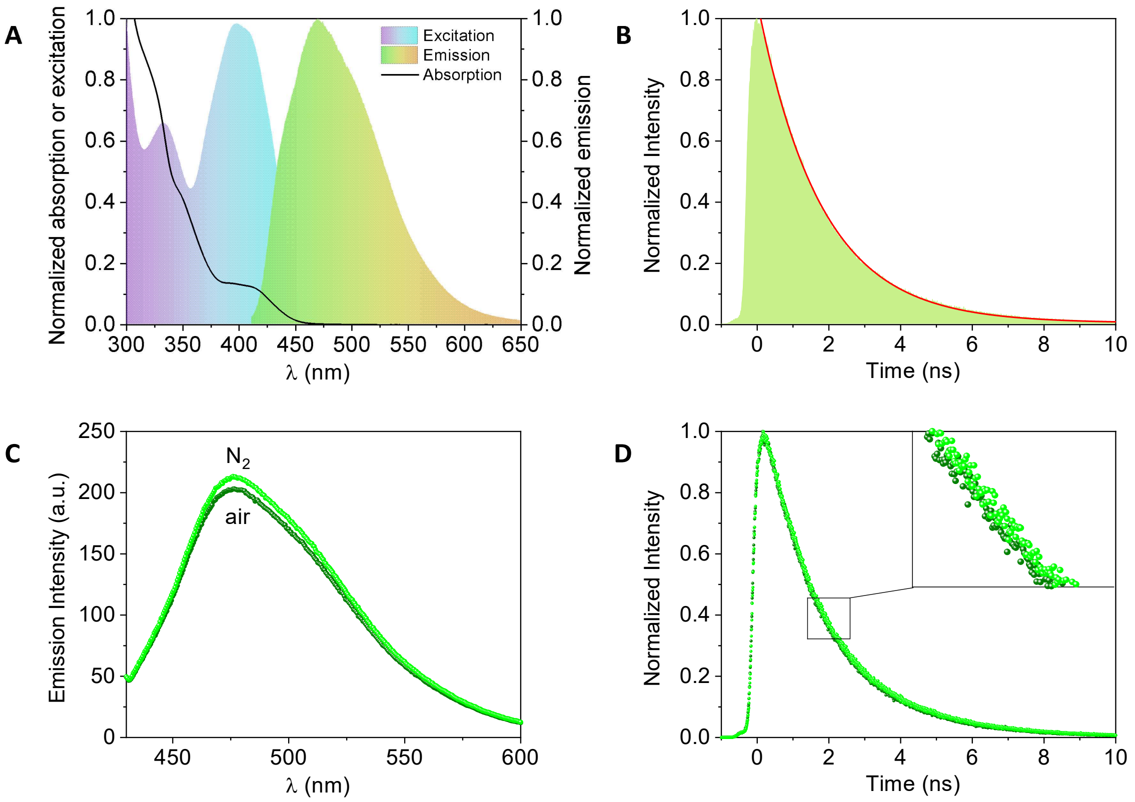

Figure S16. a) Normalized absorption (black line), excitation (ultraviolet-blue) and fluorescence (λ_{exc}= 400 nm, green) spectra for $[CuOTf]_2$·tol/BINAP (20 μM) in acetonitrile. b) Normalized fluorescence decay trace (λ_{exc} = 372 nm), with a band pass filter centered at 450 nm for $[CuOTf]_2$·tol/BINAP (20 μM) in acetonitrile. c and d) Emission (c) and time-resolved fluorescence (d) of $[CuOTf]_2$·tol/BINAP (20 μM) in aerated (green olive) or deaerated (green) acetonitrile. Inset: zoom image.

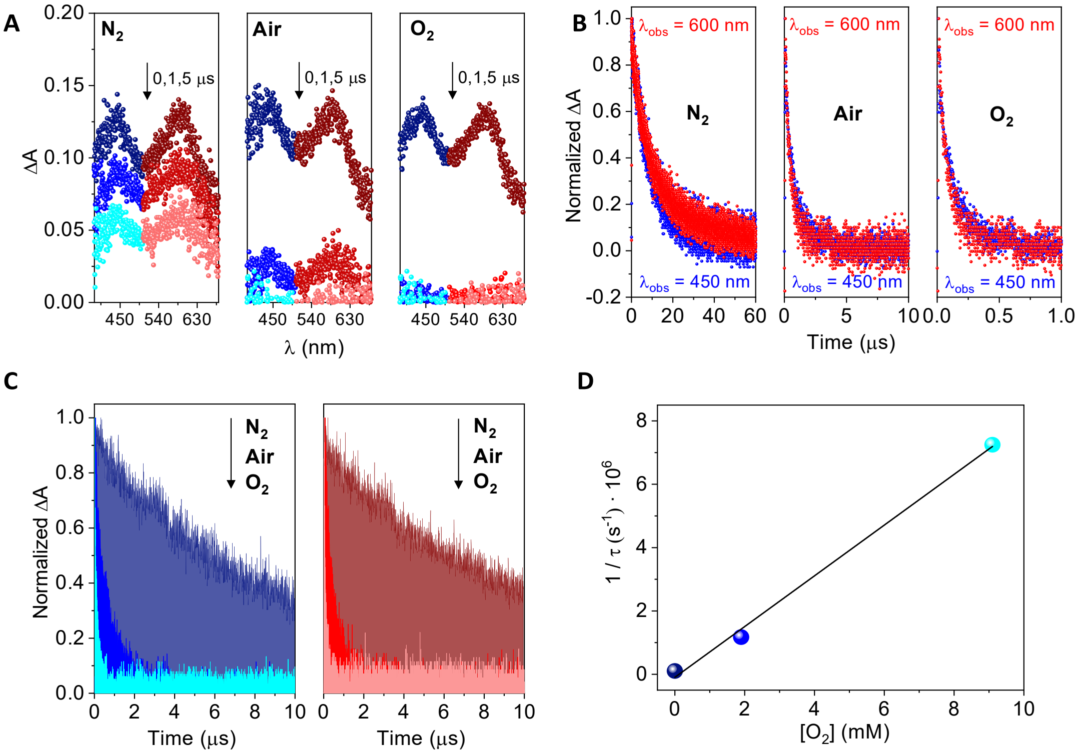

Figure S17. a) Transient absorption spectra (λ_{exc} = 355 nm) for $[CuOTf]_2$·tol/BINAP (20 µM) in acetonitrile at different timescales (0, 1 and 5 µs) after laser pulse in aerated and purged (by N_2 or O_2) atmosphere. b) Normalized transient decay traces at λ_{exc} = 355 nm monitoring at λ_{mon} = 450 (blue) or 650 (red) nm for Cu-BINAP (20 µM) in aerated and purged (by N_2 or O_2) atmosphere. c) Transient decay traces comparison (λ_{exc} = 355 nm) for $[CuOTf]_2$·tol/BINAP (20 µM) at different atmosphere (N_2, aerated or O_2) after monitoring at λ_{mon} = 450 (blue, left) or 650 (red, right) nm. D) Stern-Volmer plot.

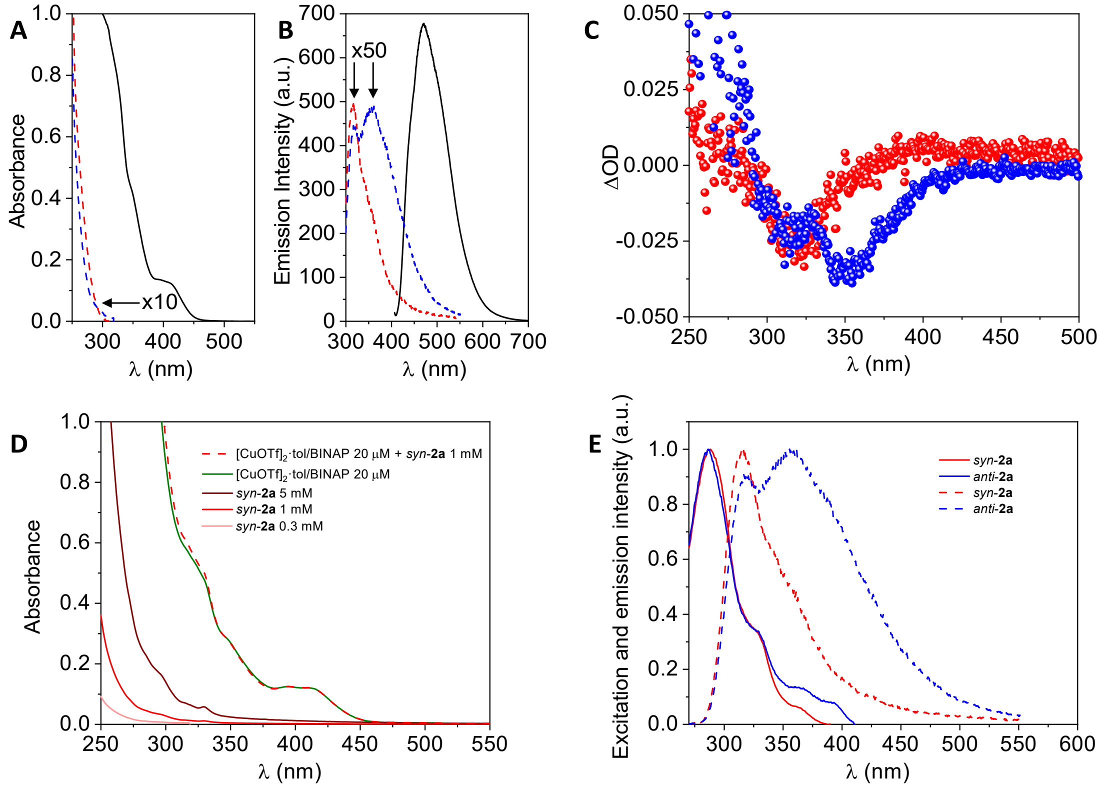

Figure S18. a) Absorption spectra (x10) of *syn*-**2a** (red) and *anti*-**2a** (blue) 300 mM isomer. $[CuOTf]_2$·tol/BINAP (20 mM, black) is included for comparison. b) Fluorescence spectra (x50, λ_{exc} = 280 nm) of *syn*-**2a** (red) and *anti*-**2a** (blue) 300 mM isomer. $[CuOTf]_2$·tol/BINAP (20 mM, black) is included for comparison. c) Transient absorption spectra (λ_{exc} = 280 nm) of *syn*- **2a** (red) and *anti*-**2a** (blue) 1 mM isomer. d) Absorption spectra of *syn*-**2a** isomer at different concentrations up to 5

mM. [CuOTf]$_2$·tol/BINAP (20 mM, green) in absence or presence of *syn*-**2a** 1 mM is included for comparison. e) Normalized excitation (solid line) and fluorescence (dot line) spectra for *syn*-**2a** (red) and *anti*-**2a** (blue) 1 mM isomer in acetonitrile.

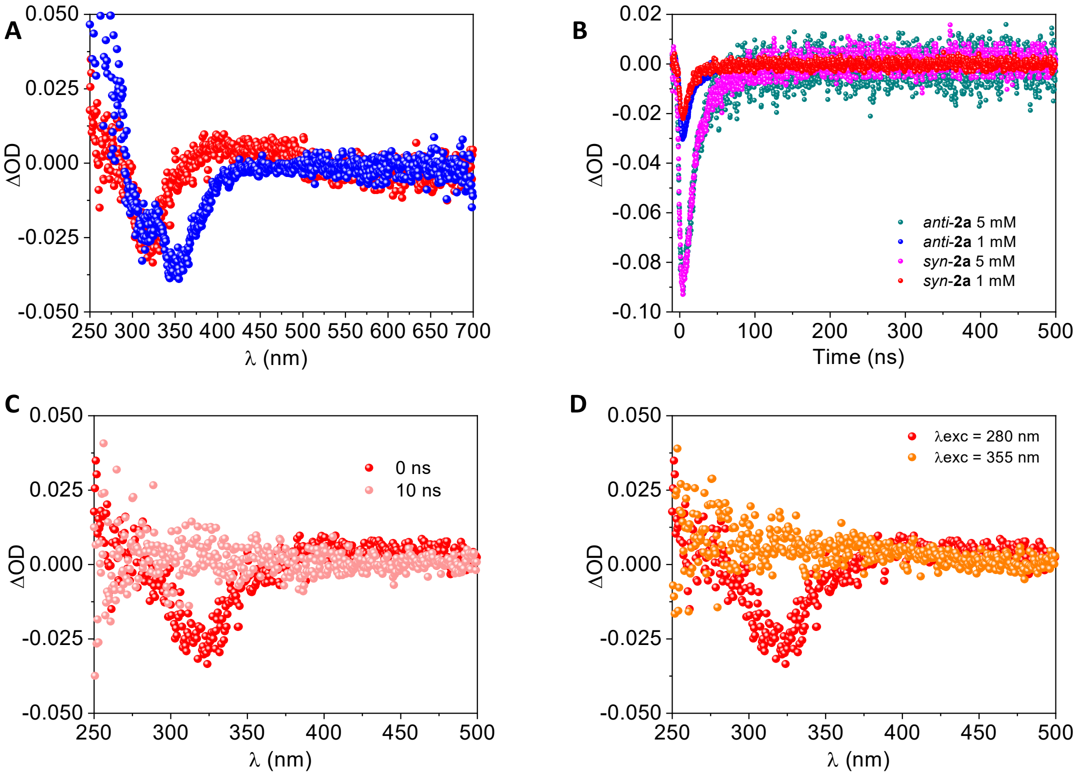

Figure S19. a) Transient absorption spectra (λ_{exc} = 280 nm) of *syn*- **2a** (red) and *anti*-**2a** (blue) 1 mM isomer. B) Decay traces (λ_{exc} = 280 nm) of *syn*-**2a** (λ_{obs} = 325 nm, red and pink) and *anti*-**2a** (λ_{obs} = 350 nm, blue and turquoise) at 1mM or 5 mM, respectively. c) Transient absorption spectra (λ_{exc} = 280 nm) of *syn*- **2a** 1 mM immediately after laser pulse (red) or after 10 ns (light red). d) Transient absorption spectra of *syn*- **2a** 1 mM at λ_{exc} = 280 nm (red) or 355 nm (orange) registered mmediately after laser pulse.

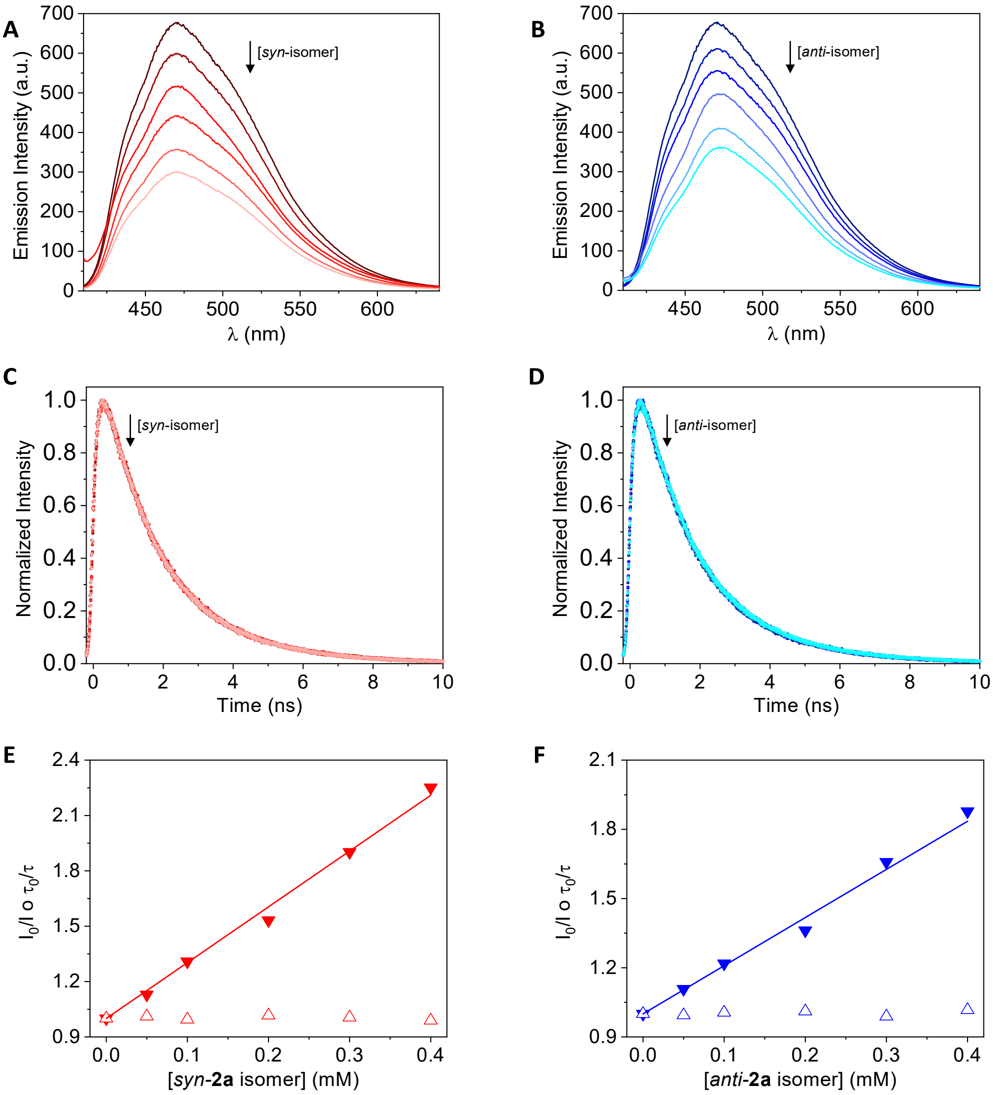

Figure S20. A) and b) Fluorescence emission (λ_{exc} = 400 nm) for $[CuOTf]_2$·tol/BINAP (20 μM) upon addition of increasing concentrations of *syn*-**2a** (red) y *anti*-**2a** (blue) isomer (up to 400 μM) in acetonitrile. c and d) Fluorescence decay traces (λ_{exc} = 445 nm, band pass filter centered at 500nm) for $[CuOTf]_2$·tol/BINAP (20 μM) upon addition of increasing concentrations of *syn*-**2a** (red) y *anti*-**2a** (blue) isomer (up to 400 μM) in acetonitrile. e and f) Stern-Volmer plots for steady-state (solid triangles) or time-resolved (empty triangles) quenching fluorescence experiments by addition of the *syn*-**2a** (e) or *anti*-**2a** (f) isomer.

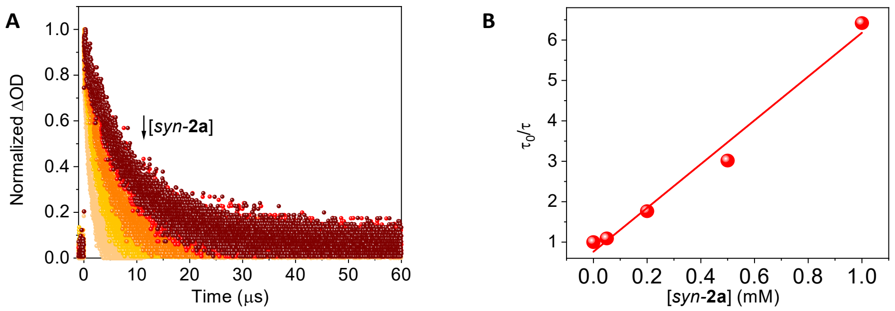

Figure S21. a) Transient decay traces (λ_{exc} = 355 nm, λ_{mon} = 450 nm) for $[CuOTf]_2$·tol/BINAP (20 mM) upon addition of increasing concentrations of *syn*-isomer (*Z*-**2a**) up to 1 mM in purged acetonitrile. B) Corresponding Stern-Volmer plot.

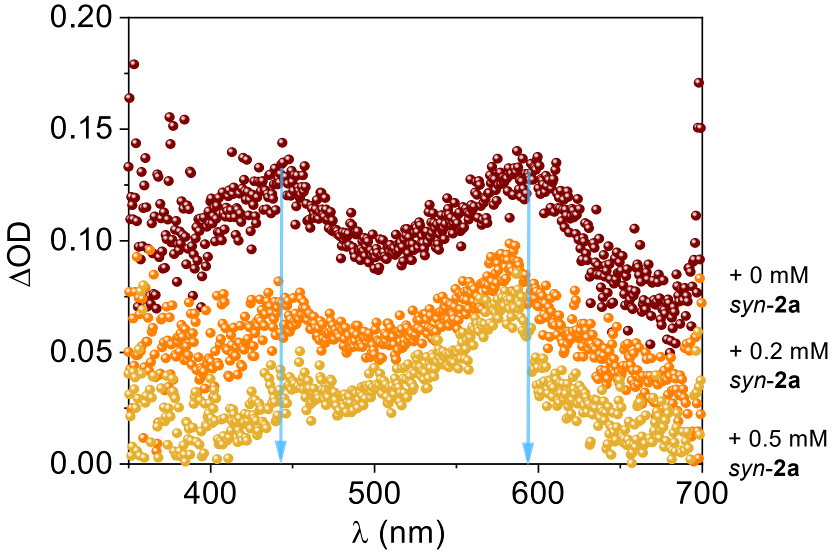

Figure S22. Transient absorption spectra (λ_{exc} = 455 nm) for $[CuOTf]_2$·tol/BINAP (60 mM) upon addition of increasing concentrations of *syn*-isomer (*Z*-**2a**) in acetonitrile monitored after 3 µs laser pulse under N_2.

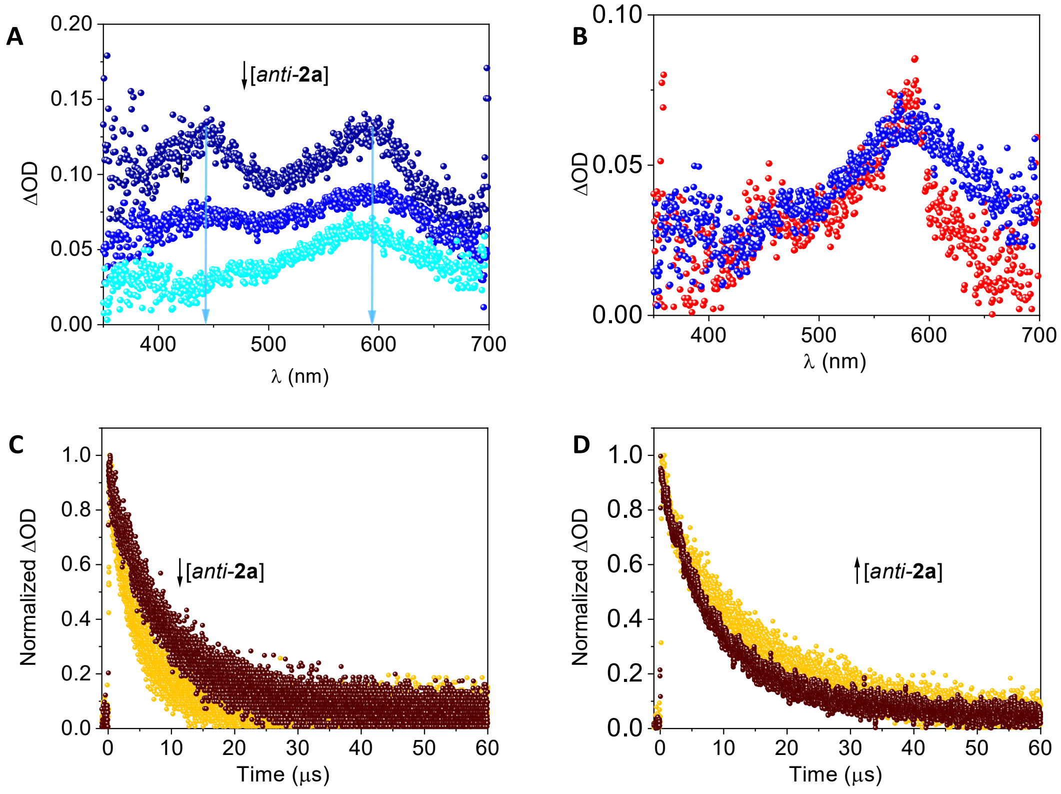

Figure S23. a) Transient absorption spectra (λ_{exc} = 355 nm) for $[CuOTf]_2$·tol/BINAP (20 µM) upon addition of increasing concentrations up to 15 mM of anti-isomer (*E*-**2a**) in acetonitrile monitored after 3 µs laser pulse under N_2. b) Transient comparative (λ_{exc} = 355 nm) for $[CuOTf]_2$·tol/BINAP (20 µM) in the presence of 5 mM of *syn*-isomer (red) or 15 mM *anti*-isomer (blue) under N_2. c and d) Decay traces comparison (λ_{exc} = 355 nm) for $[CuOTf]_2$·tol/BINAP (20 µM) in the presence of 15 mM of *anti*-isomer (*E*-**2a**) after monitoring at λ_{mon} = 450 (c) or 590 nm (d) pulse under N_2.

DFT Studies

DFT studies were performed using the B3LYP[23,24] as the functional and the 6-31G** basis set. Geometry optimizations were performed without symmetry restrictions and in all cases the solvent (acetonitrile) effect was taken into account by means of the solvation model (SMD).[25] The vertical transitions (i.e. excitation energies) have been computed at the TD-DFT level of theory. All calculations were carried out with the Gaussian 16 program package.[26] Simulation of UV-vis spectrum was performed using GaussSum 3.0.[27]

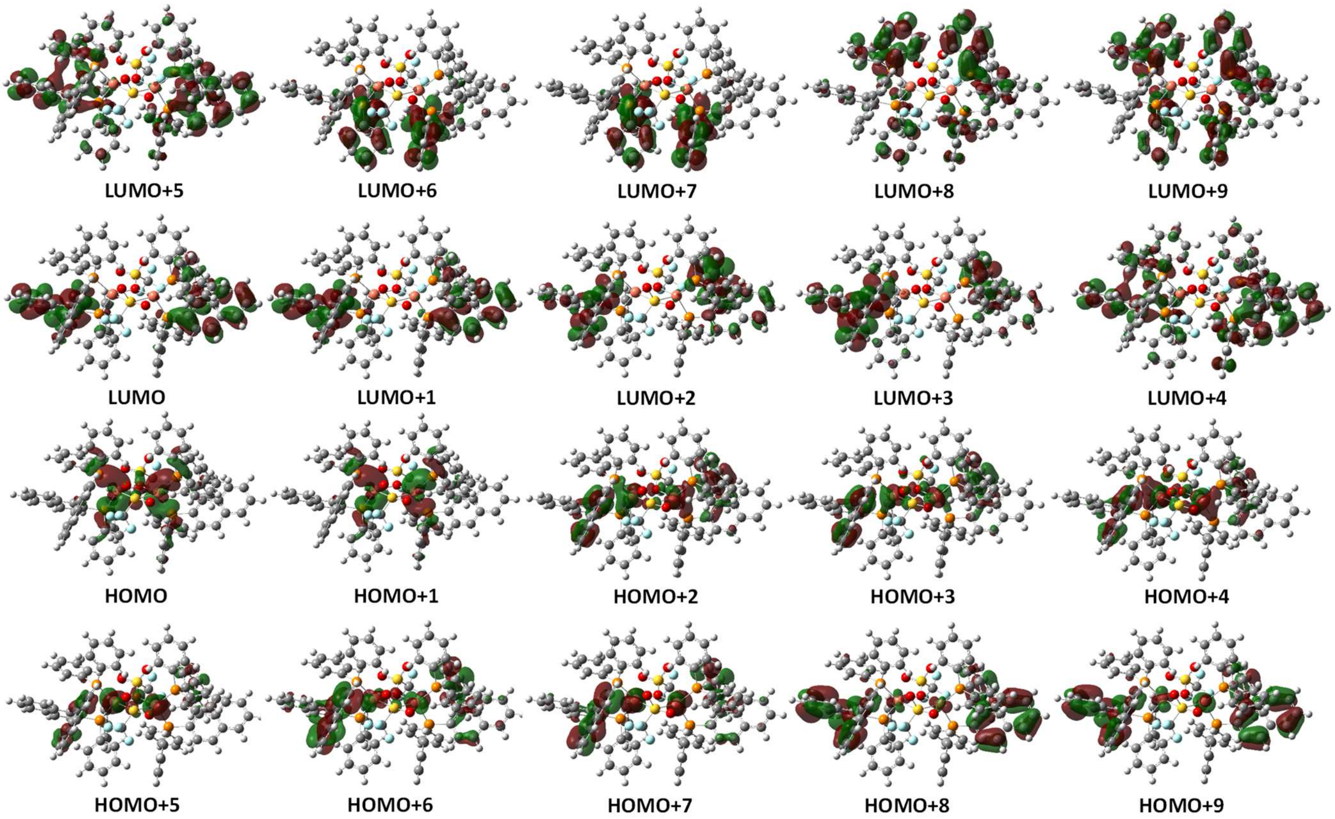

Figure S24. Selected molecular orbitals contributing $[Cu(BINAP)(OTf)]_2$ transitions. Atoms color: Cu (Pink), P (Orange),S (Yellow), F (Cyan), O (Red), C (Grey), H (White).

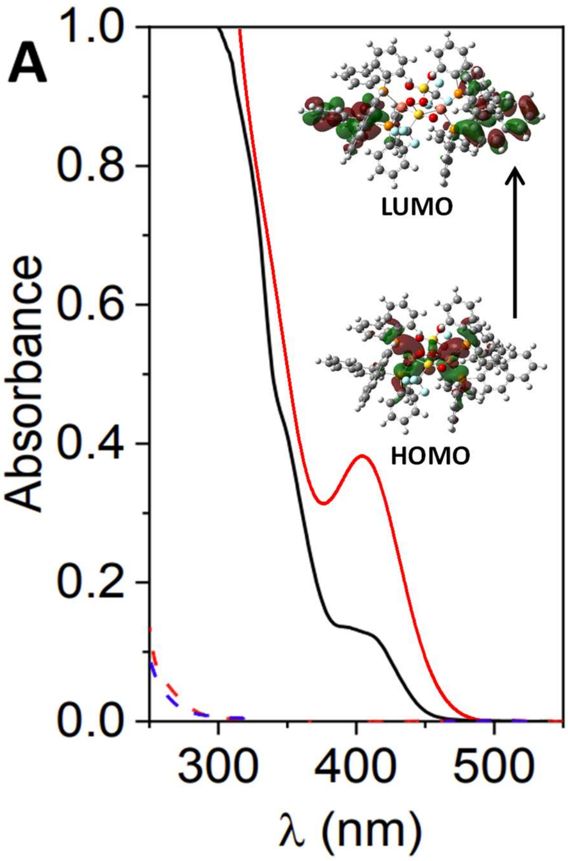

Figure S25. a) Absorption spectra of Cu/BINAP (black), calculated $[Cu(BINAP)_2(OTf)]_2$ (red), of *syn*-**2a** (dashed red) and *anti*-**2a** (dashed blue) isomers.

Table S8. First 60 calculated electronic transitions for $[Cu(BINAP)_2(OTf)]_2$ by TD-DFT using B3LYP/6-31G**

λ (nm)	Osc. Strength	Major contributions[a]
414.6	0.0895	HOMO->L+1 (56%), H-1->LUMO (41%),
414.1	0.0003	HOMO->LUMO (57%), H-1->L+1 (39%)
384.9	0.006	HOMO->L+2 (57%), H-1->L+3 (40%),
384.7	0.0301	HOMO->L+3 (57%), H-1->L+2 (39%)
377.8	0.0024	H-1->LUMO (55%), HOMO->L+1 (42%)
377.7	0.0	H-1->L+1 (58%), HOMO->LUMO (39%)
359.4	0.005	H-2->LUMO (62%), H-3->L+1 (31%)
359.2	0.0332	H-2->L+1 (61%), H-3->LUMO (33%)
354.9	0.0004	H-1->L+2 (56%), HOMO->L+3 (41%)
354.8	0.0	H-1->L+3 (58%), HOMO->L+2 (39%)
347.1	0.0583	HOMO->L+4 (61%), H-1->L+5 (31%)
347.0	0.0039	H-1->L+4 (47%), HOMO->L+5 (44%)
339.0	0.0013	H-4->LUMO (29%), H-3->LUMO (26%), H-5->L+1 (16%), H-2->L+3 (10%)
338.7	0.0144	H-4->L+1 (32%), H-3->L+1 (26%), H-5->LUMO (17%),
336.0	0.0264	H-2->L+2 (49%), H-3->L+3 (29%),
335.7	0.0032	H-2->L+3 (45%), H-3->L+2 (28%),
331.5	0.0045	HOMO->L+6 (55%), H-1->L+8 (32%),
330.9	0.0262	HOMO->L+8 (48%), H-1->L+6 (38%)
325.6	0.0236	H-6->LUMO (18%), H-4->L+1 (27%), H-3->L+1 (26%), H-2->LUMO (14%)
325.5	0.0119	H-6->L+1 (17%), H-4->LUMO (25%), H-3->LUMO (24%), H-2->L+1 (14%)
323.0	0.0002	HOMO->L+7 (55%), H-1->L+9 (19%),
322.1	0.0124	H-1->L+7 (43%), HOMO->L+9 (32%)
319.5	0.0066	H-5->L+1 (32%),H-9->LUMO (15%), H-7->LUMO (14%), H-2->L+1 (12%)
319.4	0.0179	H-1->L+4 (21%), HOMO->L+5 (20%), H-5->LUMO (19%),
319.1	0.0259	H-1->L+4 (26%), HOMO->L+5 (24%), H-5->LUMO (15%)
318.7	0.0015	H-1->L+5 (59%), HOMO->L+4 (34%)
318.2	0.0005	H-4->L+2 (33%), H-3->L+2 (30%), H-2->L+3 (11%), H-5->L+3 (14%),
317.9	0.0032	H-4->L+3 (30%), H-3->L+3 (30%), H-5->L+2 (15%), H-2->L+2 (12%)

313.8	0.005	L+9 ->LUMO (13%), H-7->L+1 (14%), H-6->L+2 (10%)
313.8	0.001	H-7->LUMO (13%),H-8->L+1 (12%), H-2->L+3 (10%)
311.6	0.002	H-10->LUMO (39%), H-11->L+1 (28%), H-8->LUMO (10%)
311.5	0.0063	H-10->L+1 (39%), H-11->LUMO (29%)
309.8	0.0025	HOMO->L+11 (16%), H-1->L+10 (16%), H-1->L+8 (12%), HOMO->L+6 (13%)
309.6	0.0077	HOMO->L+10 (15%), H-5->LUMO (14%), H-4->L+1 (11%),
308.9	0.0129	H-1->L+6 (22%), HOMO->L+8 (12%), H-6->LUMO (12%)
308.72	0.0025	H-6->L+1 (19%), H-4->LUMO (13%), H-5->L+1 (13%)
307.5	0.0164	HOMO->L+8 (25%), H-1->L+6 (20%), HOMO->L+10 (18%), H-1->L+11 (10%)
307.3	0.0311	HOMO->L+12 (51%), H-1->L+13 (14%), H-1->L+8 (12%)
307.0	0.0025	H-1->L+8 (13%), HOMO->L+11 (11%)
306.9	0.0073	H-1->L+12 (25%), HOMO->L+13 (21%), H-13->LUMO (10%)
306.7	0.0232	H-2->L+4 (22%), H-1->L+12 (12%), HOMO->L+13 (10%)
306.4	0.0	HOMO->L+11 (13%), H-12->LUMO (13%), H-1->L+8 (13%), H-13->L+1 (12%), H-1->L+10 (11%),
305.3	0.0042	H-3->L+3 (32%), H-4->L+3 (19%), H-2->L+2 (10%)
305.2	0.0027	H-3->L+2 (28%), H-4->L+2 (16%), H-2->L+3 (10%)
302.7	0.0346	H-2->L+4 (22%), H, H-12->L+1 (20%), -13->LUMO (19%), H-3->L+5 (10%),
302.6	0.0002	H-2->L+5 (22%), H-3->L+4 (17%), H-12->LUMO (15%), H-13->L+1 (15%)
301.1	0.0009	HOMO->L+9 (44%), H-1->L+7 (40%)
300.3	0.0023	H-1->L+9 (61%), HOMO->L+7 (21%)
300.0	0.0188	H-5->L+2 (20%), H-7->L+3 (17%), H-6->L+2 (13%)
299.9	0.0021	H-5->L+3 (19%), H-7->L+2 (18%), H-6->L+3 (13%)
297.9	0.0742	H-8->L+2 (25%), H-7->L+3 (19%), H-9->L+3 (15%), H-5->L+2 (12%)
297.8	0.0398	H-8->L+3 (24%), H-7->L+2 (16%), H-9->L+2 (16%), H-5->L+3 (13%)
295.3	0.0144	HOMO->L+13 (36%), H-1->L+14 (20%), HOMO->L+16 (14%), H-1->L+12 (12%)
294.8	0.0349	HOMO->L+14 (37%), H-1->L+13 (31%)
294.7	0.0007	H-9->LUMO (34%), H-6->L+1 (26%), H-7->LUMO (15%), H-5->L+1 (14%)
294.6	0.0021	H-9->L+1 (31%), H-6->LUMO (24%), H-7->L+1 (18%), H-5->LUMO (11%)
293.6	0.0066	H-4->L+4 (41%), H-3->L+4 (15%)
293.2	0.0042	H-6->L+2 (19%), H-4->L+3 (17%), H-5->L+2 (16%), H-8->L+2 (11%),
293.0	0.0018	H-5->L+3 (24%), H-6->L+3 (24%), H-4->L+2 (18%), H-8->L+3 (15%)
292.7	0.0175	H-2->L+6 (18%), H-3->L+5 (12%)

[a] Higher than 10%

Optimized X, Y, Z Coordinates of $[Cu(BINAP)(OTf)]_2$

C	6.60336100	-1.09005700	-4.85102800
C	6.78831800	-1.89721200	-3.70442900
C	6.31866500	-1.49033500	-2.47622800
C	5.62644400	-0.25547800	-2.31800400
C	5.43761500	0.55130900	-3.48607900
C	5.94060400	0.10906000	-4.73867500
C	4.74725600	1.77966600	-3.35976800
C	4.22859500	2.17116000	-2.15340100
C	4.39930000	1.39147000	-0.97337800
C	5.12408800	0.19430900	-1.04193100
C	9.20205000	0.47632700	0.14660700
C	9.71847900	-0.26543800	1.23446800
C	8.88072000	-1.08222700	1.95533200
C	7.50423500	-1.19352700	1.62205700
C	6.97271300	-0.46092000	0.51384400
C	7.87400200	0.37974500	-0.20388200
C	5.56854900	-0.57005000	0.17761400
C	4.74041500	-1.38301600	0.95538600
C	5.30340100	-2.09966900	2.05246200
C	6.63254500	-2.01630300	2.37206800
H	6.98089700	-1.42106700	-5.81399500
H	7.30464600	-2.84852100	-3.79395300
H	6.47118200	-2.12401000	-1.61206300
H	5.78381800	0.73950400	-5.60985900
H	4.60758800	2.40235200	-4.23901400

H	3.66447000	3.09189900	-2.10075800
P	3.37622900	1.90636100	0.49153300
H	9.85965000	1.12830900	-0.42083300
H	10.76916600	-0.18497400	1.49700900
H	9.25572000	-1.65475200	2.79955900
H	7.50694800	0.95987900	-1.04061400
P	2.91984900	-1.61393400	0.59936600
H	4.66824800	-2.73822700	2.65003400
H	7.02857000	-2.58302000	3.21061300
Cu	1.74341300	0.33615400	0.33264500
C	1.87044400	4.13747200	-0.32488500
C	3.09180500	3.71774700	0.21795800
C	4.06425500	4.68857500	0.51179800
C	3.82952300	6.03625500	0.24691000
C	2.62064400	6.43878500	-0.32362500
C	1.64432700	5.48629900	-0.60987600
H	1.10937300	3.40850500	-0.57036400
H	5.00878500	4.39911800	0.95674700
H	4.59403900	6.77084800	0.48380000
H	2.44171300	7.48796100	-0.54152400
H	0.70138200	5.78345700	-1.05621900
C	4.48794900	1.95119300	1.95935200
C	5.85709000	2.24177200	1.85698400
C	6.64523100	2.37651000	2.99924400
C	6.07305000	2.23358700	4.26436500
C	4.71101200	1.95710400	4.37717000
C	3.92042500	1.81678300	3.23457400
H	6.31575700	2.35532200	0.88129100
H	7.70560400	2.58786700	2.89695400
H	6.68592800	2.33952000	5.15508400
H	4.25408000	1.85217000	5.35700800
H	2.86053200	1.61726600	3.33822600
C	3.93927700	-4.73556700	-1.93617600
C	4.00904400	-3.74634100	-0.95593400
C	2.92526700	-2.88167200	-0.73594600
C	1.76519500	-3.04121600	-1.50560900
C	1.69569800	-4.03205000	-2.48543700
C	2.78264200	-4.87841200	-2.70394800
H	4.78829100	-5.39359500	-2.09838500
H	4.90756500	-3.65185600	-0.35578000
H	0.91742000	-2.38072100	-1.35279300
H	0.79452300	-4.13090300	-3.07980900
H	2.72999900	-5.64540300	-3.47147900
C	1.78105700	-4.02843500	4.41996600
C	1.92090000	-2.63999300	4.45771200
C	2.22079300	-1.93122000	3.29491900
C	2.39221100	-2.60283600	2.07365100
C	2.22874000	-3.99614000	2.04008500
C	1.92739900	-4.70260000	3.20637100
H	1.55358300	-4.57997100	5.32780300
H	1.79080400	-2.10137200	5.39128100
H	2.31854800	-0.85300800	3.34452700
H	2.35219800	-4.53677000	1.10879800
H	1.81285000	-5.78213600	3.16370400
O	-0.25457700	0.32811500	1.28182400
O	0.25481300	0.32585800	-1.28309900
Cu	-1.74323300	0.33486400	-0.33339300
S	0.38516900	1.01232700	-2.64686600
S	-0.38526000	1.01927000	2.64323900
O	-0.87898000	0.98908100	-3.39865100
O	1.12084500	2.27843000	-2.58735900
O	-1.12130700	2.28491400	2.57925200
O	0.87883300	0.99892300	3.39519500
C	1.49015800	-0.14123500	-3.61174500
C	-1.48992500	-0.13148200	3.61184900
F	0.82923400	-1.25646300	-3.94614200
F	1.89432400	0.46819900	-4.73078900
F	2.56533900	-0.48229600	-2.89232200
F	-2.56511900	-0.47501900	2.89363700
F	-1.89406000	0.48135100	4.72904500
F	-0.82874400	-1.24554700	3.94963500
C	-9.71839200	-0.26727500	-1.23461500

C	-9.20199500	0.47664000	-0.14820500
C	-7.87401600	0.38055400	0.20267700
C	-6.97275900	-0.46174600	-0.51318100
C	-7.50425100	-1.19654800	-1.61995400
C	-8.88066800	-1.08569200	-1.95366400
C	-6.63259800	-2.02103900	-2.36811200
C	-5.30351700	-2.10398100	-2.04813000
C	-4.74054400	-1.38512400	-0.95249400
C	-5.56865800	-0.57040400	-0.17653100
C	-6.78923500	-1.88883800	3.70827500
C	-6.60445900	-1.07910000	4.85308300
C	-5.94151600	0.11967300	4.73817200
C	-5.43816400	0.55901700	3.48469800
C	-5.62685000	-0.25038300	2.31840900
C	-6.31924600	-1.48480100	2.47926600
C	-5.12422300	0.19649600	1.04142400
C	-4.39923100	1.39337200	0.97033300
C	-4.22857100	2.17566800	2.14865500
C	-4.74757400	1.78697800	3.35578000
H	-10.76902400	-0.18716400	-1.49748300
H	-9.85956700	1.12990200	0.41779300
H	-7.50698700	0.96234900	1.03826400
H	-9.25563800	-1.65988900	-2.79676900
H	-7.02859400	-2.58944400	-3.20552900
H	-4.66841200	-2.74390600	-2.64428100
P	-2.92000800	-1.61556100	-0.59582100
H	-7.30568800	-2.83988400	3.79984000
H	-6.98227800	-1.40787100	5.81670500
H	-5.78485800	0.75207000	5.60796300
H	-6.47160600	-2.12042300	1.61650400
P	-3.37594400	1.90498700	-0.49552000
H	-3.66416100	3.09611700	2.09404200
H	-4.60797300	2.41160000	4.23366300
C	-1.76602900	-3.03714700	1.51339500
C	-2.92575000	-2.87987800	0.74273400
C	-4.00944900	-3.74421000	0.96442800
C	-3.93995400	-4.73085400	1.94729100
C	-2.78368100	-4.87141400	2.71603300
C	-1.69681100	-4.02538700	2.49586000
H	-0.91833200	-2.37686900	1.35924200
H	-4.90769200	-3.65148400	0.36357600
H	-4.78889800	-5.38865200	2.11078700
H	-2.73126300	-5.63637100	3.48560900
H	-0.79591000	-4.12248100	3.09094200
C	-2.39254600	-2.60827800	-2.06759500
C	-2.22111200	-1.93978800	-3.29058300
C	-1.92129300	-2.65155400	-4.45156200
C	-1.78159600	-4.03991400	-4.41028200
C	-1.92798500	-4.71097000	-3.19497300
C	-2.22920800	-4.00150300	-2.03047700
H	-2.31878900	-0.86170000	-3.34295000
H	-1.79117700	-2.11533100	-5.38651000
H	-1.55419200	-4.59378300	-5.31671400
H	-1.81355900	-5.79040700	-3.14955500
H	-2.35267100	-4.53974500	-1.09780800
C	-3.83044100	6.03476500	-0.25448000
C	-4.06539100	4.68659300	-0.51650600
C	-3.09113900	3.71676100	-0.22527700
C	-1.86787400	4.13792800	0.31203700
C	-1.64136100	5.48732900	0.59405200
C	-2.61941000	6.43879900	0.31044900
H	-4.59637400	6.76857400	-0.48921700
H	-5.01154900	4.39589500	-0.95719100
H	-1.10540700	3.40972600	0.55545600
H	-0.69679400	5.78570300	1.03610600
H	-2.44019200	7.48839200	0.52609600
C	-6.07187100	2.22635500	-4.26949400
C	-4.70980300	1.94967000	-4.38145600
C	-3.91957200	1.81109400	-3.23840500
C	-4.48748600	1.94748900	-1.96356000
C	-5.85665400	2.23820200	-1.86205800
C	-6.64443500	2.37120400	-3.00476900

H	-6.68447600	2.33093200	-5.16056000
H	-4.25257000	1.84319900	-5.36098800
H	-2.85967300	1.61125600	-3.34146300
H	-6.31567400	2.35310700	-0.88669300
H	-7.70484200	2.58271300	-2.90313000

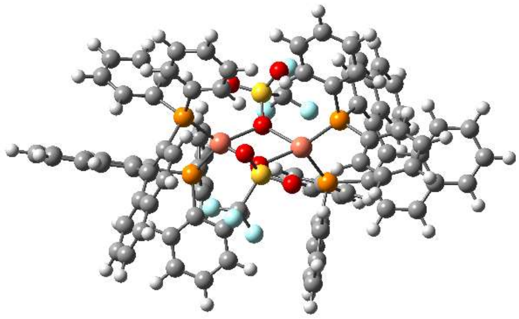

Optimized X, Y, Z Coordinates of BINAP

C	1.50774706	3.87021540	-3.47218342
C	1.93612469	3.84768825	-2.12395486
C	1.38325247	2.96061676	-1.22836436
C	0.36991832	2.04456603	-1.62972574
C	-0.05867531	2.07097461	-2.99733928
C	0.53208485	2.99852140	-3.89627534
C	-1.06480240	1.16531040	-3.41765311
C	-1.62419159	0.27841950	-2.53170307
C	-1.22430541	0.24030444	-1.16609770
C	-0.22618556	1.10629069	-0.71834521
C	-1.93668897	3.84754387	2.12338233
C	-1.50826933	3.87029168	3.47159057
C	-0.53241845	2.99883680	3.89574650
C	0.05849348	2.07131044	2.99689343
C	-0.37014790	2.04467210	1.62929862
C	-1.38366845	2.96048637	1.22786653
C	0.22608368	1.10640387	0.71799211
C	1.22437258	0.24063721	1.16580225
C	1.62430276	0.27898216	2.53139360
C	1.06480651	1.16587702	3.41726675
H	1.95184256	4.57544757	-4.16860047
H	2.70946772	4.53492566	-1.79400056
H	1.72247603	2.95162722	-0.19930026
H	0.19611844	3.00519065	-4.92996970
H	-1.38751289	1.18160426	-4.45566860
H	-2.38254021	-0.41630692	-2.87637729
P	-1.96153351	-0.99038916	0.02163792
H	-2.71017784	4.53459477	1.79338345
H	-1.95248415	4.57550081	4.16795580
H	-0.19642718	3.00569247	4.92943144
H	-1.72292295	2.95131210	0.19881549
P	1.96174994	-0.99013232	-0.02173202
H	2.38278402	-0.41558282	2.87610765
H	1.38756890	1.18236099	4.45526307
C	-1.54463445	-3.14491014	-1.70120965
C	-2.53554570	-2.31997281	-1.13795075
C	-3.88030260	-2.60452842	-1.41502623
C	-4.22499491	-3.67930010	-2.23899629
C	-3.23340276	-4.48097901	-2.80297535
C	-1.88988145	-4.20780324	-2.53323536
H	-0.49667264	-2.94717977	-1.48862168
H	-4.66337372	-1.98652053	-0.98837055
H	-5.27253006	-3.88526282	-2.44150241
H	-3.50344922	-5.31507830	-3.44427290
H	-1.10991524	-4.82875570	-2.96498994
C	-3.54946692	-0.17597472	0.52742109
C	-4.23130935	0.79172726	-0.22749530
C	-5.42033764	1.35199470	0.24124920
C	-5.95029765	0.95185836	1.46945842
C	-5.28220943	-0.00758712	2.23097553
C	-4.08815757	-0.55989764	1.76593478
H	-3.83102173	1.11194825	-1.18420481
H	-5.93425886	2.10114645	-0.35484749
H	-6.87568742	1.38968227	1.83272745
H	-5.68373376	-0.31896012	3.19110015
H	-3.56174224	-1.29220893	2.37237597
C	5.42051533	1.35234475	-0.24117947
C	4.23127171	0.79233799	0.22734910
C	3.54991907	-0.17599126	-0.52719044
C	4.08932055	-0.56081026	-1.76512885
C	5.28358921	-0.00878482	-2.22992359
C	5.95118994	0.95130381	-1.46877579
H	5.93404211	2.10199772	0.35462684
H	3.83045961	1.11324113	1.18360718
H	3.56328516	-1.29361026	-2.37131283
H	5.68566891	-0.32086385	-3.18958685
H	6.87675069	1.38890802	-1.83187268
C	3.23279400	-4.48063141	2.80340345
C	1.88933986	-4.20738052	2.53340042
C	1.54431001	-3.14452510	1.70123790

C	2.53537420	-2.31970956	1.13808375
C	3.88006561	-2.60432303	1.41543620
C	4.22454052	-3.67905558	2.23955028
H	3.50265834	-5.31470634	3.44480951
H	1.10925499	-4.82824700	2.96506506
H	0.49639989	-2.94672646	1.48845731
H	4.66326333	-1.98639959	0.98889271
H	5.27202724	-3.88505542	2.44226950

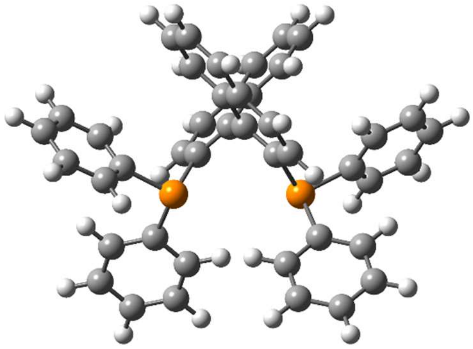

Table S9. First 60 calculated electronic transitions for Binap by TD-DFT using B3LYP/6-31G**

□ (nm)	Osc. Strength	Major contributions[a]
339.92	0.0112	HOMO->LUMO (93%)
337.19	0.0794	HOMO->L+1 (92%)
318.77	0.055	H-1->LUMO (85%)
314.51	0.0387	H-1->L+1 (80%)
305.42	0.1463	H-2->LUMO (84%)
304.19	0.0386	HOMO->L+2 (73%)
300.84	0.0121	H-2->L+1 (64%), H-3->LUMO (21%)
293.11	0.0081	H-3->L+1 (53%), H-2->L+2 (17%), HOMO->L+5 (10%)
286.22	0.0013	H-3->LUMO (58%), H-2->L+1 (18%), HOMO->L+2 (11%)
285.26	0.0432	HOMO->L+3 (58%), H-1->L+2 (32%)
279.95	0.0253	H-1->L+2 (55%), HOMO->L+3 (26%)
279.63	0.2145	HOMO->L+4 (80%), H-1->L+3 (10%)
278.57	0.0998	H-3->L+1 (33%), HOMO->L+5 (19%), H-2->L+2 (17%), H-4->LUMO (12%)
272.06	0.0029	HOMO->L+5 (52%), H-2->L+2 (43%)
269.43	0.0098	H-4->L+1 (27%), H-3->L+2 (15%), H-1->L+5 (13%)
264.91	0.064	H-1->L+3 (74%), HOMO->L+4 (12%)
262.70	0.0072	H-1->L+4 (79%)
259.59	0.0278	HOMO->L+6 (56%)
259.10	0.0159	HOMO->L+7 (48%), H-2->L+5 (14%), H-1->L+5 (11%),
257.87	0.0001	H-2->L+3 (54%), H-3->L+2 (27%)

[a] Higher than 10%

Optimized X, Y, Z Coordinates of Cu-BINAP(tph)

C	0.00000000	0.00000000	0.00000000
C	0.00000000	0.00000000	1.41410773
C	1.18326444	0.00000000	2.11748933
C	2.44160775	-0.00020747	1.44956944
C	2.43122732	0.01149653	0.01727078
C	1.19443638	0.00857377	-0.68050213
C	3.66670424	0.02817254	-0.67351299
C	4.85257113	0.03831517	0.01209640
C	4.89644381	0.00900583	1.43827539
C	3.69708522	-0.00451221	2.16146809
C	2.70650581	-3.75411679	3.98388545
C	2.54089353	-3.87598302	5.38394065
C	2.72494957	-2.77778254	6.19030778
C	3.08201545	-1.51808126	5.63885182
C	3.25122940	-1.38538889	4.22274648
C	3.04798535	-2.54538950	3.42015091
C	3.62781169	-0.11278455	3.66286032
C	3.82447447	0.97965092	4.51137743
C	3.63210084	0.82525596	5.91321357
C	3.27119417	-0.37982301	6.45951935
H	-0.94081552	-0.00179148	-0.54214436
H	-0.94376358	0.00273138	1.95136738

H	1.15857748	0.00466547	3.19980931
H	1.21333072	0.01583521	-1.76695198
H	3.66535872	0.04229579	-1.75999263
H	5.78209453	0.07211968	-0.54236810
P	6.56729503	0.36577419	2.15462814
H	2.55871382	-4.62170705	3.34758082
H	2.26748445	-4.83415859	5.81551515
H	2.60101548	-2.85526695	7.26716885
H	3.16598448	-2.47132036	2.34571414
P	4.39508835	2.60385518	3.83554694
H	3.77360373	1.67998629	6.56322035
H	3.12540865	-0.47213978	7.53252115
Cu	6.18266499	2.48290698	2.49920377
C	8.67347796	0.62918824	0.34339106
C	7.76364607	-0.27424726	0.90913745
C	7.82645622	-1.63034226	0.54403751
C	8.77386409	-2.07011520	-0.37633319
C	9.67764470	-1.16288799	-0.93845610
C	9.62570395	0.18150603	-0.57739854
H	8.64559773	1.68111260	0.61060500
H	7.13356439	-2.34633542	0.97449549
H	8.81054392	-3.11979057	-0.65349781
H	10.41836200	-1.50805589	-1.65415248
H	10.32314163	0.89395637	-1.00725037
C	6.90734368	-0.69926876	3.61073685
C	6.48293034	-2.02965748	3.73183470
C	6.87300036	-2.80070204	4.82616364
C	7.70400835	-2.25763900	5.80768458
C	8.13966019	-0.93719940	5.69414362
C	7.74181429	-0.16272721	4.60384107
H	5.82939106	-2.46338136	2.98324520
H	6.52321031	-3.82505494	4.91229158
H	8.00922182	-2.86109764	6.65745031
H	8.78967707	-0.50885884	6.45131693
H	8.08781125	0.86436265	4.51880353
C	0.53102107	3.85883468	3.05286505
C	1.62288324	3.21704998	3.63486149
C	2.90848459	3.37347072	3.09301312
C	3.08332148	4.18808507	1.96328610
C	1.98689628	4.83049283	1.38574551
C	0.71206576	4.66618523	1.92731053
H	-0.46041822	3.72916131	3.47714059
H	1.47518249	2.59019228	4.50925502
H	4.07381909	4.32623459	1.54057809
H	2.13430882	5.45730350	0.51161980
H	-0.14006043	5.16458905	1.47432113
C	5.60828303	5.19717124	7.49094044
C	6.28066533	4.00815170	7.19754695
C	5.88042544	3.22737050	6.11600449
C	4.78775666	3.61516307	5.31906922
C	4.12089501	4.80976027	5.62014145
C	4.53196684	5.59490497	6.69949268
H	5.92725834	5.81122654	8.32774732
H	7.12295272	3.69336407	7.80657468
H	6.41110762	2.30432213	5.89712579
H	3.28445150	5.13313656	5.01051824
H	4.00804065	6.52081208	6.91765358
O	6.55298160	4.23410603	1.80763238
S	7.58613470	4.69841523	0.78012600
O	7.74157942	6.15086381	0.76356671
O	8.79327230	3.85653621	0.76035721
C	6.69020616	4.27713669	-0.79843979
F	5.50067187	4.89721626	-0.84826957
F	7.40694629	4.63904848	-1.86591469
F	6.46629154	2.94782722	-0.87044137

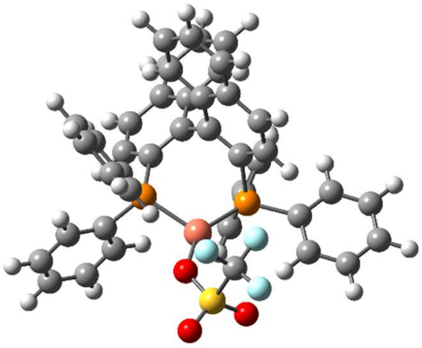

Table S10. First 60 calculated electronic transitions for Cu-Binap_Tf by TD-DFT using B3LYP/6-31G**

λ (nm)	Osc. Strength	Major contributions[a]
408.10	0.0452	HOMO->LUMO (94%)
382.42	0.0189	HOMO->L+1 (96%)
344.55	0.046	HOMO->L+2 (60%), H-1->LUMO (35%),
343.47	0.015	H-1->LUMO (56%), HOMO->L+2 (37%)
332.42	0.0174	HOMO->L+3 (77%)
328.17	0.0142	H-1->L+1 (66%), H-2->LUMO (24%)
322.45	0.0408	HOMO->L+4 (86%)
314.80	0.0074	H-2->LUMO (37%), H-3->LUMO (26%), H-2->L+1 (13%)H
313.01	0.0093	HOMO->L+5 (68%), H-2->LUMO (14%)
306.59	0.0291	H-3->LUMO (53%), H-4->LUMO (12%)
303.76	0.0216	H-4->LUMO (66%)
301.59	0.0406	H-2->L+1 (73%), H-2->LUMO (10%)
298.38	0.0113	HOMO->L+6 (78%)
297.79	0.0126	H-1->L+2 (31%), H-3->L+1 (21%), H-5->LUMO (19%)
294.23	0.0012	H-5->LUMO (55%), H-3->L+1 (30%)
292.12	0.0432	H-1->L+2 (41%), HOMO->L+7 (17%), H-3->L+1 (17%)
289.80	0.0763	HOMO->L+7 (62%), H-3->L+1 (11%)
286.56	0.0186	H-4->L+1 (59%), H-4->LUMO (10%)
286.04	0.0042	HOMO->L+8 (36%), H-1->L+3 (14%), H-2->L+2 (10%)
285.28	0.017	HOMO->L+8 (54%), H-1->L+3 (14%), HOMO->L+9 (10%)

[a] Higher than 10%

References

[1] Braunschweig, H.; Guethlein, F. Transition-Metal-Catalyzed Synthesis of Diboranes(4). *Angew. Chem. Int. Ed.* **2011**, *50*, 12613–12616.

[2] Wrackmeyer, B. Carbon-13 NMR spectroscopy of boron compounds. *Prog. NMR Spec.* **1979**, *12*, 227–259.

[3] Corpas, J.; Mauleón, P.; Arrayás, R. G.; Carretero, J. C. anti-Hydroarylation of Activated Internal Alkynes: Merging Pd and Energy Transfer Catalysis. *Org. Lett.* **2020**, *22,* 6473–6478.

[4] Lakowicz, J. R. Principles of Fluorescence Spectroscopy, 2nd Ed., Kluwer Academic/Plenum Publishers, New York, London, Moscow, Dordrecht, 1999.

[5] Martinez-Haya, R.; Miranda, M. A.; M. L. Marin. Metal-Free Photocatalytic Reductive Dehalogenation Using Visible-Light: A Time-Resolved Mechanistic Study. *Eur. J. Org. Chem.* **2017**, 2164–2169.

[6] Mackay, E. G.; Nörret, M.; Wong, L. S. -M.; Louis, I.; Lawrence, A. L.; Willis, A. C.; Sherburn, M. S. A Domino Diels–Alder Approach toward the Tetracyclic Nicandrenone Framework. *Org. Lett.* **2015**, *17,* 5517–5519.

[7] Neises, B.; Steglich, W. Simple Method for the Esterification of Carboxylic Acids. *Angew. Chem., Int. Ed.* **1978**, *17*, 522-524.

[8] Kim-Lee, S.-H.; Mauleón, P.; Gómez Arrayás, R.; Carretero, J. C. Dynamic Multiligand Catalysis: A Polar to Radical Cross-over Strategy Expands Alkyne Carboboration to Unactivated Secondary Alkyl Halides. *Chem* **2021**, *7*, 2212-2226.

[9] Leggio, A.; Belsito, E. L.; De Luca, G.; Di Gioia, M. L.; Leotta, V.; Romio, E.; Siciliano, C.; Liguori, A. One-Pot Synthesis of Amides from Carboxylic Acids Activated Using Thionyl Chloride. *RSC Adv.* **2016**, *6*, 34468-34475.

[10] Cruché, C.; Neiderer, W.; Collins, S. K. Heteroleptic Copper-Based Complexes for Energy-Transfer Processes: E → Z Isomerization and Tandem Photocatalytic Sequences. *ACS Catal*. **2021**, *11*, 8829-8836.

[11] Kunkely, H.; Pawlowski, V.; Vogler, A. Copper(I) Binap Complexes (Binap = (2,20 Bis(diphenylphosphino)-1,10-binaphthyl). Luminescence from IL and LLCT States. *Inorg. Chem. Commun.* **2008**, *11*, 1003-1005.

[12] Brégent, T.; Bouillon, J. P.; Poisson, T. Copper-Photocatalyzed Contra-Thermodynamic Isomerization of Polarized Alkenes. *Org. Lett*. **2020**, *22*, 7688−7693.

[13] Sendra, J.; Manzano, R.; Reyes, E.; Vicario, J. L.; Fernández, E. Catalytic Stereoselective Borylative Transannular Reactions. *Angew. Chem. Int. Ed.* **2020**, *59*, 2100-2104.

[14] Moure, A. L.; Arrayás, R. G.; Cárdenas, D. J.; Alonso, I.; Carretero J. C. Regiocontrolled Cu^{I}-Catalyzed Borylation of Propargylic-Functionalized Internal Alkynes. *J. Am. Chem. Soc*. **2012**, *134*, 7219-7222.

[15] Bunten, K. A.; Farrar, D. H.; Poë, A. J.; Lough A. Stoichiometric and Catalytic Oxidation of BINAP by Dioxygen in a Rhodium(I) Complex *Organometallics* **2002**, *21*, 3344-3350.

[16] a) Hattori, G.; Sakata, K.; Matsuzawa, H.; Tanabe, Y.; Miyake, Y.; Nishibayashi, Y. Copper-Catalyzed Enantioselective Propargylic Amination of Propargylic Esters with Amines: Copper−Allenylidene Complexes as Key Intermediates. *J. Am. Chem. Soc.* **2010***, 132*, 10592-10608. b) Banerjee, A.; Sarkar, S.; Shah, J. A.; Frederiks, N. C.; Bazan-Bergamino, E. A.; Johnson, C. J.; Ngai, M.-Y. Excited-State Copper Catalysis for the Synthesis of Heterocycles. *Angew. Chem. Int. Ed.* **2022***, 61*, e202113841.

[17] Miyata, K.; Nakagawa, T.; Kawakami, R.; Kita, Y.; Sugimoto, K.; Nakashima, T.; Harada, T.; Kawai, T.; Hasegawa, Y. Remarkable Luminescence Properties of Lanthanide Complexes with Asymmetric Dodecahedron Structures. *Chem. Eur. J.* **2011***, 17*, 521-528.

[18] Cain, M. F.; Hughes, R. P.; Glueck, D. S.; Golen, J. A.; Moore, C. E.; Rheingold, A. L. Synthesis and Structure of Intermediates in Copper-Catalyzed Alkylation of Diphenylphosphine. *Inorg. Chem.* **2010***, 49*, 7650–7662.

[19] Nieto, S.; Metola, P.; Lynch, V. M.; Anslyn, E. V. Synthesis of a Novel Bisphosphonium Salt Based on 2,2′-Bis(diphenylphosphino)-1,1′-binaphthyl (Binap). *Organometallics* **2008**, *27*, 3608-3610.

[20] Zang, L.; Cheng, J.; Carry, B.; Hou, Z. Catalytic Boracarboxylation of Alkynes with Diborane and Carbon Dioxide by an N-Heterocyclic Carbene Copper Catalyst. *J. Am. Chem. Soc*. **2012**, *134*, 14314−14317.

[21] Baughman, N. N.; Akhmedov, N. G.; Petersen, J. L.; Popp, B. V. Experimental and Computational Analysis of CO_2 Addition Reactions Relevant to Copper-Catalyzed Boracarboxylation of Vinyl Arenes: Evidence for a Phosphine-Promoted Mechanism. *Organometallics* **2021**, *40*, 23−37.

[22] Lee, G. M.; Bowes, E. G.; Vogels, C. M.; Decken, A.; Westcott, S. A. Cyclisations of alkynoic acids using copper(I) arylspiroborate complexes, *Tetrahedron* **2019**, *75* 2106−2112.

[23] Becke, A. D. Density-Functional Exchange-Energy Approximation with Correct Asymptotic Behavior. *Phys. Rev. A* **1988***, 38*, 3098–3100.

[24] Becke, A. D. Density‐functional Thermochemistry. III. The Role of Exact Exchange. *J. Chem. Phys.* **1993***, 98,* 5648‐5652.

[25] Marenich, A. V.; Cramer, C. J.; Truhlar, D. G. Universal Solvation Model Based on Solute Electron Density and on a Continuum Model of the Solvent Defined by the Bulk Dielectric Constant and Atomic Surface Tensions. *J. Phys. Chem. B* **2009**, *113*, 6378–6396.

[26] Frisch, M. J.; Trucks, G. W.; Schlegel, H. B.; Scuseria, G. E.; Robb, M. A.; Cheeseman, J. R.; Scalmani, G.; Baro-ne, V.; Petersson, G. A.; Nakatsuji, H.; Li, X.; Caricato, M.; Marenich, A. V.; Bloino, J.; Janesko, B. G.; Gomperts, R.; Mennucci, B.; Hratc, D. J. Gaussian 16, Revision C.01. Gaussian Inc., Wallingford CT, 2016.

[27] O'Boyle N. M., Tenderholt A.L. Langer K. M, *J. Comp. Chem.* **2008***, 29*, 839-845.

NMR spectra

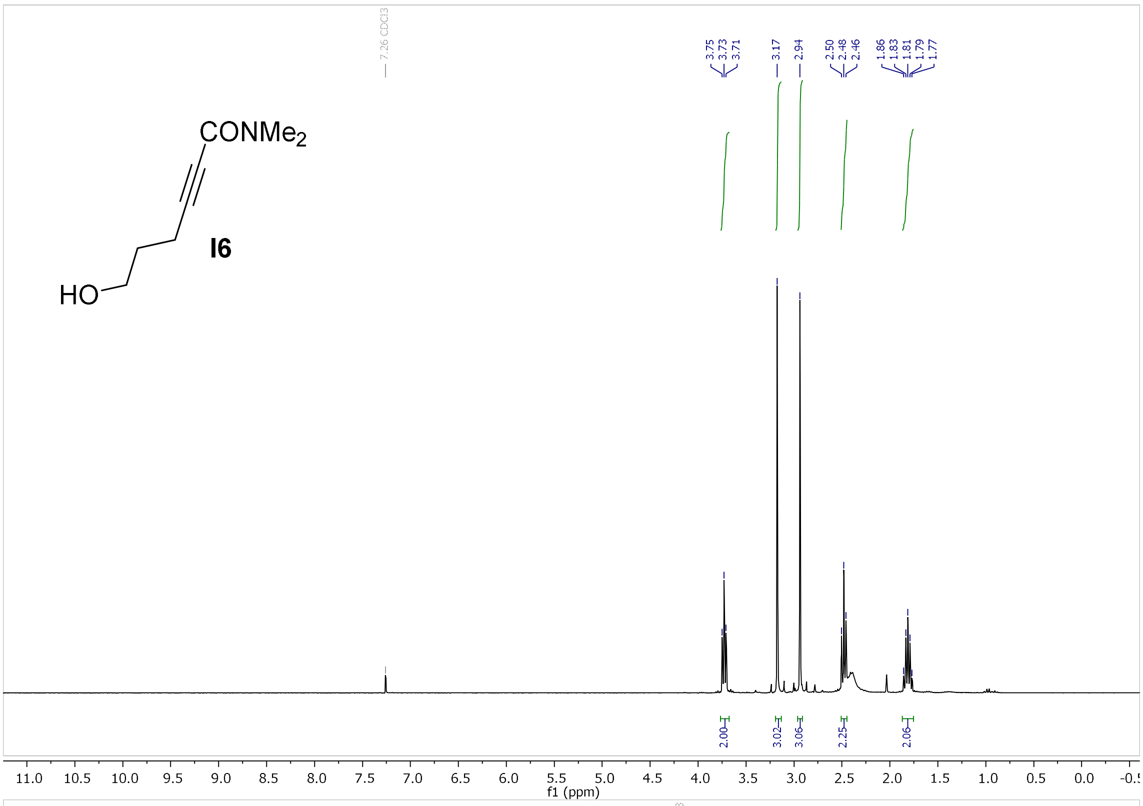

CONONMe2
I6
HO
7.26 CDCl3
3.75
3.73
3.71
3.17
2.94
2.50
2.48
2.46
1.86
1.83
1.81
1.79
1.77
2.00
3.02
3.06
2.25
2.06
11.0 10.5 10.0 9.5 9.0 8.5 8.0 7.5 7.0 6.5 6.0 5.5 5.0 4.5 4.0 3.5 3.0 2.5 2.0 1.5 1.0 0.5 0.0
f1 (ppm)

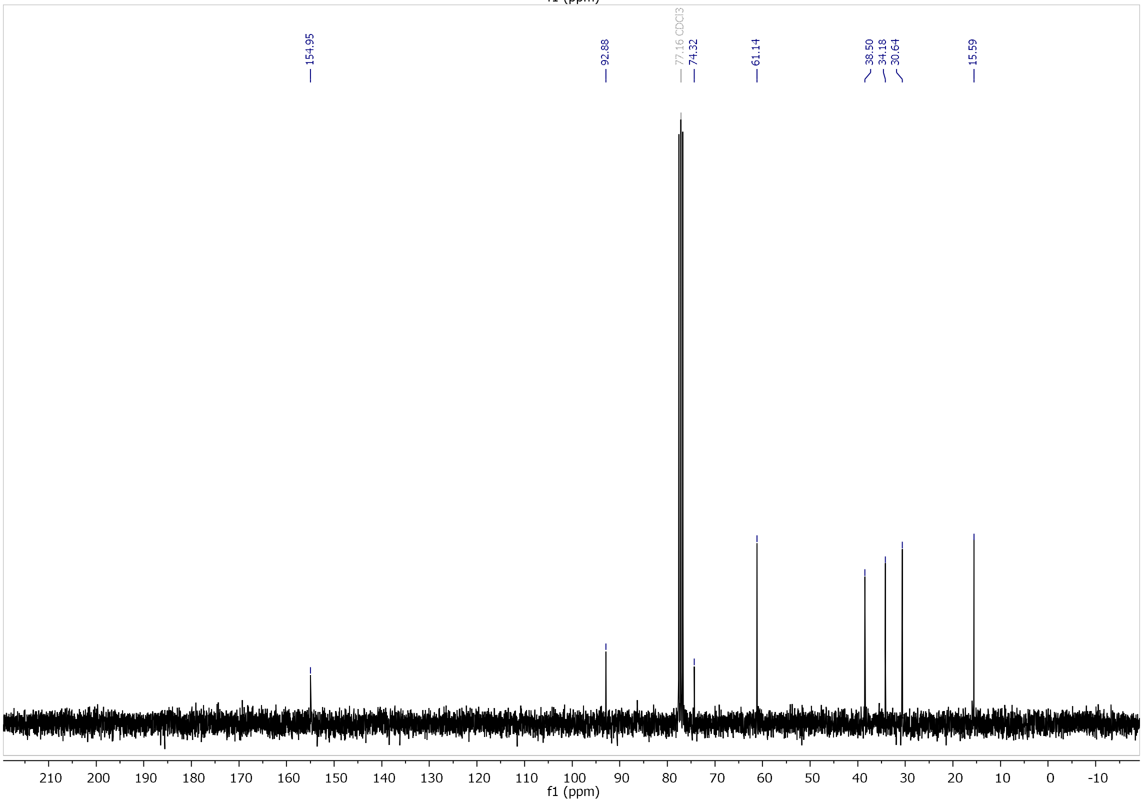

154.95
92.88
77.16 CDCl3
74.32
61.14
38.50
34.18
30.64
15.59
210 200 190 180 170 160 150 140 130 120 110 100 90 80 70 60 50 40 30 20 10 0 -10
f1 (ppm)

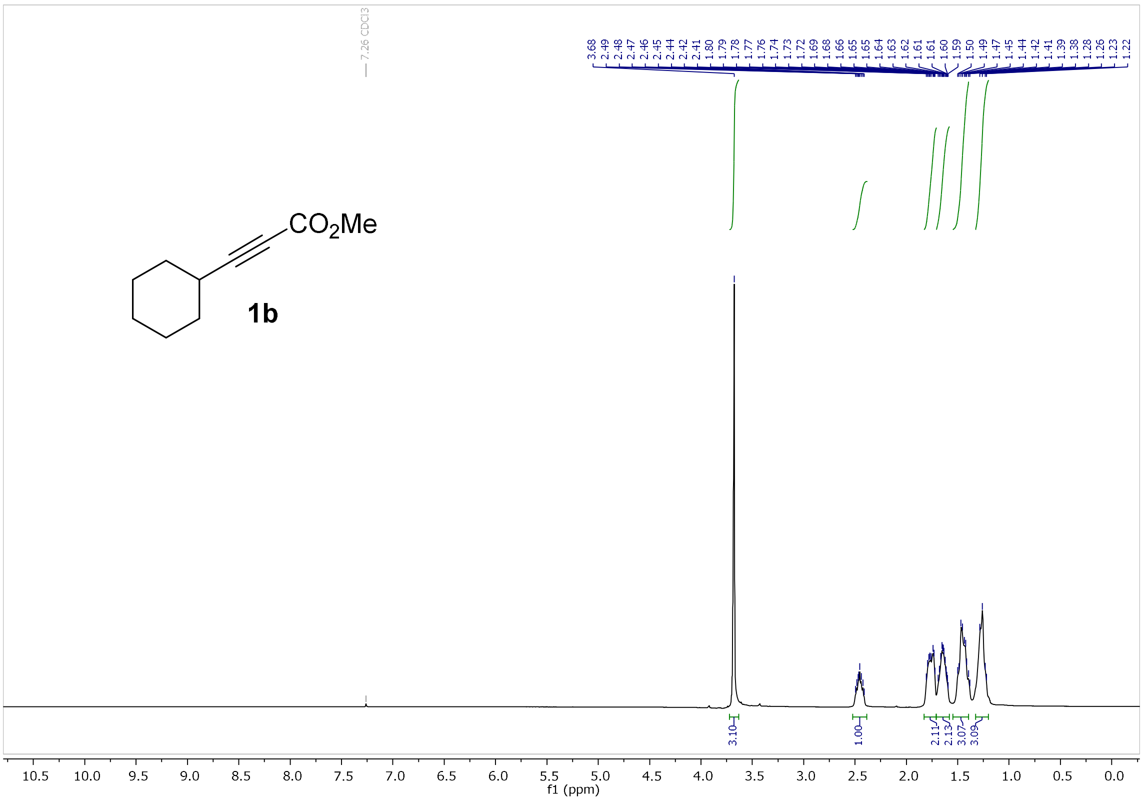
7.26 CDCl3
3.68 2.49 2.48 2.47 2.46 2.45 2.44 2.42 2.41 1.80 1.79 1.78 1.77 1.76 1.74 1.73 1.72 1.69 1.68 1.66 1.65 1.65 1.64 1.63 1.62 1.61 1.61 1.60 1.59 1.50 1.49 1.47 1.45 1.44 1.42 1.41 1.39 1.38 1.28 1.26 1.23 1.22
CO2Me
1b
3.10
1.00
2.11
2.13
3.07
3.09
f1 (ppm)

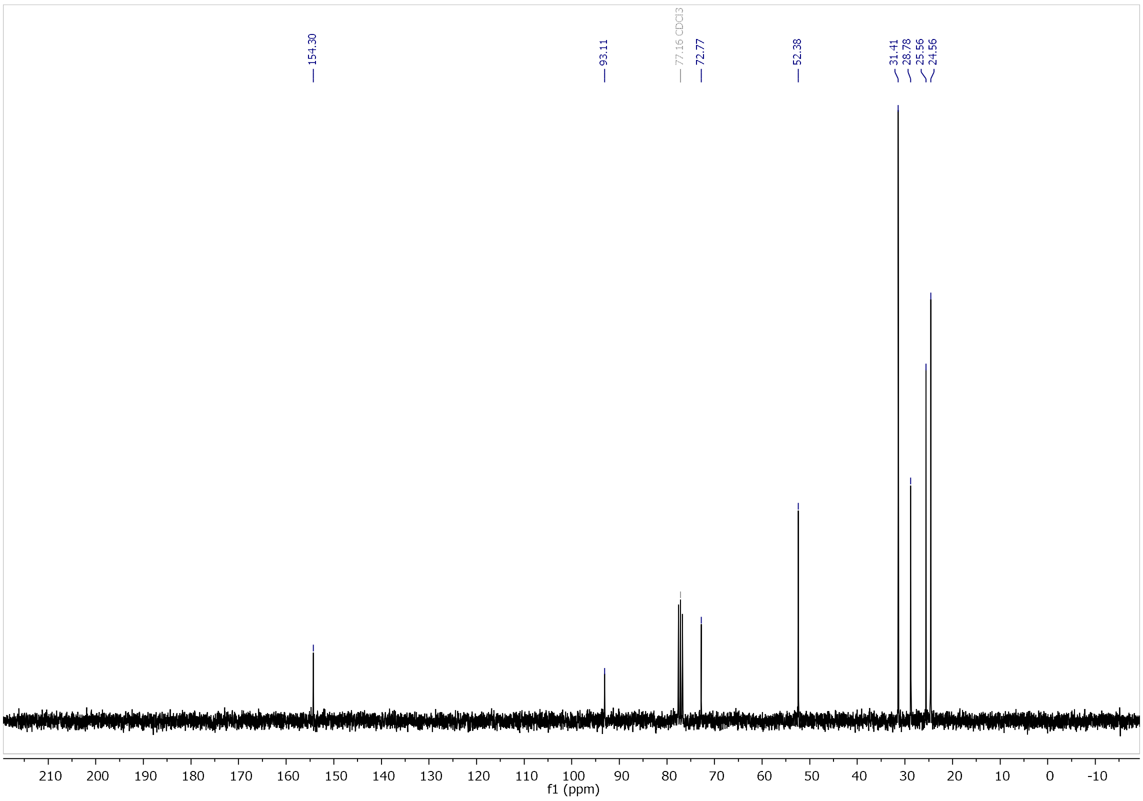
154.30
93.11
77.16 CDCl3
72.77
52.38
31.41
28.78
25.56
24.56
f1 (ppm)

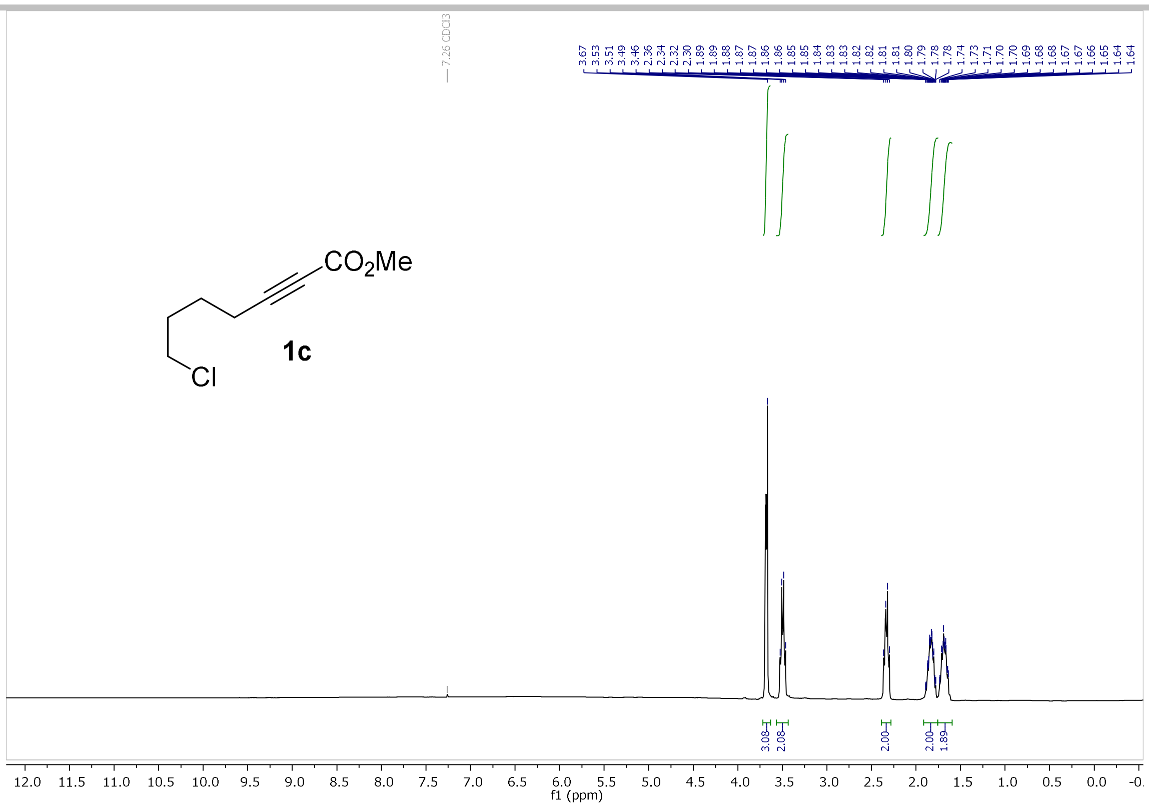
CO2Me
Cl
1c
f1 (ppm)

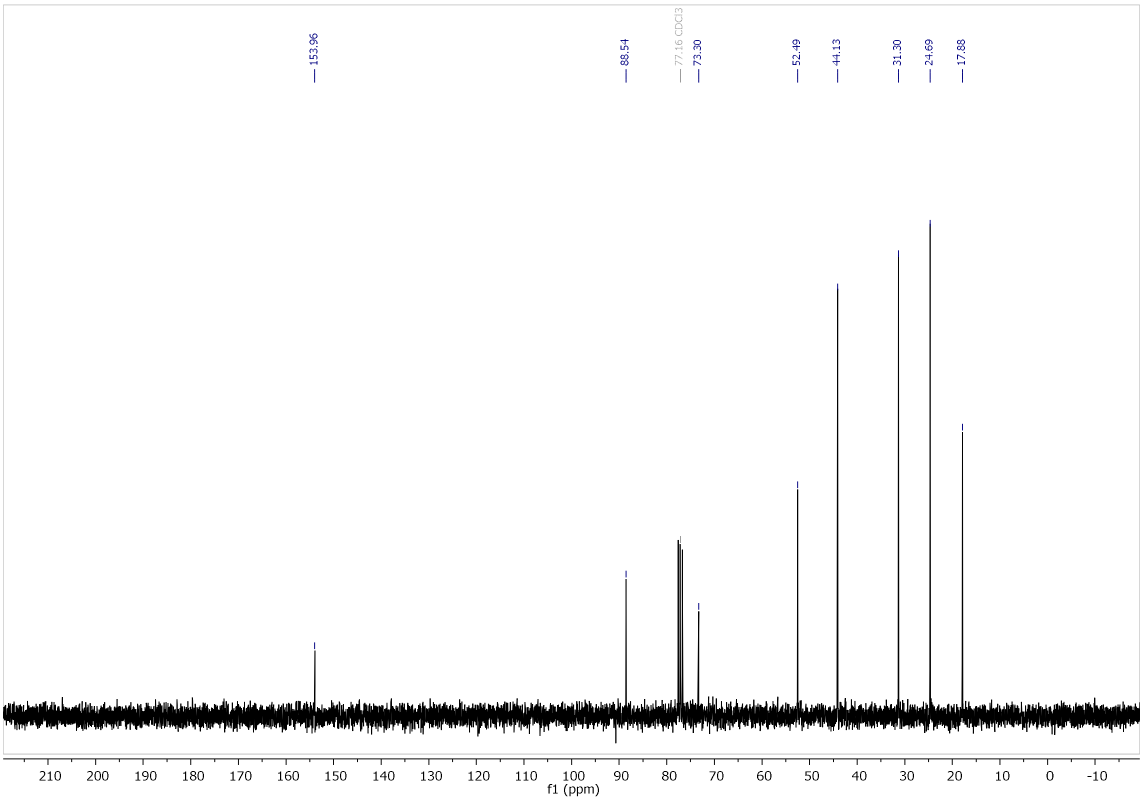
153.96
88.54
77.16 CDCl3
73.30
52.49
44.13
31.30
24.69
17.88
f1 (ppm)

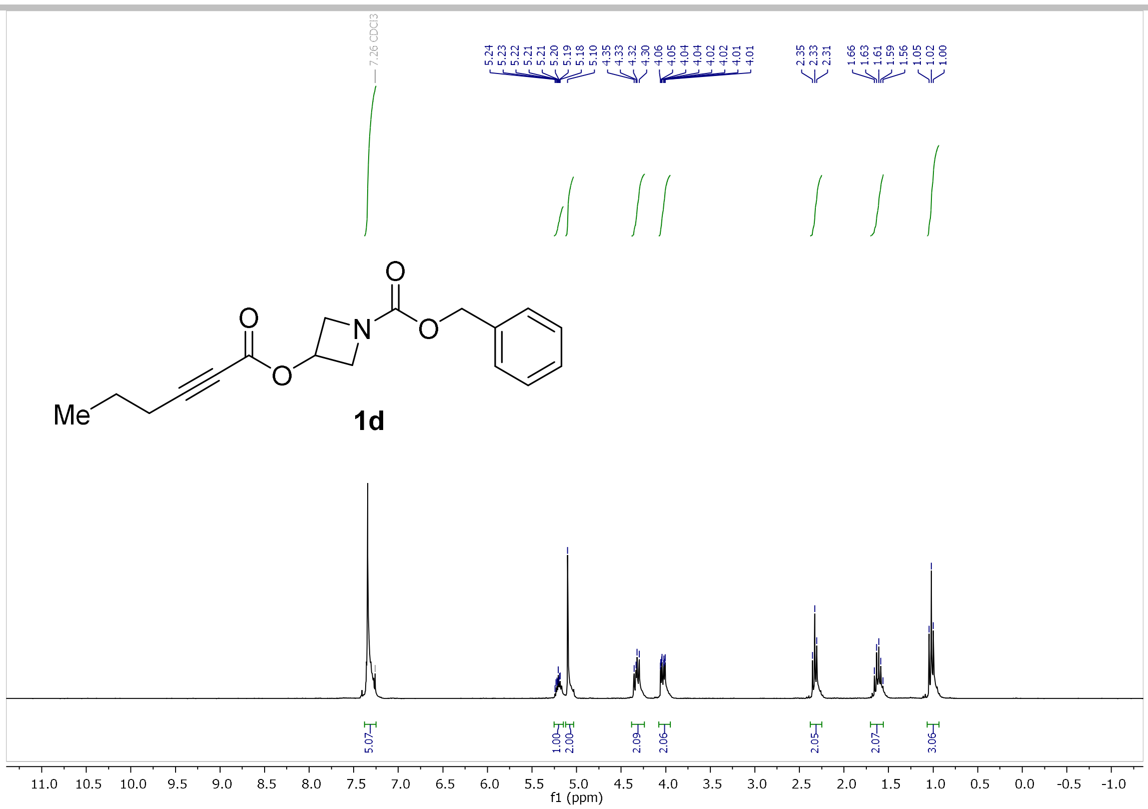
7.26 CDCl3
5.24
5.23
5.22
5.21
5.21
5.20
5.19
5.18
5.10
4.35
4.33
4.32
4.30
4.06
4.05
4.04
4.04
4.02
4.02
4.01
4.01
2.35
2.33
2.31
1.66
1.63
1.61
1.59
1.56
1.05
1.02
1.00
Me
O
O
N
O
O
1d
5.07
1.00
2.00
2.09
2.06
2.05
2.07
3.06
11.0 10.5 10.0 9.5 9.0 8.5 8.0 7.5 7.0 6.5 6.0 5.5 5.0 4.5 4.0 3.5 3.0 2.5 2.0 1.5 1.0 0.5 0.0 -0.5 -1.0
f1 (ppm)

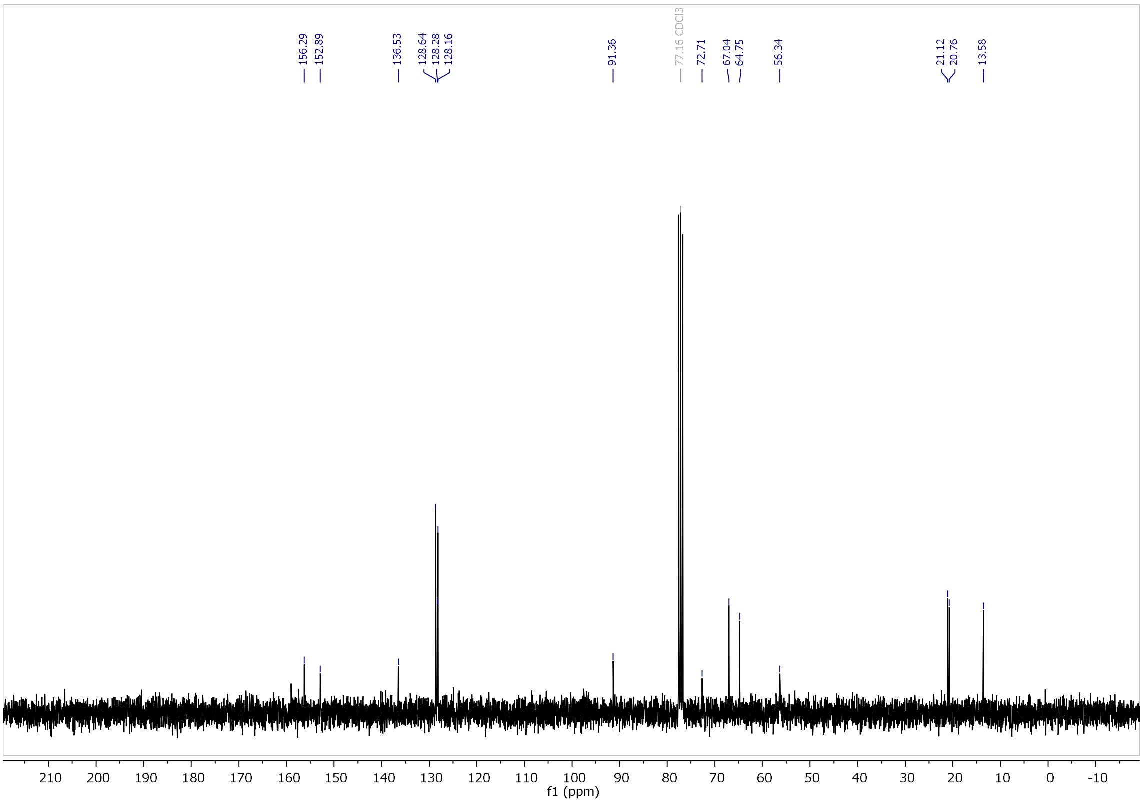
156.29
152.89
136.53
128.64
128.28
128.16
91.36
77.16 CDCl3
72.71
67.04
64.75
56.34
21.12
20.76
13.58
210 200 190 180 170 160 150 140 130 120 110 100 90 80 70 60 50 40 30 20 10 0 -10
f1 (ppm)

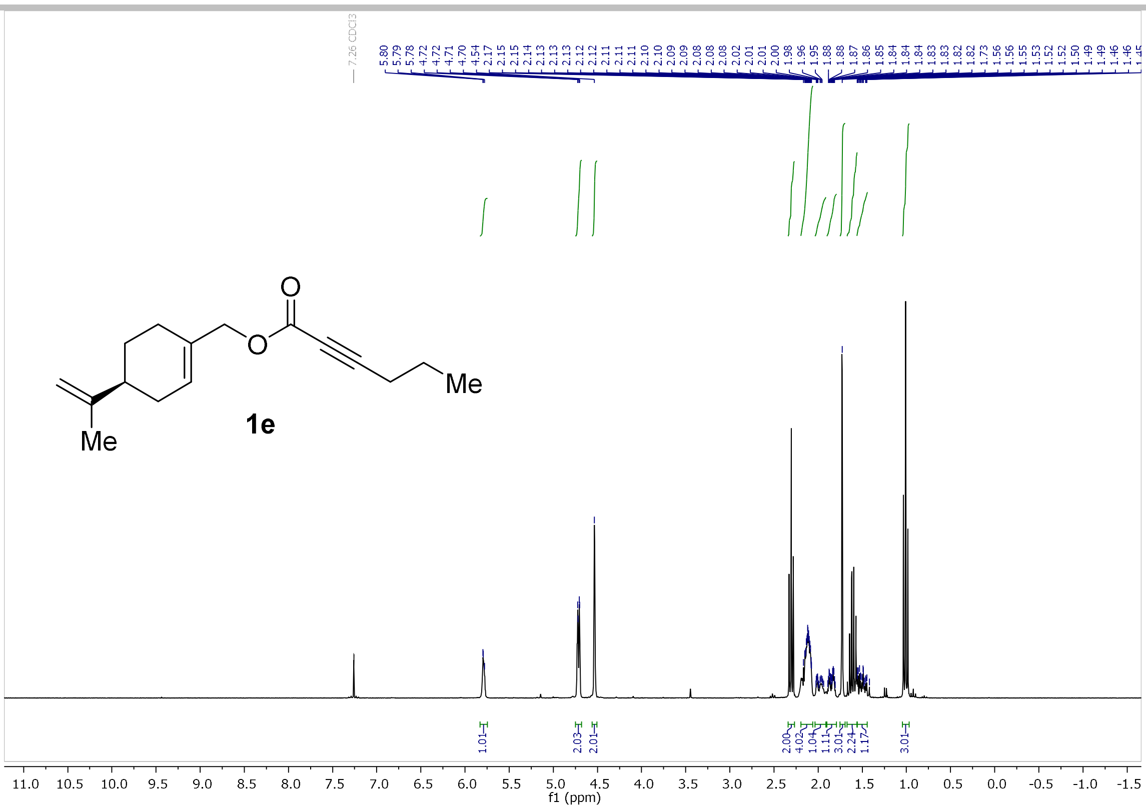
7.26 CDCl3
5.80
5.79
5.78
4.72
4.72
4.71
4.70
4.54
O
Me
Me
1e
1.01
2.03
2.01
2.00
4.02
1.04
1.11
3.01
2.24
1.17
3.01
f1 (ppm)

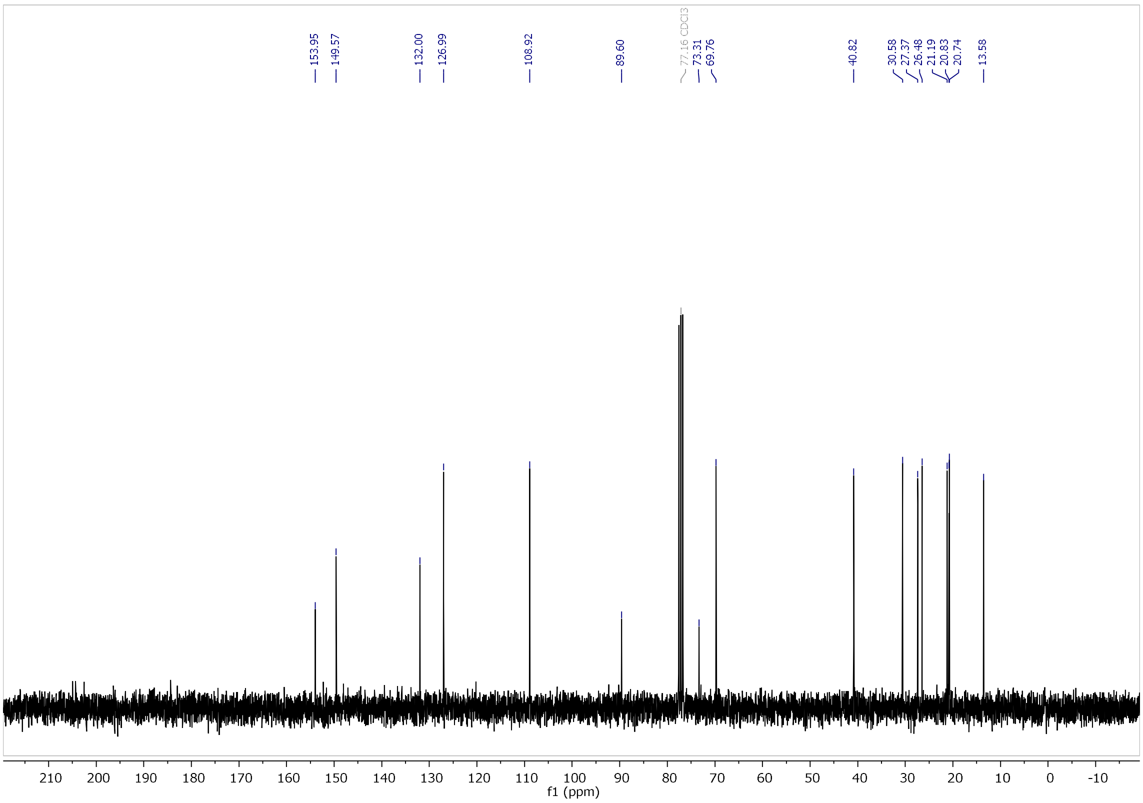
153.95
149.57
132.00
126.99
108.92
89.60
77.16 CDCl3
73.31
69.76
40.82
30.58
27.37
26.48
21.19
20.83
20.74
13.58
f1 (ppm)

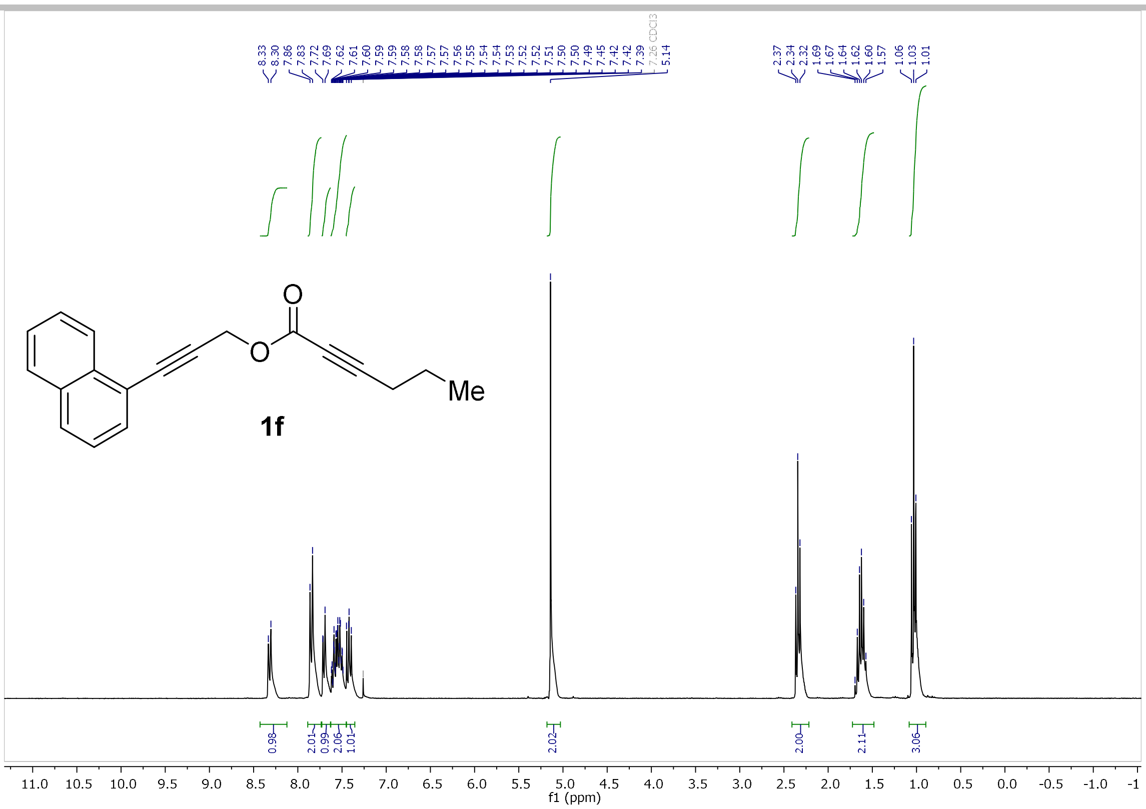
1f
Me
O
O
f1 (ppm)

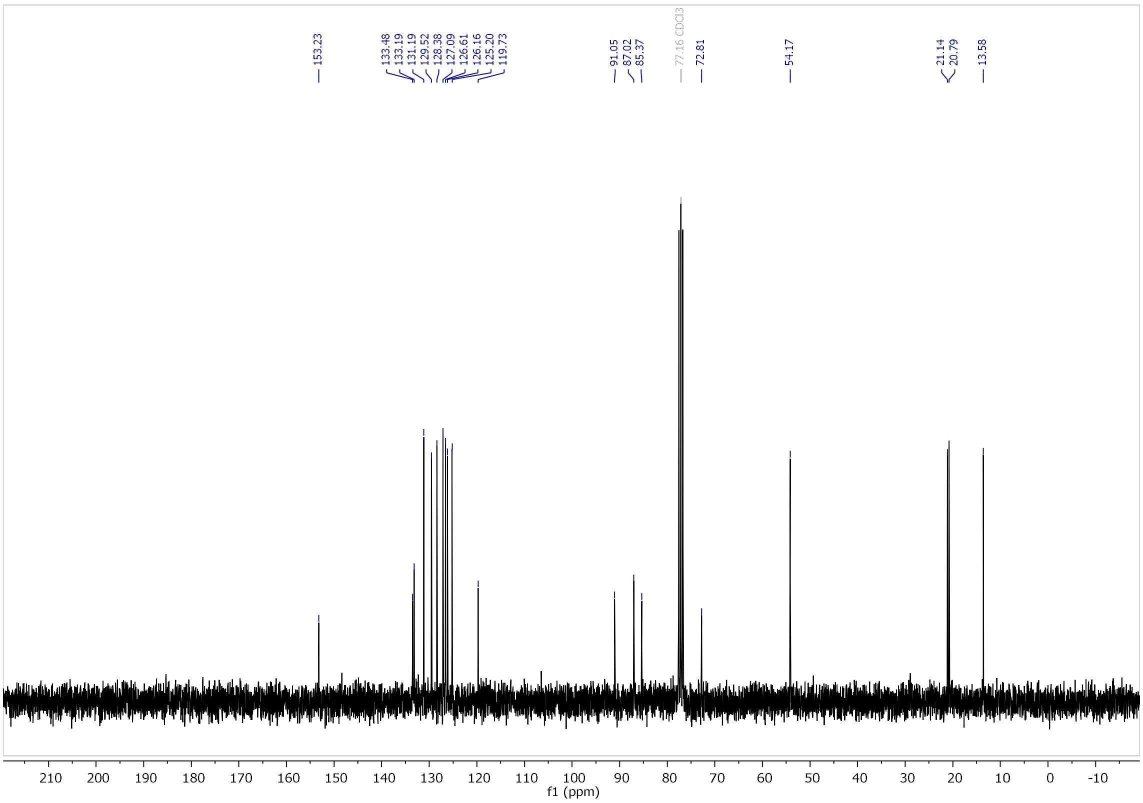
f1 (ppm)

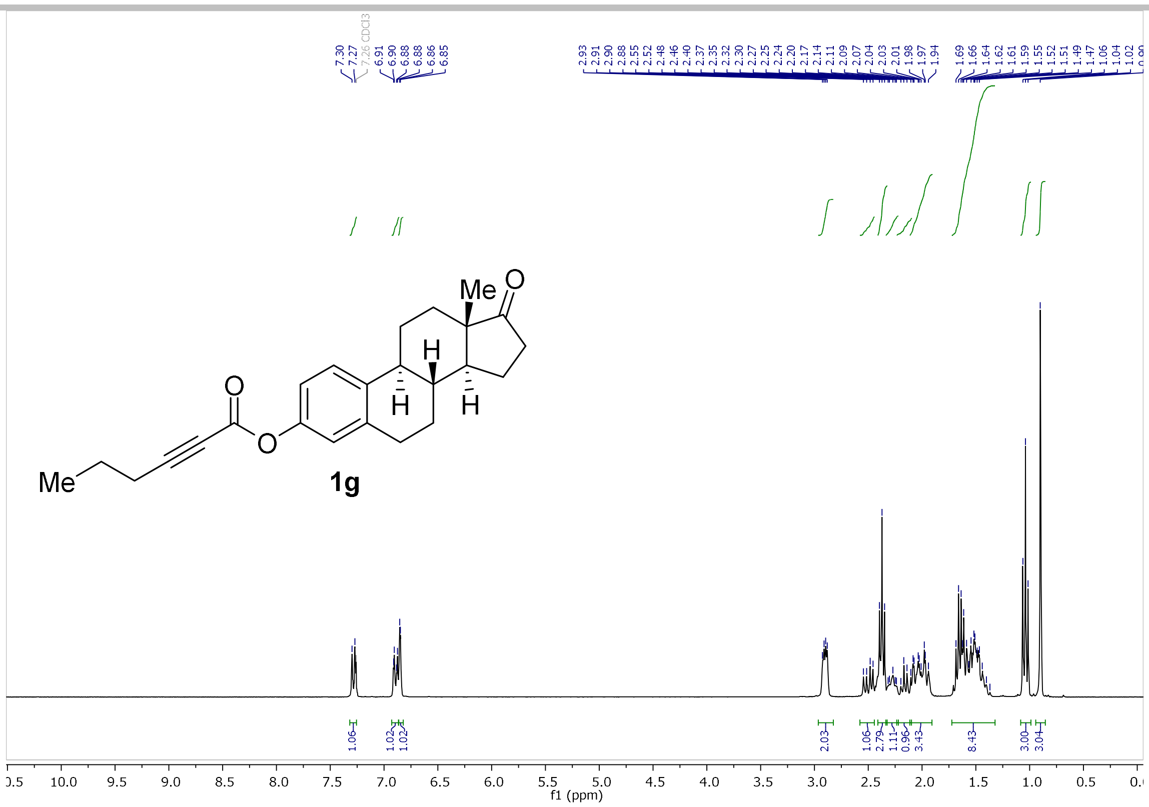
Me
O
H
H
H
O
O
Me
1g
f1 (ppm)

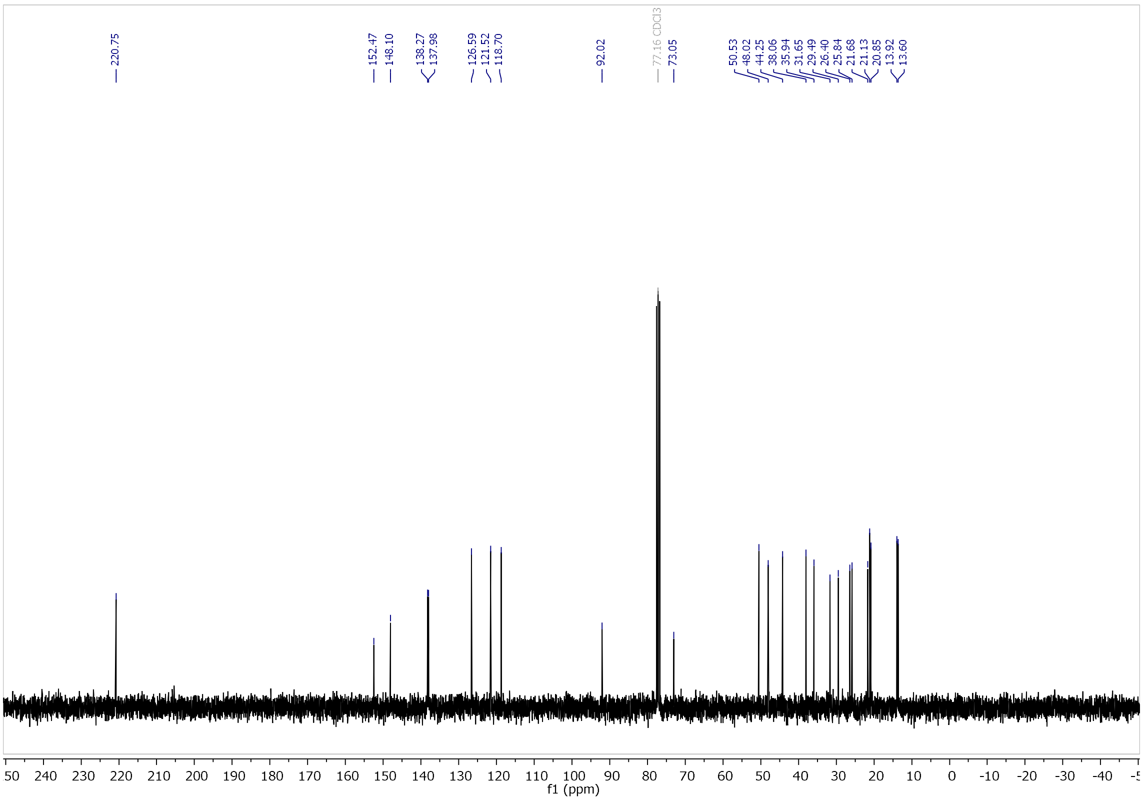
f1 (ppm)

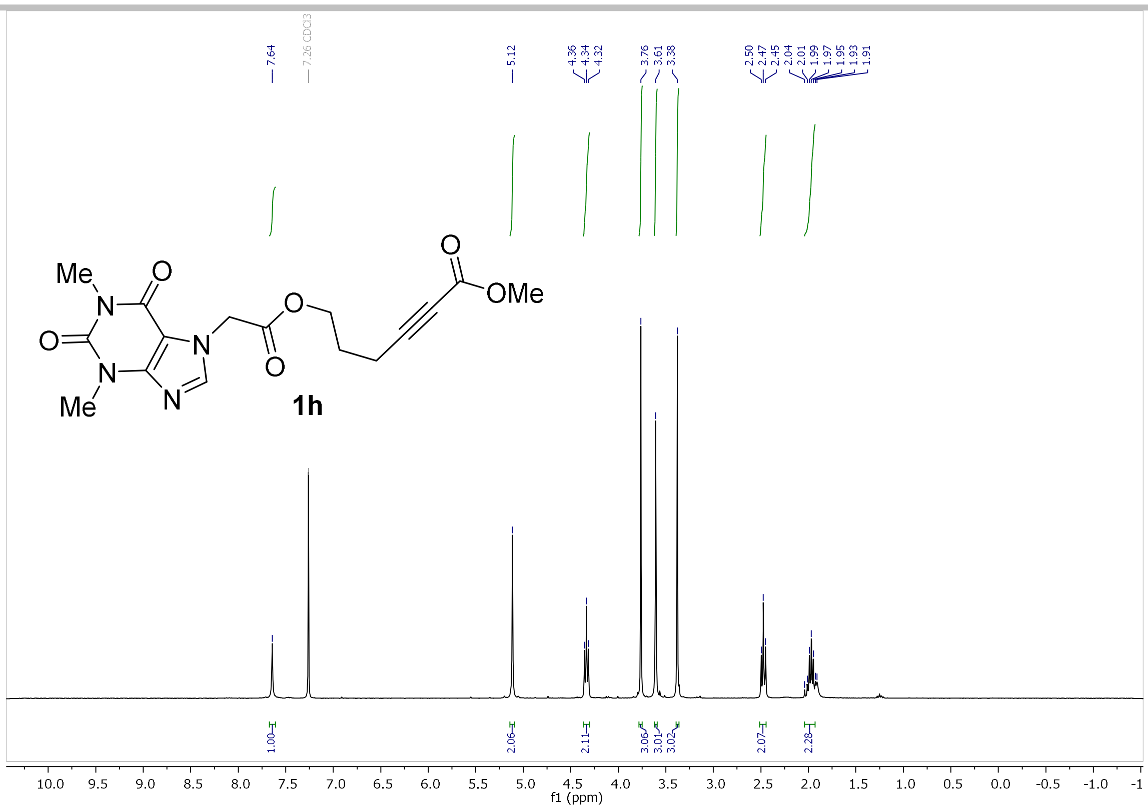
7.64
7.26 CDCl3
5.12
4.36
4.34
4.32
3.76
3.61
3.38
2.50
2.47
2.45
2.04
2.01
1.99
1.97
1.95
1.93
1.91
Me
O
N
O
N
Me
N
N
O
O
O
OMe
1h
1.00
2.06
2.11
3.06
3.01
3.02
2.07
2.28
f1 (ppm)

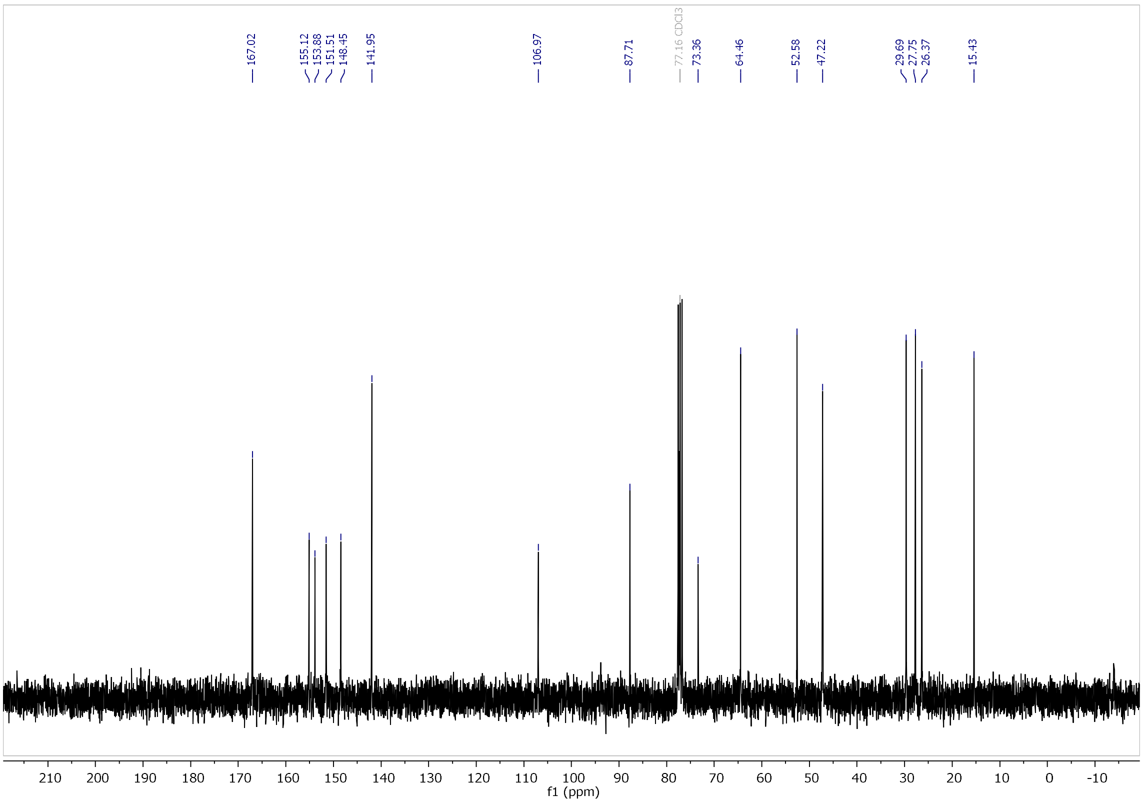
167.02
155.12
153.88
151.51
148.45
141.95
106.97
87.71
77.16 CDCl3
73.36
64.46
52.58
47.22
29.69
27.75
26.37
15.43
f1 (ppm)

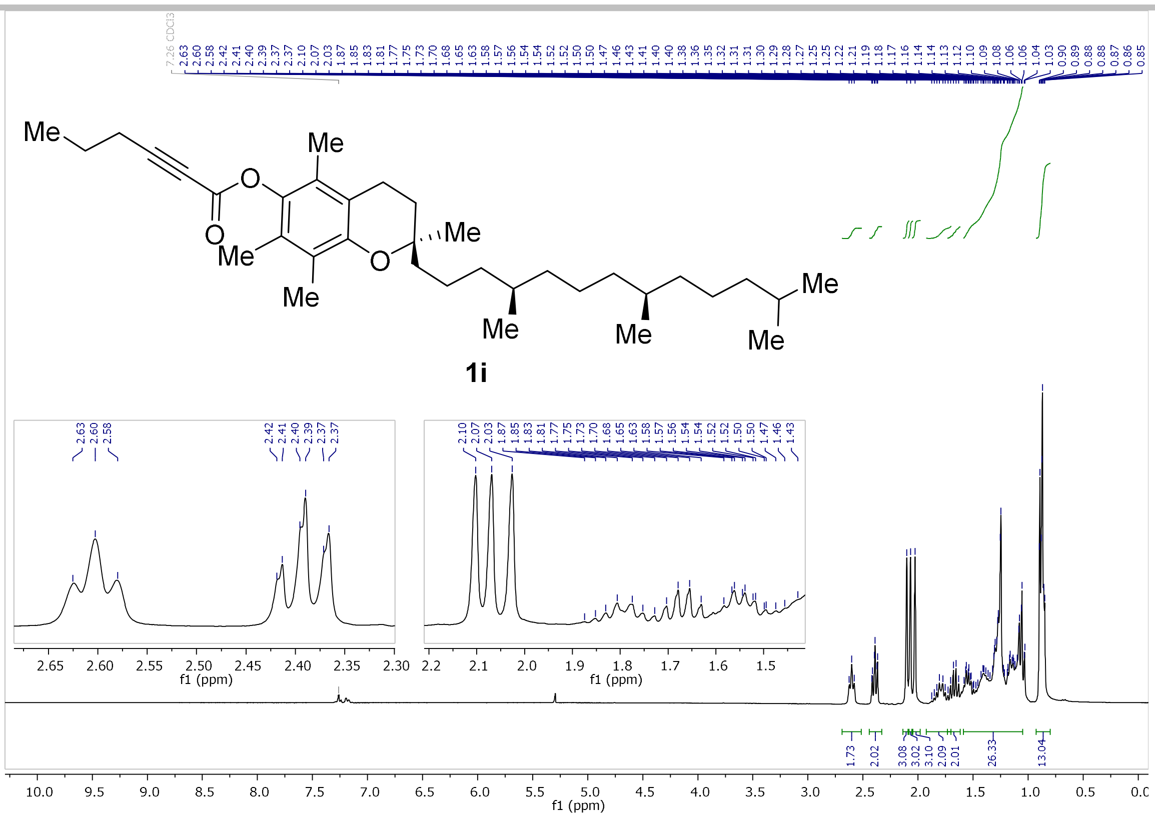
1i
f1 (ppm)

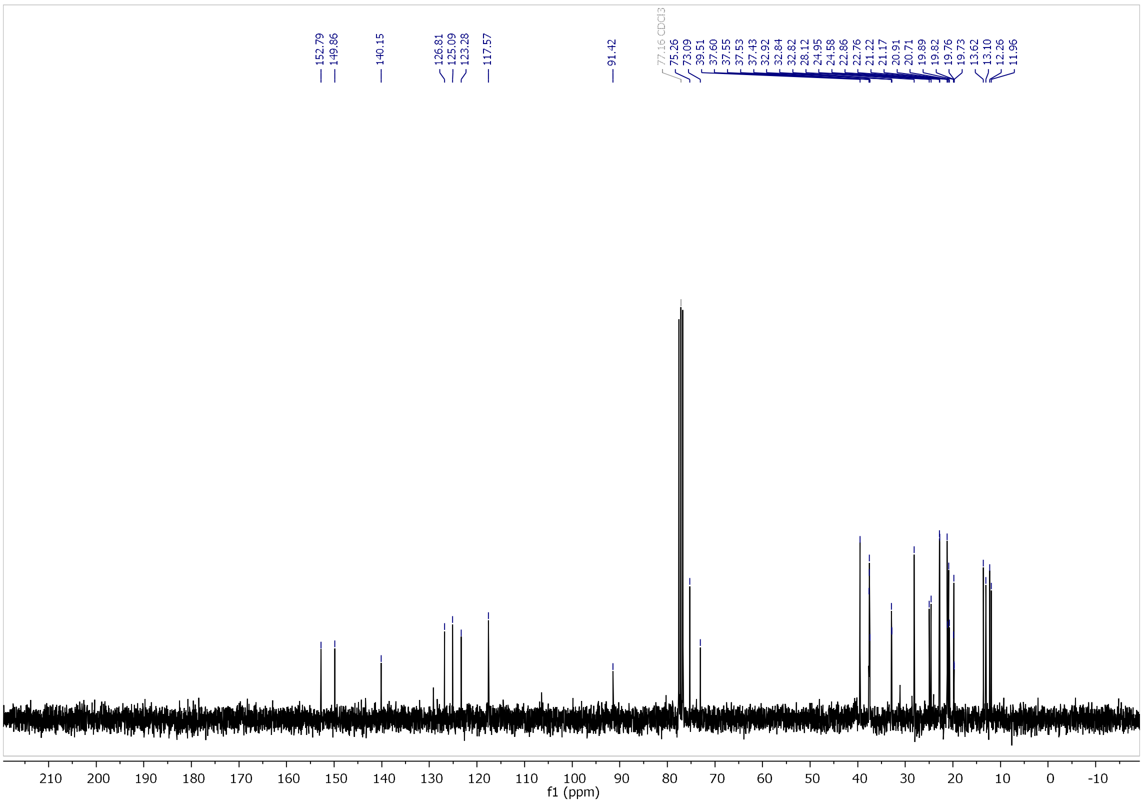
f1 (ppm)

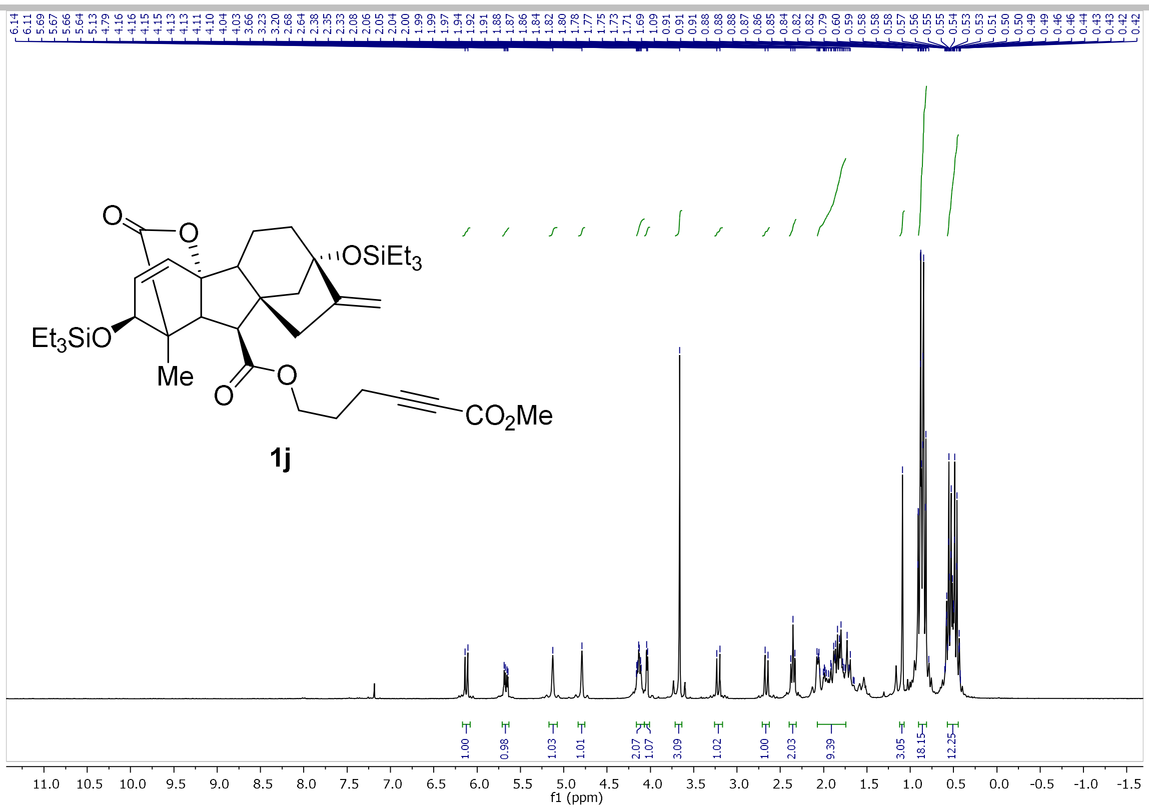
OSiEt3
Et3SiO
Me
CO2Me
1j
f1 (ppm)

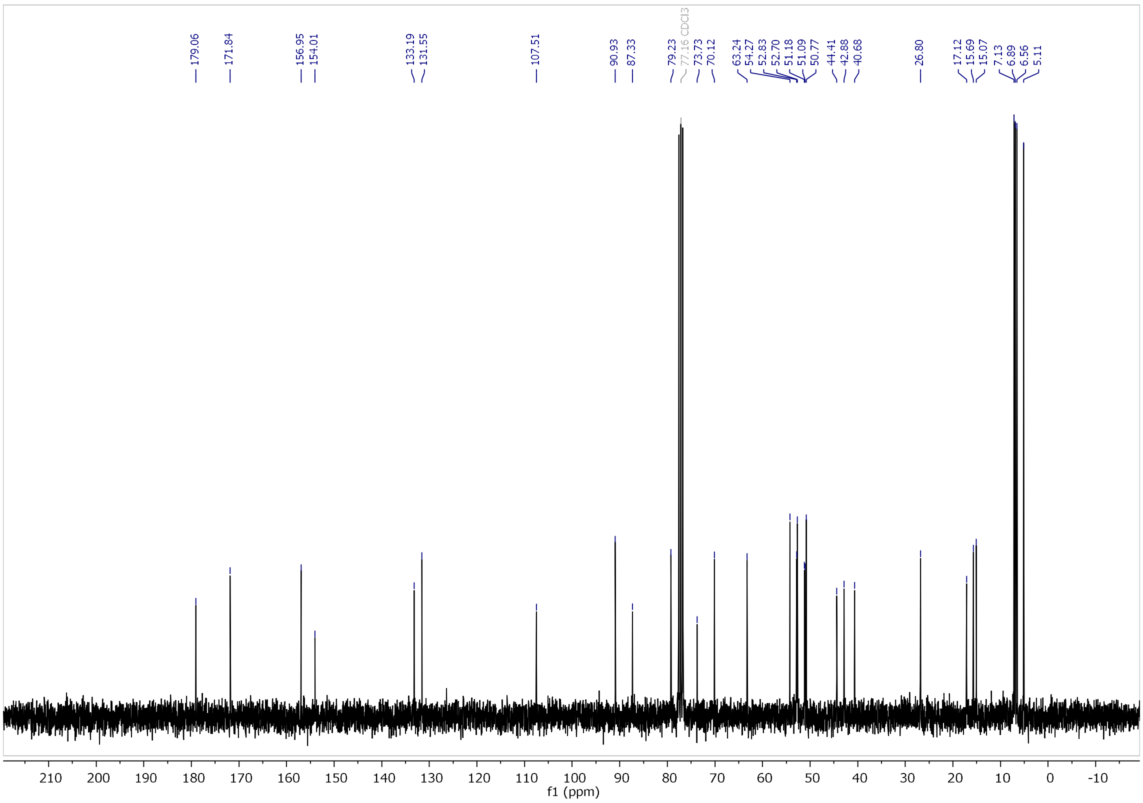
179.06
171.84
156.95
154.01
133.19
131.55
107.51
90.93
87.33
79.23
77.16 CDCl3
73.73
70.12
63.24
54.27
52.83
52.70
51.18
51.09
50.77
44.41
42.88
40.68
26.80
17.12
15.69
15.07
7.13
6.89
6.56
5.11
f1 (ppm)

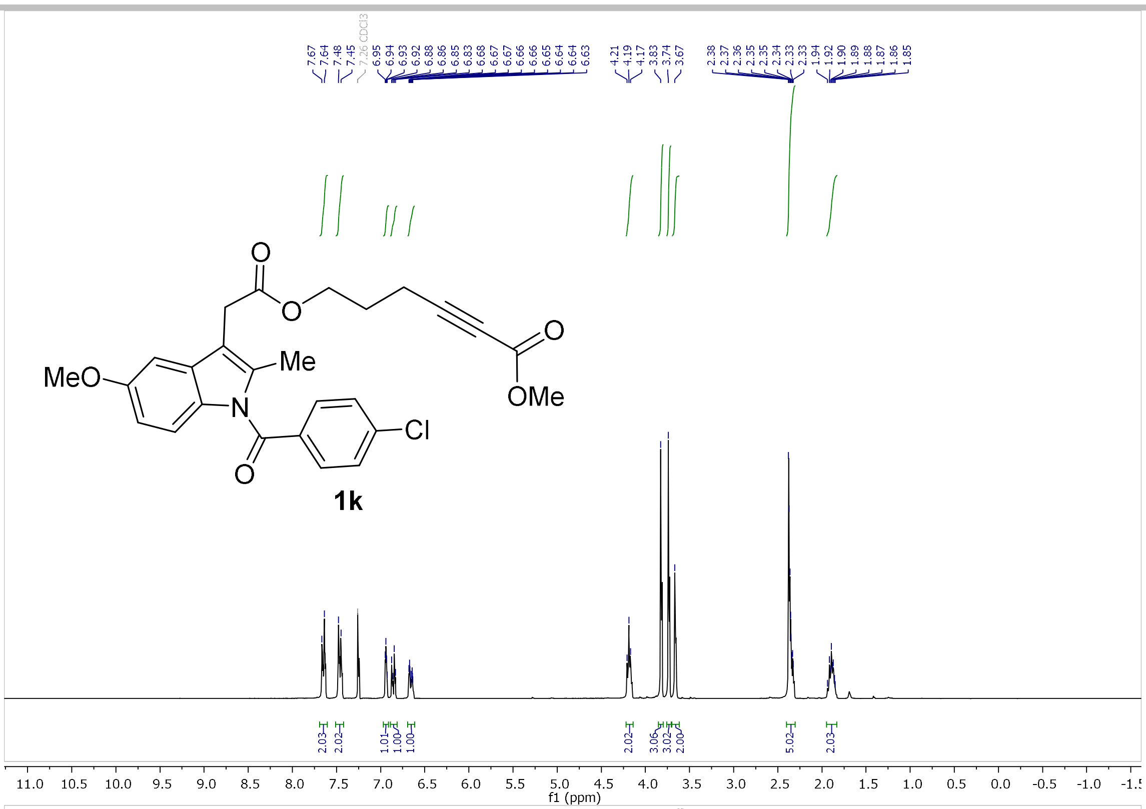
7.67
7.64
7.48
7.45
7.26 CDCl3
6.95
6.94
6.93
6.92
6.88
6.86
6.85
6.83
6.68
6.67
6.67
6.66
6.66
6.65
6.64
6.64
6.63
4.21
4.19
4.17
3.83
3.74
3.67
2.38
2.37
2.36
2.35
2.35
2.34
2.33
2.33
1.94
1.92
1.90
1.89
1.88
1.87
1.86
1.85
O
O
Me
MeO
N
O
OMe
Cl
O
1k
2.03
2.02
1.01
1.00
1.00
2.02
3.06
3.02
2.00
5.02
2.03
11.0
10.5
10.0
9.5
9.0
8.5
8.0
7.5
7.0
6.5
6.0
5.5
5.0
4.5
4.0
3.5
3.0
2.5
2.0
1.5
1.0
0.5
0.0
-0.5
-1.0
f1 (ppm)

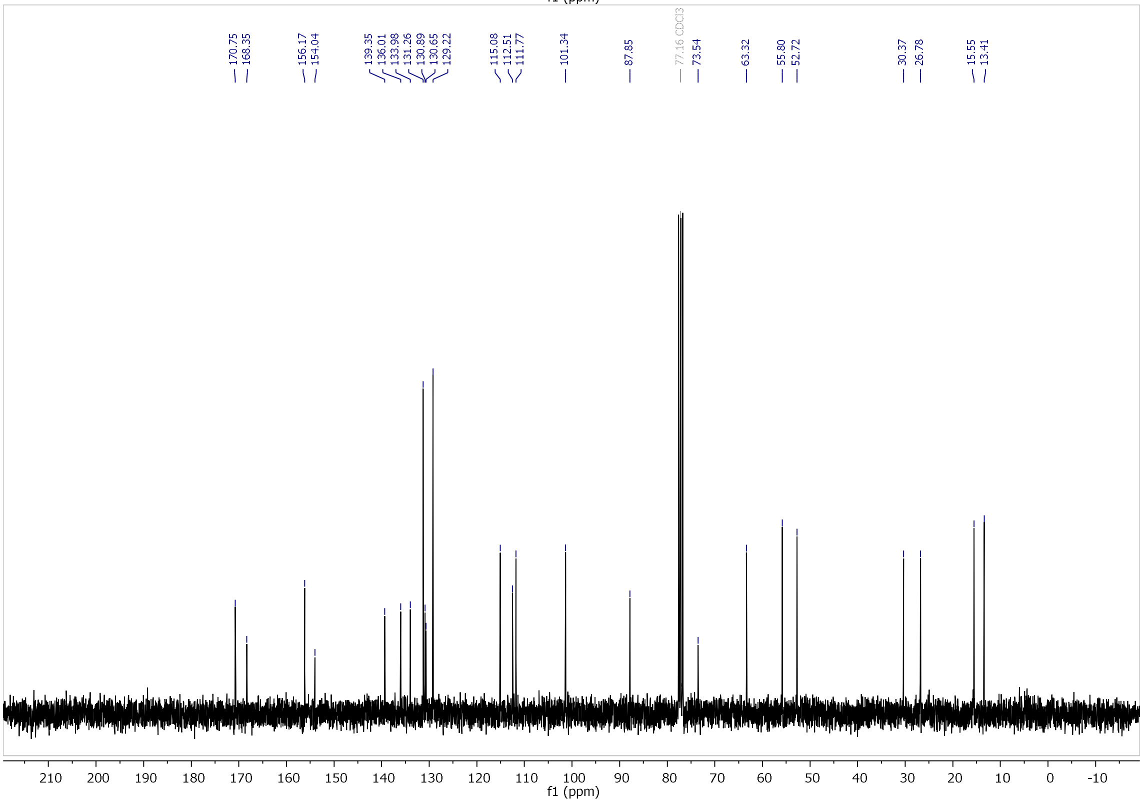
170.75
168.35
156.17
154.04
139.35
136.01
133.98
131.26
130.89
130.65
129.22
115.08
112.51
111.77
101.34
87.85
77.16 CDCl3
73.54
63.32
55.80
52.72
30.37
26.78
15.55
13.41
210
200
190
180
170
160
150
140
130
120
110
100
90
80
70
60
50
40
30
20
10
0
-10
f1 (ppm)

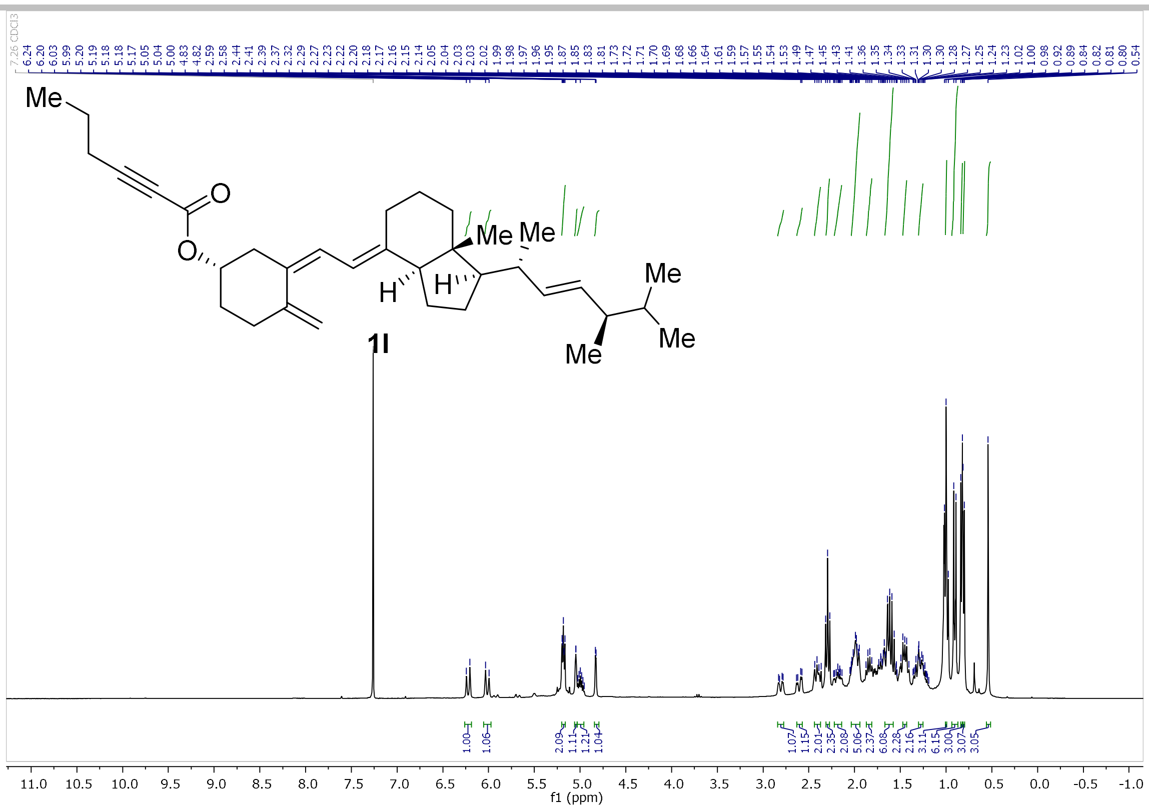

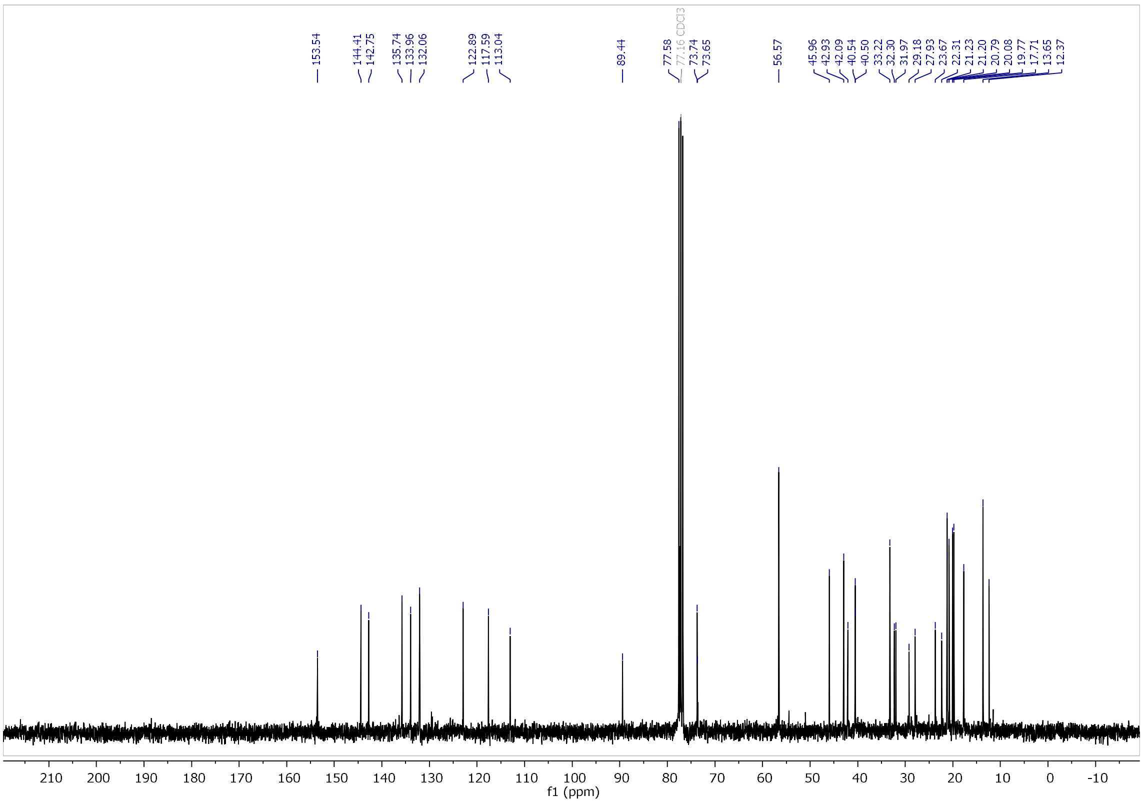

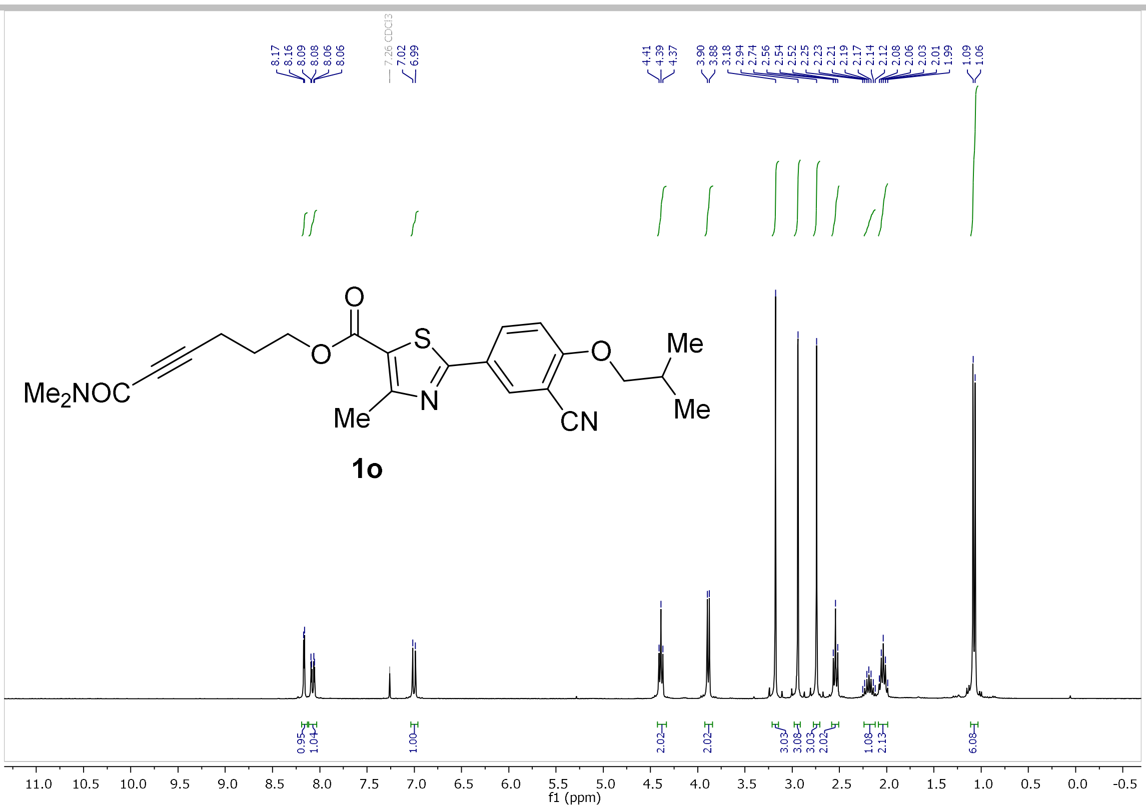

8.17 8.16 8.09 8.08 8.06 8.06
7.26 CDCl3
7.02 6.99
4.41 4.39 4.37
3.90 3.88 3.18 2.94 2.74 2.56 2.54 2.52 2.25 2.23 2.21 2.19 2.17 2.14 2.12 2.08 2.06 2.03 2.01 1.99
1.09 1.06
Me2NOC
O
S
N
Me
CN
1o
0.95 1.04 1.00 2.02 2.02 3.03 3.08 3.03 2.02 1.08 2.13 6.08
f1 (ppm)

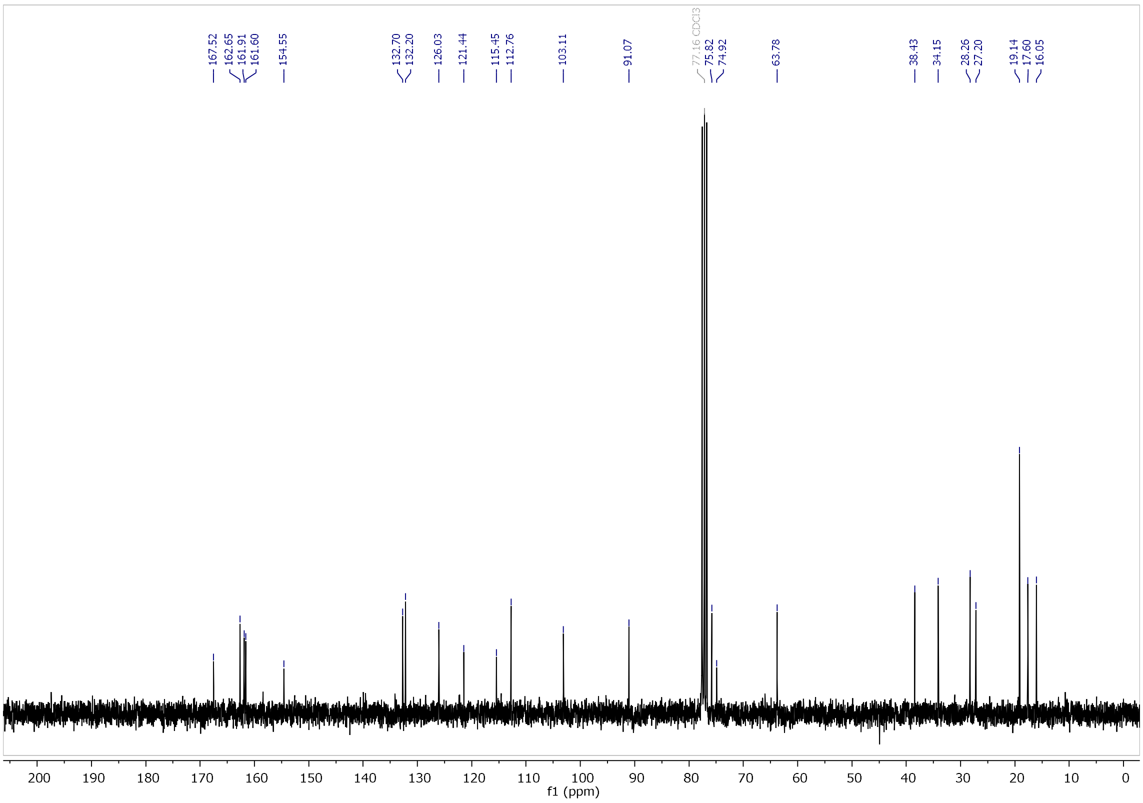

167.52 162.65 161.91 161.60 154.55
132.70 132.20 126.03 121.44 115.45 112.76 103.11 91.07
77.16 CDCl3
75.82 74.92 63.78
38.43 34.15 28.26 27.20 19.14 17.60 16.05
f1 (ppm)

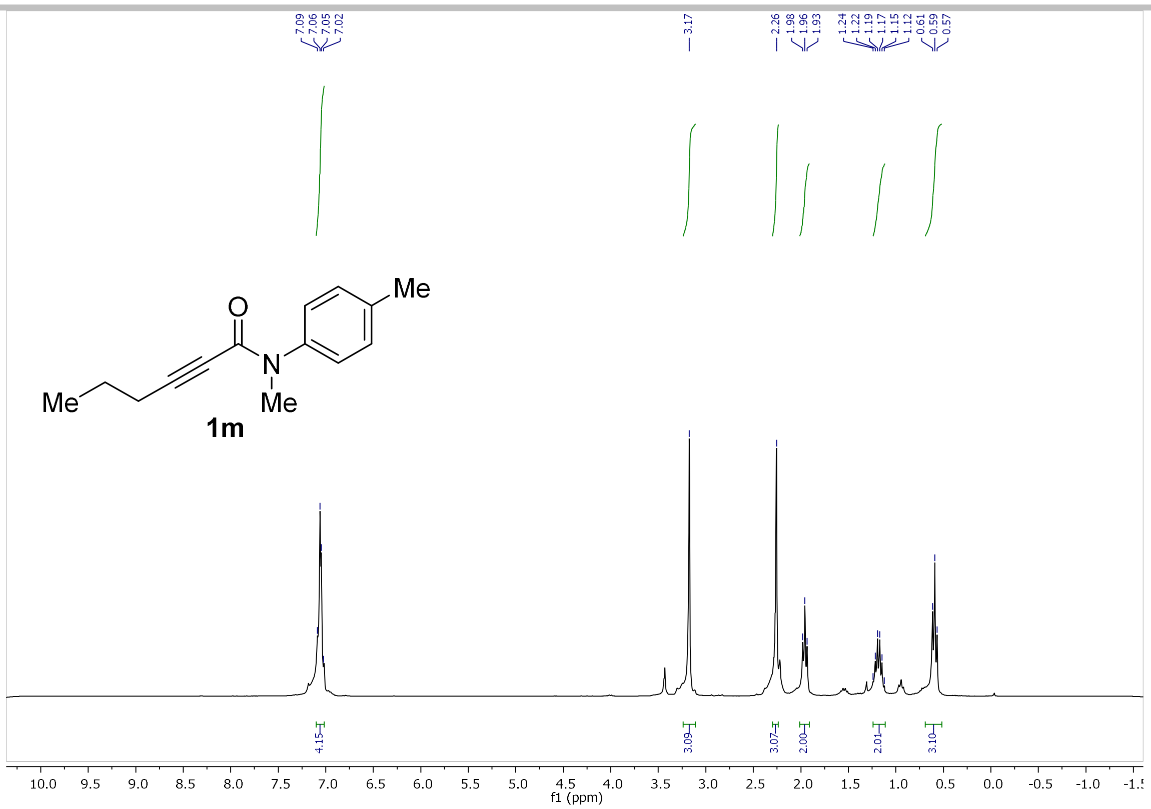
7.09
7.06
7.05
7.02
3.17
2.26
1.98
1.96
1.93
1.24
1.22
1.19
1.17
1.15
1.12
0.61
0.59
0.57
O
N
Me
Me
Me
1m
4.15
3.09
3.07
2.00
2.01
3.10
f1 (ppm)

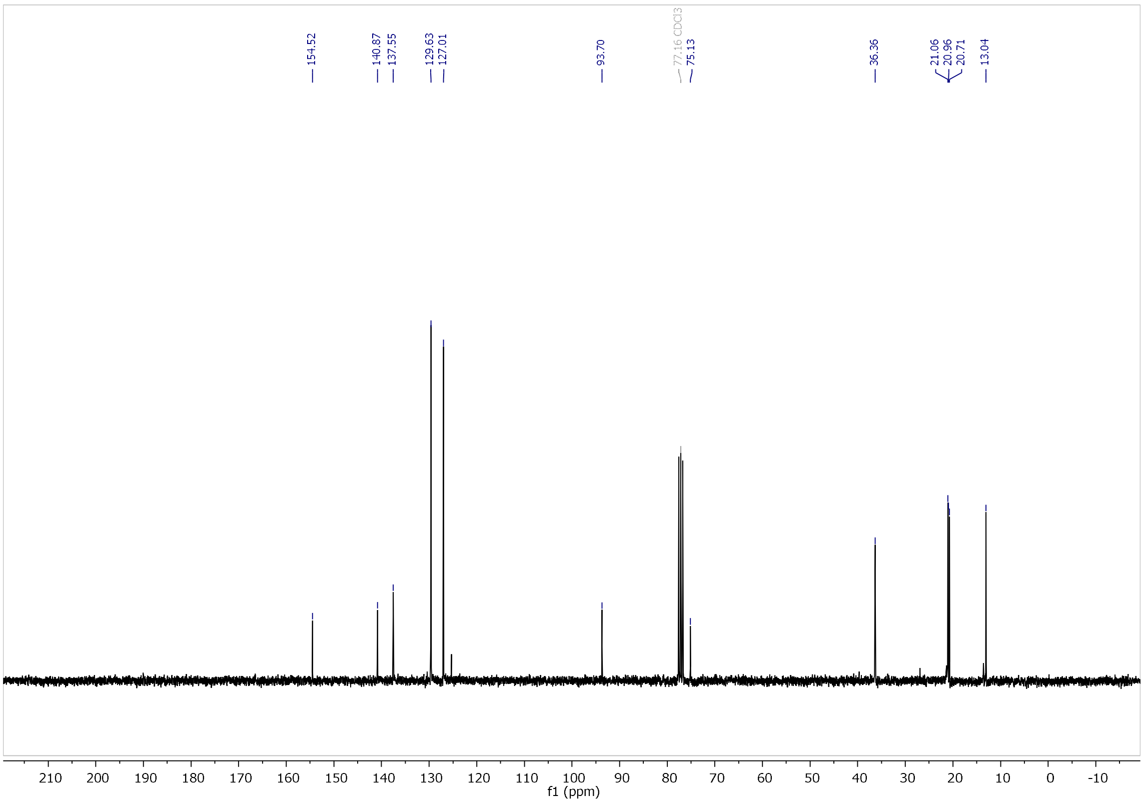
154.52
140.87
137.55
129.63
127.01
93.70
77.16 CDCl3
75.13
36.36
21.06
20.96
20.71
13.04
f1 (ppm)

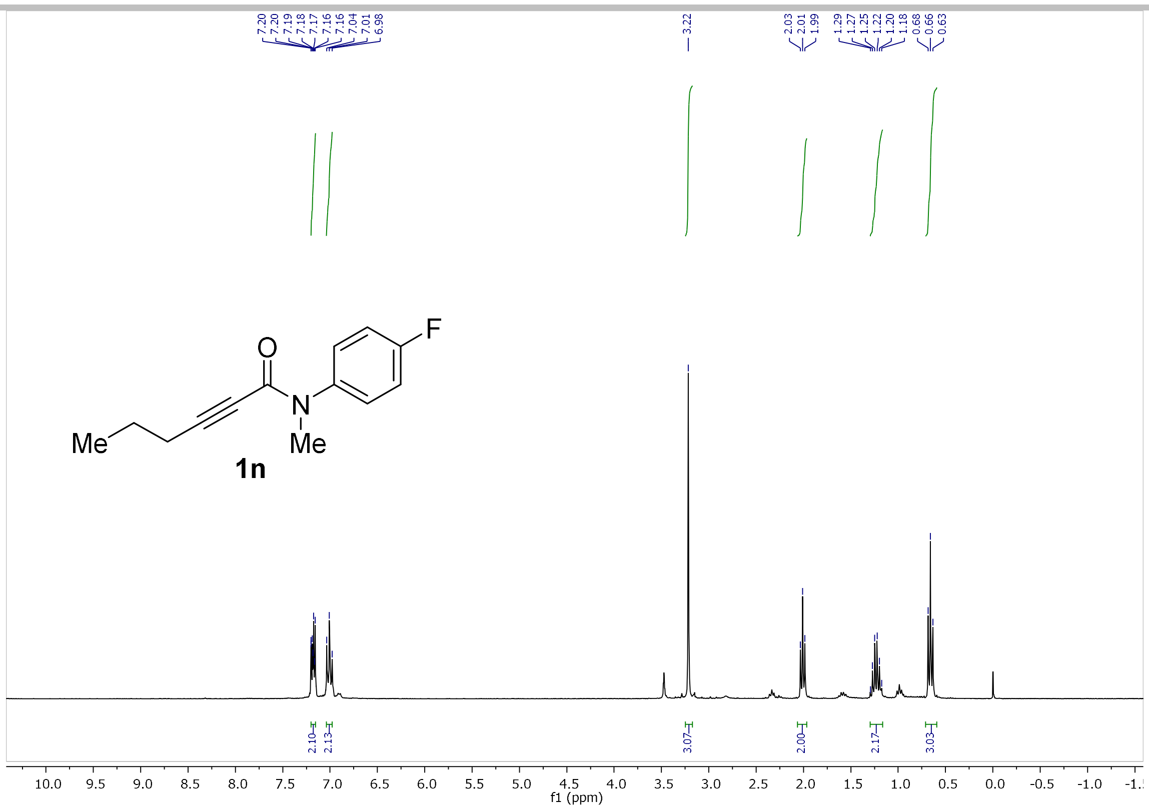

7.20
7.20
7.19
7.18
7.17
7.16
7.16
7.04
7.01
6.98
3.22
2.03
2.01
1.99
1.29
1.27
1.25
1.22
1.20
1.18
0.68
0.66
0.63
1n
2.10
2.13
3.07
2.00
2.17
3.03
f1 (ppm)

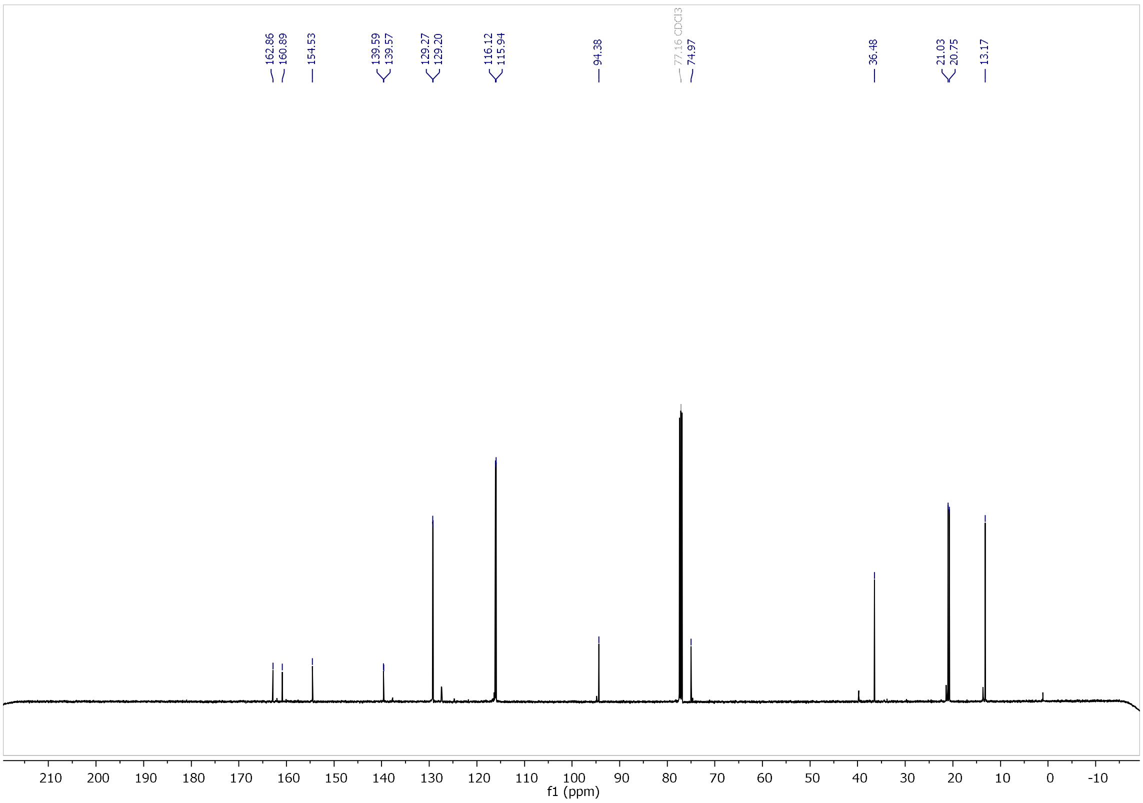

162.86
160.89
154.53
139.59
139.57
129.27
129.20
116.12
115.94
94.38
77.16 CDCl3
74.97
36.48
21.03
20.75
13.17
f1 (ppm)

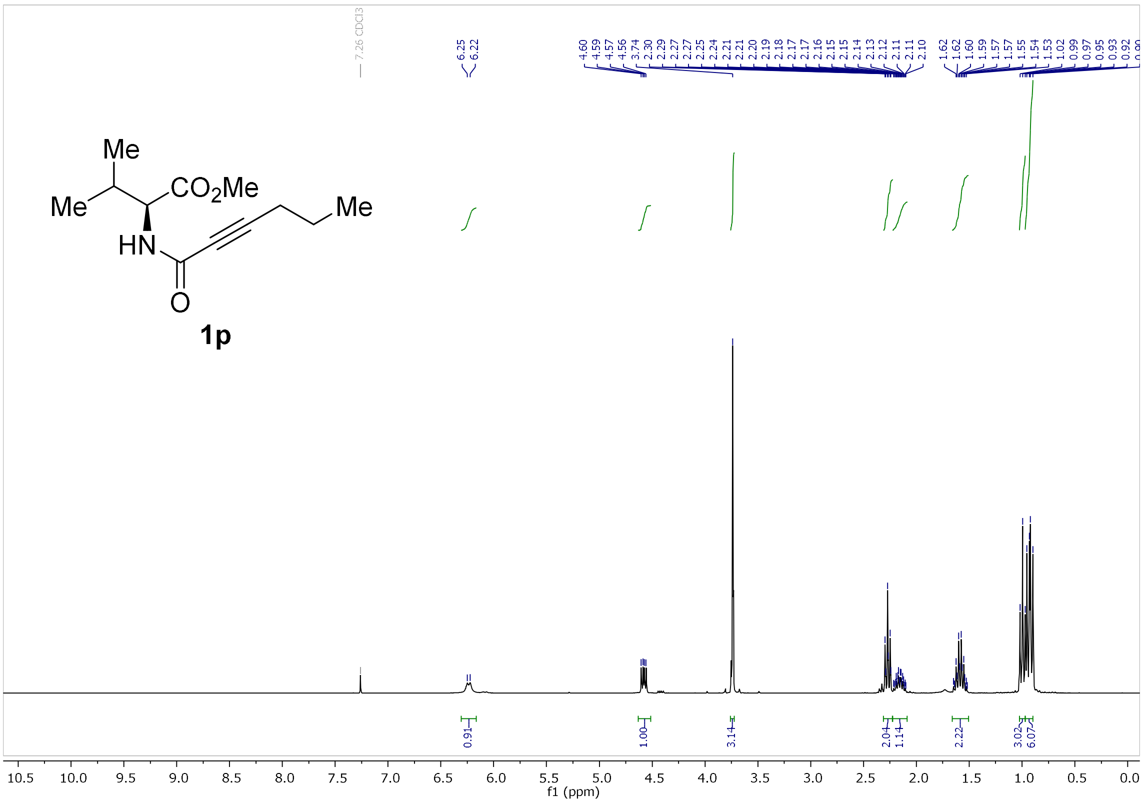
Me
CO2Me
Me
HN
Me
O
1p
7.26 CDCl3
f1 (ppm)

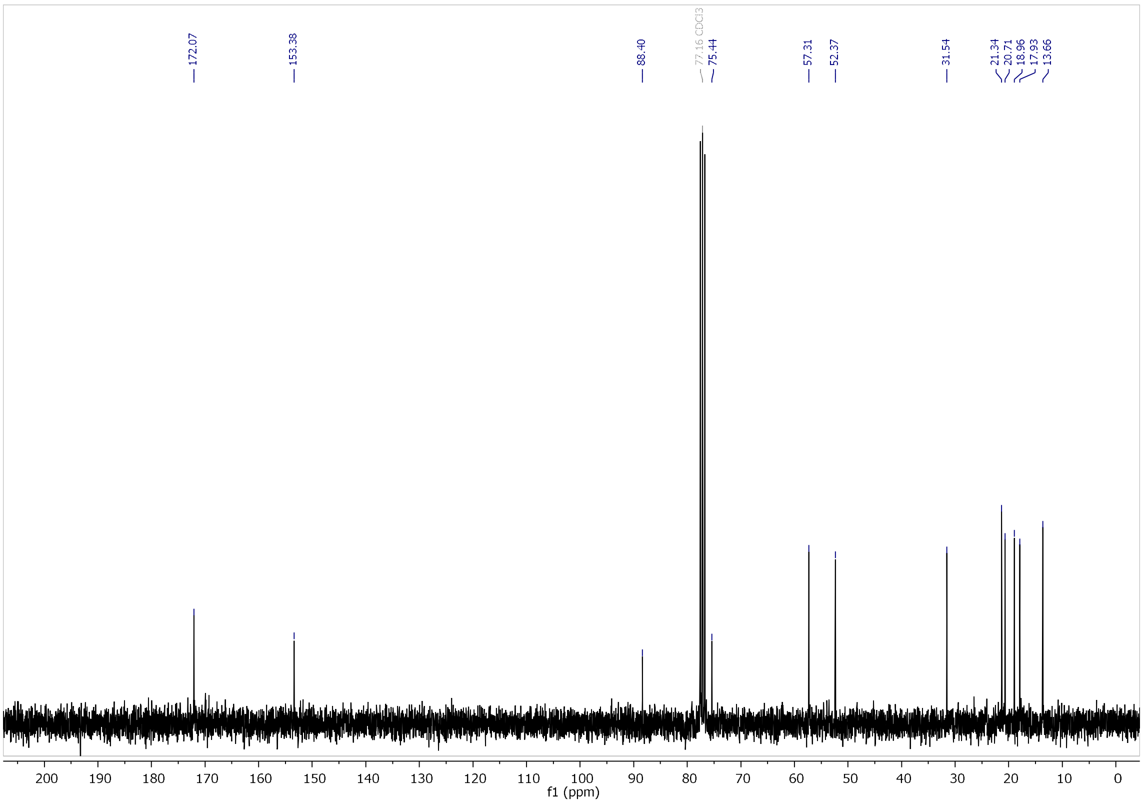
172.07
153.38
88.40
77.16 CDCl3
75.44
57.31
52.37
31.54
21.34
20.71
18.96
17.93
13.66
f1 (ppm)

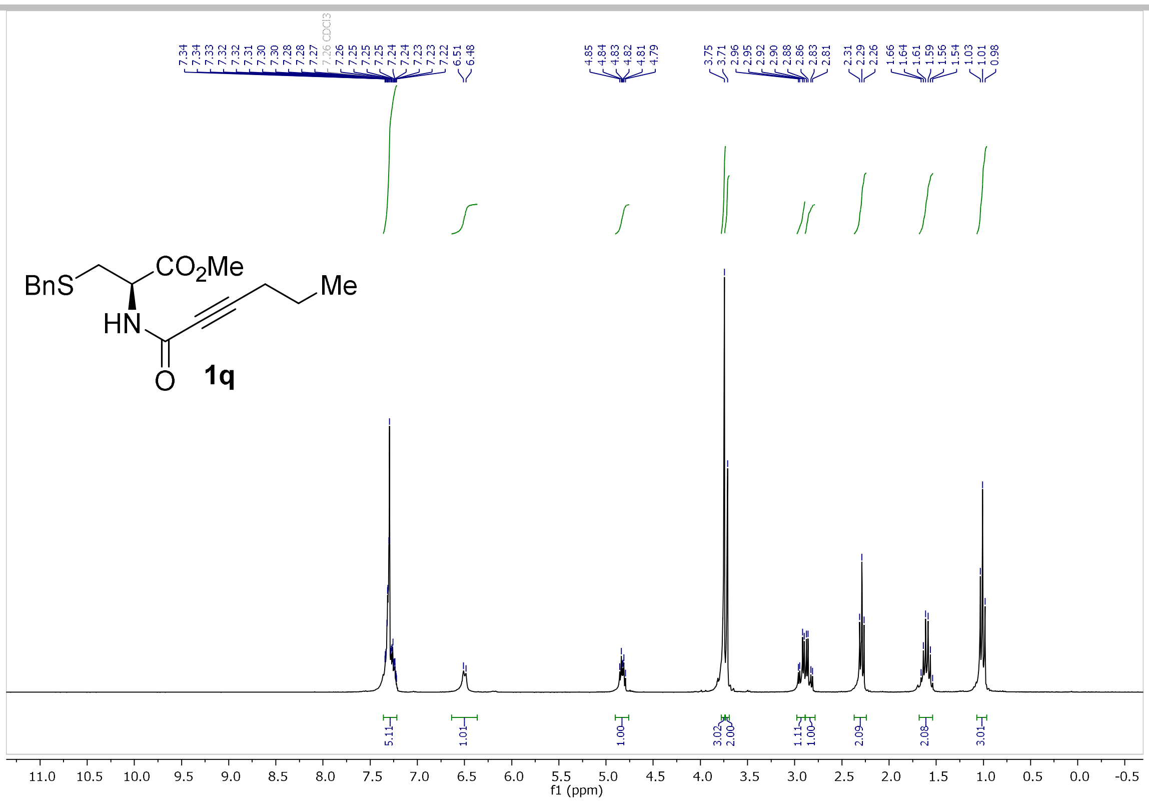

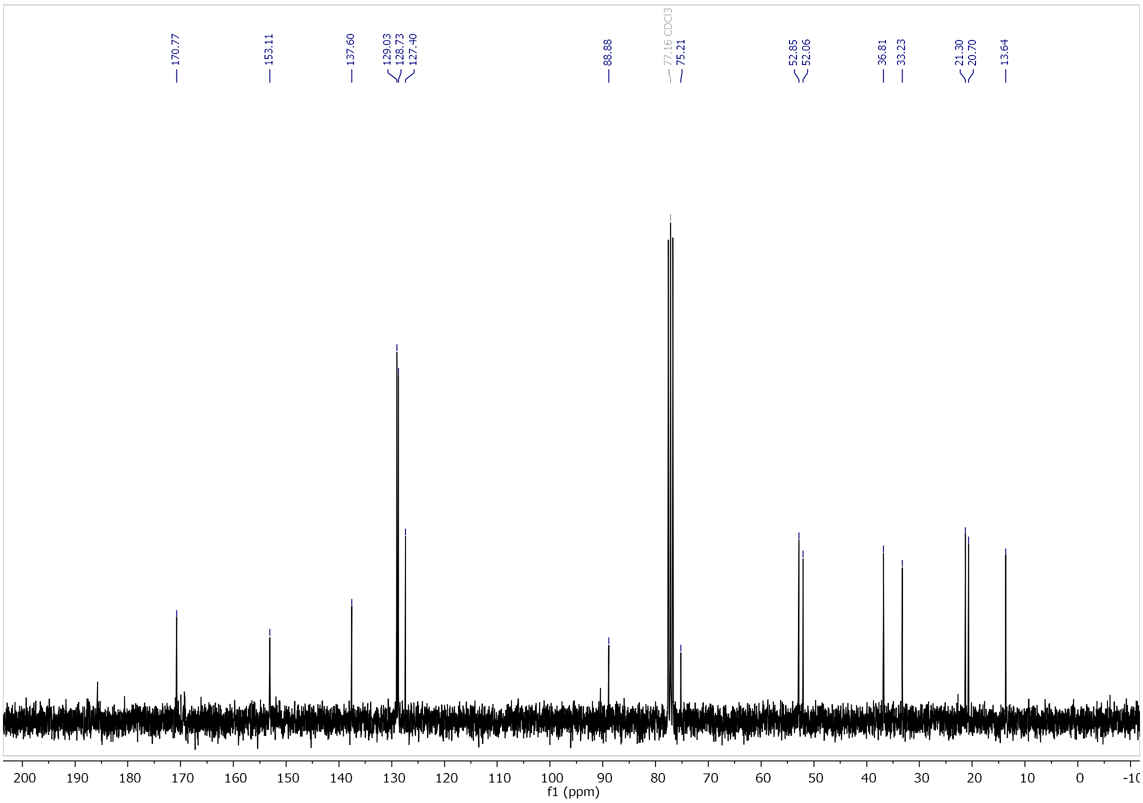

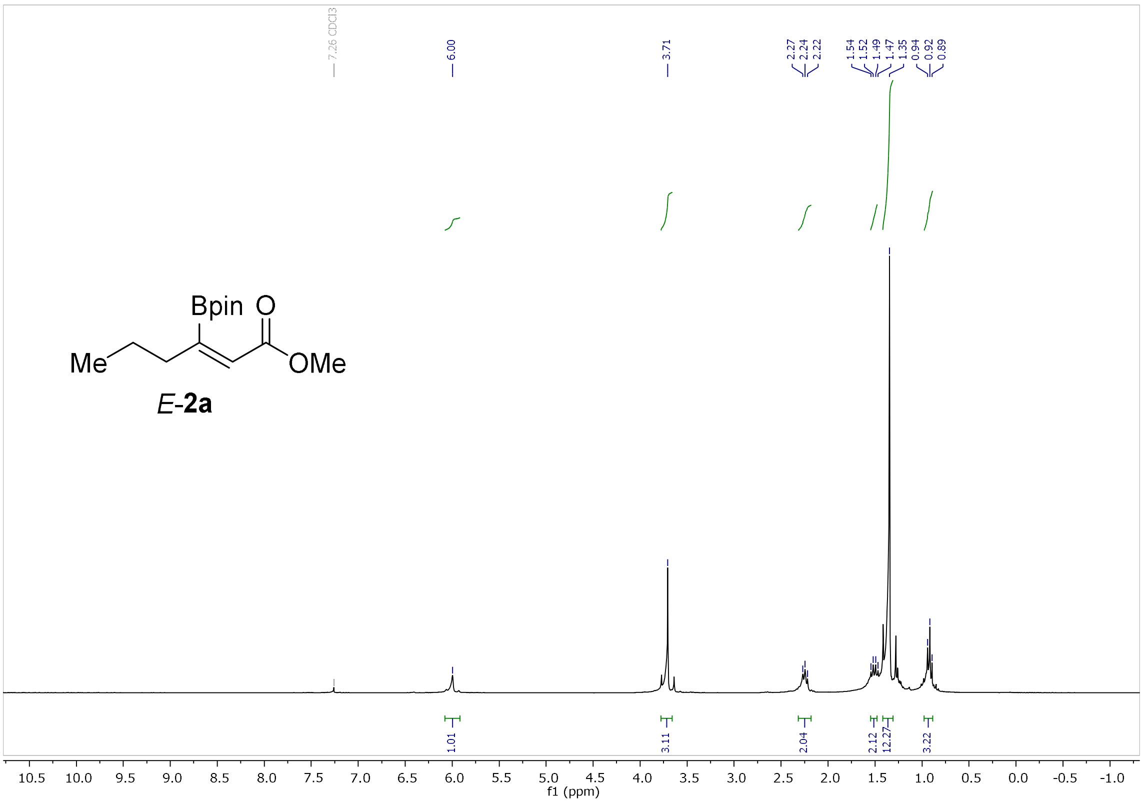
7.26 CDCl3
6.00
3.71
2.27
2.24
2.22
1.54
1.52
1.49
1.47
1.35
0.94
0.92
0.89
Bpin O
Me
OMe
E-2a
1.01
3.11
2.04
2.12
12.27
3.22
10.5 10.0 9.5 9.0 8.5 8.0 7.5 7.0 6.5 6.0 5.5 5.0 4.5 4.0 3.5 3.0 2.5 2.0 1.5 1.0 0.5 0.0 -0.5 -1.0
f1 (ppm)

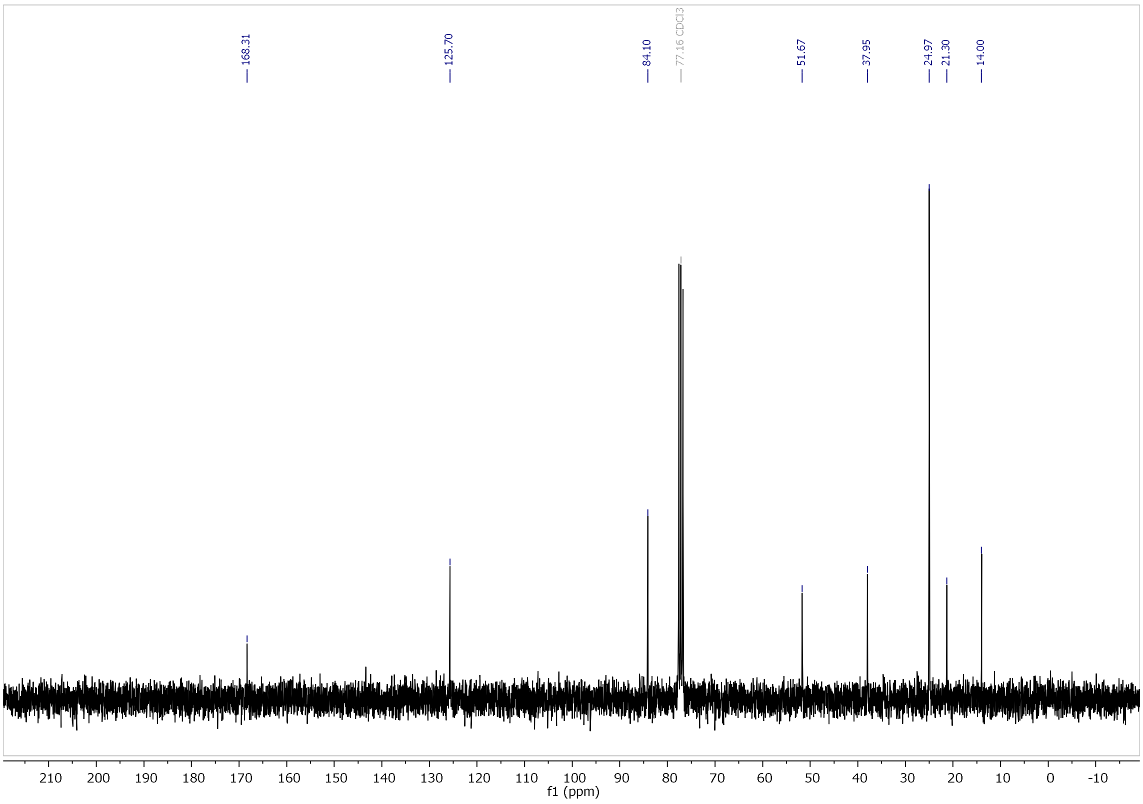
168.31
125.70
84.10
77.16 CDCl3
51.67
37.95
24.97
21.30
14.00
210 200 190 180 170 160 150 140 130 120 110 100 90 80 70 60 50 40 30 20 10 0 -10
f1 (ppm)

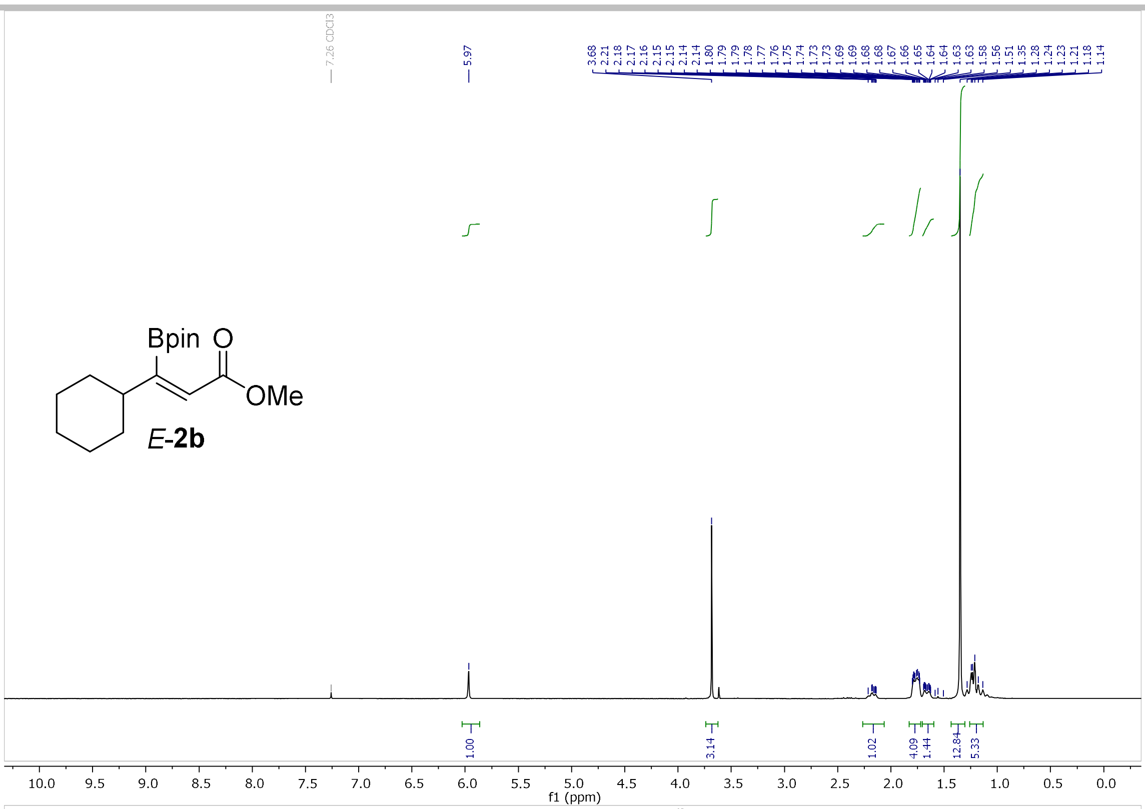
7.26 CDCl3
5.97
3.68
2.21
2.18
2.17
2.16
2.15
2.15
2.14
2.14
1.80
1.79
1.79
1.78
1.77
1.76
1.75
1.74
1.73
1.73
1.69
1.69
1.68
1.68
1.67
1.66
1.65
1.64
1.64
1.63
1.63
1.58
1.56
1.51
1.35
1.28
1.24
1.23
1.21
1.18
1.14
Bpin
O
OMe
E-2b
1.00
3.14
1.02
4.09
1.44
12.84
5.33
10.0 9.5 9.0 8.5 8.0 7.5 7.0 6.5 6.0 5.5 5.0 4.5 4.0 3.5 3.0 2.5 2.0 1.5 1.0 0.5 0.0
f1 (ppm)

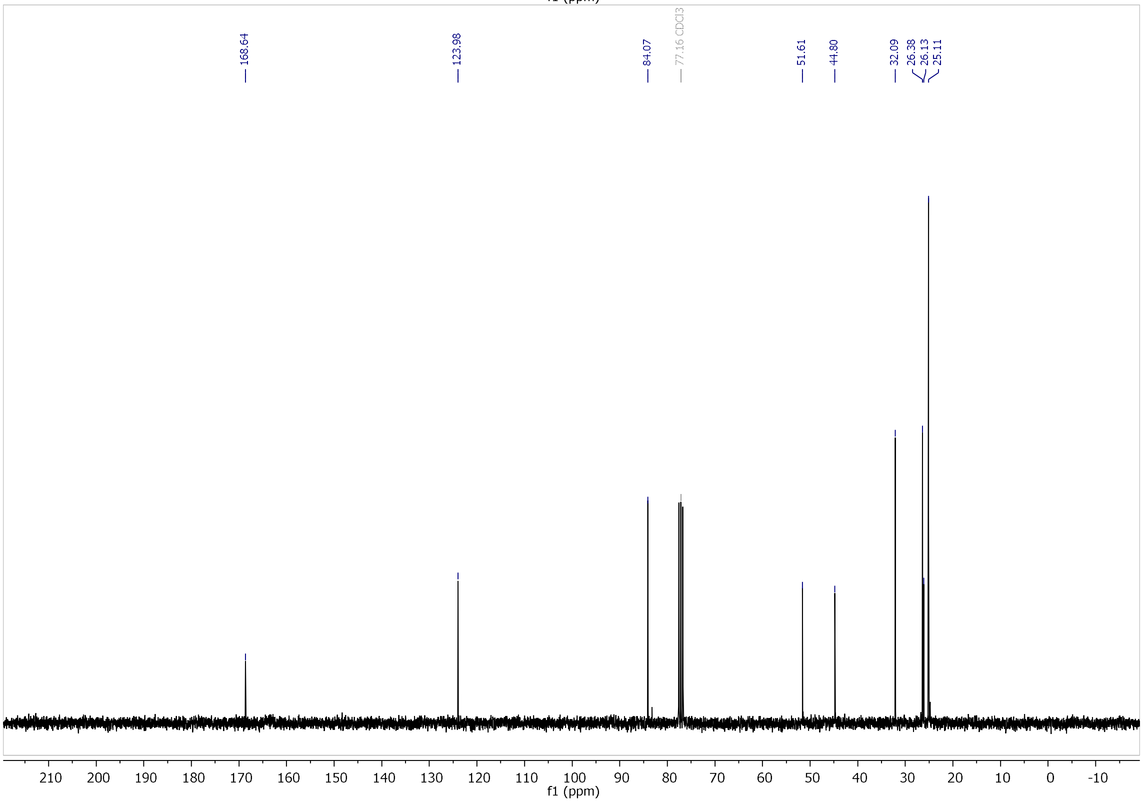
168.64
123.98
84.07
77.16 CDCl3
51.61
44.80
32.09
26.38
26.13
25.11
210 200 190 180 170 160 150 140 130 120 110 100 90 80 70 60 50 40 30 20 10 0 -10
f1 (ppm)

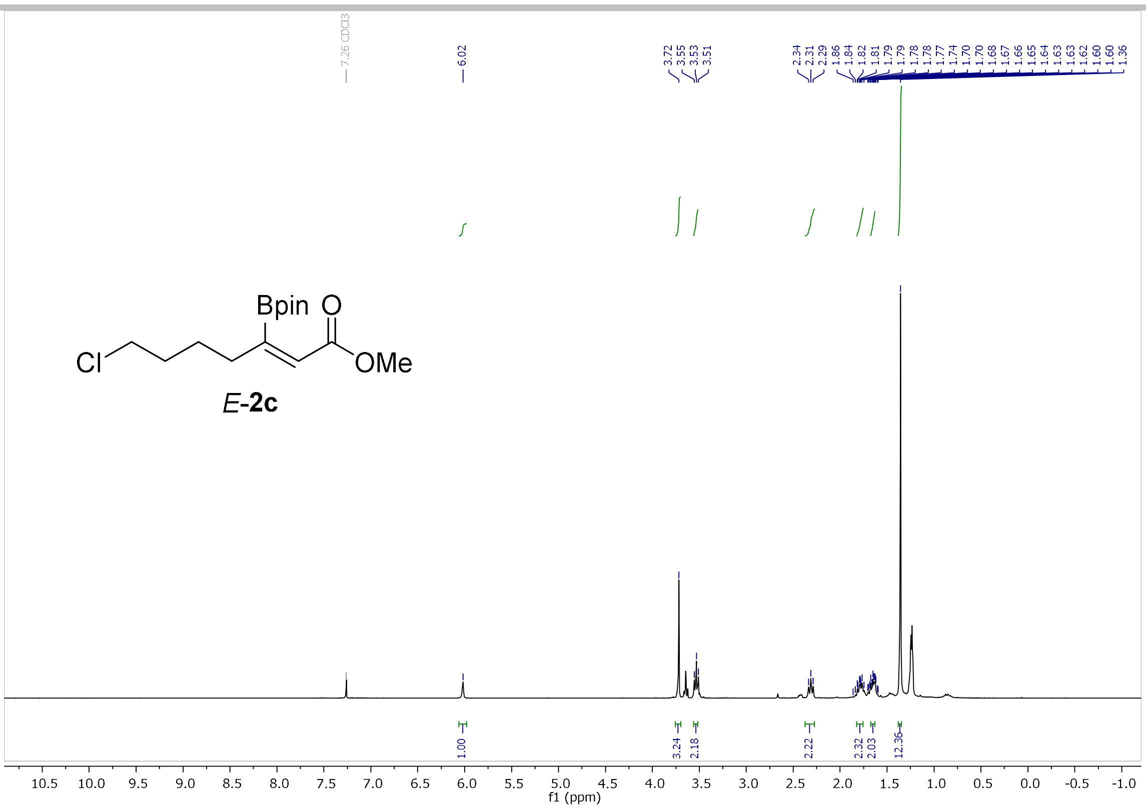
7.26 CDCl3
6.02
3.72
3.55
3.53
3.51
2.34
2.31
2.29
1.86
1.84
1.82
1.81
1.79
1.79
1.78
1.78
1.77
1.74
1.70
1.70
1.68
1.67
1.66
1.65
1.64
1.63
1.63
1.62
1.60
1.60
1.36
Bpin
O
Cl
OMe
E-2c
1.00
3.24
2.18
2.22
2.32
2.03
12.36
f1 (ppm)

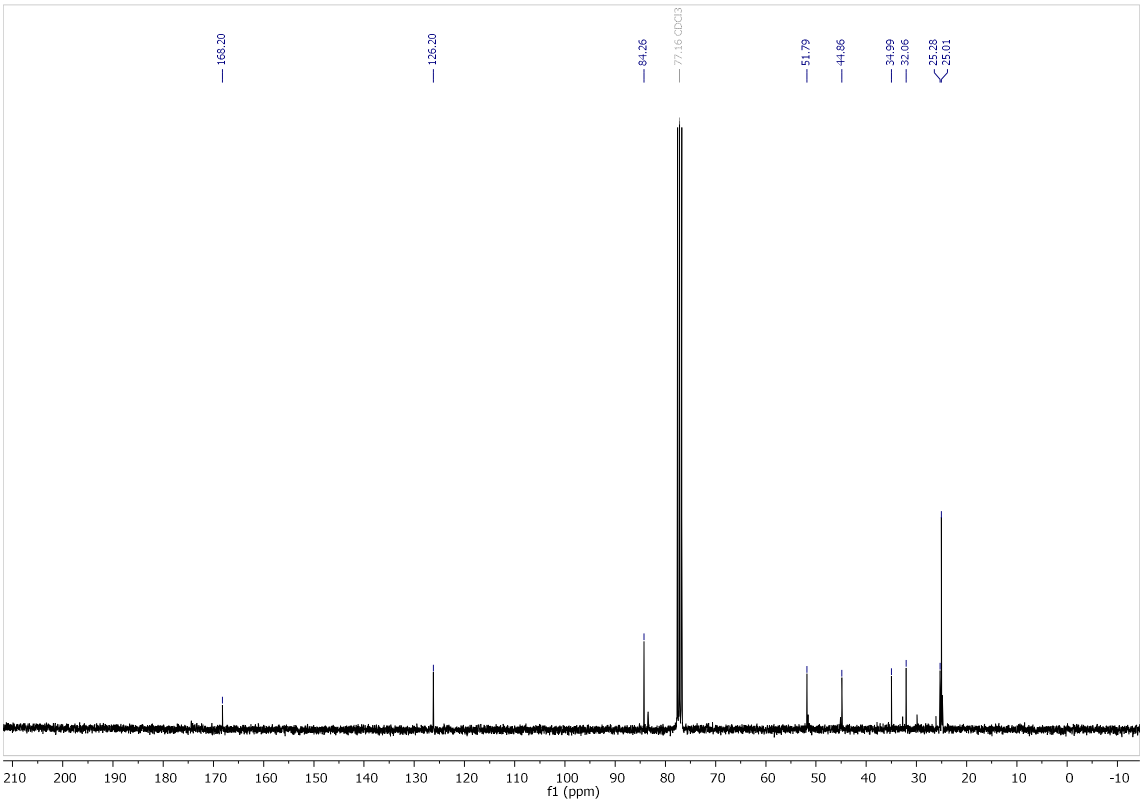
168.20
126.20
84.26
77.16 CDCl3
51.79
44.86
34.99
32.06
25.28
25.01
f1 (ppm)

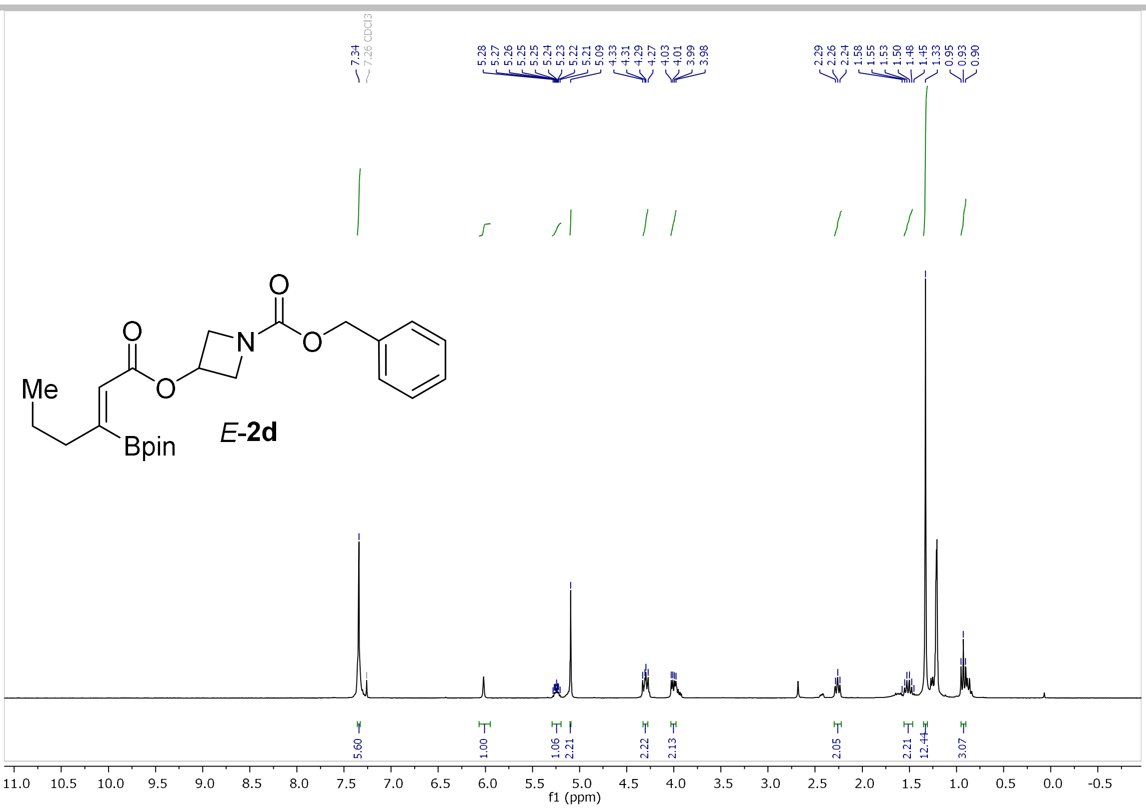
7.34
7.26 CDCl3
5.28 5.27 5.26 5.25 5.25 5.24 5.23 5.22 5.21 5.09 4.33 4.31 4.29 4.27 4.03 4.01 3.99 3.98
2.29 2.26 2.24 1.58 1.55 1.53 1.50 1.48 1.45 1.33 0.95 0.93 0.90
Me
Bpin
O
N
E-2d
5.60 1.00 1.06 2.21 2.22 2.13 2.05 2.21 12.44 3.07
f1 (ppm)

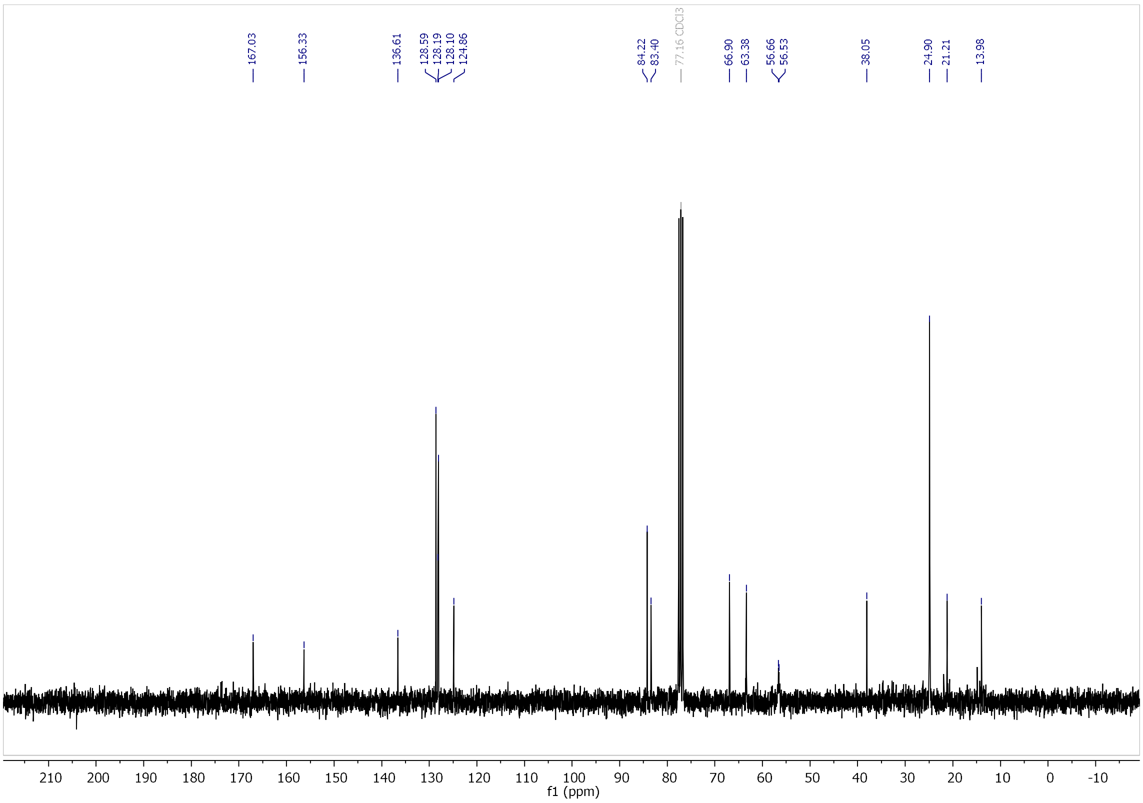
167.03
156.33
136.61 128.59 128.19 128.10 124.86
84.22 83.40
77.16 CDCl3
66.90 63.38 56.66 56.53
38.05
24.90 21.21 13.98
f1 (ppm)

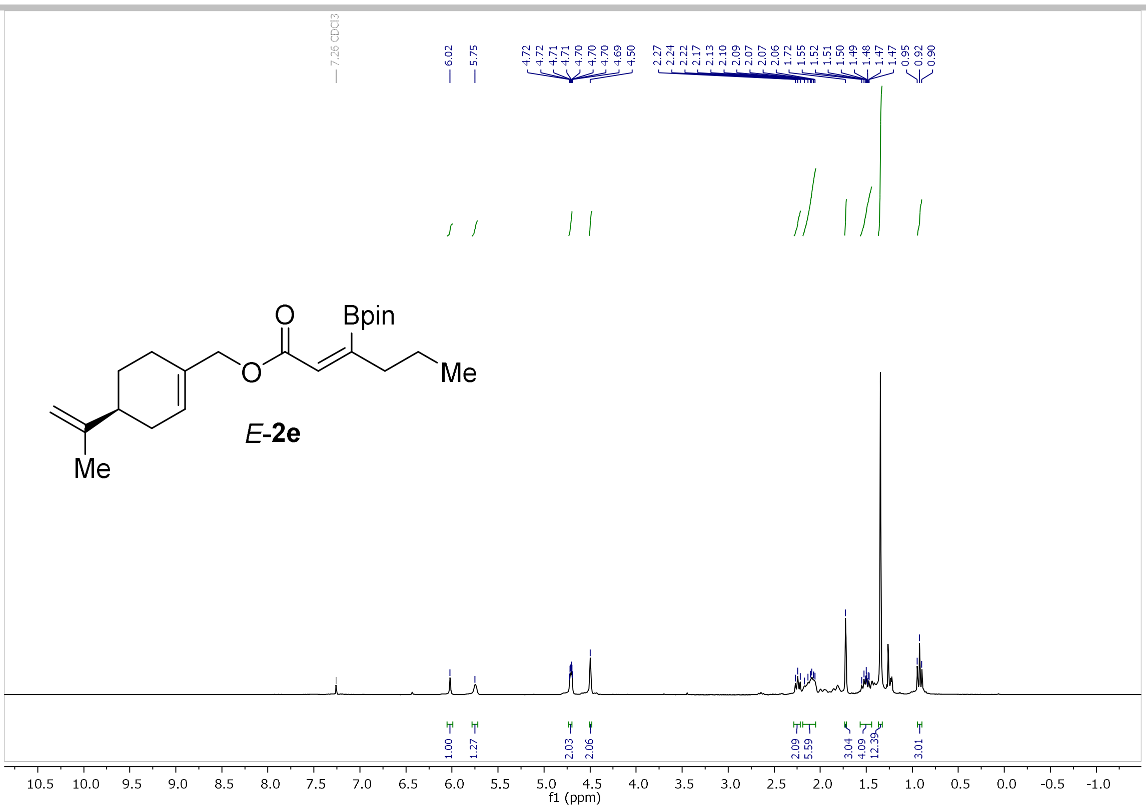
Bpin
O
O
Me
Me
E-2e
f1 (ppm)

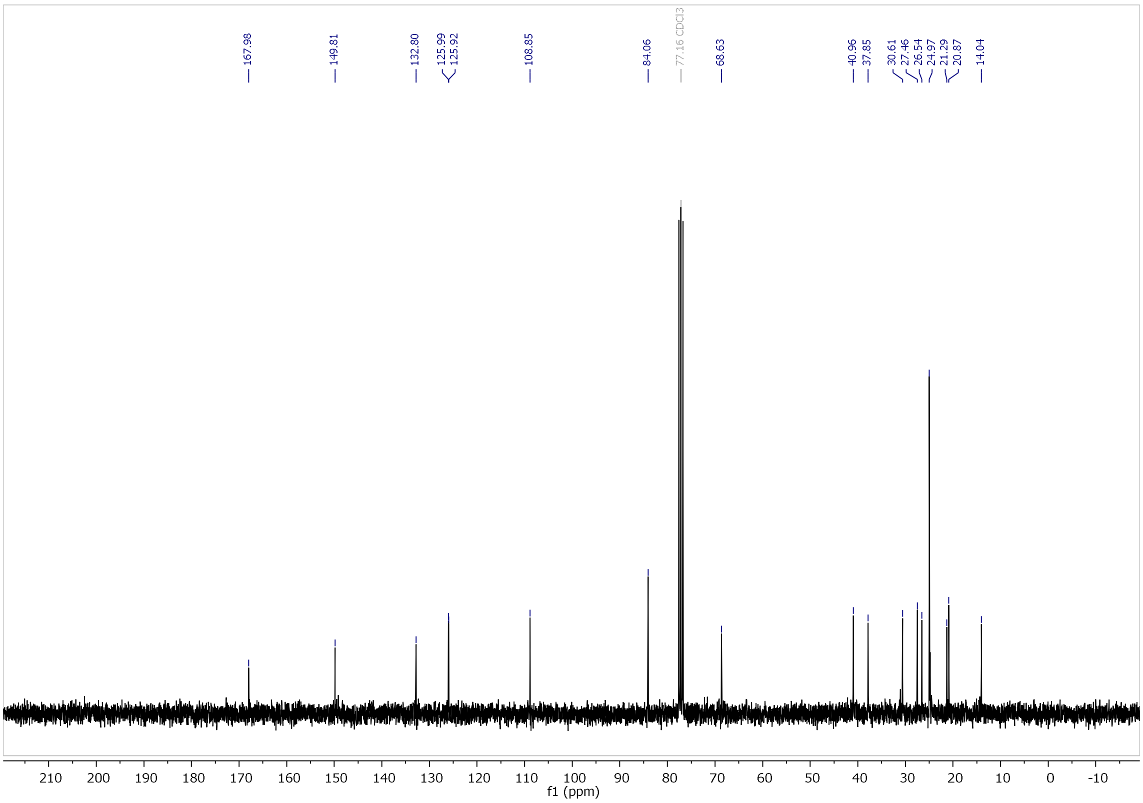
f1 (ppm)

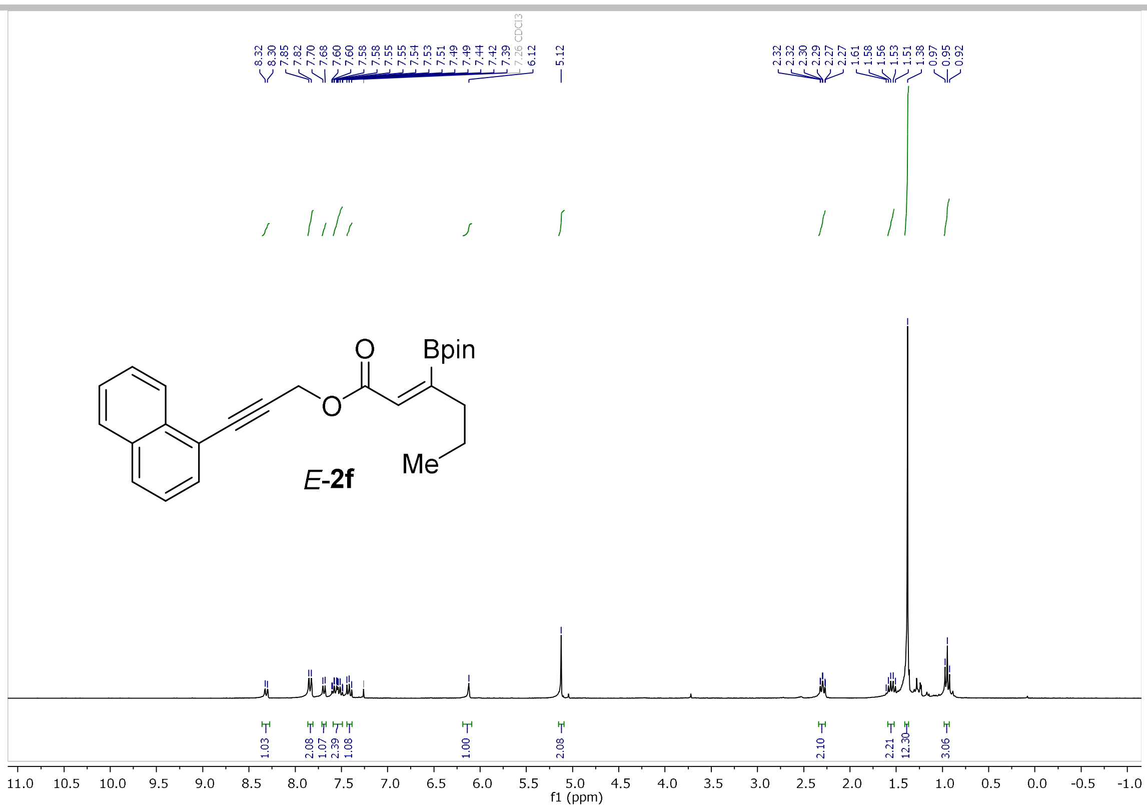

Bpin
O
O
Me
E-**2f**
f1 (ppm)

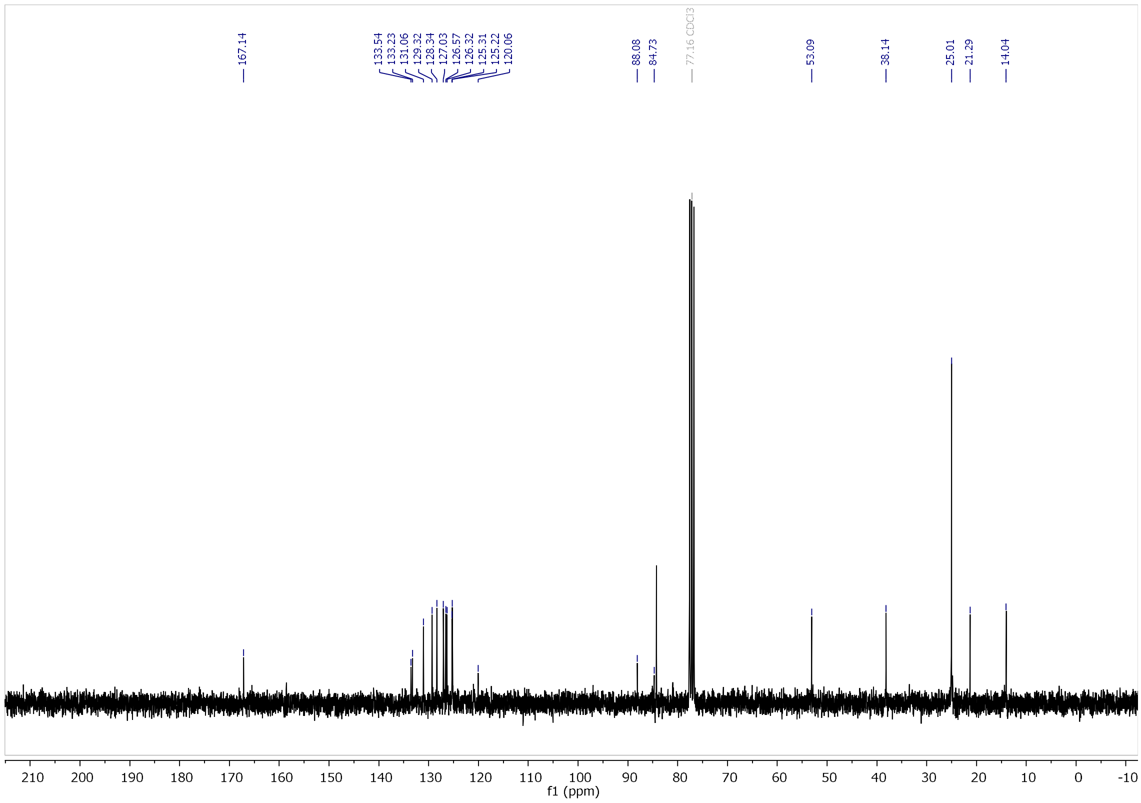

f1 (ppm)

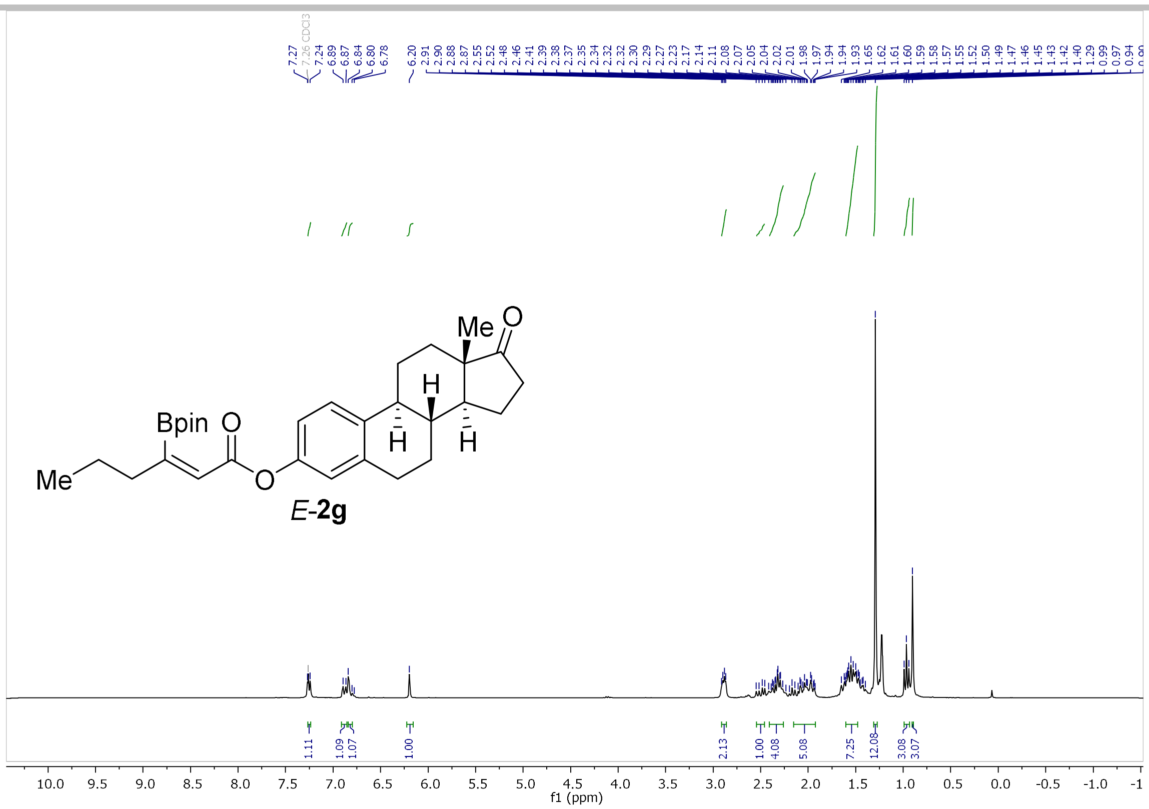

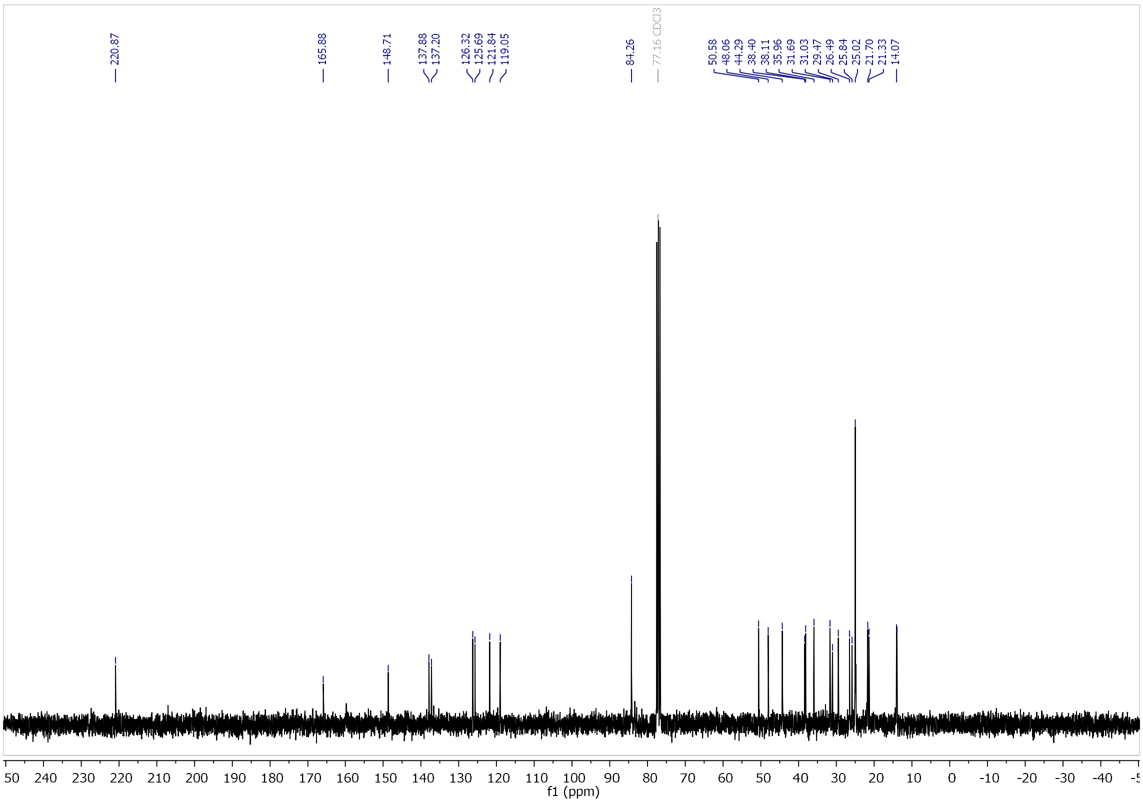

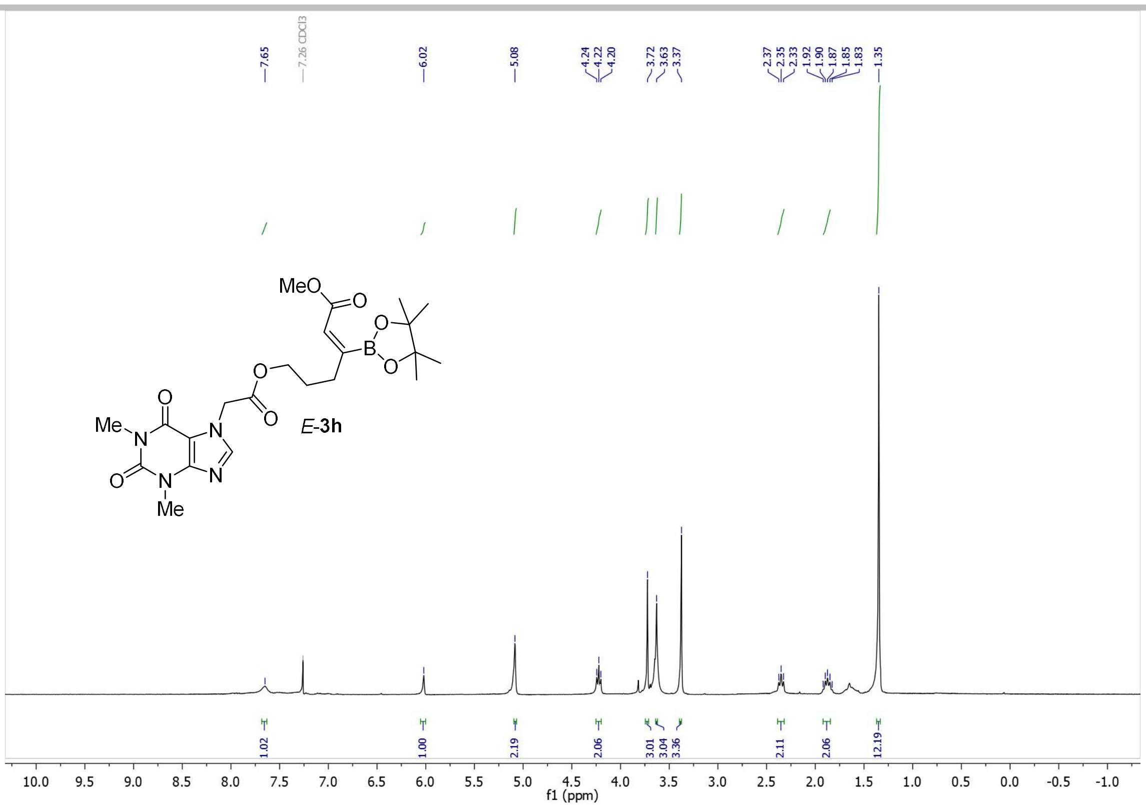
7.65
7.26 CDCl3
6.02
5.08
4.24
4.22
4.20
3.72
3.63
3.37
2.37
2.35
2.33
1.92
1.90
1.87
1.85
1.83
1.35
MeO
O
O
B
O
O
O
Me
N
O
N
N
O
N
Me
E-3h
1.02
1.00
2.19
2.06
3.01
3.04
3.36
2.11
2.06
12.19
f1 (ppm)

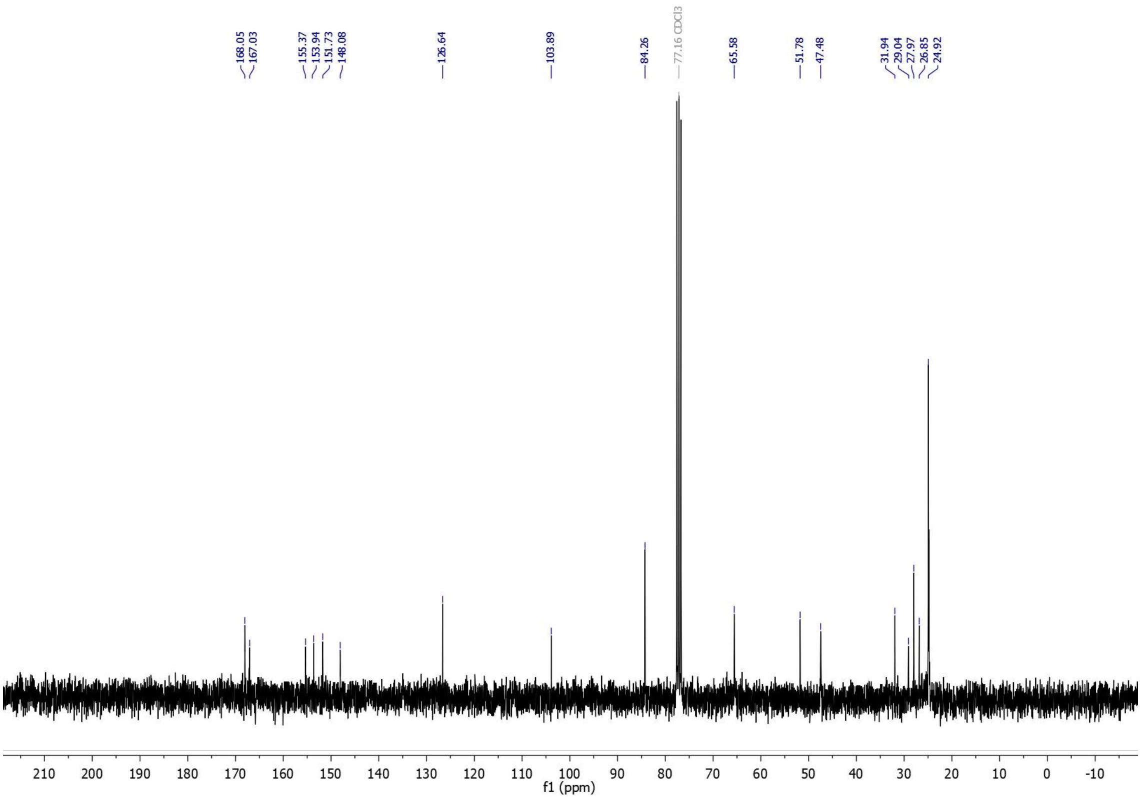
168.05
167.03
155.37
153.94
151.73
148.08
126.64
103.89
84.26
77.16 CDCl3
65.58
51.78
47.48
31.94
29.04
27.97
26.85
24.92
f1 (ppm)

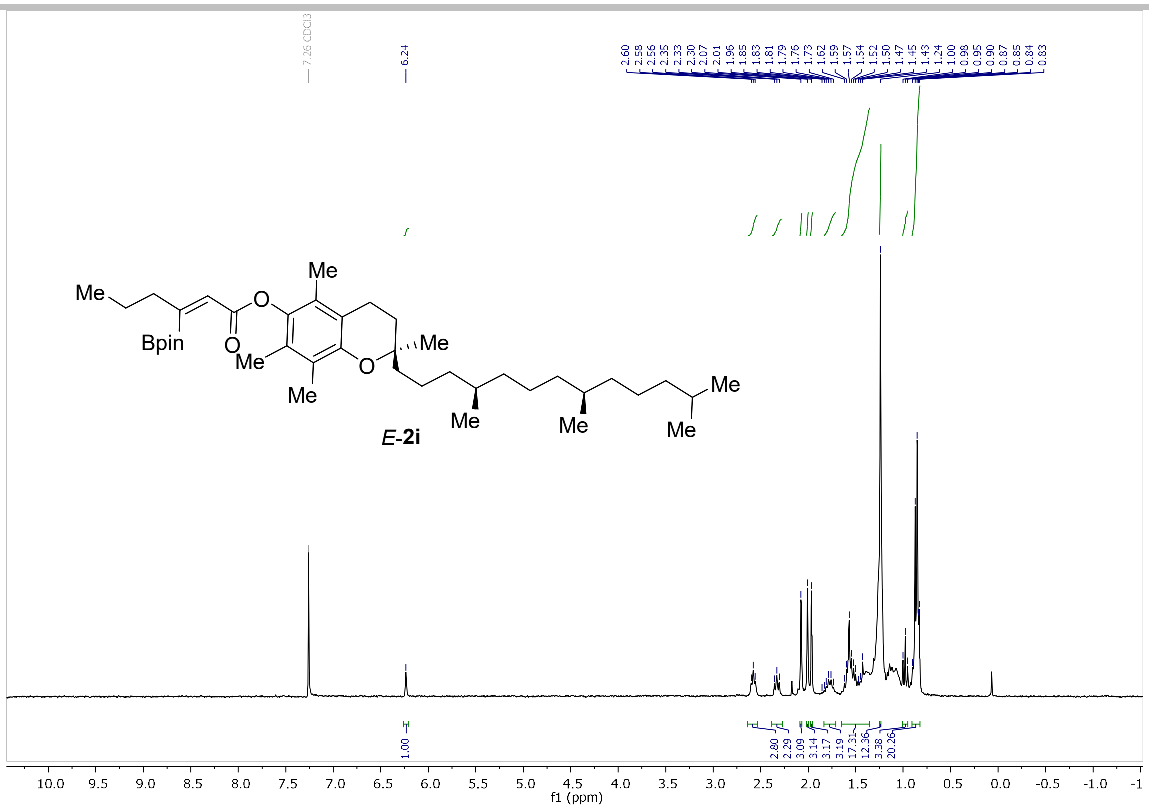

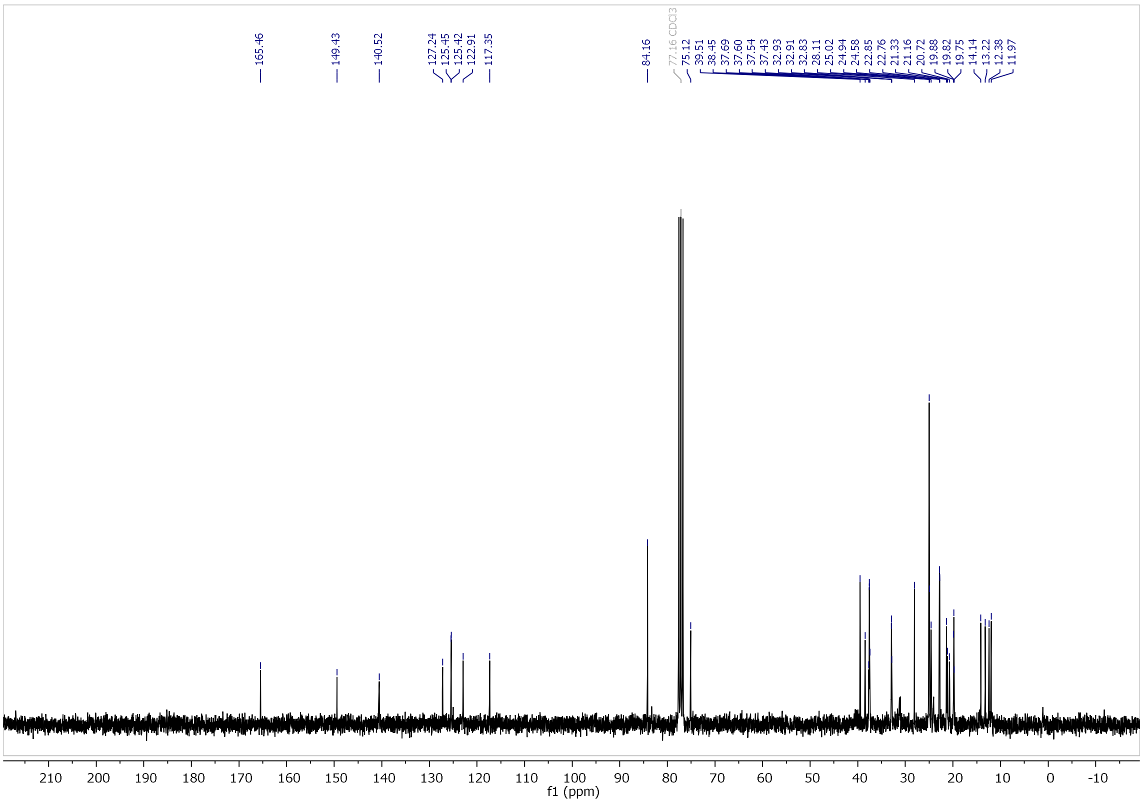

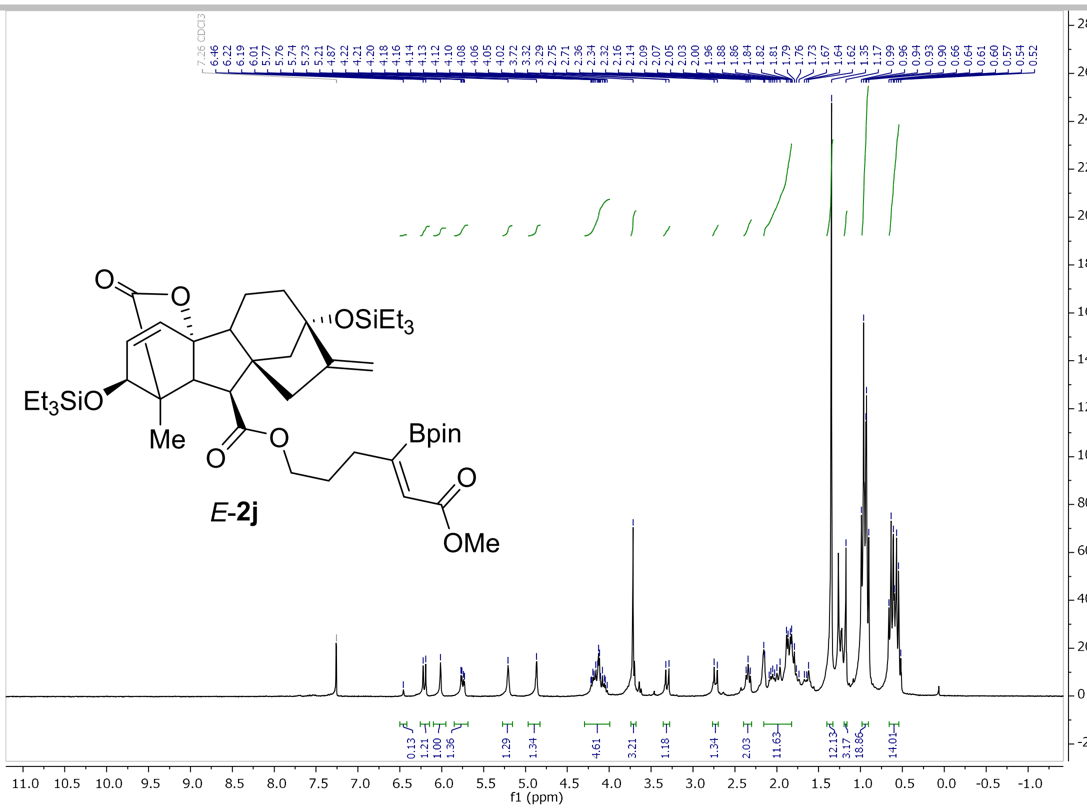

E-2j

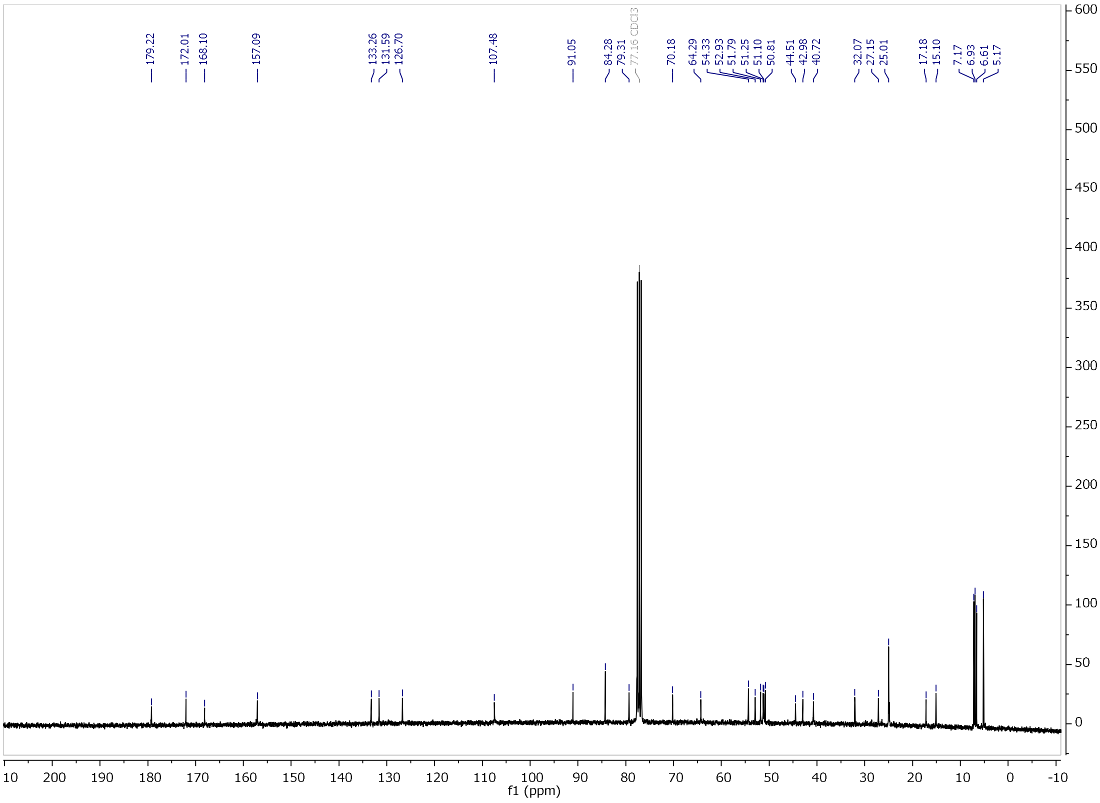

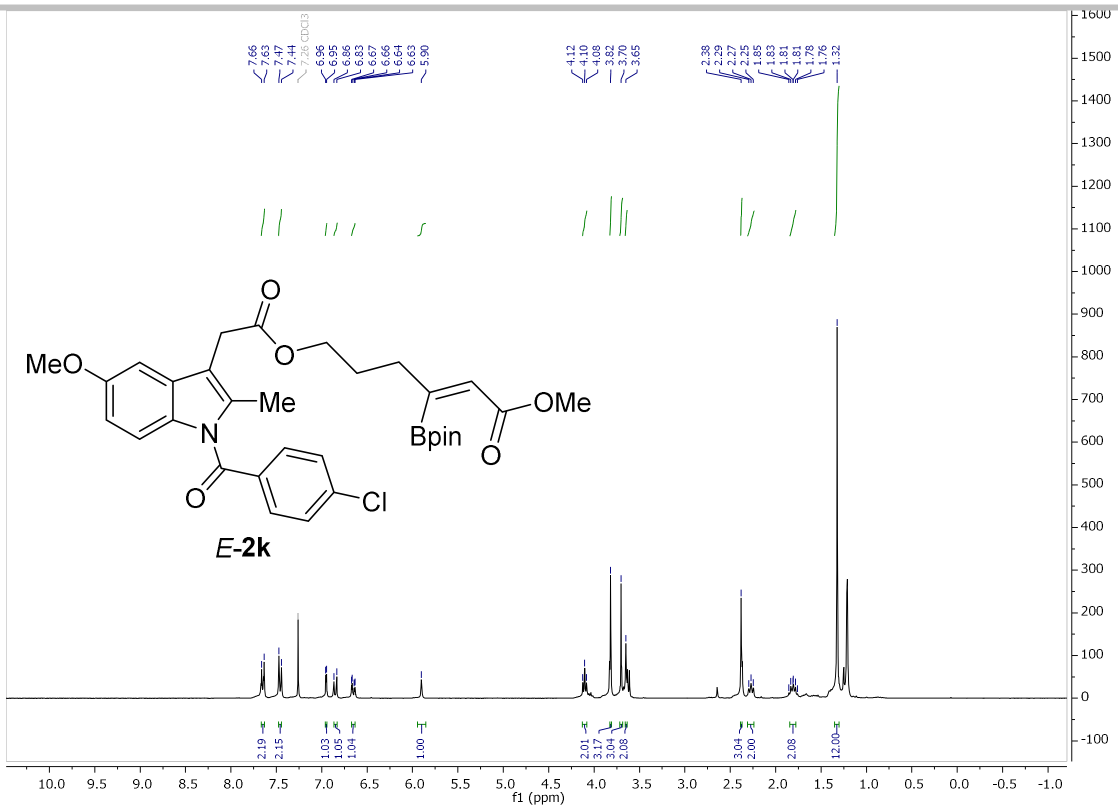
MeO
Me
N
O
Cl
O
O
Bpin
OMe
O
E-2k

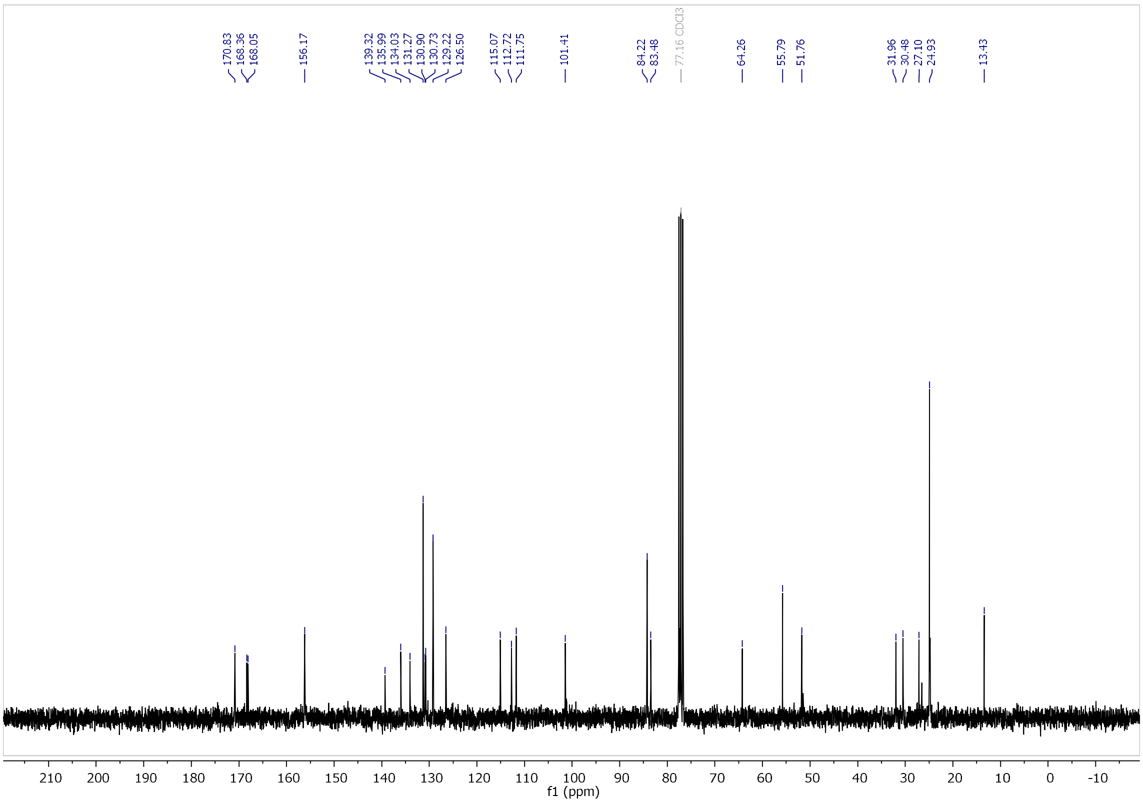

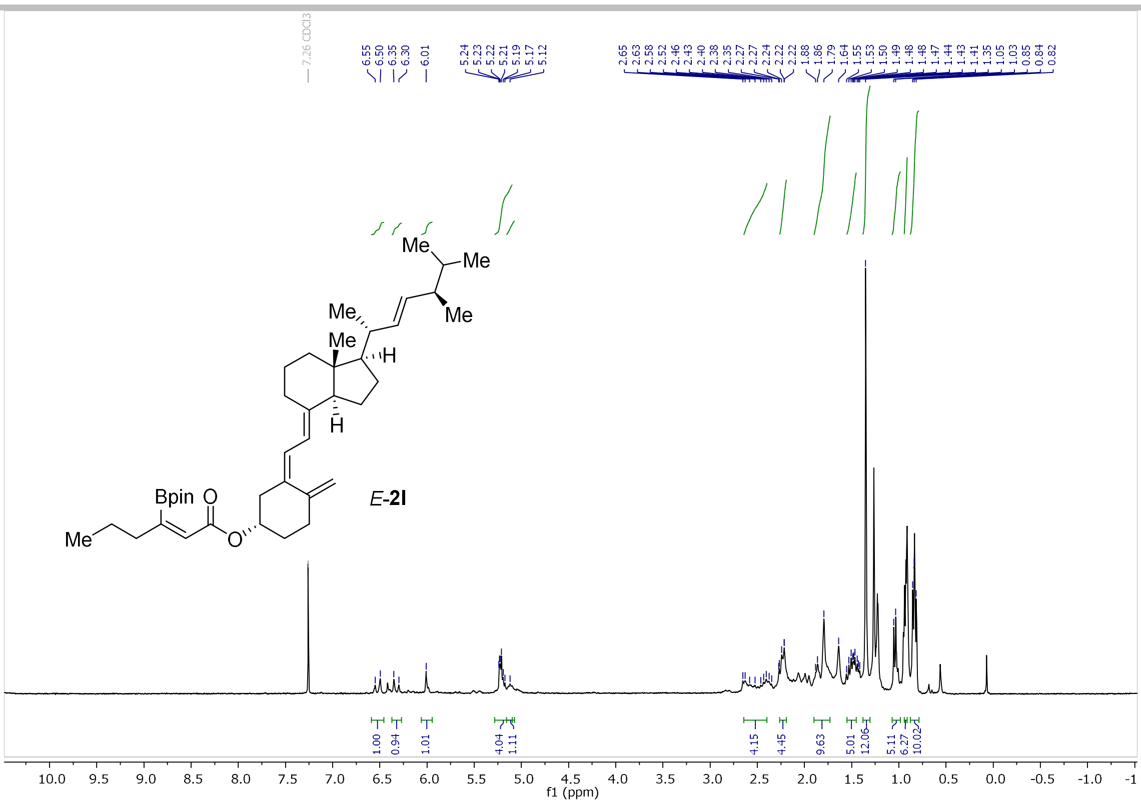
7.26 CDCl3
6.55
6.50
6.35
6.30
6.01
5.24
5.23
5.22
5.21
5.19
5.17
5.12
2.65
2.63
2.58
2.52
2.46
2.43
2.40
2.38
2.35
2.27
2.27
2.24
2.22
2.22
1.88
1.86
1.79
1.64
1.55
1.53
1.50
1.49
1.48
1.48
1.47
1.44
1.43
1.41
1.35
1.05
1.03
0.85
0.84
0.82
Me
Me
Me
Me
Me
H
H
Bpin
O
Me
O
E-2l
1.00
0.94
1.01
4.04
1.11
4.15
4.45
9.63
5.01
12.06
5.11
6.27
10.02
10.0 9.5 9.0 8.5 8.0 7.5 7.0 6.5 6.0 5.5 5.0 4.5 4.0 3.5 3.0 2.5 2.0 1.5 1.0 0.5 0.0 -0.5 -1.0
f1 (ppm)

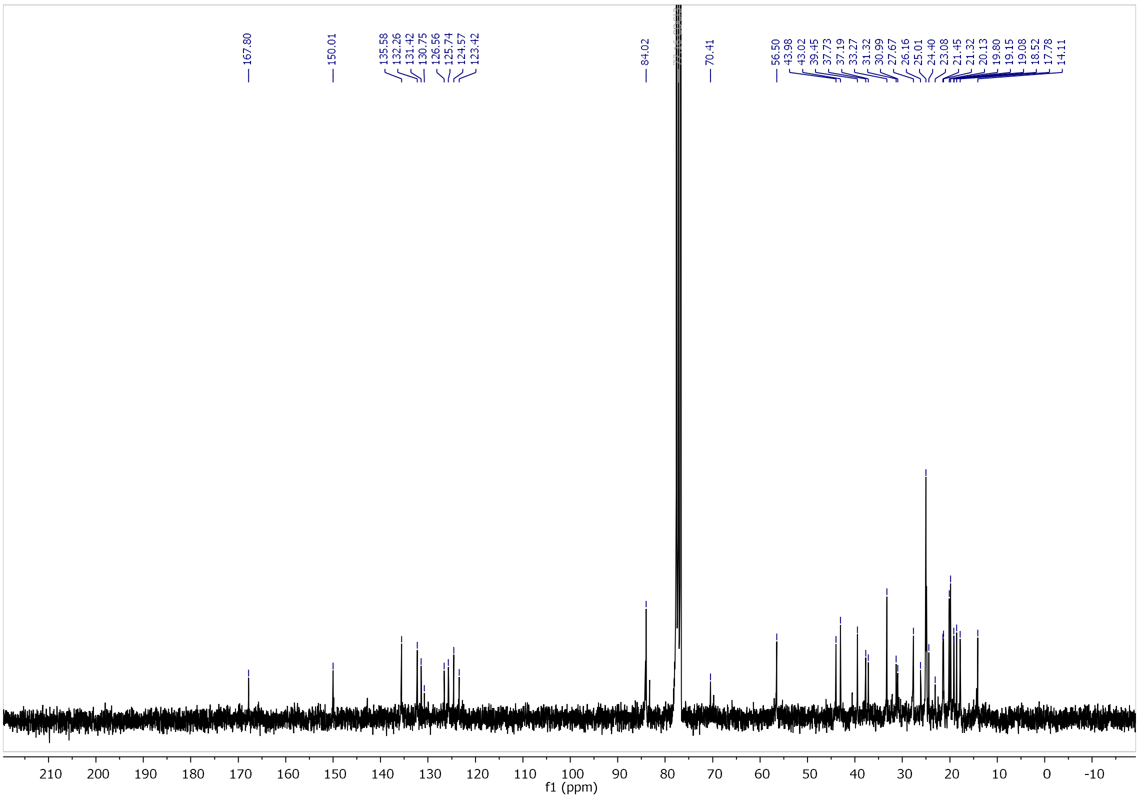
167.80
150.01
135.58
132.26
131.42
130.75
126.56
125.74
124.57
123.42
84.02
70.41
56.50
43.98
43.02
39.45
37.73
37.19
33.27
31.32
30.99
27.67
26.16
25.01
24.40
23.08
21.45
21.32
20.13
19.80
19.15
19.08
18.52
17.78
14.11
210 200 190 180 170 160 150 140 130 120 110 100 90 80 70 60 50 40 30 20 10 0 -10
f1 (ppm)

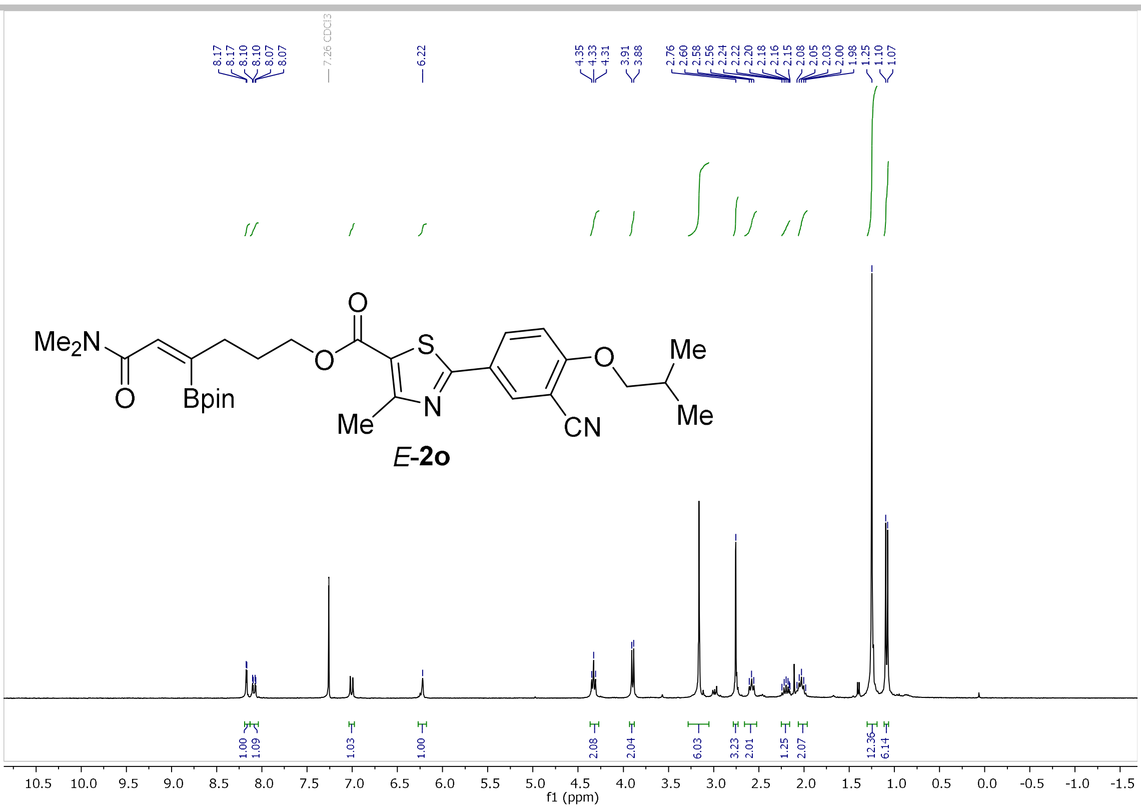
Me2N
O
Bpin
O
O
S
N
Me
CN
O
Me
Me
E-2o
f1 (ppm)

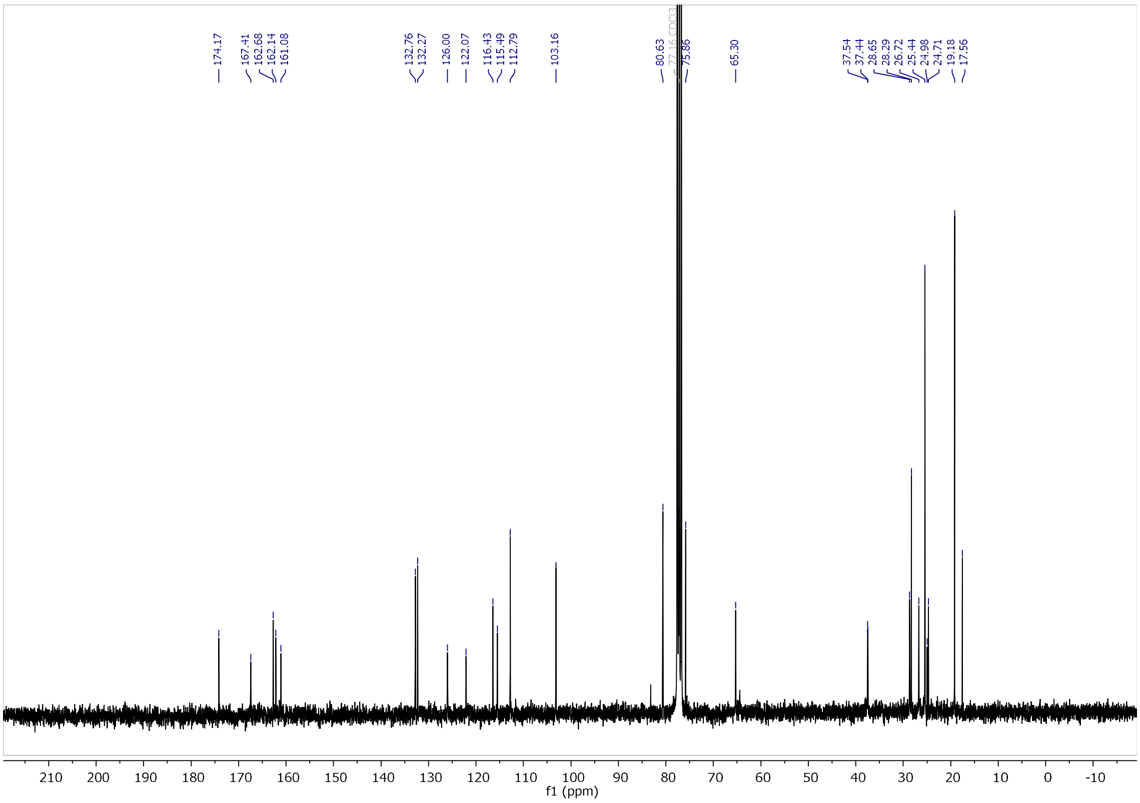
f1 (ppm)

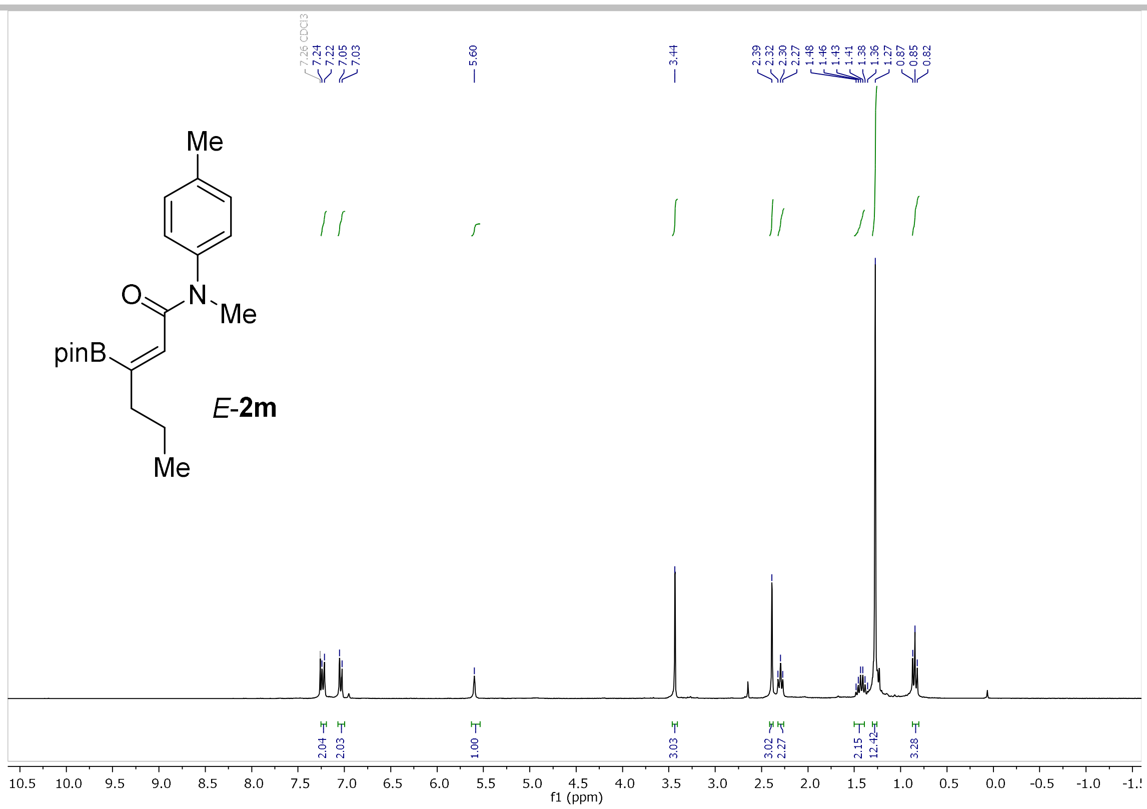
7.26 CDCl3
7.24
7.22
7.05
7.03
5.60
3.44
2.39
2.32
2.30
2.27
1.48
1.46
1.43
1.41
1.38
1.36
1.27
0.87
0.85
0.82
Me
O
N
Me
pinB
E-2m
Me
2.04
2.03
1.00
3.03
3.02
2.27
2.15
12.42
3.28
10.5 10.0 9.5 9.0 8.5 8.0 7.5 7.0 6.5 6.0 5.5 5.0 4.5 4.0 3.5 3.0 2.5 2.0 1.5 1.0 0.5 0.0 -0.5 -1.0
f1 (ppm)

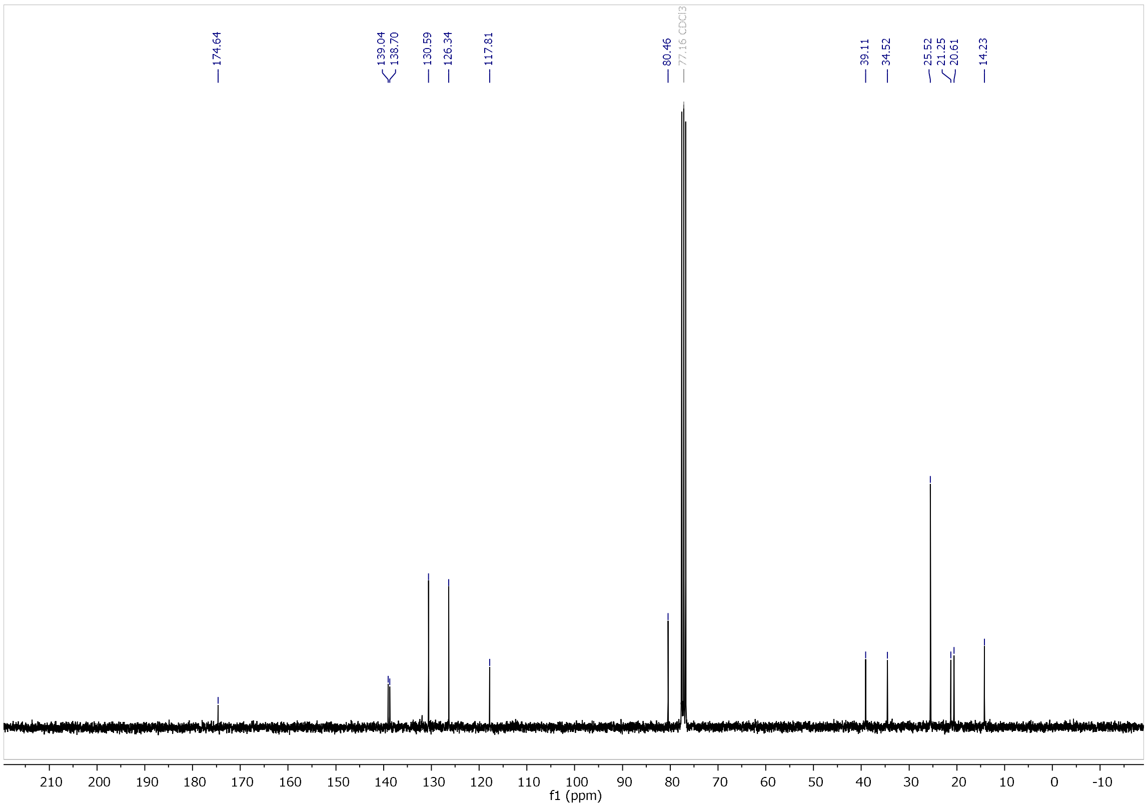
174.64
139.04
138.70
130.59
126.34
117.81
80.46
77.16 CDCl3
39.11
34.52
25.52
21.25
20.61
14.23
210 200 190 180 170 160 150 140 130 120 110 100 90 80 70 60 50 40 30 20 10 0 -10
f1 (ppm)

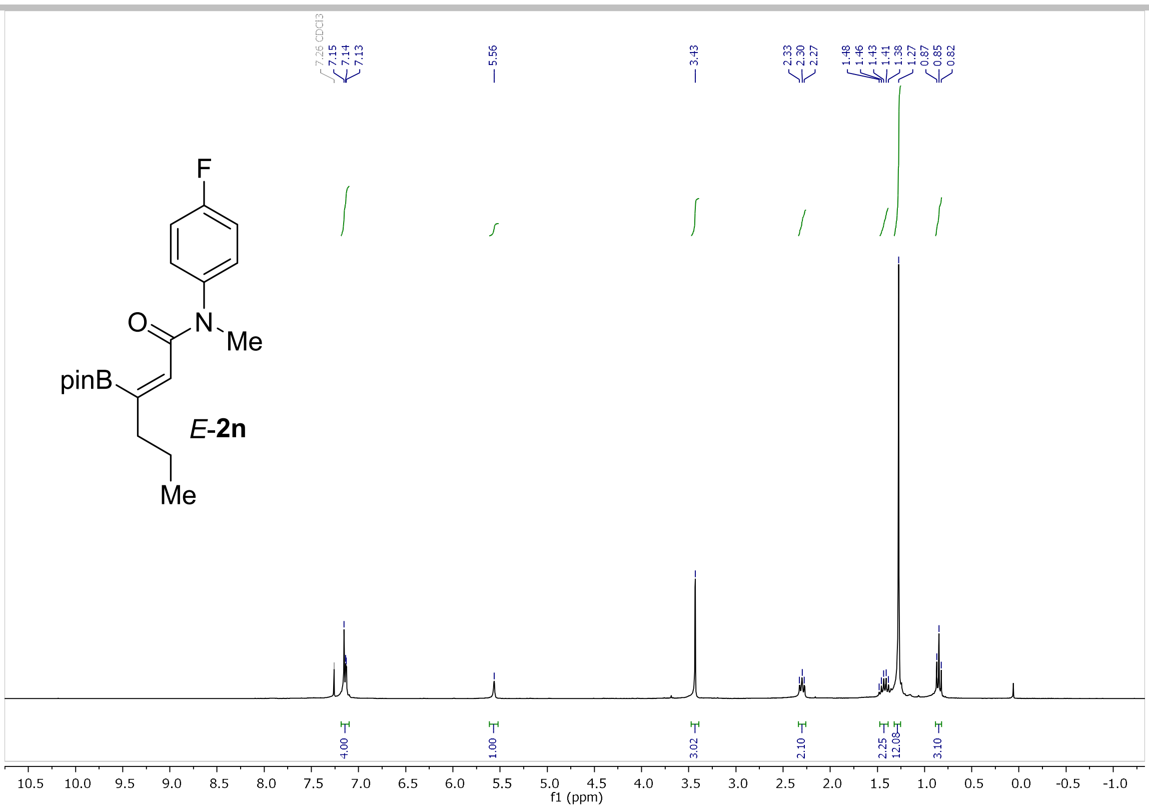
F
O
N
Me
pinB
E-2n
Me
7.26 CDCl3
7.15
7.14
7.13
5.56
3.43
2.33
2.30
2.27
1.48
1.46
1.43
1.41
1.38
1.27
0.87
0.85
0.82
4.00
1.00
3.02
2.10
2.25
12.08
3.10
f1 (ppm)

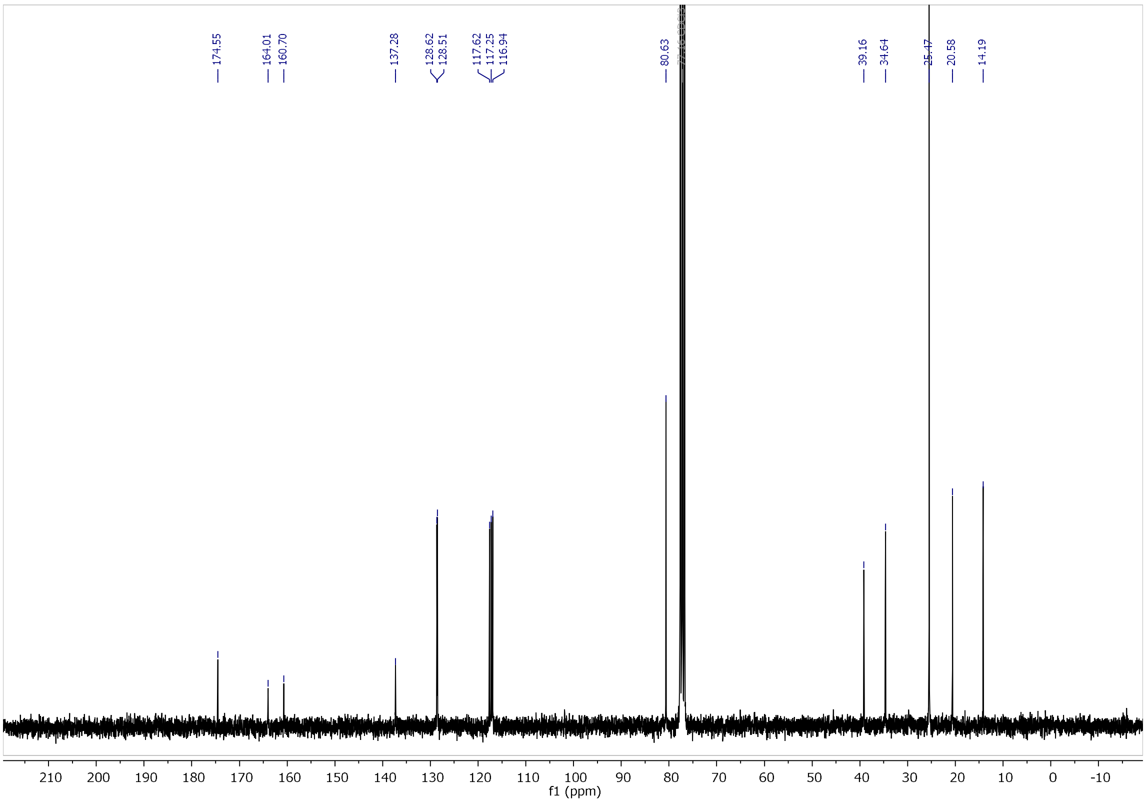
174.55
164.01
160.70
137.28
128.62
128.51
117.62
117.25
116.94
80.63
39.16
34.64
25.47
20.58
14.19
f1 (ppm)

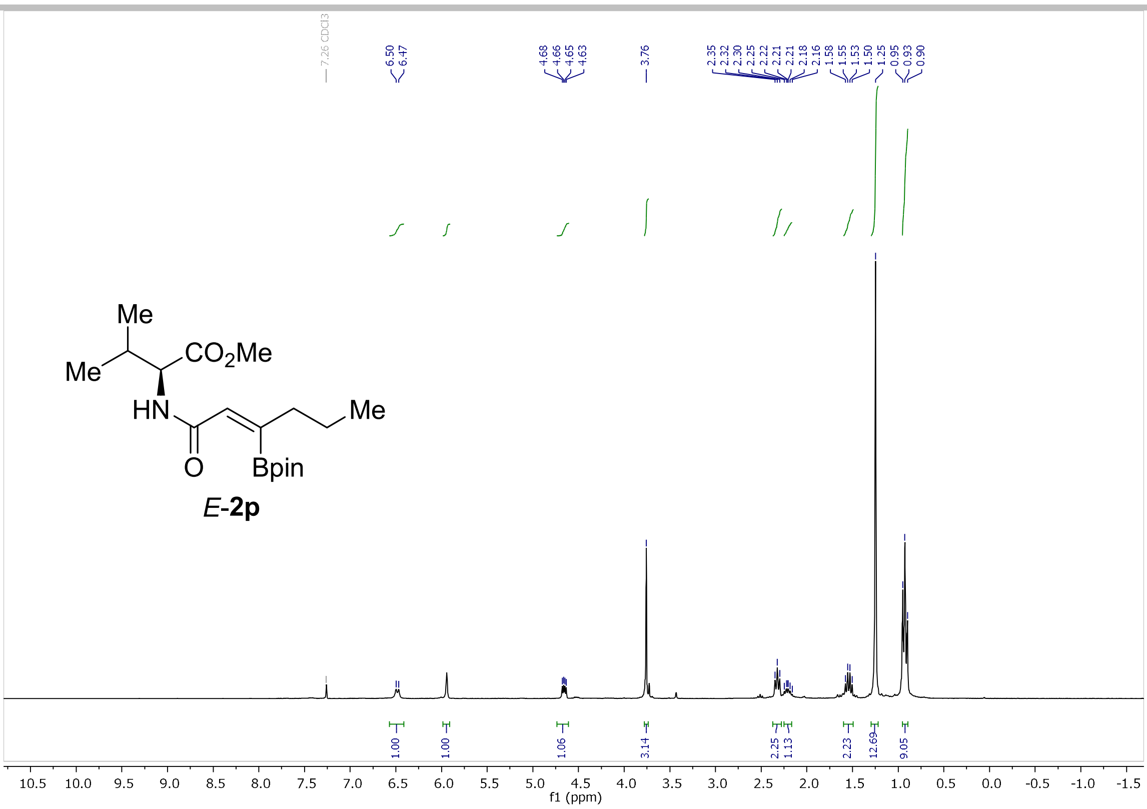
7.26 CDCl3
6.50
6.47
4.68
4.66
4.65
4.63
3.76
2.35
2.32
2.30
2.25
2.22
2.21
2.21
2.18
2.16
1.58
1.55
1.53
1.50
1.25
0.95
0.93
0.90
Me
CO2Me
Me
HN
Me
O
Bpin
E-2p
1.00
1.00
1.06
3.14
2.25
1.13
2.23
12.69
9.05
10.5 10.0 9.5 9.0 8.5 8.0 7.5 7.0 6.5 6.0 5.5 5.0 4.5 4.0 3.5 3.0 2.5 2.0 1.5 1.0 0.5 0.0 -0.5 -1.0 -1.5
f1 (ppm)

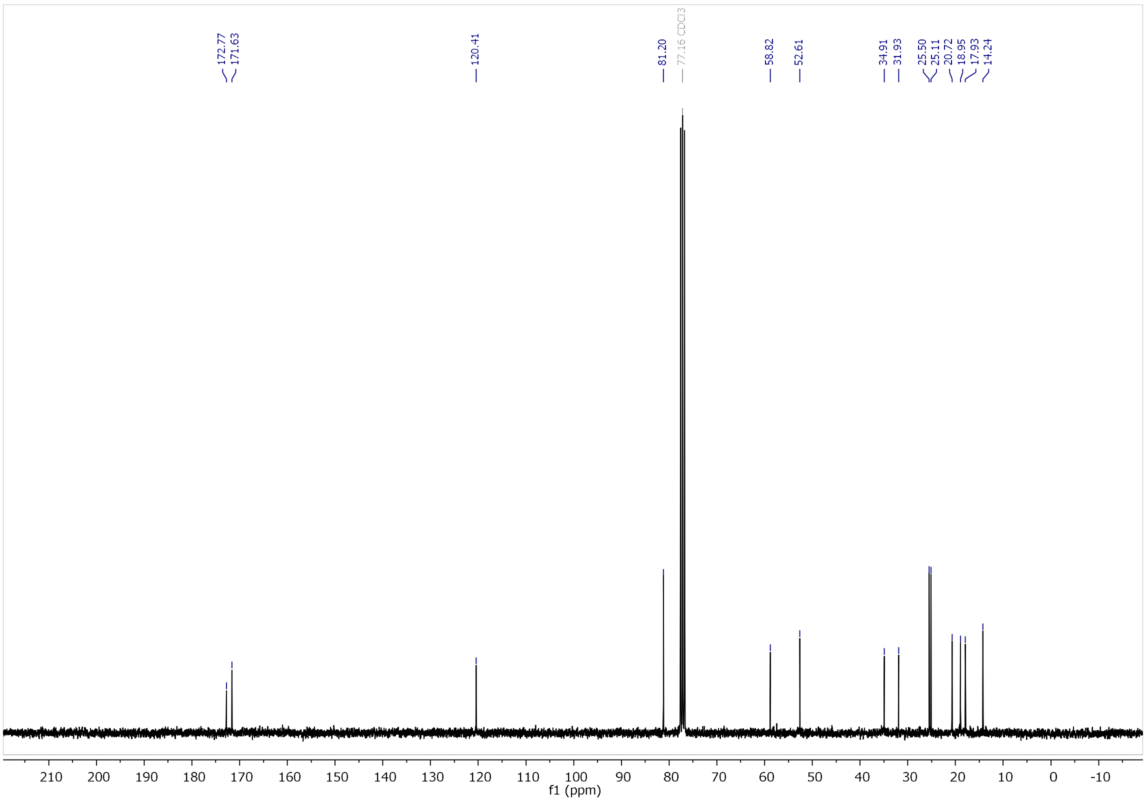
172.77
171.63
120.41
81.20
77.16 CDCl3
58.82
52.61
34.91
31.93
25.50
25.11
20.72
18.95
17.93
14.24
210 200 190 180 170 160 150 140 130 120 110 100 90 80 70 60 50 40 30 20 10 0 -10
f1 (ppm)

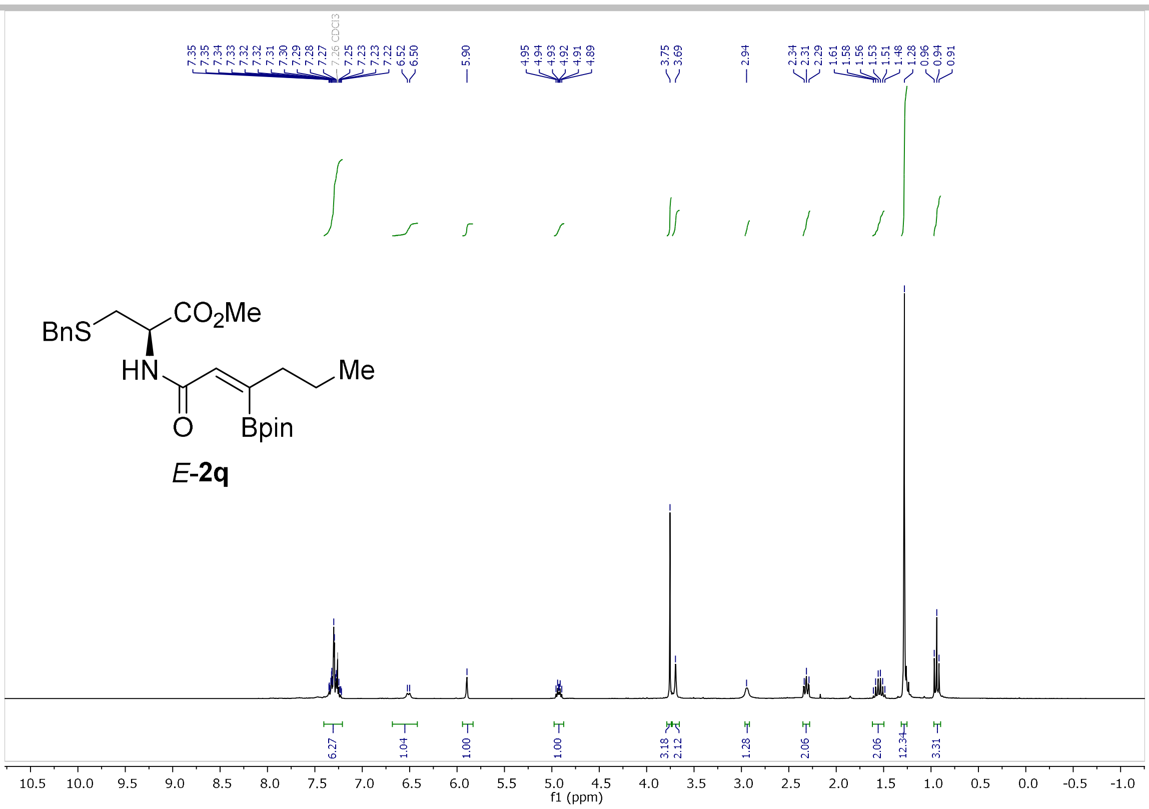
BnS
CO2Me
HN
Me
O
Bpin
E-2q
f1 (ppm)

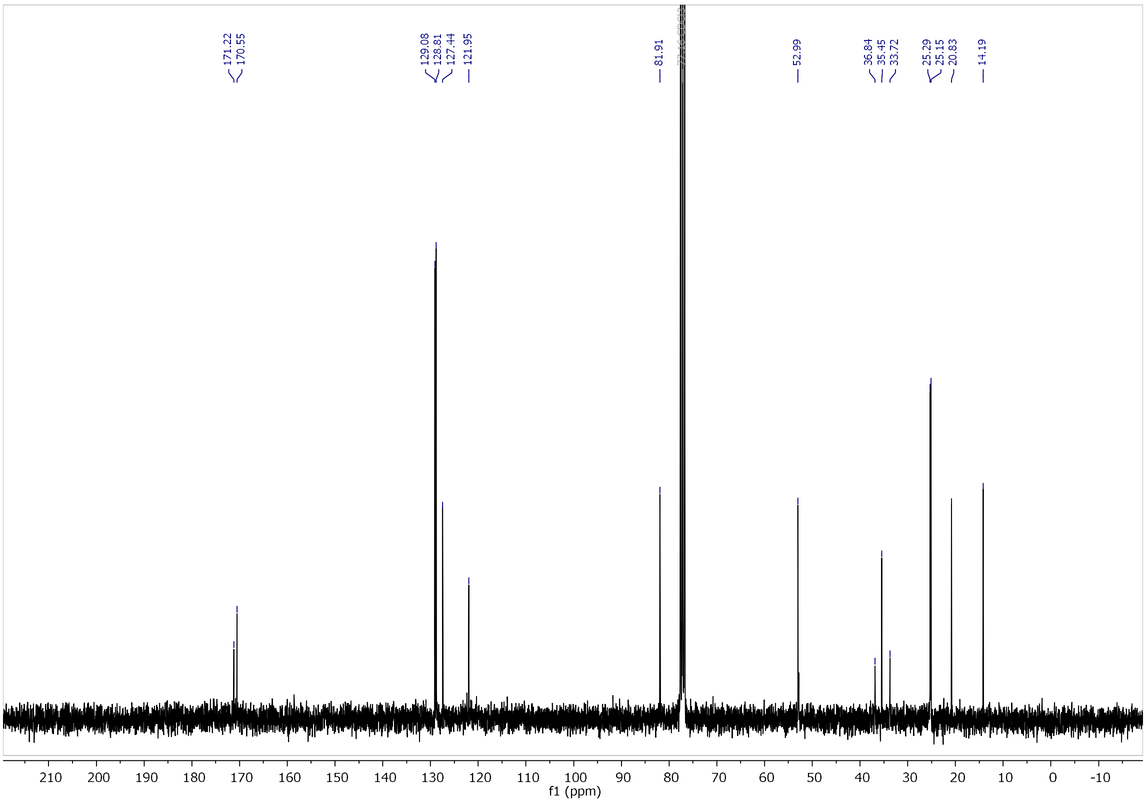
171.22
170.55
129.08
128.81
127.44
121.95
81.91
52.99
36.84
35.45
33.72
25.29
25.15
20.83
14.19
f1 (ppm)
